# The 2015 Annual Meeting of SETAC German Language Branch in Zurich (7–10 September, 2015): Ecotoxicology and environmental chemistry—from research to application

**DOI:** 10.1186/s12302-016-0088-3

**Published:** 2016-07-06

**Authors:** Inge Werner, Annette Aldrich, Benjamin Becker, Dennis Becker, Markus Brinkmann, Michael Burkhardt, Norbert Caspers, Sophie Campiche, Nathalie Chèvre, Rolf-Alexander Düring, Beate I. Escher, Fabian Fischer, Sabrina Giebner, Katharina Heye, Henner Hollert, Marion Junghans, Cornelia Kienle, Katja Knauer, Muris Korkaric, Veronika Märkl, Jane Muncke, Jörg Oehlmann, Georg Reifferscheid, Daniel Rensch, Andreas Schäffer, Sabrina Schiwy, Simon Schwarz, Helmut Segner, Eszter Simon, Rita Triebskorn, Etiënne L. M. Vermeirssen, Thomas Wintgens, Markus Zennegg

**Affiliations:** 1 Swiss Centre for Applied Ecotoxicology Eawag-EPFL, Überlandstrasse 133, 8600 Dübendorf, Switzerland; 2 Agroscope, Schloss 1, Postfach, 8820 Wädenswil, Switzerland; 3 Federal Institute of Hydrology, Am Mainzer Tor 1, 56068 Koblenz, Germany; 4 Goethe University Frankfurt Am Main, Max-von-Laue-Str. 13, 60438 Frankfurt, Germany; 5 RWTH Aachen University, Worringerweg 1, 52074 Aachen, Germany; 6 HSR, University of Applied Sciences, Institute of Environmental and Process Engineering UMTEC, Oberseestrasse 10, 8640 Rapperswil, Switzerland; 7 Eco(toxico)consulting, Sankt-Maternus-Eck 14, 51515 Kürten, Germany; 8 Faculty of Geosciences and Environment, University of Lausanne, 1015 Lausanne, Switzerland; 9 Justus-Liebig-University, Institute of Soil Science, IFZ, Heinrich-Buff-Ring 26-32, 35392 Giessen, Germany; 10 Cell Toxicology, Helmholtz Centre for Environmental Research (UFZ), 04318 Leipzig, Germany; 11 Environmental Toxicology, Center for Applied Geosciences, Eberhard Karls University Tübingen, 72074 Tübingen, Germany; 12 Department Cell Toxicology, Helmholtz Centre for Environmental Research (UFZ), Permoserstraße 15, 04318 Leipzig, Germany; 13 Federal Office for Agriculture, 3003 Berne, Switzerland; 14 Department of Civil Engineering, Institute of Building Materials and Construction Chemistry, Technical University Berlin, Gustav-Meyer-Allee 25, 13355 Berlin, Germany; 15 Food Packaging Forum Foundation, Staffelstrasse 8, 8045 Zurich, Switzerland; 16 Office of Waste, Water, Energy and Air (AWEL), Hardturmstrasse 105, Zurich, Switzerland; 17 Animal Physiological Ecology, University of Tübingen, Auf der Morgenstelle 5, 72076 Tübingen, Germany; 18 Centre for Fish and Wildlife Health, University of Berne, 3000 Berne, Switzerland; 19 University of Applied Sciences and Arts Northwestern Switzerland, Gründenstrasse 40, Muttenz, Switzerland; 20 Laboratory for Advanced Analytical Technologies, Swiss Federal Laboratories for Materials Science and Technology (Empa), Dübendorf, Switzerland

## Abstract

This report provides a brief review of the 20th annual meeting of the German Language Branch of the Society of Environmental Toxicology and Chemistry (SETAC GLB) held from September 7th to 10th 2015 at ETH (Swiss Technical University) in Zurich, Switzerland. The event was chaired by Inge Werner, Director of the Swiss Centre for Applied Ecotoxicology (Ecotox Centre) Eawag-EPFL, and organized by a team from Ecotox Centre, Eawag, Federal Office of the Environment, Federal Office of Agriculture, and Mesocosm GmbH (Germany). Over 200 delegates from academia, public agencies and private industry of Germany, Switzerland and Austria attended and discussed the current state of science and its application presented in 75 talks and 83 posters. In addition, three invited keynote speakers provided new insights into scientific knowledge ‘brokering’, and—as it was the International Year of Soil—the important role of healthy soil ecosystems. Awards were presented to young scientists for best oral and poster presentations, and for best 2014 master and doctoral theses. Program and abstracts of the meeting (mostly in German) are provided as Additional file 1.

**Electronic supplementary material:**

The online version of this article (doi:10.1186/s12302-016-0088-3) contains supplementary material, which is available to authorized users.

## Review

The 20th Annual Meeting of SETAC GLB took place at ETH Zurich, Switzerland, organized under the auspices of the Swiss Centre for Applied Ecotoxicology (Ecotox Centre Eawag-EPFL) and chaired by its Director, Dr. Inge Werner. Over 200 delegates from academia, public agencies and private industry of Germany, Switzerland and Austria attended and discussed the current state of science and its application presented in 75 talks and 83 posters. In addition, three invited keynote speakers provided new insights. In her plenary talk, Prof. Janet Hering, the Director of Eawag in Dübendorf, Switzerland, emphasized the increasing need for “knowledge brokers” to ensure a successful transfer of scientific information to practitioners and politicians. She called on her audience to face this important challenge. Dr. Michael Schärer, Head of the Water Body Protection Section at the Swiss Federal Office of the Environment in Berne, presented an outstanding example of knowledge brokering and transfer: the recent success story of reducing micropollutant loads in rivers of Switzerland by improving wastewater treatment nationwide. In honor of the International Year of Soil, Prof. Emmanuel Frossard of ETH Zurich provided insight into the great importance of this ecosystem and of maintaining and restoring soil quality and health.

The scientific program featured 18 sessions. “Classical” topics included aquatic, sediment, terrestrial and soil ecotoxicology, monitoring of surface waters, environmental chemistry, risk assessment of pesticides, endocrine disruption and wastewater treatment and toxicity. Strongly practice-oriented topics such as ‘ecotoxicology of construction materials’, ‘bioassay standardization procedures’ and ‘application of passive samplers in surface water monitoring’ met with considerable interest; so did “emerging” topics like ‘multiple stressor effects’ and ‘microplastics in the aquatic environment’. The following is a brief summary of the science presented in those sessions. The full program and abstracts are available as Additional file 1.

### Session 1: Ecotoxicology of construction chemicals (chairs: V. Märkl, M. Burkhardt)

Ecotoxicology of construction chemicals is a new interdisciplinary field of research. An increasing variety and large amounts of organic additives are used in construction materials. Under the recommended conditions for their application, chemicals are released into the environment. Monitoring of certain constituents demonstrated their presence in soil, groundwater and surface water. Under European Regulations (e.g., Biocidal Products Regulation, Construction Materials Regulation, REACH) their environmental risk must be evaluated. Leaching experiments (e.g., tank test, immersion test) can be used to analyze target chemicals in eluates, and the toxicity of mixtures can be measured in bioassays; however, methods to better transfer laboratory results to field conditions are still needed.

Ute Schoknecht (Bundesanstalt für Materialforschung und—prüfung, Berlin, Germany) started the session presenting results on the leaching of biocides from coatings and textiles. The test series included laboratory as well as outdoor tests lasting up to 2 years. Degradation and volatilization of organic compounds played an important role; therefore, laboratory tests tended to overestimate emissions in the field. Michael Burkhardt (HSR University of Applied Sciences, Rapperswil, Switzerland) presented results of a transport model and environmental risk assessment for leaching of biocides from house facades. A dynamic simulation run with real weather data estimated the daily emission for different scenarios over a 5-year period. Field data for storm runoff (i.e., measured driving rain) and leaching from coatings were used for calibration and validation of the model. The transport model was an excellent tool to predict surface water contamination originating from such building materials. It will be made available as a user-friendly software, COMLEAM, by 2016. Results on the ecotoxicity of leachates from epoxy resins used as organic coatings for steel construction were presented by Etienne Vermeirssen (Ecotox Centre Eawag-EPFL, Dübendorf, Switzerland). Four commercial products were tested. The eluate of one product affected bacterial bioluminescence and that of a second product was estrogenic due to elevated bisphenol A concentrations. Such results may encourage producers to change their product formulation, and consumers to choose the least harmful product; however, the hazard they pose under realistic conditions needs to be investigated. The session closed with a presentation of Veronika Märkl (Technical University Berlin, Institute of Building Materials and Construction Chemistry, Berlin, Germany) on two leaching tests for polyurethane resins used in grouting. A new tank leaching test was compared with the percolation upflow test. No significant differences were seen when eluate toxicity was tested for photosynthesis inhibition of algae. Additional bioassays will be applied to get more comprehensive information on ecotoxicological effects. Several posters presented modeling results on leaching behavior of organic biocides from coating materials. It was shown that biocides can be released in toxic concentrations from renders. The importance of “driving rain” for the amount of chemicals released was highlighted. The authors stressed the need for identifying the sources of urban pollutants and creating a typology of factors relevant to the aquatic footprint of mixtures.

The session reflected the complexity of this new area of research. There is a broad spectrum of building materials, among them biocides from facades and polyurethanes for waterproofing. All authors underlined the importance of measuring and assessing the environmental risk of inorganic and organic chemicals released from building materials. Here, bioassays are the tools of choice due to the analytical constraints with regard to unknown chemical mixtures and the cost of applying non-target analytics to eluates. In addition, modeling approaches will be needed to satisfy regulatory requirements.

### Session 2: Standardization of bioassays (chairs: N. Caspers, G. Reifferscheid)

The primary objective of this session was to describe and discuss the importance of national and international standardization to ensure the development and provision of high-quality and up-to-date methods for chemical and environmental effect monitoring. Special emphasis was put on raising the awareness, especially of the young audience, for the practical relevance of this topic. The session was opened by Norbert Caspers [Eco (toxico) consulting, Kürten, Germany] who described structures, processes and stakeholders in national and international standardization. Goals, tasks and deliverables of DIN (Deutsches Institut für Normung) in national standardization processes as well as in the representation of national interests at ISO (International Standard Organisation) were presented. Yael Schindler (Federal Office for the Environment, Berne, Switzerland) described ongoing work on the application of bioassays for assessing surface water quality within the framework of the Swiss Modular Stepwise Procedure (http://www.modul-stufen-konzept.ch). She described the requirements (e.g., applicability, sensitivity and robustness as well as data interpretability) such bioassays must meet to be of use to regulatory authorities and concluded that national and international standardization of bioassays increases the chances of incorporating these tools into regulatory frameworks. With recent examples of efforts for the international standardization of bioassays for measuring genotoxicity and hormonal effects, Sebastian Buchinger (German Federal Institute of Hydrology, Koblenz, Germany) and Etienne Vermeirssen (Ecotox Centre Eawag-EPFL, Dübendorf, Switzerland) confirmed that standardization is a prerequisite for the transfer of state-of-the-art scientific knowledge to regulation. They underlined the importance of providing scientific knowledge for this process.

### Session 3: Ecotoxicology of sediments (chairs: H. Hollert, S. Schiwy, D. Becker)

Sediments are an important topic for many scientists and regulatory authorities due to their close linkage to the aquatic food web and their potential for accumulating pollutants. The session opened with an overview of the project, Dioxin Risk Assessment for sediment Management Approaches (DioRAMA), presented by Markus Brinkmann (RWTH Aachen University, Germany). He pointed out the urgent need for a better understanding of the cause–effect relationship of dioxin-like compounds (DLCs), such as polychlorinated biphenyls, polychlorinated dibenzo-p-dioxins and furans, as well as polycyclic aromatic hydrocarbons. DioRAMA aims at the validation of cell culture-based bioassays for the quantification of DCLs in sediment and biota and their implementation in sediment management guidelines. It involves experimental investigations and modeling approaches to examine the uptake and effects of DLCs in fish. Marvin Brinke (Federal Institute of Hydrology, Koblenz, Germany) presented a study focused on developing Sediment Quality Guidelines (SQG) for quality assessment of fine freshwater sediments including an evaluation of their predictive ability. Two nematode-based tools were compared: (1) nematode-based SQG for estimating the toxic effects based on measured chemical concentrations and (2) the NemaSPEAR index. Results indicate that nematode-based SQG can be used as a screening tool in a weight-of-evidence framework. Sabrina Schiwy (RWTH Aachen University, Aachen, Germany) described the development of a new EROD assay with *Danio rerio* embryos (FE-EROD assay), a sediment contact assay, which takes into account the bioavailable potential of native sediment samples. This assay is ecologically relevant and provides a realistic exposure scenario simulating in situ exposure conditions. A number of additional biological end points (embryotoxicity, teratogenicity, dioxin-like activity and gene expression analyses) were quantified and chemical analyses performed. The results demonstrated that mechanism-specific bioassays such as FE-EROD provide important information for the evaluation of sediment quality.

### Sessions 4–7: Aquatic toxicology

A broad range of topics were covered in four sessions on aquatic toxicology. They followed a common thread: to build a bridge between research and practical application of ecotoxicological testing methods and facilitate the application of these methods in regulatory monitoring and risk assessment.

### Session 4: Aquatic toxicology I—methods (session chairs: H. Segner, M. Brinkmann)

This session focused on new methodologies for the assessment of toxic effects in aquatic organisms and covered a range of tests from single-species to multi-species assays. Svenja Boehler (University of Heidelberg, Germany) showed that toxicity data obtained with the fathead minnow embryo test are comparable to those of the zebrafish embryo test. Carolina Di Paolo (RWTH Aachen, Germany) presented the results of an inter-laboratory study on the potential of behavioral parameters as end points in toxicity tests with zebrafish larvae. Both neuroactive and neurotoxic compounds induced altered locomotory patterns of larvae. Results showed reasonable intra- and inter-laboratory reproducibility. Sebastian Beggel (Technical University Munich, Freising, Germany) tested the filtration behavior of the freshwater mussel, *Anodonta anatina*, as an indicator of water quality by means of modern real-time sensor technology. To date, this biomonitor has been evaluated for its response to increased salt concentrations; future studies will evaluate its utility for toxic chemicals. Finally, Matthias Dünne (OHB System AG, Germany) presented a commercially available, closed multi-species test system, a versatile tool to assess the effects of chemicals and bioaccumulation for a community of aquatic species including bacteria, plants, invertebrates and fish (medaka, *Oryzias latipes*).

### Session 5: Aquatic toxicology II—transcriptomics (session chairs: H. Segner, I. Werner)

Transcriptomics is a promising tool for future monitoring of the toxic effects of micropollutants, both as sublethal biomarkers and as diagnostic tools. Stephan Fischer (Eawag, Dübendorf, Switzerland) presented the results of a field study with brown trout (*Salmo trutta*). He provided convincing data on how the integration of transcriptomic methodologies can enhance the assessment of chemical exposure and effects in field studies. Alba Quesada Garcia (INIA, Madrid, Spain) showed how transcriptome arrays can help to assess the response of complex physiological systems of fish—in this case the immune system—to toxic chemicals. Rebecca Beauvais-Flueck (University of Geneva, Switzerland) applied transcriptomics (RNA sequencing) to unravel the mechanisms of mercury toxicity in aquatic phytoplankton and macrophytes. The results show clear differences in the response to mercury between the two groups of primary producers, with the most sensitive target systems being photosynthesis in phytoplankton, and the intermediary metabolism in macrophytes. Sven Koglin (RWTH Aachen, Germany) provided information on the use of microarrays and RNA sequencing to investigate how the hepatic transcriptional profile of a non-model fish species, roach (*Rutilus rutilus*), responds to a variety of dioxin-like compounds.

### Session 6: Aquatic toxicology III—challenges (session chairs: H. Segner, C. Kienle)

This session focused on some of the challenges in aquatic toxicity assessment. Carolina Di Paolo (RWTH Aachen, Germany) provided an overview of the NORMAN inter-laboratory study on biotesting of spiked water extracts, highlighting both the challenges of performing such studies and the need for more method harmonization in environmental testing. Sebastian Beggel (Technical University Munich, Freising, Germany) presented a study on the aquatic risk of antiscalants, which are present in wastewater from water desalination processes. The results indicate that the ecotoxicological relevance of these substances is higher than previously assumed. Finally, Lucas Jagodzinski (University College Cork, UK) discussed the challenges of measuring the toxicity of energy ashes from virgin wood fuels. In aquatic plants, these ashes can have both growth-promoting and toxic effects. The nature of the effect largely depends on conditions in the receiving environment.

### Session 7: Aquatic toxicology IV—mechanisms (session chairs: I. Werner, E. Simon)

Multidisciplinary approaches offer solutions to complex research questions. This session presented interesting case studies and new test systems. Smitha Pillai (Eawag, Dübendorf, Switzerland) introduced an integrated systematic methodology for better understanding the toxic mechanisms of manufactured nanomaterials (MNM) in aquatic organisms and highlighted its potential for comprehensive risk assessment. Silver and zinc metal ions dissociated from nanomaterials affected multiple end points, such as growth, oxidative stress and damage, energy metabolism and photosynthesis in the freshwater algae (*Chlamydomonas reinhardtii*). Tulasi Kirla (Eawag, Dübendorf, Switzerland) presented the results of a study on the effects of cocaine on the behavior and toxicokinetics of larval zebrafish. Internal bioaccumulation and distribution patterns of cocaine were assessed by matrix-assisted laser desorption ionization mass spectrometry imaging (MALDI-MSI). Interestingly, this drug accumulated in the eyes and remained there for a prolonged period after depuration. Additionally, it elicited hypoactivity in zebrafish larvae. Denise Brettschneider (Goethe Institute, Frankfurt, Germany) reported significant changes in reproduction and energy metabolism of snails (*Potamopyrgus antipodarum*) after dietary exposure to triclocarban (TCC, an antimicrobial personal care product) or cadmium (Cd). TCC also caused obesogenic effects. Finally, Kristina Rehberger (University Bern, Switzerland) pressented her results on an in vitro bioassay for measuring the immunotoxic potential of xenobiotics. She used rainbow trout (*Oncorhynchus mykiss*) leukocytes and quantified immune responses including cytokine gene expression, and phagocytic and oxidative burst activities.

Posters in this session provided information on chemical-specific effects as well as new methodological approaches. The toxic effects of nanomaterial-bound antifungal agents, TCC, pharmaceuticals (e.g., metoprolol), metals (methylmercury and lead chromate), and pesticides (i.e., metamitron) were investigated using a variety of aquatic bioassays. A physiologically based pharmacokinetic model was applied to predict the internal exposure concentrations in rainbow trout (*Oncorhynchus mykiss*) organs based on in vivo exposure concentrations. Fish acute toxicity was predicted using the rainbow trout gill cell line (RTgill-W1) as an animal-free alternative to the fish acute toxicity testing. It was demonstrated that results of an in vitro metabolism assay developed for rainbow trout were transferrable to other species, such as common carp, and the presence of a defense system in early life stages of rainbow trout was confirmed. Effect-directed analysis (EDA) was shown to be a promising tool for water quality assessment, combining the advantages of bioassays with chemical analytics to identify compounds causing toxicity. EDA was used to identify the source of unexplained toxic and teratogenic activities found in surface water receiving untreated wastewater discharges in Germany. Complementary to the more established in vitro bioassays, a small-scale genotoxicity assay (micronucleus tests) with a zebrafish liver cell line and larvae was successfully incorporated into EDA studies. Finally, the relevance of pulsed dosing tests in higher-tier risk assessment of plant production products (PPP) was highlighted.

### Session 8: Terrestrial ecotoxicology (chairs: A. Aldrich, F. Fischer)

Presentations in this session focused exclusively on bees and amphibians. It was evident that there is a need for additional toxicity testing methods for these groups. Annette Aldrich (Agroscope, Wädenswil, Switzerland) reported that amphibians are experiencing increasing pressure from diverse stressors, in particular loss of habitat, and plant protection products (PPP). The data requirement for authorization of PPP in the European Union (EU 283/2013) determines that amphibians must be considered to be in prospective environmental risk assessment. However, amphibians do not need to be tested for PPP authorization; therefore, there is a lack of information regarding PPP toxicity to this group. Dermal uptake is considered to be a significant route of exposure, but so far there is no adequate testing system. Katharina Kaufmann (BASF SE, Limburgerhof, Germany) presented the results of a skin absorption in vitro test (OECD TG 428), which was adapted to amphibian skin (*Xenopus laevis*). Dermal absorption and uptake were quantified using two reference substances, caffeine and testosterone. To address dermal toxicity, an amphibian skin corrosion test based on OECD TG 431 was successfully used. Her studies showed that amphibian skin was significantly more permeable for chemicals than human skin.

Loss of habitat, habitat quality and exposure to PPP are also important stressors for bee populations, and more studies on the effects of environmental chemicals on bees are urgently needed. It is important, however, to understand testing procedures to reliably interpret results. For example, wetting agents used in PPP contact studies with *Bombus terrestris* and *Osmia bicornis* to facilitate PPP permeation can influence PPP toxicity (Eva Eschenbach, University of Koblenz-Landau, Germany). Promising new methodologies were presented to measure (1) the influence of PPP on the pollination efficiency of *Bombus terrestris* (poster) and (2) the effects of PPP exposure via wings (wing contact test) in *Osmia bicornis* (poster).

### Session 9: Soil ecotoxicology (chairs: R.-A. Düring, S. Campiche)

Soils are increasingly being recognized as important and valuable ecosystems. In this session, Daniel Wächter (Agroscope, Wädenswil, Switzerland) presented an overview of the Swiss National Soil Monitoring Network (NABO) which was initiated in the mid-1980s to monitor long-term changes in soil quality. Chemical analysis initially focused on heavy metals and acidification, but with improving analytical methods soil monitoring networks are able to integrate a larger array of organic contaminants such as pesticides. A well-defined conceptual approach is now needed to prioritize compounds to be monitored. It will be based on results from multi-residue analysis of 40 target pesticides in soil samples and a risk assessment to rank their relevance. Aurea Chiaia-Hernandez (Eawag, Dübendorf, Switzerland) presented a strategy for prioritizing organic soil contaminants for monitoring. Using an environmental partitioning model, she identified chemicals that preferentially partition to soil.

Sophie Campiche (Ecotox Centre Eawag-EPFL, Lausanne, Switzerland) showed study results on the toxicity of commercial wood preservatives in soil using the collembolan reproduction and earthworm avoidance tests The two tests showed similar sensitivity to iodocarb (IPBC), but earthworms were more sensitive to propiconazole and wood preservatives containing mixtures of active ingredients. Nicole Spann (Bielefeld University, Germany) presented a study on the effects of copper on the metabolome of two nematode species (*Caenorhabditis elegans* and *Panagrellus redivivus*). Nematodes were exposed in liquid test medium to two concentrations of copper (reproduction-EC_50_ and ½ EC_50_). Effects on the metabolome were predominantly related to carbohydrate, energy and amino acid metabolism.

### Session 10: Passive sampling and passive dosing (chairs: E. L. M. Vermeirssen, B. Becker)

Passive sampling of chemicals in diverse matrices such as air, water, sediment, soil or biota is extensively used in basic and applied research. Over the past 20 years, the technique has matured and passive samplers are now increasingly accepted in a regulatory context as tools that facilitate environmental monitoring. Markus Zennegg (Empa, Dübendorf, Switzerland) showed that silicone sheets used as a passive samplers to locate sources of PCB pollution allowed for an accurate pinpointing of PCB sources, as well as for monitoring the effectiveness of measures taken to eliminate PCB contamination of rivers. Neither biota nor sediment monitoring was sensitive enough to locate the unknown PCB sources. Benjamin Becker (BfG, Koblenz, Germany) and colleagues applied silicone sheets to assess the spatial and temporal concentrations of hydrophobic contaminants across five German rivers. Accurate information on in situ sampling rates is needed to derive accurate time-weighted average concentrations from the mass of a compound sorbed to a kinetic passive sampler. Becker and colleagues used performance reference compounds to correct for these environmental factors such as flow rate or biofouling. The highest concentrations of PCBs and DDT metabolites were found in the Elbe. PAHs were most elevated in the Saar and shown to fluctuate appreciably over various passive sampling campaigns. To establish in situ sampling rates for 88 compounds, a combination of passive sampling and broad LC–MS/MS compound screening was applied in Swiss rivers (poster). Ahmed Tlilli (Eawag, Dübendorf, Switzerland) presented the study results on using Empore disks as a “biomimetic” tool to sample a mixture of chemicals from four Swiss wastewater treatment plant (WWTP) effluents. He tested the passive sampler extracts on periphyton originating from river sections up- and downstream from the effluent discharges. Using this pollution-induced community tolerance (PICT) approach he showed that periphyton from the downstream river stretches were adapted to the chemical mixture emitted as WWTP effluent. End points such as bacterial and algal production or photosynthetic yield all showed similar responses. Results underline the sensitivity of the method (maximal observed difference down-/upstream reached almost 100-fold) and indicate its potential for monitoring contaminant point sources. In a different type of application, Fabian Fischer and colleagues (Justus-Liebig University, Giessen, Germany) used silicone rings to passively dose PAHs in the standardized aquatic nematode reproduction test. The study highlights three advantages of using passive dosing—as opposed to standard compound spiking: (1) freely dissolved concentrations of PAHs remain constant over a 5-day exposure period; (2) freely dissolved concentrations of PAHs remain constant irrespective of the food dose used in the assay; (3) passive dosing enables the use of non-depletive equilibrium passive sampling to measure freely dissolved concentrations at the end of toxicity tests.

### Session 11: Environmental chemistry (chairs: A. Schäffer, M. Zennegg)

This session presented new insights into the fate and environmental concentrations of a variety of pollutants. Andreas Schäffer (RWTH Aachen University, Germany) discussed the long-term persistence of chemicals in soil by forming non-extractable residues (NER). NER are formed by all chemicals that enter the soil and sediments to various extents. Entrapment of xenobiotic NER in the solid matrix (type I) must be considered as stabilization of a chemical in soil from which parts may be released long term, whereas xenobiotic NER covalently bound to humic matter (type II) and NER bound to biogenic residues (type III), the latter formed by readily biodegradable chemicals, are of no environmental concern. Lena Schinkel (Helmholtz Centre for Environmental Research, Leipzig, Germany) presented information on the distribution patterns of synthetic pyrethroids in sediments of European river estuaries and deltas. Synthetic pyrethroids are insecticides which are increasingly used worldwide. When they enter water bodies, they tend to sequester to sediments and can cause toxicity to invertebrates in the ng/g (dry weight) range. Screening results showed elevated concentrations in several German rivers. Calculated sum of toxic units (TU) showed high toxic potential for these river sediments, mainly due to cypermethrin. Rolf-Alexander Düring, Gießen University, presented a study on the potential of technogenic and biogenic palladium nanoparticles of catalyzing hydrodehalogenation of persistent organic pollutants (POPs). A goal was to depict possible transformation pathways of hexachlorobenzene and one PCB congener using complementary analytical approaches (SPME–GC–MS and PTR-TOF–MS). Finally, Markus Zennegg (Empa, Dübendorf, Switzerland) presented the case of a Swiss farm whose cattle were highly contaminated with PCBs. Dioxin-like PCB (dl-PCB) concentrations in veal exceeded the permitted maximum levels (ML) of 4 pg WHO-TEQ_05_/g lipid weight (lw) set by the European Union by more than a factor of 4, and the sum of the six marker PCB exceeded the permitted ML of 40 ng/g lw by more than a factor of 12. Investigations revealed that wall paint applied in the stable more than 40 years ago contained 3–16 % PCBs with the same congener pattern. The stable was subsequently decontaminated. This case shows that persistent organic pollutants like PCBs, banned for decades, can still contaminate animals, humans and the environment.

### Session 12: Monitoring of surface waters (chairs: B. Escher, K. Peschke, R. Triebskorn)

This session had a strong focus on treated wastewater and its influence on surface water quality, as well as monitoring tools for water quality assessment. The session was opened by Ueli Ochsenbein’s (Water and Soil Protection Laboratory, Department of Organic Analytical Chemistry, Berne, Switzerland) presentation on an extensive sampling campaign conducted in Bernese rivers. The concentrations of pesticide transformation products exceeded those of the respective parent compounds by 75–98 %, indicating that target analysis of parent compounds alone does not suffice for the assessment of water quality, but transformation products should be considered. They should also be assessed for their toxicity, as demonstrated by the study presented by Katharina Heye (Goethe University, Frankfurt, Germany). A transformation product of carbamazepine was shown to be more toxic than its parent and thus added to the environmental risk of the mixture despite being present at relatively low concentrations. Marion Junghans (Ecotox Centre Eawag-EPFL, Dübendorf, Switzerland) presented an evaluation concept for mixtures of micropollutants from non-point sources. She concluded that grab samples are not representative of pollution levels for the highly variable exposure dynamics such sources create.

Wastewater treatment plant effluents are important sources of micropollutants. The presentation by Cornelia Kienle (Ecotox Centre Eawag-EPFL, Dübendorf, Switzerland) introduced the EcoImpact project focused on monitoring receiving streams using a combination of chemical analysis, in vitro and in vivo bioassays and ecological indicators. Moving from Switzerland to a larger European stream, the Danube, Maria König (UFZ—Helmholtz Centre for Environmental Research, Leipzig, Germany) presented results on the impact of untreated wastewater on the Danube River in Novi Sad, Serbia. Despite the large flows, untreated wastewater constitutes up to 30 % of the river’s flow. Accordingly, the effects shown by a number of in vitro end points including hormonal effects and the adaptive stress response came as no surprise.

Multiple chemical as well as non-chemical stressors can adversely impact wildlife. The study presented by Elisabeth Berger (Senckenberg Research Institute and Natural History Museum, Frankfurt, Germany) demonstrated the important role played by pesticides, industrial chemicals and wastewater-associated micropollutants in reducing the abundance of sensitive aquatic invertebrates. Establishing a direct correlation between micropollutant concentrations and trait-based indices for water quality is a challenge, however. In this regard, Mira Kattwinkel (Eawag, Dübendorf, Switzerland) demonstrated the utility of correlation analyses for ecological assessment of communities with low biodiversity.

### Session 13: Multiple stressors (chairs: I. Werner, M. Korkaric)

Understanding the impact of multiple environmental and chemical stressors on ecosystems is one of the greatest challenges in ecotoxicology. Matthias Liess (UFZ, Leipzig, Germany) presented lessons learned from several studies. Extrapolation from the laboratory to the field situation and from acute to chronic toxicity is challenged by a lack of system context in short-term laboratory toxicity tests. Important factors are (1) recovery and thus the long-term manifestation of toxic effects on populations and (2) the effect culmination following re-occurring stressor exposure. Both are a function of inter- and intraspecies competition and must be taken into account for model predictions of multiple stressor effects in the environment—Multiple stressor effects often play a role in population declines. Helmut Segner (University of Berne, Switzerland) presented information on the decline of brown trout populations in Swiss rivers over the last few decades. The decline is most pronounced in Swiss midland rivers, which are characterized by a high prevalence of trout infected with *Tetracapsuloides bryosalmonae*, which causes proliferative kidney disease (PKD). These rivers also experienced the greatest increase in water temperature and contain relatively high concentrations of micropollutants. Studies showed that PKD-associated mortality of trout is positively correlated with temperature, possibly due to downregulation of immune pathways. Moreover, data from a pilot study, in which brown trout were exposed to 30 % wastewater, indicate that PKD-associated mortality is enhanced by chemical pollution. Complex interactions of environmental factors (nutrients, warming, parasites) also affected the food web of freshwater lakes. Piet Spaak (Eawag, Dübendorf, Switzerland) and colleagues examined the diverse ways in which cyanobacteria affect other species in lake communities. They showed that harmful cyanobacteria blooms were caused by a combination of lake warming and re-oligotrophication (N:P ratio). The greater abundance of toxin-producing cyanobacteria reduced food quality for *Daphnia* sp, which, in turn, increased *Daphnia* susceptibility to the parasite *Caullerya mesnili*. Finally, Lars Straub (University of Berne, Switzerland) presented a study on honey bees exposed to both the ectoparasitic mite, *Varroa destructor,* and the neonicotinoid insecticides, thiamethoxam and clothianidin, via pollen in late spring (6 weeks). Results showed the seasonality of effects: significant delayed effects of combined stressors on bee longevity manifested only in autumn. Similarly, bees exposed to combined stressors showed the greatest losses in general body weight in autumn. As a consequence, honey bee colonies exposed to both *V. destructor* and insecticides may encounter increased winter losses. The study highlighted the importance of considering time-lag effects when examining multiple stressors.

### Session 14: Risk assessment of pesticides (chairs: N. Chèvre, A. Aldrich, M. Junghans)

This session was primarily focused on prospective risk assessment under the PPP authorization legislation. Katja Knauer (Federal Office for Agriculture, Berne, Switzerland) and Annette Aldrich (Agroscope, Wädenswil, Switzerland) showed how monitoring data may be used to improve PPP risk assessment and management in Switzerland. They proposed that authorization of PPP should be reviewed, if measured environmental concentrations (MEC) exceed the regulatory acceptable concentrations (RACs). To analyze whether maximal predicted environmental concentrations (PEC_max_) are indeed “worst case”, Beate Fulda (Agroscope, Wädenswil, Switzerland) compared PEC_max_ in surface waters calculated using the EXPOSIT 3.01 model, with MEC obtained from Swiss monitoring programs. In over 99 % of samples, MEC were lower than PEC_max_. The authors conclude that the calculated PEC_max_ are sufficiently conservative, but caution that monitoring programs using monthly grab or 7- to 14-day composite samples may miss peak concentrations in the environment. A related study (poster) analyzed the effect of PPP application patterns on PEC calculations. For PPP with long degradation half-lives (DT_50_), PEC_max_ depends on the time between applications, while for PPP with low DT_50_, the frequency of applications is more important. In an example of actual application practices in a vineyard (poster), the median of the time between PPP applications and average amounts applied were equal to predictive model assumptions. Saskia Knillmann (UFZ, Leipzig, Germany) presented evidence that PPP use patterns for a given geographical area are influenced by climatic conditions. Based on an analysis of insecticide use and climate data from several European countries, she concluded that the average spring air temperature is a useful predictor of the amount of insecticide applied: the warmer the spring, the more is the insecticide applied and the earlier is the first application.

The link between effects measured in the laboratory and in the field was the topic of Ralf Schäfer (University of Koblenz-Landau, Germany). He criticized that field studies and theory-driven research were severely underrepresented at SETAC conferences. Moreover, he challenged model-based extrapolation as a tool to close the postulated laboratory–field divide. Moving the focus from an assessment of individual chemicals to the receiving ecosystems by (1) testing the predictive power of ecological theories for pesticide effects in the field or (2) better, linking the existing chemical and biological monitoring programs could overcome this problem. In a similar plea, Martina Ross-Nickoll (RWTH, Aachen, Germany) stressed the need for integrative landscape assessment. Effects of pesticides and other chemicals on biological diversity should be evaluated at the landscape level. For this purpose, a tool based on landscape ecology and remote sensing was used to simulate the effects of different risk mitigation measures on biodiversity.

Marcel Mathis (Agroscope, Wädenswil, Switzerland) used a comprehensive assessment approach, combining environmental risk assessment with economics, to compare the sustainability of using neonicotinoid (current use restricted or banned) and pyrethroid insecticides, for combatting *Psylliodes chrysocephalus* in rapeseed production. The authors concluded that—under the assumptions made for this study—the use of pyrethroids in spray applications is more sustainable than the use of neonicotinoid-coated seeds. This is mainly due to the observation that infestations do not occur every year. Lea Perseke (Knoell Consulting GmbH, Germany) reviewed information on the effects of the fungicide, prochloraz, in fish. While the data showed evidence for endocrine effects and growth inhibition, available exposure data did not indicate a risk. In an effort to address the risk of PPP mixtures and identify the trophic level most at risk, Nathalie Vallotton (Dow Europe GmbH, Horgen, Switzerland) proposed a new approach to aquatic mixture risk assessment. The methodology separately assesses risk for each trophic level: fish, invertebrates, non-vascular plants and vascular plants. Unlike the EU, the USA has already embraced the concept of trophic level-based toxicity benchmarks.

### Session 15: Endocrine disruption (chairs: S. Schwarz, J. Oehlmann)

A broad overview was provided on current topics in research on endocrine disrupting chemicals in the environment, ranging from specific analytics to visible effects on the whole organism. It gave insights into recent advances in linking analytical and ecotoxicological techniques, the characterization of complex mixtures and the development of internationally standardized protocols for the testing of hormonally active chemicals. Thus more and more useful tools are becoming available for investigating the endocrine effects of complex mixtures and field samples.

Andreas Schönborn (Zürcher Hochschule für angewandte Wissenschaften, Wädenswil, Switzerland) introduced the planar-YES (yeast estrogen screen), a method coupling thin-layer chromatography with effect-driven analysis, which was used to measure estrogenicity in the OxiScreen project. The influence of several oxidation techniques on the endocrine potential of these water samples was assessed. Overall, it was shown that the planar-YES can be used for the investigation of surface water samples with comparable sensitivity to other reporter gene assays. At the same time, the assay linked with non-target screening allows identification of unknown endocrine-active chemicals and transformation products in complex environmental samples. A study presented by Nina Schlotz (Universitätsklinikum, Freiburg i. Breisgau, Germany) addressed the estrogenicity of chemical mixtures (industrial chemicals, pesticides, and phytochemicals) using the human cell-based ER-CALUX^®^ reporter gene assay. Cornelia Geiß (Goethe University, Frankfurt am Main, Germany) presented the projects ValMolRepro I and II, involving the development of a standard OECD technical guideline with the freshwater mudsnail, *Potamopyrgus antipodarum,* for testing of reproductive toxicants, including endocrine disrupters. An international inter-laboratory validation study was conducted to confirm the comparability of the results. The results emphasize the robustness and reproducibility of the method. Furthermore, *P. antipodarum* proved to be highly sensitive to a variety of chemicals with endocrine potential.

Several posters addressed a variety of topics, in particular, the input of estrogenic compounds from non-point sources to Swiss surface waters, bisphenol A-free baby bottles as a potential source for endocrine disruptors, and the development of screening tools for the detection and assessment of anti-androgenic substances for application in EDA. The T47D-Kbluc-assay was confirmed as a promising tool for the assessment of WWTP effluents for endocrine potential. Following a study on the effects of different storage conditions on endocrine activity of water samples, it was recommended that wastewater samples should be tested immediately after sampling or stored at 4 °C until testing.

### Session 16: Microplastics (chairs: J. Muncke, K. Heye)

Microplastics in the environment are receiving increasing attention. This session provided insight to this emerging field and highlighted future research questions. Overall, the session gave an excellent overview on current challenges for advancing the understanding of the effects of microplastics in the environment. Foremost is the development of standardized analytical methods which can serve as basis for policy action. Further, effect-directed assays to measure the ecotoxicological potential of plastic leachate are desirable, as well as a more robust understanding of microplastics interaction with different trophic levels (including the human food chain), either mechanically, as source or vector for hydrophobic micropollutants.

Sylvia Frey (Ocean Care, Wädenswil, Switzerland) gave an overview of the current knowledge on the occurrence and biological impacts of microplastics in freshwater and saltwater bodies. Microplastics can function as vectors of chemicals by accumulating persistent organic pollutants (e.g., PCBs) and transferring them to living organisms. There is an urgent need to better understand such interactions and to curb plastic inputs into the aquatic environment from: (1) single-use plastics, (2) microbeads in cosmetics, and (3) plastic fibers which are not completely removed in WWTPs. A major barrier to advancing research and implementing policy action on the issue of microplastics is the lack of standard analytical methods to quantify microplastics. With currently used non-standard methods, the risk of false positives can be considerable. Florian Storck (Water Technology Center, Karlsruhe, Germany) presented his research on analytics and occurrence of microplastics in inland waterways. He recommends not to rely on visual sample inspection only, but to include chemical analysis like Raman microscopy. Nicole Bandow (Bundesanstalt für Materialforschung und Prüfung, Berlin, Germany) measured chemicals released from three different types of microplastics: polystyrene, high density polyethylene, and polyvinyl chloride. The plastic materials were either directly eluted, or first exposed to ultraviolet (UV) light or thermic stress and then eluted. Many different peaks were detected in eluates, even more after plastics were exposed to UV or heat. Their identification is challenging, and an ecotoxicological evaluation based on analytical results is difficult due to the lack of toxicological data. She concluded that effect-based assays are needed to assess the toxicity of the eluates. Concentrations of microplastics in Swiss lakes and rivers were measured by Florian Faure (Swiss Federal Institute of Technology, Lausanne, Switzerland). All water bodies investigated contained microplastics. Concentrations increased by 5–150 times after stormwater overflow events. Faure and colleagues also analyzed selected additives and monomers in the plastic particles, as well as hydrophobic micropollutants. All chemicals of interest were detected, including bisphenol A, nonylphenol, and several phthalates.

Two studies presented as posters showed that WWTPs retain most particles (over 92 %) in the sludge; however, microplastics were found in effluents from all WWTPs sampled. The majority of microplastics in effluents consist of polyethylene. Particles were small with highest frequencies in the size range of 100–200 µm.

### Session 17: Wastewater treatment and toxicity (chairs: T. Wintgens, D. Rensch)

Wastewater treatment is a major water quality control measure to protect aquatic ecosystems and drinking water resources. This session aimed at presenting advances in wastewater treatment systems with respect to removal of organic micropollutants and reducing potential ecotoxicological effects in receiving water bodies. This topic has particular relevance in Switzerland where a new water protection ordinance requires WWTP to significantly reduce micropollutant emissions via technological upgrading of the 100 most relevant WWTP (of 700).

Pascal Wunderlin (VSA, Switzerland) described a testing framework to investigate the suitability of ozonation for the removal of micropollutants from wastewater. The tests determine the most effective ozone treatment for a water sample and measure the formation of potentially hazardous oxidation by-products such as bromate and *N*-nitrosodimethylamine (NDMA). These tests are to be complemented by a suite of biological effect assays. Rebekka Teichler (Eawag, Switzerland) presented the results of a study performed at the first full-scale ozonation plant in Switzerland, WWTP Neugut. It was focused on the formation of transformation products (TP) and their reduction by different types of post-treatment systems [conventional filters, biofilm system, and (biologically) activated carbon]. While concentrations of some TP were little reduced, NDMA underwent significant biological degradation. Cornelia Kienle (Ecotox Centre Eawag-EPFL, Switzerland) used ecotoxicological bioassays to measure the efficiency of post-treatment systems at WWTP Neugut. She showed that all combinations of ozonation plus post-treatment systems significantly reduced the biological effects of effluents. Katharina Peschke (University of Tübingen, Germany) presented the results of a study in Baden-Württemberg on advanced wastewater treatment with powdered activated carbon (PAC) and its contribution to ecosystem health in receiving waters. She found positive impacts of advanced wastewater treatment on fish. Similarly, Sabrina Giebner (Goethe University Frankfurt/Main, Germany) studied the effectiveness of treatment combinations, including ozonation and PAC, to reduce a wide range of ecotoxicological effects. In her study, advanced technologies decreased estrogenic activity, but not anti-estrogenic activity of the WWTP effluent. Sand filtration reduced mutagenicity (which was increased after ozonation) confirming the importance of a biological filter system as ozonation post-treatment. Jan Svojitka (University of Applied Sciences and Arts Northwestern Switzerland, Muttenz) investigated the process combination, PAC, and membrane filtration, for effluent treatment with respect to the removal of micropollutants, biological effects as well as membrane performance. The results confirm an efficient removal of micropollutants for this compact process. Finally, Dennis Becker (Goethe University Frankfurt/Main, Germany) reported results from the European ENDETECH project on enzymatic degradation of antibiotics in water and wastewater samples using a fungal laccase. While antibiotics were successfully removed by the process, antibiotic activity could not be reduced significantly.

### Awards for young scientists

#### Conference presentation awards

Kirla Krishna Tulasi (Eawag, Dübendorf, Switzerland) received the award for best conference platform presentation with her talk “Toxicokinetic and internal distribution of cocaine in zebrafish larvae: unexpected accumulation and retention in the eyes”. In second place was Katharina Heye (Goethe Universität, Frankfurt, Germany) “Comparative toxicity of carbamazepin and its main on *Chironomus riparius”,* and Fabian Fischer (Justus Liebig Universität, Giessen, Germany) was awarded the third place for his presentation “Passive dosing of hydrophobic organic chemicals in toxicity tests with *C. elegans”.*

The best poster award went to Sina Ostermann (Goethe Universität, Frankfurt, Germany) “Is there an influence of storage conditions on endocrine activity of wastewater samples?”. Two posters shared the second place: Christoph Parsch (Goethe Universität, Frankfurt, Germany) “Are wastewater treatment plants relevant sources of microplastic in aquatic ecosystems?”, and Theophil Kroller (Fachhochschule Technikum, Vienna, Austria) “Validation and application of a cell culture assay for screening for endocrine-active compounds in wastewater”.

#### SETAC GLB awards

Alena Thierbach (Dept. Environmental Toxicology at Eawag, Switzerland) won the award for best master’s thesis 2014 for her study—Mechanistic and systematic understanding of the toxicity of manufactured nanomaterials in the algae “*Chlamydomonas reinhardtii*”. The award for the best doctoral thesis went to Christoph Moschet (Dept. Environmental Chemistry, Eawag, Switzerland) for his thesis on “Addressing blind spots in the assessment of pesticides in surface waters: a complete screening using trace-level mass spectrometry techniques and complementary sampling strategies”.

## Additional file

10.1186/s12302-016-0088-3 The 20th Annual Meeting of SETAC GLB took place September 7–10, 2015 in Zurich, Switzerland. The complete program and abstracts are available as Additional file 1.

