# Supplementary material for: The 2015 Annual Meeting of SETAC German Language Branch in Zurich (7–10 September, 2015): Ecotoxicology and environmental chemistry—from research to application
Source: Environ Sci Eur. 2016 Jul 6;28(1):20. doi: 10.1186/s12302-016-0088-3 (PMC5044970; doi:10.1186/s12302-016-0088-3)
Supplement: Supplementary file 1 — 10.1186/s12302-016-0088-3 The 20th Annual Meeting of SETAC GLB took place September 7–10, 2015 in Zurich, Switzerland. The complete program and abstracts are available as Additional file 1. [file 12302_2016_88_MOESM1_ESM.pdf]

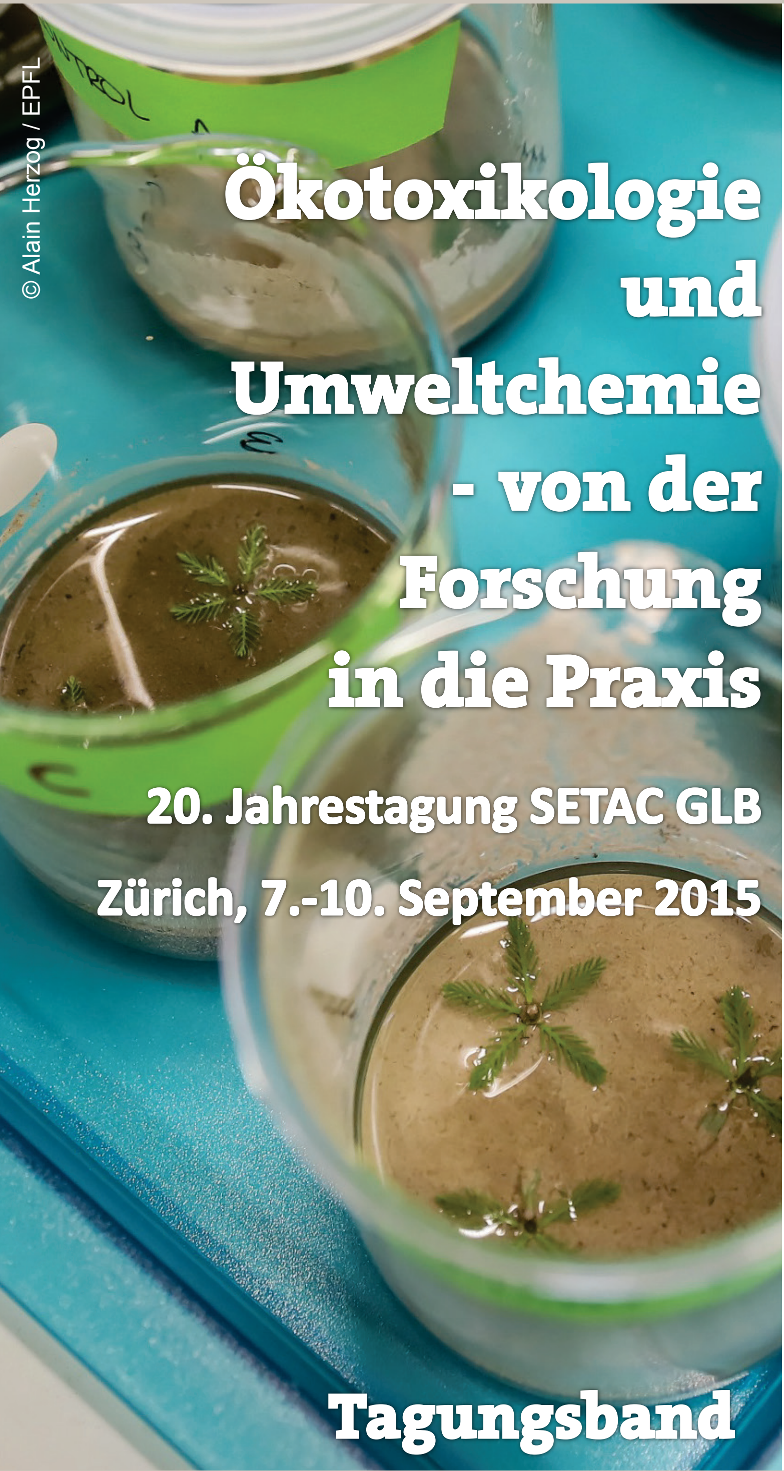

© Alain Herzog / EPFL

Ökotoxikologie und Umweltchemie - von der Forschung in die Praxis

SETAC
German Language Branch

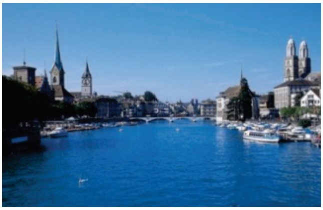

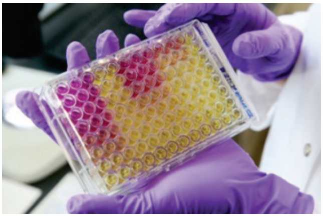

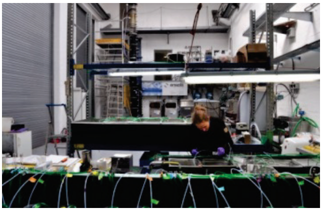

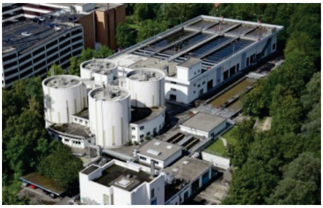

20. Jahrestagung SETAC GLB

Zürich, 7.-10. September 2015

Tagungsband

oekotoxzentrum
centre ecotox

Inhaltsverzeichnis

Wissenschaftliches Komitee **5**

Organisationskomitee **5**

Grusswort des SETAC-GLB Präsidenten **6**

Grusswort des Organisationsteams **7**

Grusswort des Gesundheits- und Umweltdepartements der Stadt Zürich **8**

Die Gastgeber **9**

Der Tagungsort **9**

Allgemeine Informationen **10**

- Anschrift 10
- Anreise zur ETH Zürich 10
- Tagungsbüro 13
- Mittagessen 13
- WLAN 13

Das Rahmenprogramm **13**

- Get Together, Montag, 7. September 2014, 18:30 Uhr 13
- Gesellschaftsabend, Dienstag, 8. September, 20:00 Uhr 13
- Tagungsparty, Mittwoch, 9. September, 20.30 Uhr 13
- Exkursionen 14
  - Führung ARA Neugut und Forum Chriesbach 15
  - Führung Erdbebensimulator 15
  - Stadtführung Zürich 16

Übersicht Tagungsprogramm **17**

- Montag, 07.09.2015 17
- Dienstag, 08.09.2015 17
- Mittwoch, 09.09.2015 18
- Donnerstag, 10.09.2015 19

Inhaltsverzeichnis

Sessions **20**

Dienstag, 08.09.2015 20

Mittwoch, 09.09.2015 22

Donnerstag, 10.09.2015 25

Plenarvorträge 34

Vorträge **39**

Ökotoxikologie von Bauchemikalien 40

Biotest-Normungsverfahren 44

Sedimentökotoxikologie 48

Aquatische Ökotoxikologie 51

Terrestrische Ökotoxikologie 62

Bodenökotoxikologie 65

Passive Sammler 69

Umweltchemie 74

Monitoring von Oberflächengewässern 79

Risikobewertung Pestizide 86

Multiple Stressoren 94

Aquatische Ökotoxikologie 98

Endokrine Disruption 102

Mikroplastik in der aquatischen Umwelt 106

Abwasserbehandlung und -toxizität 110

Poster **120**

Ökotoxikologie von Bauchemikalien 121

Biotest-Normungsverfahren 125

Sedimentökotoxikologie 128

Aquatische Ökotoxikologie 133

Terrestrische Ökotoxikologie 156

Bodenökotoxikologie 160
Passive Sammler 162
Umweltchemie 163
Monitoring von Oberflächengewässern 170
Toxizität von Chemikalienmischungen 175
Risikobewertung Pestizide 180
Multiple Stressoren 185
Endokrine Disruption 187
Mikroplastik in der aquatischen Umwelt 192
Abwasserbehandlung und –toxizität 195
Erstautoren/Presenter-Verzeichnis 203

Danksagung

Wir danken folgenden Firmen und Institutionen für ihre freundliche Unterstützung

aeschbach CHOCOLATIER

BMG AN ARCADIS COMPANY

harlan®

Huntingdon Life Sciences
Working for a better future

IES Innovative Environmental Services

HilTrade

infochroma.ch
infochroma ag

Waters
THE SCIENCE OF WHAT'S POSSIBLE.™

eawag
aquatic research

SpringerOpen

TECOmedical Group
always your partner

Schweizerische Eidgenossenschaft
Confédération suisse
Confederazione Svizzera
Confederaziun svizra

Eidgenössisches Departement für Wirtschaft, Bildung und Forschung WBF
Bundesamt für Landwirtschaft BLW

Schweizerische Eidgenossenschaft
Confédération suisse
Confederazione Svizzera
Confederaziun svizra

Bundesamt für Umwelt BAFU

ibacon

RLP AgroScience

BAYER
Science For A Better Life

syngenta

knoell
worldwide registration

oekotoxzentrum
centre ecotox

symrise
always inspiring more...

RIFCON
REGULATORY AFFAIRS INFOBROKERAGE FAUNISTICS

SCC
WE CARE FOR YOUR SUCCESS

DR. U. NOACK LABORATORIEN
Your partner for contract research and experimental services since 1986

FCI
FONDS DER CHEMISCHEN INDUSTRIE

Umwelt Bundesamt

Wissenschaftliches Komitee

Inge Werner	Oekotoxzentrum Eawag-EPFL
Carsten Brühl	Universität Landau
Michael Burkhardt	Hochschule für Technik Rapperswil
Natalie Chèvre	Universität Lausanne
Otto Daniel	Agroscope
Rolf Düring	Universität Giessen
Beate Escher	UFZ Leipzig
Andreas Häner	BMG Engineering
Henner Hollert	RWTH Aachen
Jörg Öhlmann	Universität Frankfurt
Georg Reifferscheid	BfG Koblenz
Daniel Rensch	AWEL Zürich
Kristin Schirmer	Eawag
Helmut Segner	Universität Bern
Rita Triebskorn	Universität Tübingen
Etienne Vermeirssen	Oekotoxzentrum Eawag-EPFL
Thomas Wintgens	Fachhochschule Nordwestschweiz
Markus Zennegg	Empa

Organisationskomitee

Inge Werner	Oekotoxzentrum Eawag-EPFL
Brigitte Bracken	Oekotoxzentrum Eawag-EPFL
Klaus Peter Ebke	Mesocosm GmbH
Bettina Hitzfeld	Bundesamt für Umwelt
Marion Junghans	Oekotoxzentrum Eawag-EPFL
Cornelia Kienle	Oekotoxzentrum Eawag-EPFL
Katja Knauer	Bundesamt für Landwirtschaft
Christa McArdell	Eawag
Anke Schäfer	Oekotoxzentrum Eawag-EPFL

Grusswort des SETAC-GLB Präsidenten

Liebe Tagungsteilnehmerinnen und Tagungsteilnehmer

Ich begrüsse Sie ganz herzlich zur diesjährigen Jahrestagung des deutschsprachigen Zweiges von SETAC Europe (SETAC-GLB). Es ist mir eine besonders große Freude, dass die 20. SETAC GLB Jahrestagung in der Schweiz stattfindet und damit ein klares Zeichen für die Verankerung im gesamten deutschsprachigen Raum setzt! Die Tagung erwartet uns mit rund 140 Beiträgen in 15 Sessions sowie 3 spannenden Plenarvorträgen. Sie steht unter dem Motto „Ökotoxikologie und Umweltchemie - Von der Forschung in die Praxis".

Der Transfer von Forschung in die Praxis ist sicherlich kein Thema was auf die Ökotoxikologie beschränkt ist, aber durch die Tripartheit unserer Fachgesellschaft natürlich ein idealer Platz diesen voranzutreiben. Schon Leonardo da Vinci wusste „Stets muss die Praxis auf guter Theorie beruhen“ und deshalb freue ich mich auf spannende Vorträge und Diskussionen, aber auch Pausengespräche, im Rahmen unserer Jahrestagung. Dass es nicht immer ein einfacher Diskurs zwischen Vertretern von Behörden, Unternehmen und Wissenschaftlern ist, drückt sich im Aphorismus von Prof. Quadbeck-Seeger aus: „Theorie und Praxis sind Partner, aber nicht immer Freunde“. Umso wichtiger ist der Austausch gerade auf nationaler Ebene im Rahmen der Jahrestagung, denn auf dieser Ebene werden weiterhin wichtige Entscheidungen getroffen und die SETAC GLB bietet mehr Freiraum zum Austausch als größere Tagungen.

Auch die Forschungsförderung ist letztendlich zu einem bedeutenden Teil noch national organisiert. Dort ist die Förde rung der Schadstoffforschung, insbesondere im Bereich der Grundlagenforschung, oft unterrepräsentiert (Hollert et al. Environm. Sci. Eur. 23: 24–34). Erstmals ist es der SETAC GLB gelungen, sich als Fachgesellschaft für die Fachkollegienwahl bei der Deutsche Forschungsgemeinschaft registrieren zu lassen und KandidatInnen vorzuschlagen. Fachkollegien fällen die Entscheidungen über die Förderung von Anträgen, insofern ist die personelle Zusammensetzung wichtig. Allen Wahlberechtigten, d.h. Personen die an deutschen wissenschaftlichen Institutionen arbeiten, empfehle ich deshalb an der Wahl teilzunehmen und SETAC KandidatInnen im Bereich der Ökologie der Tiere und in der Wasserforschung zu unterstützen. Denn Grundlagenforschung bleibt wichtig – oder mit Joseph J. Thompson überspitzt ausgedrückt: „Forschung auf dem Gebiet der angewandten Wissenschaft führt zu Reformen; Forschung auf dem Gebiet der reinen Wissenschaft führt zu Revolutionen.“

Ich freue mich also auf einen angeregten und anregenden Austausch von Fachwissen zwischen Grundlagenwissenschaft und Praxis und danke den Mitgliedern des Organisationskomitees, des Wissenschaftlichen Komitees und unseren Gastgebern am Schweizerischen Zentrum für Angewandte Ökotoxikologie Eawag-EPFL (Oekotoxzentrum) sehr herzlich für die Organisation der Tagung!

Prof. Dr. Ralf B. Schäfer

Grusswort des Organisationsteams

Sehr geehrte Tagungsteilnehmer

Wir begrüssen Sie mit einem herzlichen „Grüezi“ in Zürich!

Die diesjährige Tagung der SETAC GLB wurde federführend vom Schweizerischen Zentrum für Angewandte Ökotoxikologie (kurz: Oekotoxzentrum Eawag-EPFL) organisiert. Das Oekotoxzentrum wurde 2008 gegründet als Anlaufstelle und Drehscheibe für Forschung, Entwicklung, Dienstleistung und Bildung in angewandter, praxisorientierter Ökotoxikologie in der Schweiz. Es bildet es eine Brücke zwischen Forschung und Praxis.

So lautet denn auch das Thema dieser Tagung nicht ganz zufällig „Ökotoxikologie und Umweltchemie: Von der Forschung in die Praxis“. Hierbei ist besonders die Ökotoxikologie noch sehr gefordert. Schreckt doch die Komplexität der biologischen Antworten und ökologische Interaktionen noch viele Regulatoren ab, die auf eine klare und „wasserfeste“ Interpretation der erhobenen Daten angewiesen sind. Die Wissenschaft und Einrichtungen wie das Oekotoxzentrum sind daher gefordert, eine Brücke zwischen Forschung und Praxis zu schlagen. Dazu gehört die Wissensvermittlung, aber auch der effiziente Dialog zwischen Politik und Wissenschaft (siehe Plenarvorträge Prof. Janet Hering und Dr. Michael Schärer). Entwicklung von Testverfahren sind wichtig, jedoch werden sie meist erst durch die Standardisierung (eine Session dazu gibt es am Dienstag) regulatorisch einsetzbar. So ein „Turmbau zu Babel“ erfordert viel Ausdauer, Fach- und Detailwissen, und obwohl sich diese Arbeiten nicht in hochkarätigen Fachzeitschriften veröffentlichen lassen, sind sie doch für die Akzeptanz und Anwendung ökotoxikologischer Methoden unablässig.

Wir hoffen nun, dass wir Ihnen mit 77 Vorträgen und 87 Postern ein spannendes Tagungsprogramm zusammengestellt haben, und dass der Rahmen dieser Tagung Ihnen viel Austausch mit Kollegen, vor allem auch grenzübergreifend, ermöglicht. Dass die Tagung in Zürich stattfindet, hat dazu geführt, dass sich dieses Jahr wesentlich mehr Kollegen aus der Schweiz beteiligen als sonst. Wir freuen uns sehr darüber. Allerdings hat die Vielsprachigkeit der Schweiz auch das wiederholt diskutierte Thema der Tagungssprache Deutsch wieder in den Vordergrund gerückt. Sie werden deshalb bemerken, dass ein grösserer Anteil der Präsentationen auf Englisch gegeben wird.

Wir bedanken uns herzlich für die wertvolle Unterstützung durch den Vorstand und die Geschäftsstelle der SETAC GLB, des Wissenschaftliche Komitees, der Sponsoren und der studentischen Helfer. Vor allem danke ich dem Organisationskomitee für seinen grossen Einsatz für das gute Gelingen dieser Tagung!

Ihnen, liebe Teilnehmer, wünschen wir einen angenehmen Aufenthalt in Zürich, viel Erkenntnisgewinn und wertvolle neue Kontakte!

I. Werner

Dr. Inge Werner

Grusswort des Gesundheits- und Umweltdepartements der Stadt Zürich

Sehr geehrte Damen und Herren

Es freut mich ausserordentlich, als Vertreter der Stadtverwaltung ein Grusswort an die Teilnehmenden der SETAC GLB – Jahrestagung 2015 in Zürich richten zu dürfen.

Wie Sie vielleicht wissen, wurde die Stadt Zürich schon wiederholt in verschiedenen Rankings als Stadt mit der besten Lebensqualität ausgezeichnet. Nun gibt es für die Definition von Lebensqualität keine allgemein anerkannten Normen. Ich wage aber die Behauptung, dass der Zustand der Umwelt – u.a. die Luft- und die Wasserqualität – diese Lebensqualität ganz wesentlich mitbestimmt.

Ich möchte kurz beispielhaft auf die Luftqualität eingehen. Hier wurden in den letzten Jahrzehnten durch ein breit angelegtes Massnahmenpaket grosse Fortschritte erzielt. Unsere Umgebungsluft in der Stadt erfüllt zwar noch immer nicht alle Anforderungen der eidgenössischen Luftreinhalte-Verordnung, die Anzahl und das Ausmass der Grenzwertüberschreitungen haben aber markant abgenommen. Für die Formulierung der erwähnten Massnahmen müssen einerseits die Schadstoffimmissionen und andererseits die Wirkungsmechanismen bei der Entstehung der Schadstoff-Frachten bekannt sein. Nur so können effiziente und effektive Aktivitäten zur Verbesserung der Luftqualität entwickelt werden.

Ökotoxikologie und Umweltchemie bilden das Kerngeschäft der SETAC. Angesichts des immer noch zunehmenden Eintrags von Chemikalien aller Art in die natürliche Umwelt scheint es mir von enormer Bedeutung, das Verhalten und die Auswirkungen dieser Stoffe sorgfältig zu untersuchen. Für eine Umsetzung der im Labor gewonnenen Erkenntnisse in die Praxis ist es wiederum entscheidend, Werkzeuge zu entwickeln, welche z.B. von Behörden im Vollzug des Umweltrechts eingesetzt werden können.

Dem Programm der SETAC GLB-Jahrestagung 2015 entnehme ich, dass eine Brücke zwischen Forschung und Praxis geschlagen werden soll. Praxisrelevante Themen werden im Fokus stehen. Damit leisten Sie einen entscheidenden Beitrag zur nachhaltigen Verbesserung der Umweltqualität.

Ich wünsche Ihnen eine erfolgreiche Jahrestagung 2015 mit spannenden Referaten und vielen interessanten fachlichen und persönlichen Begegnungen.

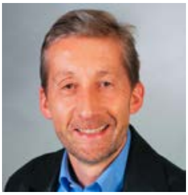

Freundliche Grüsse

Peter Bär
Leiter Abteilung Umwelt, stv. Direktor

Die Gastgeber

Die Tagung wird vom Schweizerischen Zentrum für Angewandte Ökotoxikologie Eawag-EPFL (Oekotoxzentrum) in Dübendorf organisiert, mit Unterstützung der Eawag, des Bundesamts für Landwirtschaft (CH), des Bundesamts für Umwelt (CH) und der SETAC GLB Geschäftsstelle.

Das Oekotoxzentrum Eawag-EPFL ist die Anlaufstelle und Drehscheibe für Forschung, Entwicklung, Dienstleistung und Bildung in angewandter, praxisorientierter Ökotoxikologie in der Schweiz. Sein Ziel ist es, Effekte von Chemikalien auf die Umwelt zu erkennen und zu beurteilen, und Risiken zu minimieren. Mit Schwerpunkten in Wissensmanagement, Wissens- und Technologietransfer bildet es eine Brücke zwischen Forschung und Anwendung. Die Tätigkeiten des Oekotoxzentrum erfolgen in enger Zusammenarbeit mit externen Partnern aus Forschung, Behörden, und Wirtschaft. Der Hauptsitz des Oekotoxzentrums ist an der Eawag, dem Wasserforschungs-Institut des ETH-Bereichs, in Dübendorf. Der zweite Standort mit den Schwerpunkten Boden- und und Sedimentökotoxikologie befindet sich an der EPFL in Lausanne.

Der Tagungsort

Schon vor 7000 Jahren liessen sich erste Siedler am Ausfluss des Zürichsees nieder. Die Römer gründeten in Zürich die Zollstation Turicum. Heute ist die **Stadt Zürich** mit rund 400'000 Einwohnern die grösste Stadt der Schweiz und ihr wirtschaftliches, wissenschaftliches und gesellschaftliches Zentrum. Schon sieben Mal wurde die Stadt am Zürichsee mit Blick auf die Alpen als Stadt mit der weltbesten Lebensqualität ausgezeichnet.

Die Tagung findet auf dem zentralen Campus der **Eidgenössischen Technischen Hochschule (ETH) Zürich** statt, einer der weltweit führenden technisch-naturwissenschaftlichen Universitäten. Sie ist bekannt für ihre exzellente Lehre, eine wegweisende Grundlagenforschung und den direkten Transfer von neuen Erkenntnissen in die Praxis. 1855 von Gottfried Semper erbaut, zählt die ETH Zürich heute rund 18'500 Studierende aus über 110 Ländern, davon 4000 Doktorierende. Forschenden bietet sie ein inspirierendes Umfeld und ihren Studierenden eine umfassende Ausbildung. 21 Nobelpreisträger, die an der ETH Zürich studiert, gelehrt oder geforscht haben, unterstreichen den hervorragenden Ruf der Hochschule..

Allgemeine Informationen

Die Tagung findet auf dem zentralen Campus der ETH Zürich im focusTerra (NO-Gebäude) statt.

Anschrift

focusTerra
Sonneggstrasse 5
8092 Zürich

Anreise zur ETH Zürich

Vom Flughafen Zürich per Zug zum Hauptbahnhof (Zürich HB): Folgen Sie bitte den Wegweisern am Flughafen.

Vom Hauptbahnhof Zürich ab Haltestelle «Bahnhofstrasse/HB»: Tram Nr. 6 (Richtung Zoo) bis Haltestelle «ETH/Universitätsspital», Fahrzeit: ca. 6 Minuten

Vom Hauptbahnhof Zürich ab Haltestelle «Bahnhofplatz/HB»: Tram Nr. 10 (Richtung Flughafen oder Bahnhof Oerlikon) bis Haltestelle «ETH/Universitätsspital» oder Tram Nr. 3 (Richtung Klusplatz) bis Haltestelle «Central» (1 Station), ab «Central» mit der Polybahn (Abfahrt alle drei Minuten) bis zur Polyterrasse, Fahrzeit: ca. 8 Minuten

Sonst: Tram Nr. 6, 9 und 10 bis ETH/Universitätsspital oder Polybahn bis Polyterasse ETH. Da an der ETH Zürich nur wenige Parkplätze zur Verfügung stehen, empfiehlt es sich, mit den öffentlichen Verkehrsmitteln anzureisen. Mehr zu Parkmöglichkeiten an der ETH finden Sie unter www.ethz.ch/de/campus/standorte-anreise/standorte-ZH/HG.html. Die am nächsten gelegenen öffentlichen Parkhäuser sind das Parkhaus Hohe Promenade http://www.parkhaus-hohepromenade.ch/ und die Parkgarage am Central http://www.pls-zh.ch/parkhaus/central.jsp?pid=central mit Tarifen von 4-5 CHF/h.

Für die öffentlichen Verkehrsmittel benötigen Sie ein gültiges Ticket für die Zone 110 (Stadt Zürich). Fahrplan- und Ticketauskünfte erhalten Sie beim Zürcher Verkehrsverbund ZVV www.zvv.ch. Informationen zu Ticketoptionen für den Transport vor Ort finden Sie unter ZVV Tickets www.zvv.ch/zvv/de/abos-und-tickets. Der ZVV bietet auch eine Fahrplan App für Android und iOS Geräte an (http://www.zvv.ch/zvv/de/service/apps/fahrplan-app.html).

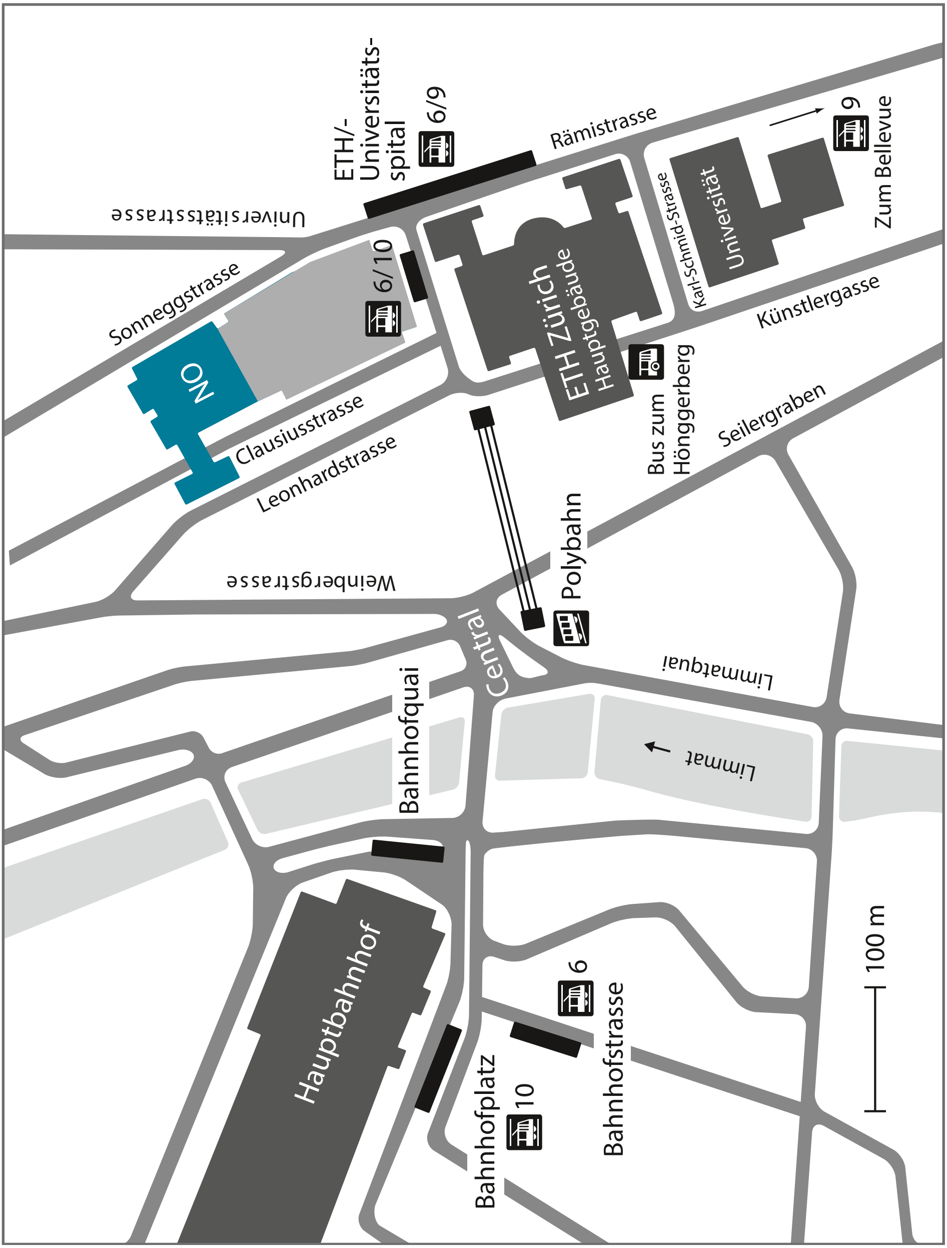

NO= Focus Terra

Allgemeine Informationen

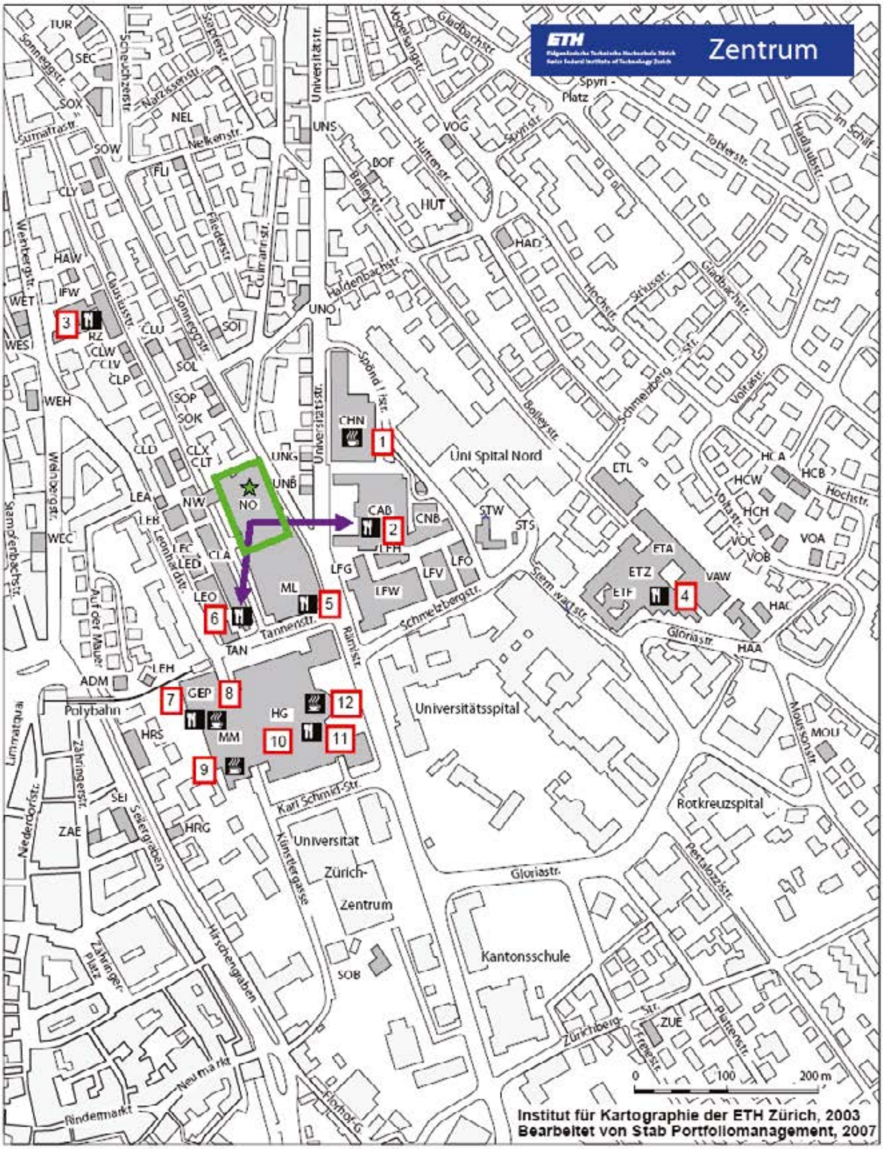

Weg zu den Restaurants

NO (Focus Terra), Ausgang Clausiusstrasse (C-Stock) nehmen, nach links drehen und der Strasse bis an die Hauptstrasse folgen (ca. 150 m) danach Drehtüre verwenden für Clausiusbar (6). Für das Restaurant Foodlab (2) nehmen Sie den Ausgang D-Stock, dann Rämistrasse überqueren.

Tagungsbüro

Das Tagungsbüro befindet sich im Foyer im C-Stock des focusTerra (NO-Gebäude) an der ETH Zürich. Das Büro ist am Montag ab 13:30 Uhr, am Dienstag ab 7:30 Uhr und am Mittwoch und Donnerstag ab 8:00 Uhr geöffnet.

Mittagessen

Die Kosten für das Mittagessen sind im Tagungsbeitrag enthalten Während der Mittagspausen können Sie sich entweder in der Clausiusbar (asiatische Gerichte) oder im Restaurant Foodlab (Pasta und Salatbuffet) verpflegen (siehe Plan auf vorgehender Seite). Die Mensa Polyterasse (7) bietet auch von 17:30-19:30 Uhr günstige Abendessen an. Kaffee, Tee und Gebäck stehen in den Kaffeepausen im Foyer des Tagungsgebäudes bereit.

WLAN

Steht vom 07.09.2015, 06.00 Uhr – 10.09.2015, 20.00 Uhr jedem Teilnehmer zur Verfügung.

Netz anwählen: **WLAN public oder public-5**

Username: **SETAC-GLB15**

Passwort: **Tagung15**

Das Rahmenprogramm

Get Together, Montag, 7. September 2014, 18:30 Uhr

Das Get Together findet am Eröffnungsabend im Foyer des D-Stocks des NO-Gebäudes (focusTerra) statt. Es stehen Getränke und ein kleiner Imbiss bereit. Für Unterhaltung sorgen die Alphornbläser Zürich.

Gesellschaftsabend, Dienstag, 8. September, 20:00 Uhr

Zum Konferenzdinner gehen wir ins historische Zunfthaus Linde Oberstrass www.linde-oberstrasse.ch mit eigener Brauerei. Die Linde Oberstrasse (Universitätsstrasse 91) befindet sich in 10 min Laufdistanz vom Konferenzgebäude. Alternativ Anreise mit Tram 9 oder 10 bis zur Haltestelle Winkelriedstrasse.

Tagungsparty, Mittwoch, 9. September, 20.30 Uhr

Die Tagungsparty findet im Club Mundwerk in Zürich-Oerlikon, Dörflistrasse 76, statt. Für packende Livemusik sorgt die Zürcher Band The Birthday Girls www.the-birthday-girls.com. The Birthday Girls, das sind vier in Zürich lebende Jungs (!) aus vier verschiedenen Ländern

(Österreich, Chile, England, Deutschland), die ihre unterschiedlichen kulturellen und musikalischen Wurzeln zu einer einzigartigen Mischung aus Crossover Indie Rock und Folk verschmelzen.

Für den kleinen Hunger steht ein Pizzawagen vor dem Gebäude. (Essensbon ist im Partybeitrag inbegriffen). Anreise: Von Zürich mit Tram 11 (bis Sternen Oerlikon), 10 oder 14 (jeweils bis Salersteig). Von der Haltestelle in Fahrtrichting rechts in die Allenmoosstrasse einbiegen und dann links in die Dörflistrasse (siehe Karte). An der Kreuzung mit der Tramstrasse befindet sich auf der rechten Seite eine Tankstelle (Agip). Gleich rechts der Tankstelle ist der Club Mundwerk im Untergeschoss einer Baracke.

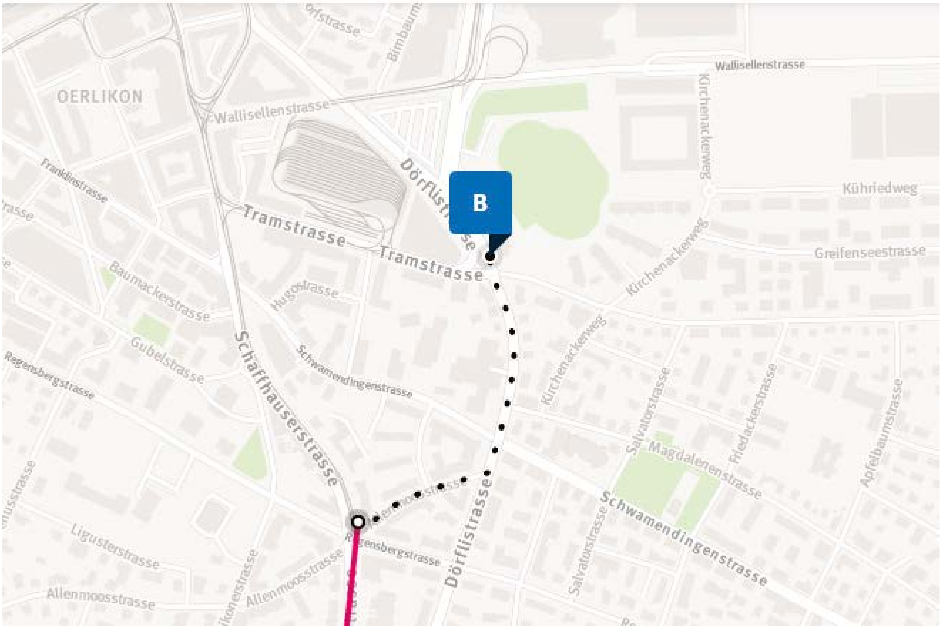

Exkursionen

Die Exkursionen finden am Montag, 07.09. vor Beginn des wissenschaftlichen Programms bzw. am Dienstag, 08.09. und Mittwoch, 09.09. nach Ende der Vorträge statt.

Führung ARA Neugut und Forum Chriesbach

In der **Abwasserreinigungsanlage (ARA) Neugut** in Dübendorf wurde 2014 die die erste grosstechnische Ozonungsanlage in einer Schweizer Kläranlage in Betrieb genommen, um Spurenstoffe aus dem bereits gereinigten Abwasser zu entfernen. Über die nächsten 20 Jahre sollen rund 100 der 700 Kläranlagen der Schweiz mit speziellen Reinigungsverfahren für Mikroverunreinigungen ausgerüstet werden, um die Gewässerqualität zu verbessern. Das Oekotoxzentrum analysiert das neue Verfahren derzeit direkt in der Kläranlage, um wichtige Erkenntnisse für die Praxis zu gewinnen. Zusammen mit der Eawag untersucht es die Effizienz und Stabilität der Ozonung mit nachgeschalteter biologischer Reinigung. Schwerpunkte sind hierbei der Einfluss der Wasserzusammensetzung auf die Entfernung der Mikroschadstoffe und die Effizienz verschiedener biologischer Nachbehandlungsmethoden der Ozonung.

Der Bau des neuen Eawag-Hauptgebäudes – des **Forum Chriesbach** – hat neue Massstäbe im Bereich der nachhaltigen Entwicklung gesetzt. Die baulichen und technischen Massnahmen liegen an den Grenzen des zur Zeit der Planung Machbaren. Beispielsweise verbraucht das Haus, das in den Bürozonen ohne herkömmliche Heizung und Kühlung auskommt, viermal weniger Energie als ein konventionelles Gebäude. Und mit dem Einbau eines ausgeklügelten Sanitärsystems – Urin trennende NoMix WCs, Spülung mit Regenwasser – werden neueste Forschungsergebnisse der Eawag im Praxisalltag erprobt. Die umweltschonende und erneuerbare Produktion und Beschaffung von Strom wird durch eine eigene Photovoltaikanlage und den Einkauf von Ökostrom gewährleistet. Die Investitionen für die energetischen Massnahmen werden mit tieferen Betriebskosten mehr als wettgemacht.

Der Besuch der ARA Neugut wird mit der Besichtigung des Forum Chriesbach kombiniert und findet am **Montag, 07.09. von 13.40 bis 17.00 Uhr** statt. Die Teilnehmenden treffen sich um **13.40 Uhr am Bahnhof Stettbach** und werden dort mit einem Minibus abgeholt. Der Bahnhof Stettbach wird mit S-Bahn S3, S9 oder S12 in 7 min von Zürich HB erreicht und liegt noch in der Stadtzone Zürich.

Führung Erdbebensimulator

Im Tagungsgebäude focusTerra, dem erdwissenschaftlichen Forschungs- und Informationszentrum der ETH Zürich, kann ein Erdbebensimulator besucht werden. Mit dem über drei Tonnen schweren Gerät lässt sich die Bodenbewegung von Erdbeben bis Magnitude 8 simulieren. Der Simulator ist eine Art Containerraum, der mit Tischen und anderen beweglichen Gegenständen eingerichtet ist, und in dem bis zu 10 Personen gleichzeitig Platz finden. Besucherinnen und Besucher können anhand von real aufgezeichneten Erdbebensignalen das Phänomen "Erdbeben" gefahrlos am eigenen Körper erfahren. Die Erdbebensimulation soll nicht nur ein Bewusstsein dafür schaffen, dass auch Europa durch Erdbeben gefährdet ist, sondern auch zeigen, wie man sich bei einem Erdbeben richtig verhält.

Achtung: Der Besuch des Erdbebensimulators erfolgt auf eigene Gefahr. Für Kleinkinder bis zum 5. Geburtstag, Personen mit Rücken- oder Nackenproblemen oder anderweitigen Ge-

brechen sowie Hochschwangere ist der Besuch des Simulators nicht geeignet. Kinder zwischen 5 und 11 Jahren dürfen den Simulator nur in Begleitung besuchen.

Die Führung findet am **Dienstag, 08.09., 18:00 – 19:30 Uhr**, und am **Mittwoch, 09.09., 17:30-19:00 Uhr** statt.

Treffpunkt. Foyer D-Stock

Stadtführung Zürich

Die Stadt Zürich mit rund 400'000 Einwohnern ist die grösste Stadt der Schweiz und ihr wirtschaftliches, wissenschaftliches und gesellschaftliches Zentrum. Besonders reizvoll sind die historische Altstadt und die malerische Lage an Limmat und Zürichsee – Gewässer mit Trinkwasserqualität – mit Blick auf die schneebedeckten Alpen. Auch berühmte Kirchen wie das Frauenmünster mit seinen prachtvollen Fenstern von Marc Chagall sind einen Besuch wert. Schon sieben Mal wurde Zürich als die Stadt mit der weltbesten Lebensqualität ausgezeichnet.

Die Stadtführung wird am **Dienstag, 08.09., 18:00 – 19:30 Uhr** und am **Mittwoch 09.09., 17:30 – 19.00 Uhr** angeboten.

Treffpunkt: Foyer C-Stock, am Registrierungstisch

Übersicht Tagungsprogramm

Montag, 07.09.2015

		Raum
ab 13:30	Öffnung des Tagungsbüros – Registrierung	NO Gebäude, Foyer C-Stock
13:40-17:00	Exkursion: Besichtigung der ARA Neugut (Ozonungsanlage) / des Forum Chriesbach	Bahnhof Stettbach
14:00-18:00	Vorstandssitzung SETAC GLB	NO Gebäude, Raum E11
17:00-18:30	Sitzung der Arbeitsgruppe „Ecological Risk Assessment for Amphibians and Reptiles“	NO Gebäude, Raum E39
18:30	Get together, Apéro und Unterhaltung	NO Gebäude, Foyer D-Stock

Dienstag, 08.09.2015

		Raum
ab 07:30	Registrierung und Posteraufbau	NO Gebäude, Foyer C-Stock
08:30 - 08:40	Begrüssung Gastgeber: Dr. Inge Werner	Grosser Hörsaal C60
08:40 - 08:45	Begrüssung Fachgesellschaft: Prof. Dr. Ralf Schäfer	Grosser Hörsaal C60
08:45 - 09:00	Begrüssung: Herr Peter Bär, Leiter Abt. Umwelt, Gesundheits- und Umweltdepartement der Stadt Zürich	Grosser Hörsaal C60
09:05 - 09:40	Plenarvortrag: Prof. Dr. Janet Hering, Direktorin Eawag	Grosser Hörsaal C60
09:40 - 10:15	Plenarvortrag: Dr. Michael Schärer, Sektionschef, Sektion Gewässerschutz, Schweizerisches Bundesamt für Umwelt	Grosser Hörsaal C60
10:15 - 11:00	Kaffeepause und Poster Session	Foyers C- und D-Stock
11:00 - 12:20	Sessions	C60, C6

		Raum
12:20 - 13:45	Mittagspause	
13:45 - 15:05	Sessions	C60, C6
15:05 - 15:45	Kaffeepause und Poster Session	Foyers C- und D-Stock
15:45 - 16:45	Sessions	C60, C6
16:45 - 17:45	Mitgliederversammlung SETAC GLB	C60
18:00 - 19:30	Exkursion: Führung Erdbebensimulator	Treffpunkt:<br>Foyer D-Stock
18:00 - 20:00	Exkursion: Stadtführung Zürich<br>*Achtung: überschneidet sich mit Konferenzdinner*	Treffpunkt:<br>Foyer C-Stock
20:00	Gesellschaftsabend und Konferenzdinner<br>Zunfthaus „Linde Oberstrass“	Universitäts-strasse 91, Zürich

Mittwoch, 09.09.2015

		Raum
ab 08:00	Registrierung	NO Gebäude,<br>Foyer C-Stock
08:30 - 09:30	Sessions	C60, C6
09:30 - 10:15	Plenarvortrag Emmanuel Frossard, Stv. Leiter, Institut für Agrarwissenschaften, ETH Zürich	Grosser Hörsaal C60
10:15 - 11:00	Kaffeepause und Poster Session	Foyers C- und D-Stock
11:00 - 12:20	Sessions	C60, C6, C44
12:20 - 13:45	Mittagspause	
13:45 - 15:05	Sessions	C60, C6, C44
15:05 - 15:45	Kaffeepause und Poster Session	
15:45 - 17:05	Sessions	C60, C6

		Raum
17:30 - 19:00	Exkursion: Führung Erdbebensimulator	Treffpunkt:<br>Foyer D-Stock
17:30 - 19:30	Exkursion: Stadtführung Zürich	Treffpunkt:<br>Foyer C-Stock
20:30	Studentenparty mit den *Birthday Girls*<br>Club “Mundwerk”	Dörflistrasse 76,<br>Zürich-Oerlikon

Donnerstag, 10.09.2015

		Raum
ab 08:00	Registrierung	NO Gebäude,<br>Foyer C-Stock
08:30 - 09:30	Sessions	C60, C6
09:30 - 10:05	Kaffeepause und Poster Session	Foyers C- und D-<br>Stock
10:05 - 10:35	Preisverleihung: Beste Diplom/Master-/Doktorarbeit 2014	C60
10:35 - 11:55	Sessions	C60, C6
12:00 - 12:30	Preisverleihung: Beste Konferenzbeiträge<br>von Nachwuchswissenschaftlern und Verabschiedung	Grosser Hörsaal<br>C60
12:30	Mittagessen und Ende der Tagung	

Sessions

Dienstag, 08.09.2015

	Grosser Hörsaal C60	
**08:30**	**Eröffnung der Tagung:**<br>Gastgeber: Dr. Inge Werner, Oekotoxzentrum Eawag-EPFL<br>Präsident der Fachgesellschaft: Prof. Dr. Ralf Schäfer<br>Gesundheits- und Umweltdepartement der Stadt Zürich: Herr Peter Bär	
**09:05**	Prof. Janet Hering, Direktorin der Eawag, Dübendorf<br>„Wissensvermittlung: Eine wichtige Massnahme um Forschung und Praxis zu verbinden“	
**09:40**	Dr. Michael Schärer, Leiter der Sektion Gewässerschutz, Bundesamt für Umwelt, Bern<br>„Spurenstoffelimination – Der Schweizer Weg“	
**10:15**	**Kaffeepause und Poster Session**<br>**Foyers C- und D-Stock**	
	**Hörsaal C6**	**Grosser Hörsaal C60**
**11:00**	**Ökotoxikologie von Bauchemikalien**<br>**Chairs: M. Burkhardt, V. Märkl**	**Aquatische Ökotoxikologie I - Methoden**<br>**Chairs: H. Segner, M. Brinkmann**
**11:00**	01. Schoknecht U.: Beobachtungen aus Freilandversuchen mit biozidhaltigen Materialien	12. Böhler S.: Der Embryo der Dickkopf-Elritze als Alternative für die Bewertung von Toxizität und Teratogenität
**11:20**	02. Burkhardt M.: Effektabschätzung von Bioziden im Fassadenabfluss und im Regenwasserabfluss	13. Di Paolo C.: Alcohol, coffee and cigarettes: interlaboratory evaluation of candidate positive control chemicals for behavioural assessment in zebrafish larvae
**11:40**	03. Vermeirssen E.: Organische Beschichtungen im Schweizer Stahlbau und deren Ökotoxizität	14. Hartmann J.: Listen to the clams: Introducing mussel filtration behaviour as a biomarker for environmental pollution
**12:00**	04. Märkl V.: Vergleichende Untersuchungen zum ökotoxikologischen Gefährdungspotential von wasserabdichtenden Polyurethansystemen	15. Dünne M.: AquaHab®, a closed aquatic multispecies system for ecotoxicological purposes - past, present and future activities
**12:20**	**Mittagessen**	

**13:45**	**Biotest-Normungsverfahren**<br>**Chairs: N. Caspers, G. Reifferscheid**	**Aquatische Ökotoxikologie II – Transkriptomics**<br>**Chairs: H. Segner, I. Werner**
**13:45**	05. Caspers N.: Strukturen, Prozesse und Akteure der nationalen/internationalen Normung	16. Fischer S.: Effect-Assessment of wastewater effluents by molecular biomarkers in brown trout (*Salmo trutta*)
**14:05**	06. Schindler Y.: Bedürfnisse und Anforderungen an Biotests aus regulatorischer Sicht	17. Quesada-Garcia (Segner H.): Immunomodulatory activity of thyroid-active compounds in rainbow trout (*Oncorhynchus mykiss*)
**14:25**	07. Buchinger S.: Turmbau zu Babel (1. Stock)	18. Beauvais-Flück R.: Effects of methylmercury on aquatic primary producers: a transcriptomic approach
**14:45**	08. Vermeirssen E.: Turmbau zu Babel (2. Stock)	19. Koglin S.: Characterization of transcriptional responses to dioxins and dioxin-like contaminants in roach (*Rutilus rutilus*) using whole transcriptome analysis
**15:05**	**Kaffeepause und Poster Session**<br>**Foyers C- und D-Stock**	
**15:45**	**Sedimentökotoxikologie**<br>**Chairs: H. Hollert, D. Becker**	**Aquatische Ökotoxikologie III**<br>**Chairs: H. Segner, C. Kienle**
**15:45**	09. Brinkmann M.: Ergebnisse des Projektes DioRAMA - Erfassung Dioxin-ähnlicher Wirkung in Sediment und Biota zur Sedimentbewertung	20. Di Paolo C.: The NORMAN interlaboratory study on biotesting of spiked water extracts
**16:05**	10. Brinke M.: Development and application of Sediment Quality Guidelines (SQG) for quality assessment of fine freshwater sediments	21. Feiner M. (Beggel S.): Increased reverse osmosis concentrate toxicity following application of antiscalants - Acute toxicity tests with the amphipods *Gammarus pulex* and *Gammarus roeseli*
**16:25**	11. Schiwy S.: Entwicklung und Anwendung von Sedimentkontakttests zur Untersuchung des bioverfügbaren Schädigungspotentials in Zebrabärblings - (*Danio rerio*) Embryonen	22. Jagodzinski L. S.: Energy ashes from virgin wood fuel show different potentials for growth promotion and toxicity in *Lemna minor* (L.)

**16:45**	**Grosser Hörsaal C60**<br>**Mitgliederversammlung SETAC GLB**
**18:00**	**Exkursionen**
**20:00**	**Konferenzdinner**

Mittwoch, 09.09.2015

	**Hörsaal C6**	**Grosser Hörsaal C60**
**08:30**	**Terrestrische Ökotoxikologie**<br>**Chairs: A. Aldrich, F. Fischer**	**Monitoring von Oberflächengewässern**<br>**Chairs: B. Escher, R. Triebskorn**
**08:30**	23. Eschenbach E.: Bestimmung der Wirksamkeit von Netzmitteln (Aceton, Tween80, Triton X-100 und Etalfix) und ihr Einfluss auf die akute Toxizität von Dimethoat anhand von *Bombus terrestris* und *Osmia bicornis* Kontaktstudien	38. Ochsenbein U.: Mikroverunreinigungen in bernischen Gewässern: Belastungssituation und neue ökotoxikologische Beurteilung der Risiken
**08:50**	24. Aldrich A.: Ergebnisse des Schweizer Workshops zu Amphibien und Pflanzenschutzmitteln	39. Wittmer I.: Beurteilungskonzept für Mikroverunreinigungen aus diffusen Einträgen
**09:10**	25. Kaufmann K.: Ecotoxicology of amphibians - adaption of dermal *in vitro* test systems to amphibian skin	40. Kienle C.: EcoImpact - Auswirkungen von Mikroverunreinigungen aus Abwasser auf Fliessgewässerökosysteme: ökotoxikologische und chemische Untersuchungen an 24 Schweizer Gewässern
**09:30**	**Grosser Hörsaal C60**<br>**Prof. Emmanuel Frossard, Institut für Agrarwissenschaften, ETH Zürich**<br>**„Soil health and its significance for management"**	
**10:15**	**Kaffeepause und Poster Session**<br>**Foyers C- und D-Stock**	

	Hörsaal C6	Grosser Hörsaal C60	Hörsaal C44
**11:00**	**Bodenökotoxikologie**<br>**Chairs: R.-A. Düring, S. Campiche**	**Monitoring von Oberflächengewässern**<br>**Chairs: B. Escher, R. Triebskorn**	**Multiple Stressoren**<br>**Chairs: I. Werner, M. Korkaric**
**11:00**	26. Wächter D.: Long-term monitoring of organic pollutants in soil: a conceptual approach to select pesticides potentially accumulating in soil	41. König M.: Water Quality Screening for Endocrine Disruption and Adaptive Stress Responses by *in vitro* bioassays in the River Danube in Serbia	53. Liess M.: Kombinierte Wirkung von Schadstoffen und Umweltstressoren
**11:20**	27. Chiaia-Hernandez A.: Prioritizing organic contaminants in Swiss soils using environmental partitioning and high resolution mass spectrometry	42. Berger E.: Bestimmung von kritischen chemischen Konzen-trationen für benthische Invertebraten an Hand von deutschlandweiten Monitoring Daten	54. Segner H.: Combined effects of parasites, climate and pollution on brown trout, *Salmo trutta*, in Swiss rivers
**11:40**	28. Campiche S.: Wood preservatives in Switzerland: from market analysis to soil organisms ecotoxicity testing	43. Kattwinkel M.: Wie stressor-spezifisch sind traitbasierte Indizes?	55. Tellenbach (Spaak, P.): Cyanobacteria and *Daphnia* parasite epidemics
**12:00**	29. Spann N.: Möglichkeiten der Metabolomik in der Ökotoxi-kologie - Effekte von Kupfer auf das Metabolom der Nematoden *Caenorhabditis elegans* und *Panagrellus redivivus*	44. Heye K.: Vergleichende Toxizitätsuntersuchung von Carbamazepin und seiner Hauptmetaboliten gegenüber *Chironomus riparius*	56. Straub L.: Time-lag effects of combined pesticide and parasite stressors on honey bees
**12:20**	**Mittagessen**<br>**ETH Mensa**		
**13:45**	**Passive Sammler**<br>**Chairs: E. Vermeirssen, B. Becker**	**Risikobewertung Pestizide**<br>**Chairs: N. Chèvre, K. Knauer**	**Aquatische Ökotoxikologie IV – Mechanismen**<br>**Chairs: I. Werner, E. Simon**
**13:45**	30. Zennegg M.: PDMS Passivsammler ein ausgezeichnetes Werkzeug zur Identifikation von PCB Punktquellen in Gewässern	45. Knauer K.: Vom Monitoring zur Regulierung von Pflanzenschutzmitteln	57. Pillai S.: Mechanistic and systemic understanding of the toxicity of manufactured nanomaterials in *Chlamydomonas reinhardtii*

**14:05**	31. Becker B.: Anwendung von Passivsammlern für das Monitoring hydrophober organischer Schadstoffe in Flüssen	46. Fulda B.: Vergleichbarkeit modellierter PSM Konzentrationen mit Daten aus dem Schweizer Gewässermonitoring	58. Kirla K.T.: Toxicokinetics and internal distribution of cocaine in zebrafish larvae: unexpected accumulation and retention in the eyes
**14:25**	32. Tlili A.: Combining passive sampling technologies with toxicity tests to assess effects of micropollutants from wastewater effluents on periphyton	47. Ross-Nickoll M.: Biodiversität und strukturelle Vielfalt in der Agrarlandschaft - eine übergeordnete Perspektive auch für die Pestizidzulassung?	59. Brettschneider D.: Einfluss unterschiedlicher Futtermengen auf die Reproduktion und den Energiestoffwechsel von *Potamopyrgus antipodarum* unter chronischer Schadstoffexposition
**14:45**	33. Fischer F.: Passive dosing of hydrophobic organic chemicals in toxicity tests with *Caenorhabditis elegans*	48. Mathis M.: Nachhaltigkeits-bewertung von Insektiziden gegen Rapserdfloh	60. Rehberger K.: *In vitro* assessment of immunotoxic potentials of chemicals for rainbow trout (*Oncorhynchus mykiss*)
**15:05**	**Kaffeepause und Poster Session**<br>**Foyers C- und D-Stock**		

	**Hörsaal C6**	**Grosser Hörsaal C60**
**15:45**	**Umweltchemie**<br>**Chairs: A. Schäffer, M. Zennegg**	**Risikobewertung Pestizide**<br>**Chairs: N. Chèvre, K. Knauer**
**15:45**	34. Schäffer A.: Non-extractable residues classification of chemicals in soil	49. Knillmann S.: Klimavariabilität und Insektizid-Anwendung - Indikatoren für kurz-und langfristige Expositions-Vorhersagen
**16:05**	35. Schinkel L.: Distribution patterns of synthetic pyrethroids in estuarine and delta sediments in Europe	50. Schäfer R.: The ecotoxicological ivory tower, or how ecotoxicology could increase its relevance
**16:25**	36. Düring R.-A.: Katalytischer Abbau persistenter organischer Schadstoffe - Wettlauf der Analytik mit dem Transformationsgeschehen	51. Perseke L.: A critical review of prochloraz effects in fish<br>*Dieser Vortrag ist vom Prüfungsausschuss des Postgradualstudiengang zur Fachökotoxikologin (SETAC GLB GdcH) für auszeichnungswürdig befunden.*

**16:45**	37. Zennegg M.: Hohe PCB Kontamination detektiert in Kalbfleisch aus extensiver Produktion - eine Fallstudie	52. Vallotton N.: Use of the Maximum Cumulative Ratio (MCR) as a tool for prioritization of potential exposures to mixtures of Plant Protection Products: a case study based on surface water monitoring in the USA
**17:30**	**Exkursionen**	
**20:30**	**Studentenparty**	

Donnerstag, 10.09.2015

	**Hörsaal C6**	**Grosser Hörsaal C60**
**08:30**	**Endokrine Disruption**<br>**Chairs: J. Oehlmann, S. Schwarz**	**Abwasserbehandlung und -toxizität**<br>**Chairs: T. Wintgens, D. Rensch, S. Giebner**
**08:30**	61. Schönborn A.: Messung estrogener Aktivität von oxidierten Wässern mit dem planar-YES Bioassay - ein Beitrag zur Methodenentwicklung	68. Wunderlin P.: Testverfahren zur Beurteilung der Behandelbarkeit von Abwasser mit Ozon
**08:50**	62. Schlotz N.: Mixture effects of endocrine active chemicals as determined by human cell based *in vitro* bioassays	69. Teichler R.: Erste Schweizer Kläranlage mit Ozonung: Verbleib von Spurenstoffen und Entstehung von Transformationsprodukten
**09:10**	63. Geiss S.: Effekte endokriner Disruptoren auf die Reproduktion von *Potamopyrgus antipodarum*	70. Kienle C.: Erste Schweizer Kläranlage mit grosstechnischer Ozonung: Ökotoxikologische Untersuchungen zur Beurteilung der Ozonung und verschiedener Nachbehandlungen
**09:30**	**Kaffeepause und Poster Session**<br>**Foyers C- und D-Stock**	
	**Grosser Hörsaal C60**	
**10:05**	**Preisverleihung der SETAC GLB:**<br>**Beste Diplom- oder Masterarbeit und beste Doktorarbeit 2014**<br>**Vorträge der Preisträger**	

	Hörsaal C6	Grosser Hörsaal C60
**10:35**	**Mikroplastik in der aquatischen Umwelt**<br>**Chairs: J. Muncke, K. Heye**	**Abwasserbehandlung und -toxizität**<br>**Chairs: T. Wintgens, D. Rensch, S. Giebner**
**10:35**	64. Duis K.: Mikrokunststoffe in der Umwelt: Ergebnisse einer umfassenden Literatur-studie zu Eintragsquellen, Umweltkonzent-rationen, Aufnahme und Effekten in Orga-nismen	71. Triebskorn R.: Advanced wastewater treatment and its contribution to ecosys-tem health: Long-term and short-term effects of powdered activated carbon filtering in effluent-influenced surface waters
**10:55**	65. Storck F.R.: Analytik und Vorkommen von Mikroplastik in Binnengewässern	72. Giebner S.: Efficiency of advanced wastewater treatment technologies - Do we need a further treatment step?
**11:15**	66. Bandow N.: Einfluss der Alterung auf die Freisetzung von Schadstoffen aus Mikroplastik	73. Svojitka J.: Process performance of a PAC-UF system for the removal of mi-cropollutants from a biologically treated municipal wastewater
**11:35**	67. Faure F.: Microplastics in Swiss surface waters and going upstream: nature, concen-trations, interaction with pollutants	74. Becker D.: Enzymatic degradation of antibiotics in wastewater - A comparison between chemical analysis and ecotoxi-cological evaluation
	**Grosser Hörsaal C60**	
**12:00**	**Preisverleihung: Beste Konferenzbeiträge von Nachwuchswissenschaftlern & Verabschiedung**	
**12:30**	**Mittagessen & Ende der Tagung**	

Ecotoxicology
Biodegradability
Analytical Chemistry
Phys. & Chem. Properties
Residue Analysis & Field Studies
Metabolism Studies
Environmental Fate
Mesocosm Studies
Bioaccumulation
Efficacy Testing

DR. U. NOACK LABORATORIEN

Your partner
for contract research
and experimental services
since 1986

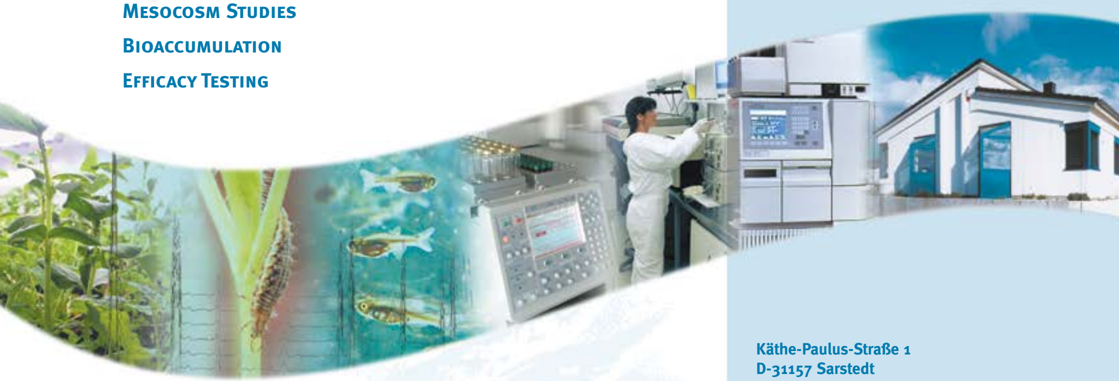

In compliance with GLP

Käthe-Paulus-Straße 1
D-31157 Sarstedt
Tel.: +49 (0) 50 66 / 70 67-0
Fax: +49 (0) 50 66 / 70 67-89
info@noack-lab.de
www.noack-lab.de
www.reach-alliance.de

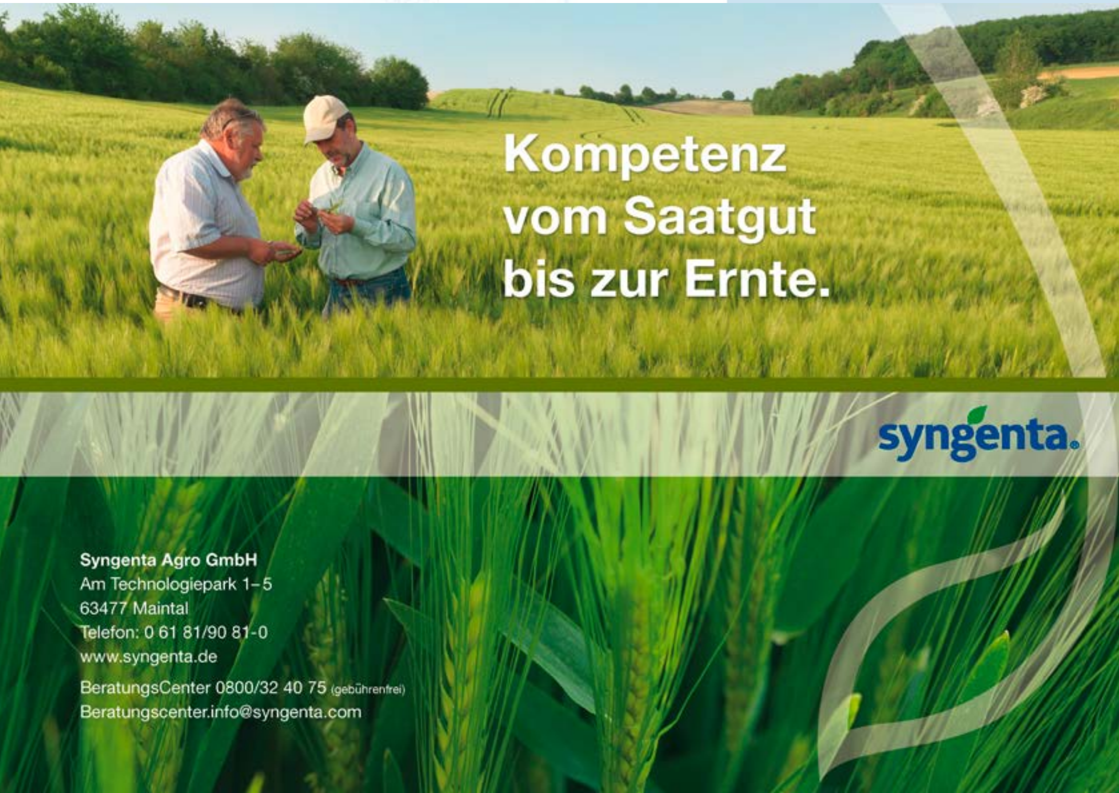

DISCOVER THE WORLD OF SCC

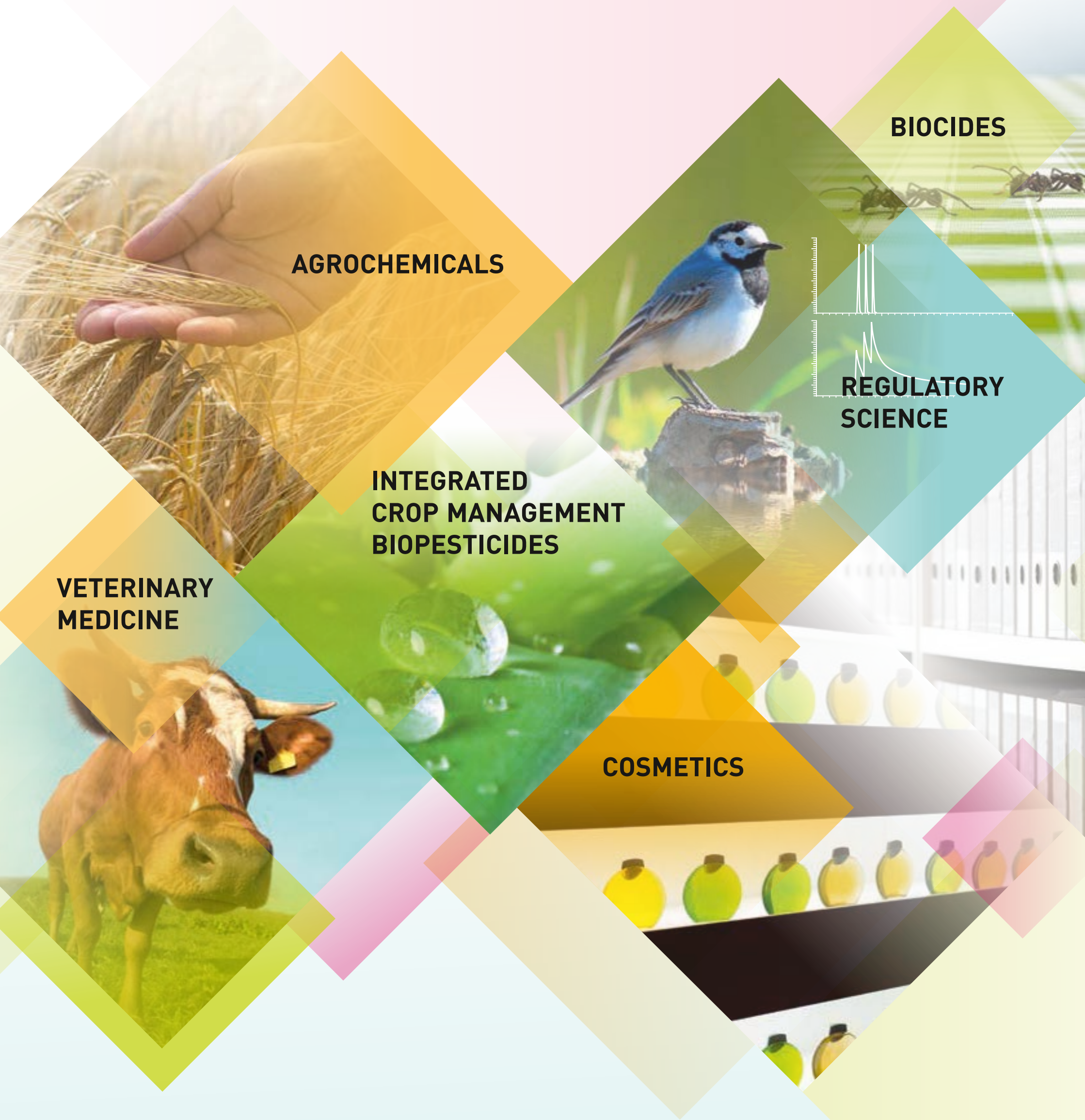

SCC Scientific Consulting Company
Chemisch Wissenschaftliche Beratung GmbH
Am Grenzgraben 11 • 55545 Bad Kreuznach • Germany
monika.hofer@scc-gmbh.de • www.scc-gmbh.de

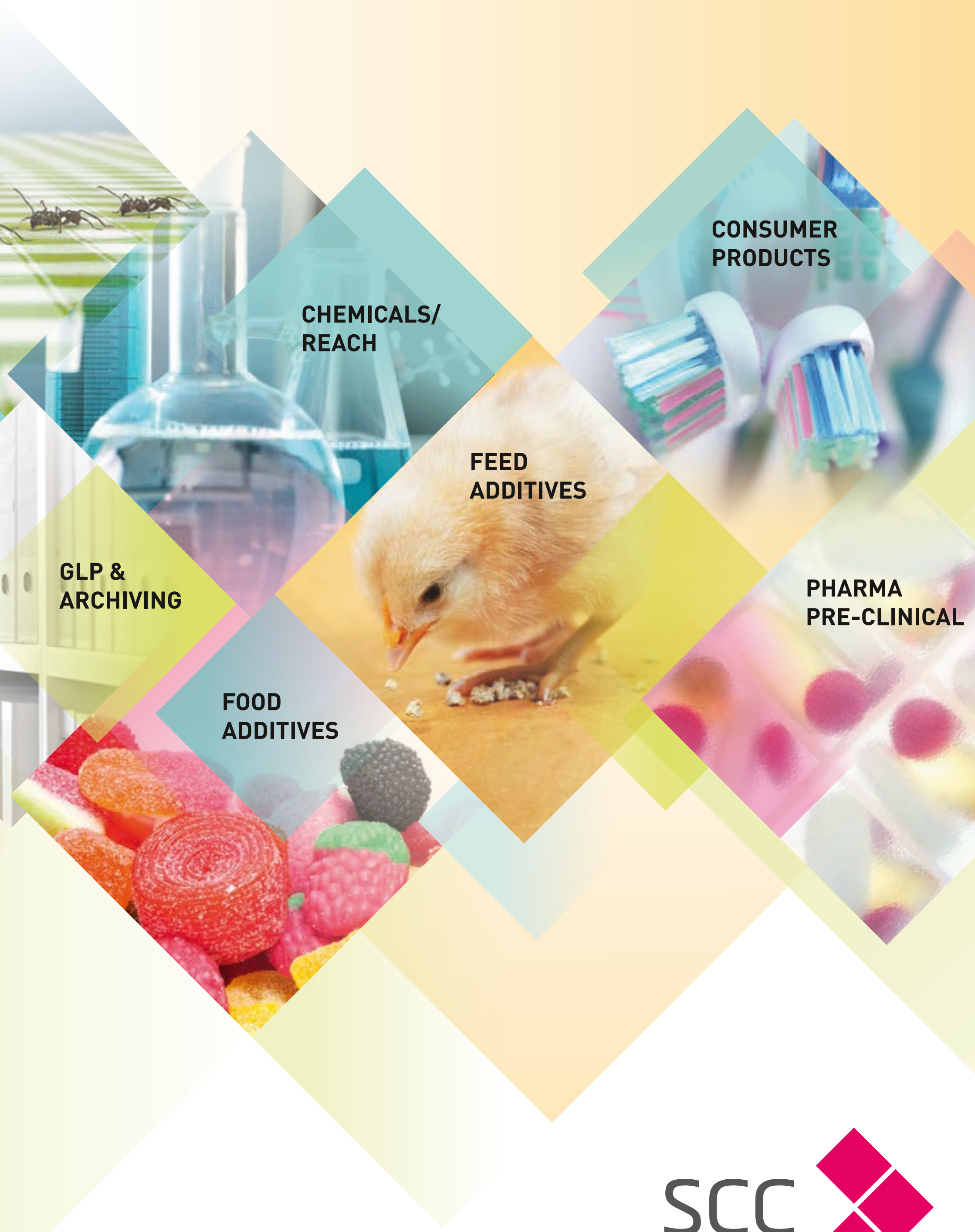

SCC
WE CARE FOR YOUR SUCCESS

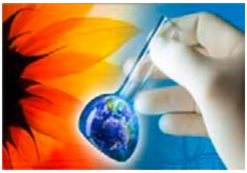

International Conference
Sustainable Chemistry: the way forward

Berlin , 24-25th September 2015

Umwelt Bundesamt

This international conference aims to support Sustainable Chemistry and its role as a key element of the future development of societies and is jointly organised by the German Federal Ministry for the Environment (BMUB) and the German Federal Environment Agency (UBA) in cooperation with the Green Chemistry Commerce Council (GC3) and the Deutsche Bundesstiftung Umwelt (DBU).

Numerous applications of Sustainable Chemistry have already demonstrated that new approaches can be developed to protect human health and the environment while at the same time generating economic benefits. However, in an increasingly globalised economy, it is insufficient to develop Sustainable Chemistry only on regional or national levels. Most of the impacts on human health and the environment occur at a global scale – and often require mitigation approaches on global level. Current use patterns of materials and energy are unsustainable and rapidly depleting our planet's available resources. The increasing consumption of goods is leading to more waste and pollution, overstraining the world's ecosystem. These issues will be discussed in a high-level panel in conjunction with the 2020 goals of the Strategic Approach to the International Chemicals Management.
Also, a concept for Sustainable Chemistry will be presented and discussed with international experts and decision makers in order to exchange about opportunities, barriers and needs. Discussions will be stirred by panel discussions and elaborated in break-out groups addressing emerging topics in sustainable chemicals management and production, illustrated by best-of examples.

Please visit our website for more information: http://www.umweltbundesamt.de/en/topics/chemicals/chemicals-management/sustainable-chemistry/sustainable-chemistry-conference-2015-start.

RLP AgroScience

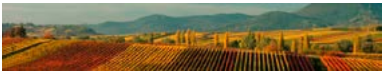

Institut für Agrarökologie

Forschung für

- den Einklang von Ökologie und Ökonomie an der Nahtstelle von Land-bewirtschaftung und Umwelt
- eine realitätsnahe Beurteilung des Verbleibs und der Auswirkung von Pflanzenschutzmitteln sowie anderer anthropogener Einflüsse auf die Umwelt
- die Entwicklung neuer Methoden der Landschaftsanalyse und -modellierung
- die Vermeidung von Abwasser und Abfall sowie die energetische Nutzung von organischen Reststoffen
- technische Innovationen zur umweltgerechten Landbewirtschaftung

http://ifa.agroscience.de

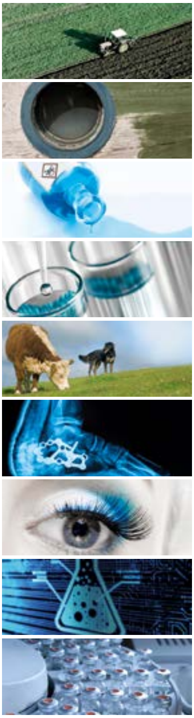

knoell worldwide registration

- Agrochemicals
- Biocides
- Industrial Chemicals
- Cosmetics
- Food Contact Materials
- Food and Feed Additives
- Medical Devices
- Biopharmaceuticals
- Veterinary Pharmaceuticals
- Training
- Product Safety

knoell provides full-services in the areas of regulatory affairs, product safety and consulting.

Whether you need complete registration dossiers, risk assessments or safety data sheets – we are ready to support you and your business. Contact us now!

We have been an independent service provider for the agrochemical, industrial chemical and pharmaceutical industry since 1996, and now have over 450 employees at sites in the EU, Asia and the USA.

Dr. Knoell Consult GmbH I info@knoell.com I **www.knoell.com**

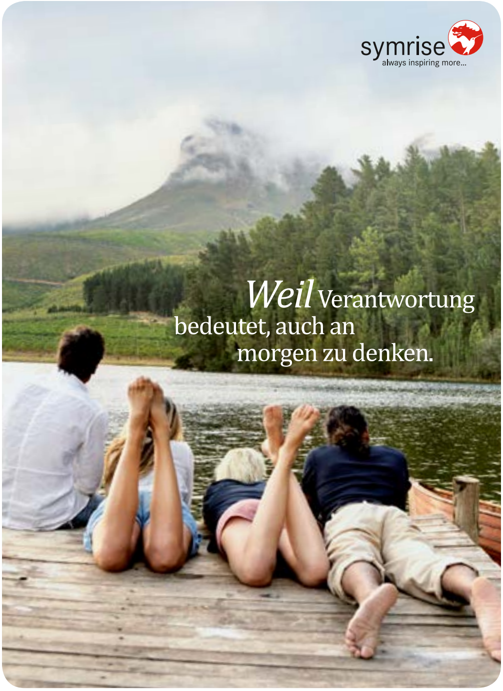

Wir bei Symrise wissen, dass Zukunft auch Verantwortung bedeutet. Deshalb ist Nachhaltigkeit ein wesentlicher Bestandteil unserer Unternehmensstrategie und fest in unseren Werten, unserer Organisation und unserem Handeln verankert. **www.symrise.com**

ibacon

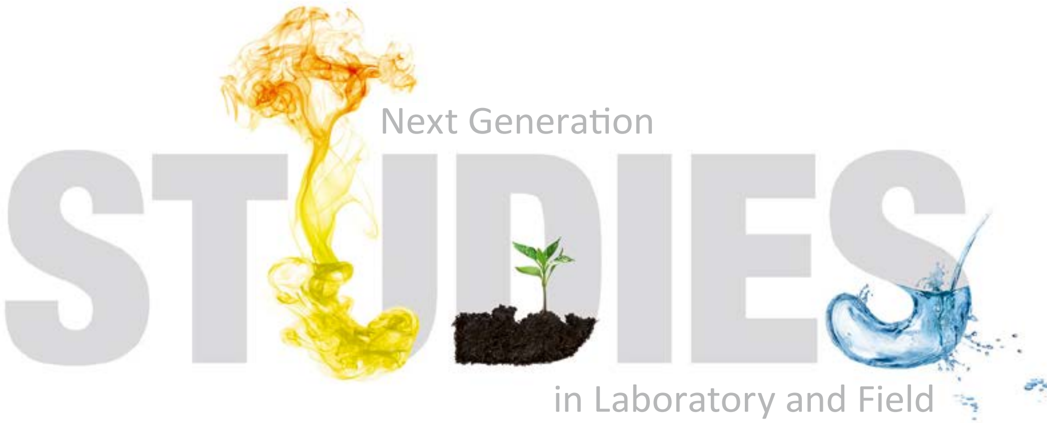

Guiding you safely

As one of the leading CROs, we support our customers in the complex process of contract research. Integrity, trustworthiness and reliability as well as our focus on quality and the customer are the trademarks of our operation.

Core Competences:

- Plant Protection
- Biocides
- Human Medicinal Products
- Veterinary Medicinal Products
- REACh GHS / CLP

We meet your needs:

- Aquatic Ecotoxicology
- Terrestrial Ecotoxicology
- Environmental Fate
- Analytical Chemistry
- Physical-Chemical Properties
- Ecological Modelling

ibacon

If you have specific questions or wish to get to know more about our general portfolio, visit our website or give us a call.

ibacon GmbH | Sabrina Westphal | Head of Business Development
Arheilger Weg 17 | 64380 Rossdorf | Germany
Phone +49 6154 697 238 | info@ibacon.com | www.ibacon.com

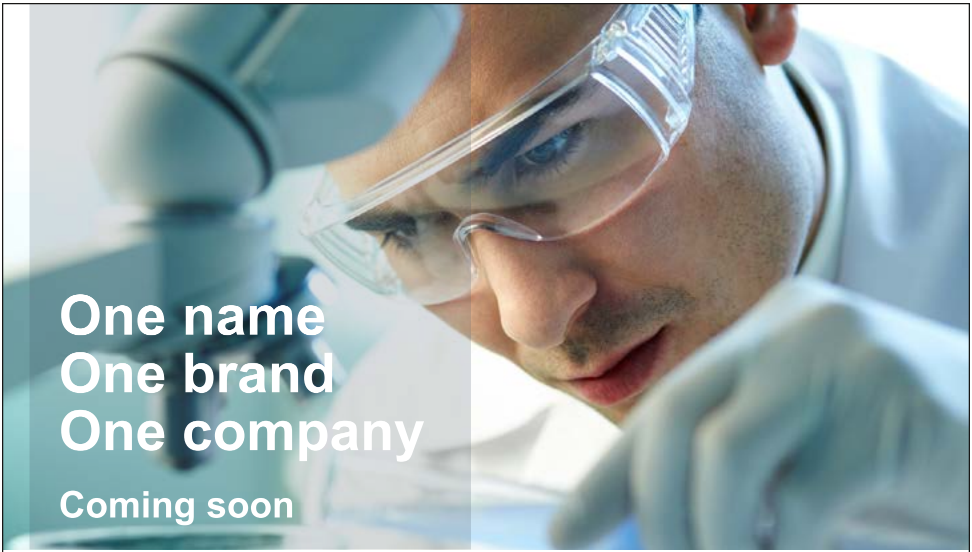

www.huntingdon.com
www.harlan.com

Huntingdon Life Sciences
Working for a better future

harlan

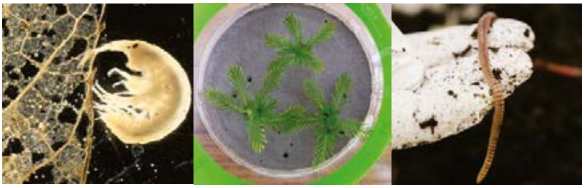

oekotoxzentrum
centre ecotox

Schweizerisches Zentrum für angewandte Ökotoxikologie
Centre Suisse d'écotoxicologie appliquée
Eawag-EPFL

Brücke zwischen Forschung und Anwendung

- Plattform für Experten aus Praxis, Behörden, Industrie und Wissenschaft
- Entwickeln und Optimieren von praxisrelevanten Test- und Analysemethoden
- Risikobewertung von Schadstoffen, Bewerten von Umweltproben

Weiterbildung

- Informieren über den neuesten Wissensstand in der Ökotoxikologie
- Weiterbildungskurse für Fachleute aus der Praxis

Beratung

- Mitwirken in nationalen und internationalen Gremien
- Bearbeiten externer Spezialaufträge und Projekte
- Erteilen von Fachauskünften

www.oekotoxzentrum.ch

Plenarvorträge

Prof. Dr. Janet Hering

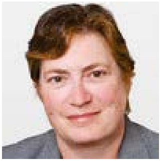

is the Director of the Swiss Federal Institute of Aquatic Science & Technology (Eawag), Professor of Environmental Biogeochemistry at the Swiss Federal Institute of Technology, Zürich (ETHZ) and Professor of Environmental Chemistry and Scientific Director of the Center for Risk Analysis and Risk Governance (CRAG) at the Swiss Federal Institute of Technology, Lausanne (EPFL).

Prof. Dr. Hering's research interests include the biogeochemical cycling of trace elements in natural waters, water treatment technologies for the removal of inorganic contaminants from drinking water, and the management of water resources and water infrastructure. Her research includes both laboratory and field experimental studies. She has been a member of the faculty of the California Institute of Technology and of the University of California, Los Angeles. She is a past recipient of the U.S. National Science Foundation's Young Investigator Award and Presidential Faculty Fellows Award. She has served as an Associate Editor for the journal Environmental Science & Technology and is currently a member of the Board of Reviewing Editors for Science.

Dr. Michael Schärer

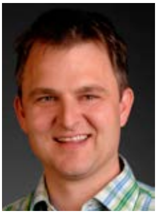

ist aufgewachsen am Zürichsee, studierte Umweltnaturwissenschaften an der ETH Zürich. Nach seiner Doktorarbeit an der ETH, arbeitete er zwei Jahre in England, bevor er 2005 ans BAFU kam. Er befasst sich seither mit dem Themen Spurenstoffe im Abwasser, Abwasserreinigung und Siedlungsentwässerung. Seit Mai 2014 ist er Leiter der Sektion Gewässerschutz die sich um die Themenbereiche Abwasserentsorgung und planerischer Grundwasserschutz kümmert.

Prof. Dr. Emmanuel Frossard

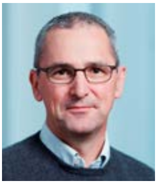

Emmanuel Frossard got his degree of "ingénieur agronome" (MSc in agricultural sciences) and his PhD in agricultural sciences from the Ecole Nationale Supérieure d'Agronomie et des Sciences Alimentaires (ENSAIA) in Nancy (France). He was a postdoctoral fellow at the Saskatchewan Institute of Pedology in Saskatoon, Canada for 2 years. In 1988, he became a senior scientist in soil science at the ENSAIA. In 1994, he was elected associate professor of Plant Nutrition in the Institute of Plant Sciences and the Department of Agricultural and Food Sciences of the ETH Zurich where he became full professor in 2000. Following some restructurations he is now Professor and in the Institute of Agricultural Sciences of the Department of Environmental Systems Science at ETH Zurich.

His work focuses on nutrient cycling in cropping and grassland systems. Together with his Group, he conducts process-oriented research at levels ranging from the molecule to the field, to understand drivers controlling nutrient fluxes and to propose integrated nutrient management schemes that will contribute to the development of ecologically efficient agricultural systems both in temperate and tropical environments. He is currently president of the steering committee of the Swiss national research program on soil as a resource (NRP 68).

Wissensvermitlung: Eine wichtige Massnahme Forschung und Praxis zu verbinden

Prof. Dr. Janet Hering, Direktorin, Eawag, Dübendorf, Schweiz

Man hört oft Klagen, dass die Forschungsergebnisse zu wenig Einfluss auf Praxis und Politik haben. Gleichzeitig hört man, dass die Forschung zu wenig Relevanz für Praxis und Politik hat, oder die Forschungsergebnisse schlecht oder nicht zum richtigen Zeitpunkt zugänglich sind. Die Eawag hat viel Erfahrung und manchmal auch Erfolg, ihre Forschung in die Praxis und die Politik zu übertragen. Hier werde ich ein paar Beispiele präsentieren und diskutieren und die Schlüssel-Faktoren für Erfolg untersuchen. Ich schlage vor, dass in Zukunft die Wissensvermittlung in den Institutionen eingebettet sein sollte. Das wird Investitionen von Institutionen, Behörden und Forschungsgeldgebern brauchen.

Spurenstoffelimination – Der Schweizer Weg

Dr. Michael Schärer, Bundesamt für Umwelt, Bern, Schweiz
michael.schaerer@bafu.admin.ch

In den letzten Jahrzehnten wurde in der Schweiz und vielen andern europäischen Ländern mit dem substantiellen Ausbau der Abwasserentsorgungsinfrastruktur die Wasserqualität stark verbessert. Auf Grund der intensiven Nutzung der Wasserressourcen (z.B. durch zunehmende Bevölkerungsdichte, Landwirtschaft, etc.) nimmt aber der Druck auf die Wasserqualität weiter zu. Im Rahmen des Projekts ‚Strategie Micropoll' des Bundesamts für Umwelt (BAFU) wurde gezeigt, dass Gewässer in dicht besiedelten Räumen (z.B. Mittelland) mit relativ hohem kommunalen Abwasseranteil stark belastet sind, und die Ausläufe von Kläranlagen bedeutende Quellen für organische Spurenstoffe darstellen. Technische Massnahmen zur Elimination der Spurenstoffe werden daher (i) bei grossen ARA (Frachtreduktion), (ii) bei ARA die in Fliessgewässer einleiten mit geringer Verdünnung (Schutz der Ökosysteme), sowie (iii) bei ARA die in Gewässer einleiten, das zur Trinkwassergewinnung genutzt wird als sinnvoll erachtet (Schutz der Trinkwasserressourcen).

Im Rahmen von Pilotstudien wurden verschiedene technischen Verfahren zur Elimination von Spurenstoffen aus dem Abwasser evaluiert. Dabei hat sich gezeigt, dass durch den Einsatz von Pulveraktivkohle (PAK) oder Ozon ein breites Spektrum der organischen Spurenstoffe durchschnittlich zu über 80% entfernt werden konnte. Anhand von ökotoxikologischen Begleituntersuchungen konnte zudem bestätigt werden, dass sich die Qualität des behandelten Abwassers durch diese Massnahmen deutlich verbessert, und ne-gative Auswirkungen auf Wasserlebewesen nahezu vollständig eliminiert werden. Die Elimination von organischen Spurenstoffen auf Kläranlagen ist technisch machbar und wird daher als wirksamste Lösung beurteilt zur kurz- bis mittelfristigen Elimination von einer Vielzahl von Spurenstoffen aus dem kommunalen Abwasser.

Basierend auf diesen Erkenntnissen wurde im Rahmen einer Änderung des Gewässerschutzgesetzes (GSchG) in der Schlussabstimmung des National- und Ständerates am 21. März 2014 die Grundlage für eine verursachergerechte Finanzierung geschaffen, und somit die Finanzierung des Ausbaus von rund 100 der 700 Schweizer Kläranlagen geregelt: durch die Erhebung einer bis 2040 befristeten Abwasserabgabe werden 75% der Erstinvestitionen von total 1.2 Milliarden Franken abgedeckt. Die konkrete Umsetzung wird aktuell im Rahmen der Gewässerschutzverordnung (GSchV) präzisiert. GSchG wie auch GSchV werden voraussichtlich am 1.1.2016 in Kraft treten.

Zusätzliche Massnahmen im Bereich der diffusen Stoffeinträge aus Siedlungsflächen , Verkehrswegen und der Landwirtschaft, wie auch Massnahmen an der Quelle und bei Industrieeinleitern sind weitere Elemente der Schweizerischen Strategie zur Reduktion der Spurenstoffe.

Soil health and its significance for management

Prof Dr. Emmanuel Frossard, Group of Plant Nutrition, Institute of Agricultural Sciences, ETH Zurich, Lindau, Switzerland
President of the steering committee of NRP 68 "Soil as a Resource"
www.nfp68.ch

Soils deliver a wide range of ecosystem services to society (food and fiber production, nutrient cycling, regulation of water flow and quality, pest regulation, carbon sequestration and cycling…). However, soils are threatened by many dangers (erosion, soil organic and biodiversity loss, compaction, sealing, nutrient mining, salinity, contamination) that affect the ability of soils to deliver these services. The concept of soil health has been developed to tackle soil degradation and to maintain/improve productivity; soil health is hence a practice-oriented concept. However, soil health has been variably defined in the literature. For Gugino et al. (2009) soil health is turned towards agriculture as it "deals with the integration of the physical, chemical and biological properties of soil for improved productivity and environmental quality". Kibblewhite et al. (2008) define a healthy soil in a broader manner, as a soil "that is capable of supporting the production of food and fiber, to a level and with a quality sufficient to meet human requirements, together with continued delivery of other ecosystem services that are essential for maintenance of the quality of life for humans and the conservation of biodiversity". Soil health assessment requests an appropriate toolbox that can be used in the field. This concept is now used in the tropics, e.g. to map African soils (Shepherd et al., 2015), in North America for the management of agricultural soils (Gugino et al., 2009) and to assess the state of soils in Europe (Kibbelwhite et al., 2008). This presentation will first recall some important soil properties and it will discuss the ecosystem services delivered by soils. It will then show how the concept of soil health can be used to evaluate the state of agricultural soils. Finally specific examples will show how management practices affect soil health and how it can be recovered, however often at the price of trade-offs.

Gugino, B.K., et al. 2009. Cornell Soil Health Assessment Training Manual, Edition 2.0, Cornell University, Geneva, NY

Kibblewhite, M. G. et al. 2008. Soil health in agricultural systems, Phil. Trans. R. Soc. B, 363, 685–701

Shepherd K. D. et al. 2015. Land health surveillance and response: A framework for evidence-informed land management, Agric. Syst. 132, 93–106

Vorträge

Ökotoxikologie von Bauchemikalien

1. Beobachtungen aus Freilandversuchen mit biozidhaltigen Materialien

Schoknecht Ute, Bundesanstalt für Materialfoschung und -prüfung (BAM), Berlin
H. Mathies, BAM; R. Wegner, MPA Eberswalde, Materialprüfanstalt Brandenburg GmbH, Eberswalde

ute.schoknecht@bam.de

Freilandversuche zur Auswaschung von Wirkstoffen aus biozid ausgerüsteten Materialien waren Bestandteil eines vom Umweltbundesamt in Dessau geförderten Forschungsvorhabens. Ein Schwerpunkt waren Untersuchungen mit Farben (Deckanstriche, Produkttyp 7 nach EU Biozidverordnung). Dafür beaufschlagten Hersteller ihre Produkte mit definierten Biozidgemischen. Zusätzlich wurde ein industriell imprägniertes Textil untersucht.

Die Farben wurden in den Laboratorien nach Herstellerangaben auf geeignete Untergrundmaterialien aufgetragen. Die beschichteten Prüfkörper und Textilproben wurden an zwei Standorten in Eberswalde (60 km nördlich von Berlin) und einem Standort in Berlin ausgebracht und für 15 bis 25 Monate der Witterung ausgesetzt. Die Mehrzahl der Prüfkörper war vertikal exponiert, einzelne Prüfkörper wurden horizontal installiert. Das ablaufende Regenwasser wurde aufgefangen und auf die enthaltenen Wirkstoffe analysiert.

Bei vertikaler Exposition ist Schlagregen auf die Testflächen erforderlich, um Ablaufwasser zu erzeugen. Weitere Witterungsparameter bestimmten die tatsächlich ausgewaschenen Wirkstoffmengen für die untersuchten Produkte. Daraus resultiert, dass für vertikal exponierte Flächen die freigesetzten Mengen nicht mit Regenmengen und nur näherungsweise mit der Menge des Ablaufwassers korrelieren. Bei horizontal exponierten Oberflächen wurden geringere Wirkstoffkonzentrationen in den Ablaufwasserproben, aber insgesamt deutlich höhere ausgewaschene Mengen beobachtet, als bei vertikal exponierten Flächen. Ursache sind die deutlich höheren Ablaufwasssermengen.

Emissionsverläufe unterschieden sich sowohl für unabhängig voneinander durchgeführte Versuche als auch für Parallelversuche. Trotzdem lagen die beobachteten Maximalkonzentrationen im Ablaufwasser und die insgesamt ausgewaschenen Wirkstoffmengen in ähnlichen Größenordnungen. Wirkstoffkonzentrationen im Ablaufwasser variierten stark zwischen Regenereignissen. Im Verlauf der Versuche nahmen die Konzentrationen tendenziell ab. Unterschiede zwischen Emissionsverläufen wurden vor allem im späteren Versuchsverlauf deutlich und sind vermutlich durch die Stabilität der einzelnen Wirkstoffe bestimmt. Mehrfach wurde beobachtet, dass Emissionen im Sommer – vereinzelt auch im Winter - sehr gering waren, und dann im Herbst bzw. Frühjahr wieder anstiegen. Wahrscheinlich verursachen sowohl Abbau und Evaporation der Wirkstoffe als auch unterschiedliche Transportprozesse im Material diesen Effekt.

Restgehaltsanalysen für Wirkstoffe in bewitterten Beschichtungen ergaben, dass wesentliche Mengen der untersuchten Stoffe nicht erfasst werden, wenn nur die Mengen in den Ablaufwasserproben und Restmengen in den Farbschichten untersucht werden. Offensicht-

lich sind Abbauprozesse oder Verdunstung bedeutsam für die Bilanzierung der Stoffmengen.

In Freilandversuchen wurden – insbesondere bei vertikal exponierten Prüfkörpern – geringere Mengen ausgewaschen als in Versuchen nach einem genormten Laborverfahren (EN 16105).

2. Effektabschätzung von Bioziden im Fassadenabfluss und im Regenwasserabfluss

Burkhardt Michael, HSR Hochschule für Technik, Rapperswil, Schweiz

Dirk Engelke, Florian Hochstrasser, Olaf Tietje

michael.burkhardt@hsr.ch

Oberflächlich abfliessendes Regenwasser aus urbanen Einzugsgebieten ist in der Regel mit Substanzen aus Baustoffen belastet und kann in gewissen Fällen zu einer Beeinträchtigung der Oberflächengewässerqualität führen. Bislang wenig untersucht ist die potentielle Gewässerbelastung durch den Einsatz von Bioziden in Fassadenbeschichtungen. Diese werden bei Regen ausgewaschen, können in Gewässer gelangen und diese belasten.

Mit einem für die Fragestellung entwickelten Niederschlag-Abfluss-Modells wurde mittels Szenarien eine Grobabschätzung zur Exposition durch zwei in Gebäudefassaden eingesetzte Biozide durchgeführt. Dabei wurden unterschiedliche Expositionsszenarien über 5 Jahre simuliert. Mit dem Modell können auch Einzelereignisse betrachtet werden.

Das hydraulische Modell basiert auf dem Konzept des Linearspeichers und der Grenzwertmethode. Datengrundlagen für das Modell bilden Regen- und Abflussmessungen, welche im Einzugsgebiet erhoben wurden.

Basierend auf dem hydraulischen Modell und den Auswaschungscharakteristiken der Biozide wurde das Verhalten der Biozide im Einzugsgebiet anhand eines Stofftransportmodells ermittelt. Die Effekte im Gewässer können anhand von wirkungsbasierten Qualitätskriterien beurteilt werden. Das Modell ist in der Lage, die Anzahl der Überschreitungen eines Grenzwerts im Gewässer zu zählen und die Dauer der Überschreitung zu kumulieren.

Das Modell eignet sich als Prognoseinstrument, das Potential zur Regenabwasser- und Gewässerbelastung in urbanen Räumen durch Biozide über 5 Jahre abzuschätzen.

3. Organische Beschichtungen im Schweizer Stahlbau und deren Ökotoxizität

Vermeirssen Etienne, Schweizerisches Zentrum für Angewandte Ökotoxikologie Eawag-EPFL, Dübendorf, Schweiz

Andrea Schifferli; Barbara Ganser; Inge Werner; Michael Burkhardt

etienne.vermeirssen@oekotoxzentrum.ch

Organische Beschichtungen aus Kunstharzen (Epoxydharz, Polyurethan), Gummierungen und Kunststoffen (PVC) werden verwendet, um einen langlebigen Korrosionsschutz von Stahlbauteilen zu bewirken. Gemäss einer Marktrecherche zu organischen Beschichtungsprodukten im Stahlhochbau und Stahlwasserbau, die im Rahmen dieses Projektes durchgeführt wurde, sind Epoxydharze die wichtigsten organischen Korrosionsschutzmittel, gefolgt von 2K-Polyurethanen (PUR) und 1K-Polyurethanen (Polyharnstoffe, PU) bzw. 2K-Polyurethanen und Kunstharz-Kombinationen. Mit 4 Marktprodukten wurden Laboruntersuchungen durchgeführt. Dafür wurden Eluate hergestellt und 5 Arten von Biotests zur Abschätzung der Ökotoxizität durchgeführt. Eluate wurden wie folgt hergestellt: Glasplatten (80 x 125 mm) wurden beschichtet und 1 oder 7 Tage getrocknet (Versuch I und II), danach wurden einzelne Platten mit 100 ml Wasser über 7 Tage geschüttelt. Epoxydharze enthalten oft Bisphenol A (BPA) und Bisphenol F (BPF). Diese Stoffe sowie 3 Derivate (z.B. BADGE) wurden im Eluat gemessen.

Eluate von 2 Produkten enthielten in beiden Versuchen wenig Bisphenol; ein drittes Produkt zeigte intermediäre Bisphenol Konzentrationen und ein viertes Produkt zeigte hohe Bisphenol-Werte (ca. 10 mg/L). Letzterer Wert liegt ca. 6000fach über dem vom Oekotoxzentrum Eawag-EPFL vorgeschlagenen Grenzwert von 1.5 µg/L.

Folgende Biotests wurden durchgeführt: Algentests, Biolumineszenztests und Ceriodaphnientests sowie 2 Testsysteme für hormonelle Wirkung (Hefetests und CALUX-Tests). Zwei Produkte waren nicht oder relativ unauffällig in den Biotests. Ein drittes Produkt zeigte eine sehr hohe Toxizität im Biolumineszenztest. Diese Toxizität liess sich keiner Substanz zuordnen. Die hormonelle Wirkung bei diesem Produkt ist nicht durch BPA erklärbar. Ein viertes Produkt zeigte eine auffällig hormonelle Wirkung. Hier dürfte sich der Effekt in beiden östrogenen Wirkungstests und dem anti-androgen Wirkungstest durch die hohe BPA Konzentration erklären lassen, die auch im Ceriodaphnien-Test zu hoher Toxizität führte.

Zurzeit liegt kein Schema vor, an Hand von welchem man Auswaschungsversuche durchführen und Biotest-Resultate für organische Beschichtungen für den Stahlhochbau und Stahlwasserbau beurteilen kann. Das DIBt-Schema für die Bewertung von Eluaten aus Baumaterialien (www.dibt.de) erweist sich als vielversprechender Ansatz für die Einordnung der Biotest-Resultate. Zu beachten ist, dass unsere Auswaschungsmethode ein Worst-Case-Szenario bezogen auf die Anwendungspraxis darstellen dürfte. Für eine abschliessende, realistische und praxisnahe Bewertung braucht es eine passende Funktion, um die Laborversuche auf die Praxis übertragen zu können.

4. Vergleichende Untersuchungen zum ökotoxikologischen Gefährdungspotential von wasserabdichtenden Polyurethansystemen

Märkl Veronika, TU Berlin

V. Märkl1), S. Reddy2), D. Stephan 1), S. Pflugmacher2), A. Reichert 3), E. Meisel 3) 1) Technische Universität Berlin, Institut für Bauingenieurwesen, FG Baustoffe und Bauchemie, Gustav-Meyer-Allee 25, 13355 Berlin/D 2) Technische Universität Berlin, Institut für Ökologie, FG Ökologische Wirkungsforschung und Ökotoxikologie, Ernst-Reuter-Platz 1, 10587 Berlin/D 3) WEBAC-Chemie GmbH, Fahrenberg 22, 22885 Barsbüttel/D

veronika.maerkl@tu-berlin.de

Unter Druck stehendes Wasser kann an Bauwerken, die nur unter hohem Aufwand oder unzumutbarem Eingriff zugänglich sind, speziell an Tunneln oder historischen Baudenkmalen erhebliche Schäden hervorrufen. Die nachträgliche Bauwerksabdichtung erfolgt typischerweise durch Injektion eines Dichtschleiers von innen in das Mauerwerk oder den anstehenden Untergrund. Hier kommen Polyurethanharze zum Einsatz, da diese Stoffklasse hinsichtlich ihrer rheologischen Eigenschaften und bezüglich des Abdichtpotentials je nach Anwendung eingestellt werden können. Da diese Materialien im Anwendungsfall in direktem Kontakt mit dem Grundwasser stehen, müssen potentielle Risiken für die Umwelt ausgeschlossen werden. Ausgelaugte Bestandteile können ansonsten in den Boden, anstehende Wässer und in das Trinkwasser gelangen [1]. Untersuchungen zur Umweltverträglichkeit von entsprechenden Materialien sind somit unabdingbar.

In dieser Studie wurde der Einfluss von drei Katalysatoren auf den Reaktionsfortschritt eines Modell-Polyurethanharzes und deren Umweltauswirkungen verglichen. Der Reaktionsfortschritt wurde mittels FTIR-Spektroskopie bestimmt. Für weitere Aussagen zur Umweltverträglichkeit wurde ein Standtest entwickelt und die erhaltenen Eluate auf Summenparameter (pH-Wert, Leitfähigkeit, TOC) geprüft. Mögliche auslaugbare Bestandteile aus den Katalysatoren, insbesondere Zn, Sn, und Bi wurden über ICP-OES bestimmt. Die gewonnenen Ergebnisse wurden mit dem bisher üblichen Auslaugverfahren, dem inversen Perkolationsversuch [2], verglichen. Zur Einschätzung der Auswirkungen von Polyurethansystemen auf die aquatische Umwelt wurde der nach DIN EN ISO 8692 (Algenwachstumshemmtest) mit der einzelligen Grünalge Scenedesmus subsicatus durchgeführt [3]. Über die gewonnenen Daten können wasserabdichtende Polyurethansysteme hinsichtlich ihrer Umweltauswirkungen eingestuft werden.

Im weiteren Verlauf des Forschungsprojekts sollen nicht nur die Auslaugverfahren verbessert werden, sondern auch das ökotoxikologische Testsystem. Es soll eine Biotestbatterie aufgebaut werden, um die tatsächlichen Auswirkungen auf die aquatische und terrestrische Umwelt beurteilen zu können.

Literatur:

[1] Leitlinie zur hygienischen Beurteilung von organischen Materialien im Kontakt mit Trinkwasser (KTW-Leitlinie), 2008.

[2] E DIN 19631 Elution von Baustoffen - Perkolationsverfahren zur Untersuchung des Elutionsverhaltens von Bodeninjektionsmitteln. 2013.

[3] DIN EN ISO 8692 - Wasserbeschaffenheit - Süßwasseralgen-Wachstumshemmtest mit einzelligen Grünalgen. 2012.

Biotest-Normungsverfahren

5. Strukturen, Prozesse und Akteure der nationalen/internationalen Normung

Caspers Norbert, eco(toxico)consulting

Andreas Paetz DIN Normenausschuss Wasserwesen (NAW)

ecotoxconsulting.nc@t-online.de

Ziele, Aufgaben und Leistungen des DIN (Deutsches Institut für Normung e.V.) bei der nationalen Normung sowie bei der Vertretung nationaler Interessen im Rahmen der ISO (International Standard Organisation) werden präsentiert.

Normung ist eine „Querschnittsaufgabe“, an der interessierte Kreise aus Forschung, Öffentlicher Hand, Industrie, Handel, Handwerk und Verbraucherverbänden mitwirken, mit dem DIN in der Rolle des Projektmanagers. Wesentliches Ziel der Normungsarbeit im nationalen und internationalen Rahmen ist die Unterstützung eines freien Warenverkehrs auf der Basis eines einheitlichen und widerspruchsfreien länderübergreifenden Normenwerks.

6. Bedürfnisse und Anforderungen an Biotests aus regulatorischer Sicht

Schindler Yael, BAFU
yael.schindler@bafu.admin.ch

Das Schweizer Gewässerschutzgesetz geht von einem umfassenden Schutzgedanken aus. Gewässer sollen vor allen negativen Einwirkungen geschützt werden, damit sie ihre vielfältigen Funktionen als Lebensräume für Pflanzen und Tiere, aber auch als Nutzungsobjekte erfüllen können. Um die Gewässer umfassend schützen zu können und Massnahmen einzuleiten, muss ihr Zustand genau bekannt sein. Das Modul-Stufen-Konzept (www.modul-stufen-konzept.ch) bildet den Rahmen für eine standardisierte Gewässeruntersuchung und -bewertung. Das Konzept ist aus Teilmethoden aufgebaut und umfasst Untersuchungen der Hydrologie, der Ökomorphologie, der Wasserchemie und der Lebensgemeinschaften von Tieren, Pflanzen und Mikroorganismen.

Die Belastung mit Mikroverunreinigungen wie Chemikalien, Medikamente und Pflanzenschutzmittel stellt die zurzeit grösste Herausforderung im stofflichen Gewässerschutz dar. Mikroverunreinigungen können bereits in sehr geringen Konzentrationen die Lebewesen in den Gewässern schädigen und das Trinkwasser verunreinigen. Um die Gewässerbelastung mit Mikroverunreinigungen zu bewerten, werden meist Einzelstoffe chemisch analysiert und die Messwerte mit effektbasierten Grenzwerten, sogenannten Qualitätskriterien, verglichen. Dieses Vorgehen soll mit der revidierten Gewässerschutzverordnung bald auch in der Schweiz eine gesetzliche Grundlage erhalten. Der Vergleich mit Qualitätskriterien ist jedoch auf bekannte Stoffe limitiert, zu denen ausreichend gute Messwerte vorliegen. Will man die Wasserqualität über solche Einzelstoffe hinaus beurteilen, sind zusätzlich integrative Methoden nötig, wie zum Beispiel Biotests. Im Rahmen des Modul-Stufen-Konzept wurde deshalb auch an der Entwicklung von Beurteilungsmethoden für den Gewässerzustand gearbeitet, welche auf Biotests basieren. Für eine erste Grobbeurteilung der Gewässerqualität von abwasserbelasteten Gewässern wurden der kombinierte Algentest und des Yeast Estrogen Sceen (YES) ausgewählt um einerseits die Photosynthese II-hemmende und andererseits die östrogen-aktive Stoffe im Gewässer zu messen.

Für eine routinemässige Untersuchung von Fliessgewässern anhand von Biotests müssen diese möglichst sensitiv, robust, kostengünstig und einfach routinemässig anwendbar und standardisiert sein. Eine nationale und internationale Standardisierung steigert also die Möglichkeiten, Biotestverfahren in die Regulatorik einzubinden und anzuwenden.

7. Turmbau zu Babel

Buchinger Sebastian, BfG
Georg Reifferscheid Etienne Vermeirssen

buchinger@bafg.de

Warum betreiben wir Forschung? Dass wir nach Goethes Faust erkennen „was die Welt im Innersten zusammenhält“? Weil wir den Stein der Weisen finden wollen? Weil es spannend ist? – Sicher gibt es eine Reihe von Gründen sich im Labor dem auch manchmal frustrierenden Geschäft der Forschung zu widmen. Gerade im Bereich der Umweltwissenschaften spielt aber sicherlich die Motivation den ’Zustand der Umwelt durch neue Erkenntnisse konkret zu verbessern, eine große Rolle. Doch wie kann das erreicht werden?

Fragen des Umweltschutzes sind immer auch sehr politische Fragen – etwas plakativ: Umweltschutz vs. Wirtschaftswachstum, Regulierung vs. Freiheit. Wissenschaftliche Erkenntnisse sind ein wichtiger Baustein bei der – politischen – Entscheidungsfindung. Dazu müssen sie belastbar, verständlich und anwendbar sein. Dies gilt in besonderem Maße für Methoden zur Erfassung von Einflüssen auf Mensch und Umwelt, die durch Stoffe in der Umwelt hervorgerufen werden können.

Leider ist es aber oftmals so wie beim Turmbau zu Babel nach der Sprachverwirrung, dass eine Vielzahl von Ansätzen und Methoden – meist auch parallel zueinander – entwickelt werden. Dies ist einerseits von Vorteil, weil verschiedene Methoden miteinander konkurrieren können – andererseits ist es im Hinblick auf regulatorische Umsetzbarkeit zwingend erforderlich, sich auf ein oder wenige Verfahren zu einigen, auf dessen Grundlage sicheres Handeln möglich ist. Diese „Einigung“ spiegelt sich im Normierungsverfahren einer Methode wider.

In zwei Vorträgen soll anhand von einigen Beispielen aus der Gegenwart und der jüngeren Vergangenheit - z.B. zu Verfahren für die Erfassung gentoxischer oder hormoneller Effekte - Interesse geweckt werden, den oft als trocken wahrgenommenen Weg von der Forschung zur Regulatorik auf sich zu nehmen, damit verlässliche Werkzeuge zur Verbesserung der Umweltqualität auch zukünftig zur Verfügung stehen.

8. Turmbau zu Babel

Etienne Vermeirssen, Oektoxzentrum
Buchinger Sebastian, Georg Reifferscheid

Etienne.vermeirssen@oekotoxzentrum.ch

Warum betreiben wir Forschung? Dass wir nach Goethes Faust erkennen „was die Welt im Innersten zusammenhält“? Weil wir den Stein der Weisen finden wollen? Weil es spannend ist? – Sicher gibt es eine Reihe von Gründen sich im Labor dem auch manchmal frustrierenden Geschäft der Forschung zu widmen. Gerade im Bereich der Umweltwissenschaften spielt aber sicherlich die Motivation den ’Zustand der Umwelt durch neue Erkenntnisse konkret zu verbessern, eine große Rolle. Doch wie kann das erreicht werden?

Fragen des Umweltschutzes sind immer auch sehr politische Fragen – etwas plakativ: Umweltschutz vs. Wirtschaftswachstum, Regulierung vs. Freiheit. Wissenschaftliche Erkenntnisse sind ein wichtiger Baustein bei der – politischen – Entscheidungsfindung. Dazu müssen sie belastbar, verständlich und anwendbar sein. Dies gilt in besonderem Maße für Methoden zur Erfassung von Einflüssen auf Mensch und Umwelt, die durch Stoffe in der Umwelt hervorgerufen werden können.

Leider ist es aber oftmals so wie beim Turmbau zu Babel nach der Sprachverwirrung, dass eine Vielzahl von Ansätzen und Methoden – meist auch parallel zueinander – entwickelt werden. Dies ist einerseits von Vorteil, weil verschiedene Methoden miteinander konkurrieren können – andererseits ist es im Hinblick auf regulatorische Umsetzbarkeit zwingend erforderlich, sich auf ein oder wenige Verfahren zu einigen, auf dessen Grundlage sicheres Handeln möglich ist. Diese „Einigung“ spiegelt sich im Normierungsverfahren einer Methode wider.

In zwei Vorträgen soll anhand von einigen Beispielen aus der Gegenwart und der jüngeren Vergangenheit - z.B. zu Verfahren für die Erfassung gentoxischer oder hormoneller Effekte - Interesse geweckt werden, den oft als trocken wahrgenommenen Weg von der Forschung zur Regulatorik auf sich zu nehmen, damit verlässliche Werkzeuge zur Verbesserung der Umweltqualität auch zukünftig zur Verfügung stehen.

Sedimentökotoxikologie

9. Ergebnisse des Projektes DioRAMA – Erfassung Dioxin-ähnlicher Wirkung in Sediment und Biota zur Sedimentbewertung

Brinkmann Markus, RWTH Aachen University
Kathrin Eichbaum, Sebastian Buchinger, Georg Reifferscheid, Henner Hollert

markus.brinkmann@bio5.rwth-aachen.de

Primäres Ziel des Projektes DioRAMA (Erfassung Dioxin-ähnlicher Wirkung in Sediment und Biota zur Sedimentbewertung) war eine Verbesserung der Wissensbasis für die Risikobewertung Sediment-gebundener Dioxin-ähnlicher Schadstoffe (DLC). Dabei wurde ein besonderer Fokus auf polychlorierte Dibenzo-p-dioxine und –furane (PCDD/F), Dioxin-ähnliche polychlorierte Biphenyle (dl-PCB), sowie polyzyklische aromatische Kohlenwasserstoffe (PAK) gelegt. Das Projekt gliederte sich hierzu in zwei separate Teilprojekte. Zum einen sollten (1) Zellkultur-basierte in vitro Biotests etabliert und validiert werden, die eine Quantifizierung von DLC in Sediment und Biota erlauben und damit eine Alternative zur chemischen Analytik mit potentiell höherem Probendurchsatz darstellen. Zum anderen sollte (2) mit Hilfe von experimentellen Untersuchungen und Modellierungsarbeiten untersucht werden, ob DLC während der Resuspension kontaminierter Sedimente für eine Aufnahme in Fische verfügbar sind und dort Schadeffekte verursachen können. Darüber hinaus konnte mithilfe von physiologisch basierten toxikokinetischen (PBTK) Modellen gezeigt werden, dass – im Gegensatz zur weit verbreiteten Meinung – die Ergebnisse von in vitro Biotests durchaus prädiktiv für Effekte in vivo sein können. Die Ergebnisse des Projektes ermöglichen somit eine Gesamtbetrachtung der Ursache-Wirkungskette Dioxin-ähnlicher Effekte beginnend beim Sediment über Biota (Gewebekonzentration), bis hin zu den damit verbundenen Schadeffekten im Fisch. Die im DioRAMA-Projekt erarbeiten und etablierten Werkzeuge für die Risikobewertung kontaminierter Sedimente können in einem nächsten Schritt zur Priorisierung belasteter Sedimente im Rahmen eines Sedimentmanagements eingesetzt, und sollen im Rahmen des Vortrages vorgestellt werden.

10. Development and application of Sediment Quality Guidelines (SQG) for quality assessment of fine freshwater sediments

Brinke Marvin, Bundesanstalt für Gewässerkunde (BfG)

Sebastian Höss, Evelyn Claus, Christel Möhlenkamp, Georg Reifferscheid, Walter Traunspurger, Peter Heininger

brinke@bafg.de

Sediment management needs tools for quality assessment that help to prioritize management or further assessment options. Especially the quality assessment of fine sediments is an important issue because they are a major binding phase for many pollutants inside waterbodies. Besides being able to release pollutants to the water phase again, fine sediments are directly impacting benthic organisms living between the fine particles. These benthic organisms comprise an abundant and diverse community that fulfils important functions in the aquatic ecosystem, such as regarding the nutrient cycle. Moreover, this community is mainly dominated by meiobenthic organisms (e.g., nematodes, tardigrades, ostracods, gastrotrichs, rotifers) and not by macroinvertebrates. Hence, tools based on nematodes have been recently developed to particularly assess the quality of fine sediments: a) nematode-based Sediment Quality Guidelines (SQG) for estimating the probability of toxic effects based on measured chemical concentrations and b) the NemaSPEAR-Index (Nematode SPEcies At Risk) that indicates pollution-induced nematode community alterations (Höss et al., 2011, Environ Int 37: 940-949). The presentation gives information about the derivation of the SQG, including the evaluation of their predictive ability. A major application of the SQG will likely be their use as a screening tool, but they can also be used for a chemical line of evidence in a weight-of-evidence framework. An example for the latter, using the NemaSPEAR for an ecological line of evidence, will be also given in the presentation. Nematode-based lines of evidence will likely facilitate the assessment of fine sediments and thus, support decision making processes of regulators. Moreover, both tools are generally also aiming at closing the knowledge gap regarding effects of pollutants on meiobenthic organisms, which are of high ecological relevance not only in fine freshwater sediments but also in many other habitats (e.g., periphyton, marine sediments, and soils).

11. Entwicklung und Anwendung von Sedimentkontakttests zur Untersuchung des bioverfügbaren Schädigungspotentials in Zebrabärblings- (Danio rerio) Embryonen

Schiwy Sabrina, Institut für Umweltforschung- RWTH-Aachen

Jennifer Bräunig, Yvonne Müller, Henriette Meyer-Alert, Henner Hollert, Steffen Keiter

s.schiwy@bio5.rwth-aachen.de

Die Europäische Wasserrahmenrichtlinie schreibt als zu erreichendes Qualitätsziel den guten ökologischen Zustand von Gewässern vor. Jedoch bestehen noch Lücken im Wissen darüber wie dieses Ziel umgesetzt werden kann. Die Sedimenttoxikologie spielt dabei eine große Rolle, da Sedimente als Schadstoffsenke und -quelle für eine Vielzahl sekundärer Verunreinigungen verantwortlich sind. Auf Basis früherer Untersuchungen wurden drei Probenstandorte ausgewählt - zwei Rheinproben (Ehrenbreitstein und Altrip) und einer aus dem Veringkanal in Hamburg. Diese Studie ist Teil des Verbundprojekts DanTox, welches, neben anderen spezifischen Endpunkten, das embryotoxische und AhR-vermittelte Potential, sowie Veränderungen im Genexpressionsmuster, ausgewählter Sedimente untersucht.

Zebrafischembryonen und Larven spielen in ökotoxikologischen Untersuchungen eine immer bedeutendere Rolle. Gerade deshalb ist es zwingend nötig mehr Wissen über den Fremdstoffmetabolismus der Zebrafischembryonen und Larven zu erfahren. Die Cytochrom P450 1 (CYP1) Gen-Familie beinhaltet in Zebrafischen vier Unterfamilien: cyp1a, cyp1b1, cyp1c1, cyp1c2 und cyp1d1. Bis auf cyp1d1 sind alle am Fremdstoffmetabolismus beteiligt und werden über den A-rylhydrocarbon Rezeptor (AhR) vermittelt. Deshalb wurden im speziellen Testsysteme entwickelt bzw. optimiert und etabliert mit denen die Aktivität und Expression der CYP1 Familie in frühen embryonalen Stadien des Zebrafisches gemessen werden können, um das bioverfügbare Schädigungspotential von Sedimenten bestimmen zu können.

Die Probe vom Veringkanal zeigte durch alle angewendeten Testsysteme das stärkste toxische Potential gefolgt von Altrip und Ehrenbreitstein. Die Ergebnisse aus der chemischen Analyse unterstützen das entsprechend unterschiedliche toxische Potential der Sedimentproben. Die Sedimentexposition führte zu einer Vielzahl an Malformationen in Zebrafischembryonen. Die LC50-Werte schwankten von 1,2 bis 25,8 mg SEQ/ml. Außerdem zeigten alle drei Sedimente eine Steigerung der Toxizität über die Zeit (24, 48, 72 und 96 hpf). Signifikante EROD Aktivität konnte ebenfalls in allen Sedimenten gemessen werden. Alle Transkripte der untersuchten Gene (cyp1a, cyp1b1, cyp1c1, cyp1c2) wurden durch den potenten AhR-Agonisten ß Naphthoflavon und die Sedimente hochreguliert und wiesen ein deutliches Expressionsmuster auf.

Im Sinne der Wasserrahmenrichtlinie ist es von besonderem Interesse Wirbeltier-basierte Testverfahren mit unterschiedlichen biologische Endpunkten, sowie Genexpressions¬analysen zu entwickeln, um das bioverfügbare Schädigungspotenzial von Sedimenten zu prüfen. Die Ergebnisse dieser Studie sind ein erster Schritt zur Etablierung solcher ökotoxikologischer Testverfahren.

Danksagung – Die Autoren danken dem Bundesministerium für Bildung und Forschung für die Finanzierung des Projektes.

Aquatische Ökotoxikologie

12. Der Embryo der Dickkopf-Elritze als Alternative für die Bewertung von Toxizität und Teratogenität

Böhler Svenja, Universität Heidelberg
Sophia Oberrauch, Susanna Mieck, Franziska Beetgen, Lukas Scholz, Thomas Braunbeck

Svenja.Boehler@cos.uni-heidelberg.de

Die Dickkopfelritze (Pimephales promelas) ist zusammen mit dem Zebrabärbling (Danio rerio) und dem Medaka (Oryzias latipes) einer der drei am häufigsten genutzten Testfischarten der OECD. Trotz der extensiven Nutzung in Toxizitätstests ist jedoch verhältnismäßig wenig über die Entwicklung der Dickkopfelritze bekannt. Nach Annahme des Fischembryotests (FET) mit dem Zebrabärbling als OECD Test-Guideline 236 ist es nun notwendig bzw. wünschenswert, das Protokoll auf weitere OECD Fischarten auszuweiten um die Anwendungsmöglichkeiten des Tests auszuweiten. Das adaptierte FET-Protokoll für die Dickkopfelritze wurde mit sechs Substanzen validiert: Isoniazid, Ethanol, Koffein, Natriumvalproat, Hydroxyharnstoff und Tretinoin. Alle Substanzen außer Isoniazid sind für ihr teratogenes Potential im Säuger bekannt. Zusätzlich zur akuten Toxizität wurden über zwanzig strukturelle Missbildungen und physiologische Dysfunktionen als teratogene Endpunkte herangezogen. Da bekannt ist, dass einige der getesteten Substanzen strukturelle Veränderungen im Bereich des knorpeligen und knöchernen Kopfskeletts verursachen, wurden die Embryonen nach Abschluss der Belastung spezifischen Färbungen unterzogen und mit normal entwickelten Embryonen verglichen.

13. Alcohol, coffee and cigarettes: interlaboratory evaluation of candidate positive control chemicals for behavioural assessment in zebrafish larvae

Di Paolo Carolina, RWTH Aachen University

Katharina Graf 1, Jessica Legradi 2, Martina Fenske 3, Richard Ottermanns 1, Jonas Hausen 1, Juliette Legler 2, Inge Werner 4, Thomas-Benjamin Seiler 1, Henner Hollert 1 1:Institute for Environmental Research, RWTH Aachen University, Aachen, Germany; 2: Institute for Environmental Studies, VU University, Amsterdam, the Netherlands; 3: Fraunhofer Institute for Molecular Biology and Applied Ecology IME, Aachen; 4: Swiss Centre for Applied Ecotoxicology Eawag-EPFL, Dübendorf, Switzerland

carolina.dipaolo@bio5.rwth-aachen.de

The impact of environmental contaminants on the behaviour of aquatic organisms is of increasing concern. Zebrafish, a well-established model species in toxicology, is also an emerging model in behavioural sciences. Behavioural alterations in zebrafish can indicate organism-level responses to compounds with different modes-of-action, in correlation with chemical classes. However, the procedures applied to evaluate behavioural changes in zebrafish early life stages (ELS) are not clearly defined and vary between laboratories and studies. That makes the interpretation and validation of results challenging. The standardization of experimental protocols and data analysis would promote the assessment of behavioural endpoints in zebrafish ELS.

This study aimed to: (i) investigate candidate positive control chemicals for short light-dark transition behavioural testing with 5dpf zebrafish larvae; and (ii) evaluate if experiments conducted in two laboratories would produce comparable results. The same set of experiments was performed at the VU University and at RWTH Aachen University. At VU, the ViewPoint automated video tracking system was used; while at RWTH a custom-made system combined with EthoVision (Noldus) was applied. Zebrafish larvae were exposed for 10, 40 or 90min to several concentrations of ethanol, caffeine and nicotine, or control conditions. Larvae were submitted to short light-dark transitions, and video tracking of locomotion was performed. Data was analysed for parameters as total distance moved and ratios between dark and light activities. Stimulated or suppressed behavioural profiles were evaluated concerning test concentrations and exposure periods.

Basal locomotion activity in control conditions differed slightly between laboratories, which can be related to the use of different fish strains. Still, patterns of behavioural responses presented intra and inter-laboratory reproducibility for the same compounds, concentrations and exposure periods. Particularly, ethanol caused highly reproducible behavioural effects. Our study indicates that results of the two laboratories were consistent for the assessment of behavioural endpoints in zebrafish larvae exposed to the test compounds. Further investigations of neuroactive and neurotoxic chemicals are needed for the establishment and interpretation of new behavioural endpoints and standardized test protocols with zebrafish ELS. As a result, the applicability of zebrafish behavioural tests for the evaluation of complex environmental samples will be facilitated.

14. Listen to the clams: Introducing mussel filtration behaviour as a biomarker for environmental pollution

Hartmann Jason, Technische Universität München

S. Beggel, K. Auerswald, B. Stoeckle, J. Geist

jason.hartmann@tum.de

Most freshwater mussel species of the Unionoida are endangered, presenting a conservation issue as they are keystone species providing essential services for aquatic ecosystems. As filter feeders with limited mobility, mussels are highly susceptible to water pollution. Despite their exposure risk, mussels are underrepresented in standard ecotoxicological methods. Our study aimed to demonstrate that mussel behavioral response to a chemical stressor is a suitable biomarker for the advancement of ecotoxicology methods that aids mussel conservation. Modern software and Hall sensor technology enabled mussel filtration behavior to be monitored real-time at very high resolution. With this technology, we present our method using Anodonta anatina and record their response to de-icing salt pollution. The experiment involved an environmentally relevant 'pulse-exposure' design simulating three subsequent inflow events. Three sublethal endpoints were investigated, Filtration Activity, Transition Frequency (number of changes from opened to closed, or vice versa) and Avoidance Behavior. The mussels presented a high variation in filtration behavior, behaving asynchronously. At environmentally relevant de-icing salt exposure scenarios, A. anatina behavior patterns were significantly affected. Treated mussels' Filtration Activity decreased during periods of very high and long de-icing salt exposure ($p < 0.001$), however, increased during short de-icing salt exposure. Treated mussels' Transition Frequency increased during periods of very high and long de-icing salt exposure ($p < 0.001$), which mirrored the Avoidance Behavior endpoint observed only by mussels under chemical stress. Characteristics of Avoidance Behavior were tighter shell closures with repeated and irregular shell movements which was significantly different to their undisturbed resting behavior ($p < 0.001$). Additionally, we found that mussels were sensitive to a chemical stressor even when the mussel's valves were closed. Our experimental method demonstrated that, with the application of current technologies, mussel behavioral response to a chemical stressor can be measured. The tested sublethal endpoints are suitable for mussel ecotoxicology studies. Avoidance Behavior proved to be a potentially suitable endpoint for mussel behavior effect concentration calculations. Therefore we recommend adult mussel behavior as a suitable biomarker for future ecotoxicological research.

15. AquaHab®, a closed aquatic multispecies system for ecotoxicological purposes - past, present and future activities

Dünne Matthias, OHB System AG
matthias.duenne@ohb.de

AquaHab® is a small closed aquatic multispecies system on laboratory level, containing different trophic levels - represented by Oryzias latipes (medaka fish), Hyalella azteca (mexican amphipod), Biomphalaria glabrata (snail of Planorbidae family), Ceratophyllum demersum (hornweed) as well as by nitrifying and denitrifying bacteria. They serve for a comprehensive exposure as well as effect assessment of chemicals. Effect endpoints from subcellular up to system level can be measured. A technical unit controls the test conditions in the 8,2 L-water tank (temperature, oxygen concentration, illumination). The closure of the system enables the assessment of the respiratory and photosynthetic activity as well as high reproducible test results. AquaHab® represents a bridge between single species tests and outdoor ponds concerning the relevance of the results for the environment, the effort to be done as well as the reproducibility of the test results.

In its present form, AquaHab® is highly suited for the risk assessment of chemicals during their development process. It is available for research projects as well as for industrial purposes. E.g., currently the long-term effect and the bioaccumulation of nanoparticles is assessed in AquaHab® in the framework of the BMBF funded project „DENANA“ (Designkriterien für nachhaltige Nanomaterialien). However, AquaHab® might be used also for further activities, e. g. to „biomonitor“ effluents from chemical parks or to investigate the risk of microplastic in aquatic systems.

An overview about past, present and future activities of this versatile tool for ecotoxicological purposes will be given at the symposium.

16. Effect-Assessment of wastewater effluents by molecular biomarkers in brown trout (Salmo trutta)

Fischer Stephan, Eawag

Ksenia Groh, Inge Werner and Kristin Schirmer

stephan.fischer@eawag.ch

Most of the tests developed for detection of chemical effects in organisms exposed in the environment are applicable to only a few model species. mRNA expression analysis of selected biomarker genes is a promising approach for field monitoring of non-model organisms because it can capture a wide spectrum of responses of organisms to chemical exposure. We established a biomarker gene set for brown trout (Salmo trutta) to assess the effects of micropollutants released by wastewater effluents. The biomarker set consisted of 20 genes which reflect different cellular stress responses like general stress, oxidative stress or biotransformation. The transcriptional regulation of these genes was measured in liver and kidney tissue samples of wild brown trout caught downstream and upstream of a wastewater treatment plant (WWTP) and from a reference river. Results from two seasonally different sampling campaigns showed that mRNA levels are site dependent. Fish taken downstream generally express a different transcriptional regulation pattern compared to fish from the upstream and a reference site, indicating that fish downstream are exposed to a higher level of environmental stressors. For example, the metal (Metallothionein B), endocrine disruption (Vitellogenin) or biotransformation (Cyp3a) sensitive genes were found to be significantly upregulated in fish from the downstream location compared to the other two sites. Results implicated a higher content of metals, potential estrogen-mimics or different pharmaceuticals downstream of the WWTP. Indeed, chemical analysis confirmed high concentrations of such compounds in the water from this site compared to the others. Our data demonstrates that quantifying expression levels of selected biomarker genes allows an assessment of exposure and related effects of chemicals. It appears that our method is a promising screening assay for assessing surface water quality. Moreover, based on the selected set of genes, predicting potential adverse effects may be possible.

17. Immunomodulatory activity of thyroid-active compounds in rainbow trout (Oncorhynchus mykiss)

Quesada-Garcia Alba, Instituto Nacional Investigaciones Agrarias y Alimentarias INIA, Madrid

Paloma Encinas Ana Valdehita Julio M Coll . Navas Instituto Nacional Investigaciones Agrarias y Alimentarias INIA, Madrid Lisa Baumann Helmut Segner Centre for Fish and Wildlife Health, University of Bern José M. Navas Instituto Nacional Investigaciones Agrarias y Alimentarias INIA, Madrid

helmut.segner@vetsuisse.unibe.ch

In mammals, an immunomodulating effect of thyroid hormones (TH) and thyroid disruptors is well documented. In fish, research on thyroid disruptors has been focused largely on adverse impacts on the development and functioning of the thyroid gland, whereas peripheral effects including the alteration of immunocapacity have been rarely studied. Here, we examine potential immunomodulating effects of thyroid-active compounds in rainbow trout. In an initial study, we demonstrated that immune cells of trout possess thyroid hormone receptors (THR) and thus are potentially responsive to THR agonists and antagonists. In addition, treatment of trout with either the active hormone, 5-triiodo-L-thyronine (T3), or the antagonist, 6-propyl-2-thiouracil (PTU), altered THR expression levels in the immune cells. These observations point to a possible impact of thyroid-.active compounds on the immune function of trout. To shed more light on this question, we used a custom-designed 60-mer oligo immune-targeted microarray to analyze the gene expression profiles induced by T3 or PTU in the head kidney of rainbow trout. Morphometric analyses of rainbow trout thyroid tissue revealed that PTU exposure increased the size of the epithelial cells, whereas T3 treatment induced no significant change. T3 and PTU had numerous differential effects on immune gene transcript profiles. The most prominent T3/PTU-induced differential gene expressions were observed for the Mitogen Associated Protein Kinases (MAPK), NFkB, Natural Killer (NK) and Toll-Like Receptor (TLR) pathways, including multipath genes (MPG) such as pleiotropic transcription factors (atf1, junb, myc), important pro-inflammatory genes (tnfa, tnf6, il1b) and interferon-related genes (ifng, irf10). From these findings, genes were identified which may serve as novel markers for T3/PTU exposure and/or to detect otherwise missed potentially immunodisrupting effects of endocrine-active environmental compounds. In conclusion, the findings from this study promote our understanding of immunmodulating actions of T3 and PTU on fish.

Acknowledgement: This study is financially supported by INIA and CICYT projects AGL2014-51773-C3-3-R, AGL2014-53190RED of the Ministerio of Economia y Competitividad, Soain.

18. Effects of methylmercury on aquatic primary producers: a transcriptomic approach

Beauvais-Flueck Rebecca, University of Geneva
V. I. Slaveykova, C. Cosio

rebecca.flueck@unige.ch

Mercury (Hg) is amongst the most toxic metals with no known biological function. Its properties: volatile, long-range transportable and biomagnified in the aquatic trophic chain have led Hg to become a global environmental issue, irrespective of the presence of local emission sources. Aquatic primary producers, such as phytoplankton and macrophytes, are key players of the biogeochemical cycle of mercury (Hg) in the aquatic environment. Indeed, the Hg bioaccumulation step from water to primary producers has been shown to be the largest increase of Hg concentrations in aquatic ecosystems reaching 104-fold and greater. It is therefore of the upmost importance to understand uptake and effects of Hg in primary producers. The effects of Hg on aquatic primary producers were only sparsely investigated until now. While it has been shown that Hg affects photosynthesis and the activity of oxidative stress enzymes in microalgae, its effects were less studied in macrophytes, especially concerning MeHg. Moreover most studies were conducted at high concentrations only and were thus not representative of environmental conditions. Using a transcriptomic approach (RNA-Seq), we therefore studied the effects at the molecular level of MeHg (5 x10^{-11}, 5 x10^{-10}, 5 x10^{-9} and 5 x10^{-8} M), in the microalga Chlamydomonas reinhardtii and the macrophyte Elodea nuttallii, adapting bioinformatics analyses to the available genomic information for the studied species. Strong response of the transcripts involved in photosynthesis was observed at all MeHg concentrations in the alga, but only at the two highest concentrations in the macrophyte. Concomitantly, MeHg affected genes involved in carbohydrates, amino acids and lipids metabolisms, as well as the expression of many transcripts coding for transporters. In fact, while ABC and calcium transporters were significantly affected by MeHg in C. reinhardtii, ATPases and ammonium transport were disregulated in E. nuttallii. Finally, metal transporters, e.g. zinc transporters, were significantly affected in both species. The transcriptome response to MeHg in the two representative aquatic primary producers was congruent with their morphological, physiological and sensitivity differences.

19. Characterization of transcriptional responses to dioxins and dioxin-like contaminants in roach (Rutilus rutilus) using whole transcriptome analysis

Koglin Sven, RWTH Aachen University

Markus Brinkmann (ebenfalls Erstautor) • Bryanna Eisner • Steve Wiseman • Markus Hecker • Kathrin Eichbaum • Beat Thalmann • Sebastian Buchinger • Georg Reifferscheid • Henner Hollert

svenkoglin@gmx.de

Riverine sediments can be contaminated with a complex mixture of lipophilic organic pollutants. Of particular concern in this context are dioxins and dioxin-like compounds (DLCs), including polychlorinated dibenzo-p-dioxins (PCDDs), polychlorinated dibenzofurans (PCDFs), polychlorinated biphenyls (PCBs) and some polycyclic aromatic hydrocarbons (PAHs). While many researchers have focused on investigating the molecular and biochemical effects of DLCs in a few standard model fish species, there is significant uncertainty with regard of the representativity of these model species for fishes native to riverine systems in Europe. In this study, the transcriptional changes in juvenile roach (Rutilus rutilus), a fish species that constitutes a large proportion of the fish biomass in bodies of freshwater throughout Europe, following treatment with equipotent concentrations of TCDD, PCB 156 or the dioxin-like PAH benzo[k]fluoranthene (BkF) were investigated. To this end, Illumina RNAseq was used to gain a comprehensive understanding of the potential molecular mechanisms and pathways of toxicity of different DLCs to this non-model species. Whole transcriptome analysis by use of ClueGO software revealed that DLCs have the potential to disrupt a number of important physiological processes, including energy metabolism, oogenesis, the immune system, apoptosis and the response to oxidative stress. However, despite use of equipotent concentrations of DLCs, there was only little conservation of transcriptional responses to each compound. TCDD provoked significant changes to abundances of transcripts of genes related to immunotoxicity and carbohydrate metabolism whereas PCB 156 caused no specific effects compared with the other two compounds. The most diverse suite of differentially expressed transcripts was caused by exposure to BkF, and included alterations to abundances of transcripts of genes that regulate the cardiovascular system and the response to oxidative stress. To our knowledge, this is the first study describing transcriptome-wide effects for different classes of DLCs in fish. These findings represent an important first step in describing complete toxicity pathways (adverse outcome pathways) for the exposure of fish to DLCs, which will be important in context with informing risk assessments of native fish species with regard to DLC toxicity.

20. The NORMAN interlaboratory study on biotesting of spiked water extracts

Di Paolo Carolina, RWTH Aachen University

Richard Ottermanns 1, Steffen Keiter 1,2, Selim Ait-Aissa 3, Werner Brack 4, Magnus Breitholtz 5, Sebastian Buchinger 6, Mario Carere 7, Carole Chalon 8, Xavier Cousin 9, Valeria Dulio 3, Beate Escher 4,10, Timo Hamers 11, Klára Hilscherová 12, Sergio Jarque 12, Adam Jonas 12, Robert Kase 13, Emmanuelle Maillot-Marechal 3, Yves Marneffe 8, Pascal Pandard 3, Andrea Schifferli 13, Tobias Schulze 4, Sven Seidensticker 1, Thomas-Benjamin Seiler 1, Janet Tang 10, Mai Thao Nguyen 14, Ron van der Oost 15, Etienne Vermeirssen 13, Radka Zounková 12, Nick Zwart 11, Henner Hollert 1 1: Institute for Environmental Research, RWTH Aachen University, Aachen, Germany; 2: Man-Technology-Environment Research Centre, School of Science and Technology, Örebro University, Örebro, Sweden; 3: INERIS, Verneuil-en-Halatte, France; 4: UFZ-Helmholtz Centre for Environmental Research, Leipzig, Germany; 5: Department of Applied Environmental Science - ITM, Stockholm University, Stockholm, Sweden; 6: Department Biochemistry and Eco-toxicology, Federal Institute of Hydrology, Koblenz, Germany; 7: Italian Institute of Health, Italy; 8: ISSeP (Scientific Institute of Public Service), Wallonia, Belgium; 9: Laboratoire d'Ecotoxicologie, Ifremer, L'Houmeau, France; 10: National Research Centre for Environmental Toxicology - Entox, the University of Queensland, Brisbane, Australia; 11: Institute for Environmental Studies -IVM, VU University Amsterdam, the Netherlands; 12: Research Centre for Toxic Compounds in the Environment - RECETOX, Faculty of Science, Masaryk University, Brno, Czech Republic; 13: Swiss Centre for Applied Ecotoxicology Eawag-EPFL, Dübendorf, Switzerland; 14: Waterproef Laboratory, Edam, the Netherlands 15: Waternet, Institute for the Urban Water Cycle, Division of Technology Research & Engineering, Amsterdam, the Netherlands

carolina.dipaolo@bio5.rwth-aachen.de

The NORMAN network is a permanent self-sustaining network for the monitoring and bio-monitoring of emerging environmental contaminants. The NORMAN working group on Bioassays (Bio WG) focuses on the application of bioanalytical tools for environmental quality monitoring. A main objective is to provide recommendations for the implementation of effect-based tools into regulatory frameworks. In this context, a blind interlaboratory study (ILS) was performed. The aim was to verify if a bioassay battery conducted in different laboratories following own protocols would produce comparable results when testing spiked surface water extracts. The lead in planning and organizing was taken by the Department of Ecosystem Analysis (ESA), RWTH Aachen University (DE). The ILS bioassay battery included acute-toxicity assays with organisms from different trophic levels (Algae, Daphnia, Zebrafish embryos); and mechanism-specific bioassays for estrogenicity (YES, ER-Luc cell lines) and mutagenicity (Ames fluctuation) assessment. Three to four participants performed each bioassay, including: BfG (DE), Waternet (NL), Waterproef (NL), INERIS (FR), IFREMER (FR), RECETOX (CZ), ISSeP (BE), ITM (SE), IVM-VU (NL), Entox/University of Queensland (AU), Ecotox Centre (CH), ESA-RWTH (DE). Pristine water from a reference site was concentrated 10.000 times with large volume solid-phase extraction. Four emerging pollutants were used for spiking: triclosan, acridine, 3-nitrobezanthrone and 17-alpha-ethinylestradiol. Extracts were spiked with either single chemicals or a mixture, in concentrations aimed to produce full dose-response curves in bioassays. The spiked extracts were prepared, separated in aliquots, identified with codes, and sent to the participating laboratories. Standardized bioassay methods (OECD, ISO) were recommended but not mandatory, and biotesters

could use their own protocols. Results were sent to RWTH, and a summary of the full ILS was provided to the ILS participants. In October 2014, a workshop was held at RWTH Aachen to present and discuss the ILS results. Bioassays produced mostly highly comparable results, even when protocols differed significantly. Suggestions for future improvements include harmonization of methods for data analysis and results evaluation. An important expected outcome of the ILS is the promotion of biotesting for water quality monitoring at the level of European policy-makers.

21. Increased reverse osmosis concentrate toxicity following application of antiscalants - Acute toxicity tests with the amphipods Gammarus pulex and Gammarus roeseli

Feiner Mona, Technische Universität München

Sebastian Beggel (sebastian.beggel@tum.de) Nadine Jäger (Nadine.Jaeger@wvv.de) Jüergen Geist (geist@wzw.tum.de)

mona.feiner@tum.de

In reverse osmosis, a frequently used technology in water desalination processes, wastewater (RO concentrate) is generated containing the retained solutes as well as so-called antiscalants (AS), i.e. chemical substances that are commonly applied to prevent membrane-blocking. In this study, a risk assessment of a possible discharge of concentrate into a small stream was conducted. The acute toxicity of two concentrates containing two different ASs and of concentrate without AS to the amphipods Gammarus pulex and Gammarus roeseli was studied. Mortality of gammarids exposed to the concentrate without AS was not different to the control, whereas concentrates including ASs caused mortality rates up to 100% at the highest test concentrations after 168 h. Resulting EC50-values were 36.2-39.4% (v/v) after 96 h and 26.6-58.0% (v/v) after 168 h. These results suggest that the ecotoxicological relevance of antiscalants is greater than currently assumed.

22. Energy ashes from virgin wood fuel show different potentials for growth promotion and toxicity in Lemna minor (L.)

Jagodzinski Lucas S., School of Biological, Earth and Environmental Sciences (BEES) & Environmental Research Institute (ERI), University College Cork (UCC), IRL

van Pelt, Frank (Department of Pharmacology and Therapeutics & ERI, UCC); O'Halloran, John (BEES & ERI, UCC); Jansen, Marcel (BEES & ERI, UCC)

l.jagodzinski@ucc.ie

Wood ash contains high amounts of plant nutrients such as phosphorus, potassium, calcium as well as several micro nutrients. In this study the plant growth enhancing properties of Sitka spruce (Picea sitchensis) wood ash were contrasted with its toxic action. The growth of common duckweed (Lemna minor) exposed to wood bottom and fly ash solids (as suspensions) and corresponding leachates was assessed in ultra-oligotrophic and eutrophic media. Doses stimulating and inhibiting plant growth under these conditions were determined. For purpose of comparison to standard waste toxicity data, ash solids and leachates were also tested as neutralized preparations. Suspended bottom and fly ash solids improved L. minor growth up to concentrations of 2.5 and 5 g/L respectively. Leachates promoted growth up to 10 g ash equivalents per litre, but for bottom ash only. Beneficial effects of ash were most pronounced on ultra-oligotrophic growth medium. In contrast, no significant growth promoting effect of wood ash supplementation was found in (eutrophic) standard test medium. Severe inhibition (EC50) of common duckweed biomass and frond growth by fly ash was observed in nutrient deficient (12-18 g/L), standard (18-22 g/L) and neutralized standard media (37-61 g/L). In comparison, higher doses of bottom ash were required to incur the same level of growth retardation in ultra-oligotrophic and eutrophic media (35-50 g/L) and in neutralized standard medium (70-95 g/L).

It is argued that growth promotion is due to the substantial content of nutrients but may be conditional upon the presence of ash solids. Phytotoxicity is due to both the elemental composition of the ash, its alkaline character, and possible interactions between these two factors. This study shows that the margin between growth promoting and toxicity inducing concentrations can be enlarged through ash neutralization. This study further underlines the importance of the receiving environment (nutrient status and pH) in determining the balance between toxicity and growth promotion, and thus the impact of ash spreading on plant growth.

Terrestrische Ökotoxikologie

23. Bestimmung der Wirksamkeit von Netzmitteln (Aceton, Tween80, Triton X-100 und Etalfix) und ihr Einfluss auf die akute Toxizität von Dimethoat anhand von Bombus terrestris und Osmia bicornis Kontaktstudien

Eschenbach Eva, Institut für Umweltwissenschaften, Universität Koblenz-Landau
Claudia Wollmann, Philipp Uhl, Peter Stahlschmidt, Lukas Jeker, Carsten A. Brühl

eva.eschenbach@gmx.de

Netzmittel sind Tenside, welche in Kontaktstudien mit Bienen und anderen Insekten benutzt werden. Sie erleichtern das Eindringen aktiver Substanzen in den Insektenkörper durch Reduktion der Oberflächenspannung von Applikationslösungen. Die OECD Ringtest Protokolle (Honigbiene) und Entwürfe für Protokolle (Solitärbienen und Hummeln) für Kontakttoxizität nennen verschiedene Netzmittel für die Applikation bei verschiedenen Bienen, obwohl alle drei Spezies einen Thorax aus Chitin besitzen, da angenommen wird dass sich die Zusammensetzung der Cuticula verschiedener Spezies unterscheidet. Während man für die Honigbiene zwischen fünf verschiedenen Netzmitteln wählen kann (Tween, Citowett, Lubrol, Agral oder Triton), hat man für Solitärbienen die Wahl zwischen Aceton und Triton; für die Hummel wird bisher nur Triton empfohlen. Bisher gibt es sehr wenige Studien zur Toxizität von Netzmitteln und deren kombinierte Toxizität mit Pestiziden während es keine Studien über ihre Wirksamkeit gibt. Eine gute Wirksamkeit ist hier definiert als schnelles Einziehen der zu applizierenden Substanz.

In der vorliegenden Studie soll die Wirksamkeit von Aceton, Tween80, Triton X-100 und Etalfix sowie ihr Effekt auf die Toxizität von Dimethoat (Positivkontrolle in den meisten Akuttests) untersucht werden. Die Netzmittel werden an Bombus terrestris (Arbeiterinnen) und Osmia bicornis getestet und die erhaltenen LD50 Werte untereinander verglichen. Basierend auf den vorhandenen Studien und auf vorläufigen Ergebnissen aus eigenen Labortests mit Osmia bicornis Weibchen (unveröffentlichte Daten) wird deutlich, dass durch den uneinheitlichen Gebrauch von verschiedenen Netzmitteln in verschiedenen Laboren unstimmige Ergebnisse für LD50 und ED50 generiert werden. Wir erwarten, dass durch Aceton und Triton X-100 die Toxizität von Dimethoat erhöht wird und beide eine mittelmäßige bis gute Wirksamkeit zeigen. Von Tween80 und Etalfix wird kein Effekt auf die Toxizität erwartet, jedoch zeigen bisherige Tests eine eher schwache Wirksamkeit von Tween. Für Etalfix wird eine gute Wirksamkeit erwartet. Die Studie wird im Juni durchgeführt, Ergebnisse werden im Juli erwartet.

24. Ergebnisse des Schweizer Workshops „Amphibien und Pflanzenschutzmittel“

Aldrich Annette, Agroscope

Marion Junghans 2, Benedikt Schmidt 3,4
2 Oekotoxzentrum, Eawag-EPFL, 8600 Dübendorf, Schweiz 3 Karch, Passage Maximilien-de-Meuron 6, 2000 Neuchâtel, Schweiz 4 Institut für Evolutionsbiologie und Umweltwissenschaften, Universität Zürich, Winterthurerstrasse 190, 8057 Zürich, Schweiz

annette.aldrich@agroscope.admin.ch

Amphibienpopulationen geraten zur Zeit durch mehrere Stressoren unter Druck. Die International Union for Conservation of Nature (IUCN) hält den Verlust an geeignetem Lebensraum für den wichtigsten Stressor, gefolgt von der Umweltbelastung durch Chemikalien, wie z. B. Pflanzenschutzmittel (PSM). Über den Einfluss von PSM auf Amphibien ist vergleichsweise wenig bekannt, da Amphibien für die Zulassung von PSM nicht explizit getestet werden. In ihrer neuen Datenanforderung (commission regulation (EU) No 283/2013) erkennt die EFSA dies an und fordert explizit eine Beurteilung für Amphibien, auch wenn spezifische Toxizitätsstudien mit Amphibien nicht aufgeführt und generell wenig Leitlinien zu deren Risikobeurteilung zur Verfügung gestellt werden. Aus diesem Grund wurde in der Schweiz von Agroscope, Karch und dem Oekotoxzentrum ein Workshop organisiert, dessen Ziel es war ein transdisziplinäres Netzwerk von Experten aufzubauen, in dem möglichst alle Aspekte des Themenfeldes vertreten sind. Etwa 30 Teilnehmer aus Wissenschaft, Behörden (kantonal, Bund, EU Nachbarländer, EU Kommission), Industrie, Umweltverbänden und Naturschutzfachstellen haben an dem eintägigen Workshop teilgenommen an dem folgende Fragen diskutiert wurden:

- Wie hoch wird das Gefährdungspotential von PSM für Amphibien im Feld eingeschätzt?
- Welche Massnahmen können auf freiwilliger Basis oder aufgrund des Vorsorgeprinzips umgesetzt werden, um den Druck auf die Amphibienpopulationen zu mindern?
- Welche Forschungsfragen sollten angegangen werden, um die Risikobeurteilung für Amphibien sinnvoll weiterentwickeln zu können?

Vor den Diskussionen wurde im Rahmen von Vorträgen noch ein Überblick über den Stand des Wissens in folgenden Bereichen gegeben: Biologie von Amphibien, Gefährdung und Schutz von Amphibien, Wirkung von PSM auf Amphibien, Ökotoxikologische Risikobeurteilung für die Zulassung von PSM, Koinzidenz von Amphibien mit dem Einsatz von PSM und Entwicklung in der EU bezüglich Pestizidrisikobewertung für Amphibien. Darüber hinaus wurde noch das Vorsorgeprinzip und seine Stellung im Schweizer sowie EU Recht juristisch erläutert.

Die Ergebnisse des Workshops werden im Vortrag vorgestellt.

25. Ecotoxicology of Amphibians - Adaption of Dermal In Vitro Test Systems to Amphibian Skin

Kaufmann Katharina, Department for Ecotoxicology, BASF SE, Speyerer Straße 2, 67117 Limburgerhof, Germany

Peter Dohmen

katharina.kaufmann@basf.com

There are indications in literature that terrestrial life stages of amphibians may be more sensitive to certain contaminants than birds or mammals. It is hypothesized that dermal exposure is potentially a significant route of exposure and toxication for amphibians, as there is evidence that their skin is more permeable than the skin of other vertebrate species. Thus, higher amounts of chemicals might enter systemic circulation by dermal uptake. Furthermore, their skin as an organ with essential functions for the organism might be impaired by dermal contact to xenobiotics.

So far, no guidelines exist to investigate dermal exposure and effects of chemicals on amphibians. This work is targeted to develop in vitro test systems as a possible model to assess the dermal uptake and toxicity of test compounds by minimizing vertebrate testing. To this end, established toxicological (mammal) test procedures were adapted to amphibian skin (Xenopus laevis): to address dermal uptake, the dermal absorption in vitro method (OECD TG 428) was tested. This was done by applying reference compounds – caffeine and testosterone – topically to freshly excised skin. Skin permeability to both substances was significantly higher compared to human data. Caffeine permeated faster across the skin than testosterone, with ventral skin tending to be more permeable than dorsal skin. As usage of frozen stored mammal skin is accepted, frozen stored skin of X. laevis was tested in parallel. By freezing (without additional preservation measures), skin permeability to caffeine was increased. To address dermal toxicity, the skin corrosion in vitro method (OECD TG 431) was used based on the MTT test. After topical application of different acetic acid dilutions to freshly excised skin, a dose-dependent reduction of skin viability was detectable.

This work has shown that the chosen methods can be applied successfully to amphibian skin, providing the basis for further investigations. In the future, well established in vitro test systems and a broad dataset of chemicals may help to assess potential amphibian risk from chemicals without the need for extensive vertebrate animal testing.

Bodenökotoxikologie

26. Long-term monitoring of organic pollutants in soil: a conceptual approach to select pesticides potentially accumulating in soil

Wächter Daniel, Swiss Soil Monitoring Network NABO
Thomas Bucheli, Andreas Gubler , Reto G. Meuli, Armin Keller , Peter Schwab, Felix Wettstein

daniel.waechter@agroscope.admin.ch

The Swiss Soil Monitoring Network NABO was set up in the mid-1980s to detect temporal changes of soil quality in the long-term. Initially, soil pollution by heavy metals and acidification were the main issues. In the meantime the analysis of organic soil pollutants improved remarkably. Consequently, soil monitoring networks are able to integrate a larger array of organic soil pollutants using either samples from new sampling campaigns or archived soil samples. Due to the large number of organic chemicals and the limited financial resources, a well-defined conceptual approach is needed to define those to be integrated in soil monitoring. Therefore, we follow a stepwise approach taking into account:

(i) Prediction, (ii) Status survey, (iii) Risk assessment, (iv) Monitoring

This approach might be applied for various groups of chemical compounds. The present study focuses on pesticides. First, selected soil samples from NABO sites were used to establish a multi-residue analytical method (stage i; Krauss & Keller, 2010). Currently, the methodology is refined to analyse a broader range of pesticides; subsequently, it will be applied to agricultural NABO monitoring sites (stage ii). The target substances were selected using the following data bases and approaches:

- Results stage i: pesticides and transformation products detected in first screening.
- Management data of NABO sites: frequency and amount of pesticides used by farmers.
- Modelling soil concentrations: estimation of the temporal evolutions.
- Risk assessment: evaluation considering the estimated concentrations.

The intended analyses will provide an overview of the pesticides exposure of NABO soils. Thereafter, a risk assessment will help to rank the detected pesticide concentrations in soils (stage iii). However, this will involve various issues, e.g. the risk by mixtures of various substances (Wittmer et al. 2014), to finally decide which pesticides are to be monitored (stage iv).

References:

Krauss M & Keller A 2010 Persistence of current-use pesticides and their transformation products in soils under real agricultural practice. SETAC Europe, 20th annual meeting. Seville (E), May 23-27.

Wittmer,I.; Junghans,M.; Singer,H.; Stamm,C., Mikroverunreinigungen - Beurteilungskonzept für organische Spurenstoffe aus diffusen Einträgen. Studie im Auftrag des Bundesamtes für Umwelt (BAFU), 2014, 105 pp, Eawag, Dübendorf.

27. Prioritizing organic contaminants in Swiss soils using environmental partitioning and high resolution mass spectrometry

Aurea C. Chiaia-Hernandez[1], Greta Stieger[2], Martin Scheringer[2,3], Thomas D. Bucheli[4], Armin Keller[5], Daniel Wächter[5] and Juliane Hollender[1,6]

[1] Eawag, Swiss Federal Institute of Aquatic Science and Technology, 8600 Dübendorf
[2] Institute for Chemical and Bioengineering, ETH Zürich, 8093 Zürich, Switzerland
[3] Leuphana University Lüneburg, 21335 Lüneburg, Germany
[4] Agroscope Institute for Sustainability Sciences ISS, 8046 Zürich, Switzerland
[5] NABO, Swiss National Soil Monitoring Network, 8046 Zürich, Switzerland
[6] Department of Environmental Systems Science (D-USYS), ETH Zürich, 8092 Zürich, Zürich, Switzerland

Email contact: aurea.chiaia@eawag.ch

There are many thousands of organic chemicals on the market and it is a challenging task to identify those with a potential long-term environmental contamination. History has shown that chemical-intensive economies continually cause contamination of the environment, that in many cases, remain undetected for years. A point in case is perfluorinated alkyl substances (PFASs), which have been used since the 1970s but were detected in the environment only after 2000. Analytical campaigns and monitoring programs can cover only a certain selection of chemicals. Therefore, strategies for identifying priority chemicals for analytical measurements are needed. In this study we aim to identify relevant chemicals starting from an extensive computer-based screening from the Swiss customs statistics and other databases in Switzerland, followed by a complementary monitoring campaign of representative soils.

The first step in the study was to develop a phase partitioning multimedia environmental fate model with dimensions of Switzerland to identify chemicals with a presence of 90% or more in soil. The results show that 2550 out of 9187 chemicals studied have high affinity to this environmental matrix. As a first approach due to the high number of candidates, a subgroup of only halogenated chemicals (> 500 chemicals) were prioritized to facilitate the analytical analysis. Halogenated substances have a well characteristic isotopic pattern and mass defects outside the soil matrix making their identification easier.

Target and suspect screening was performed in 14 sites from the Swiss National Soil Monitoring Network (NABO) chosen according to land use and vicinity to industrial and urban areas, as well as previously measured soils with persistent organic chemicals such as polychlorinated biphenyls. Soil samples were extracted by means of pressurized liquid extraction and analyzed by liquid chromatography followed by electrospray ionization (ESI) coupled to high resolution mass spectrometry (HRMS/MS). Preliminary results show that the combination of the developed multimedia fate model and HR-MS analysis resulted in the successful identification of different antibacterial substances, fungicides, fluorosurfactants, as well as the tentative identification of further chlorinated, brominated and quaternary ammonium compounds.

28. Wood preservatives in Switzerland: from market analysis to soil organisms ecotoxicity testing

Campiche Sophie, Schweizerisches Zentrum für angewandte Oekotoxikologie Eawag-EPFL

Christelle Ganne-Chédeville(2), Thomas Volkmer(2), Emilie Grand(1), Inge Werner(1), Benoit J.D. Ferrari(1) (1) Swiss Centre for Applied Ecotoxicology Eawag/EPFL, 8600 Dübendorf, Switzerland (2) Bern University of Applied Sciences, Institute for Materials and Wood Technology, 2500 Biel, Switzerland

sophie.campiche@centreecotox.ch

Wood preservatives are biocidal products used to protect wood building materials from biological attacks. For wood used for outdoor applications (Use class 3 or 4), the chemical substances employed are likely to leach into the soil and may then pose a potential risk for the soil organisms and the essential functions they performed. Currently, the available toxicity data for terrestrial organisms regarding the active ingredients present in wood preservatives are still scarce. The aim of this project was to synthesize the available knowledge regarding the toxicity for terrestrial organisms of representative active ingredients contained in wood preservatives and authorized for use in Switzerland, and to provide missing data where necessary. A market analysis was performed to determine the active ingredients the most frequently used and present on the Swiss market. Four wood preservatives containing the selected active ingredients, either present as a single substance or in a mix, were chosen for the ecotoxicological evaluation. They contained: 1) IPBC, 2) Propiconazole, 3) Boric acid and Copper(II)hydroxide, with Chromium as fixing agent; and 4) Copper(II)carbonate-Copper(II)hydroxide, Didecylpolyoxethylammoniumborat (DPAB) and Boric acid. Literature search carried out about the selected active ingredients showed a lack of information regarding the toxicity of IPBC, Propiconazole and DPAB for terrestrial organisms as well as regarding mixture toxicity for the active ingredient combinations. Therefore, collembolan reproduction and earthworm avoidance tests were performed to determine effect concentrations, either for the single active substance or for the active ingredient mixture contained in the wood preservatives. Similar range of toxicity were obtained for collembolans and earthworms for IPBC whereas earthworms showed to be more sensitive than collembolans to Propiconazole. The 2 wood preservatives containing a mix of active ingredients showed a quite high toxicity for collembolans but even greater for earthworms. The toxicity induced by the mixture of active ingredients was far below the toxic effects observed for the individual substance itself. This project will provide the basis for future studies aiming at characterizing the impact of wood preservatives on soil organisms in microcosms or in field conditions.

29. Möglichkeiten der Metabolomik in der Ökotoxikologie – Effekte von Kupfer auf das Metabolom der Nematoden Caenorhabditis elegans und Panagrellus redivivus

Spann Nicole, Tierökologie, Universität Bielefeld

Sarah Selck (Tierökologie), Walter Traunspurger (Tierökologie), Karsten Niehaus (Proteom- und Metabolomforschung), Kai Ristau (Tierökologie, alle Universität Bielefeld)

nicole.spann@uni-bielefeld.de

Die Untersuchung des Metaboloms von Organismen (Metabolomik) ist eine in der Ökotoxikologie noch relativ neue Technik. Sie ermöglicht die gleichzeitige Bestimmung einer großen Anzahl von kleinen organischen Molekülen (u.a. Zucker, Aminosäuren) und liefert ein umfassendes Bild über den Gesundheitszustand des Organismus. Durch den Einsatz der Metabolomik erhofft man sich neue stoffspezifische Biomarker zu finden und tiefere Erkenntnisse über den häufig noch nicht vollständig charakterisierten Wirkmechanismus von Umweltchemikalien zu erlangen.

In dieser Studie wurden die Auswirkungen von Kupfer auf die Reproduktion und insbesondere das Metabolom von zwei Nematodenarten untersucht. Panagrellus redivivus reagierte sensitiver auf Kupfer als Caenorhabditis elegans, resultierend in einem niedrigeren EC50 für den Endpunkt Reproduktion. Für die folgende Untersuchung des Metaboloms der Tiere wurden die Nematoden für 24 Stunden einem Kupferstress ausgesetzt (drei Behandlungen: 1.) Kontrolle ohne Kupfer, 2.) Konzentration entsprechend der Hälfte des EC50s für Reproduktion, 3.) Konzentration entsprechend dem EC50).

Bei beiden Arten führten die zwei Kupferbehandlungen zu einer veränderten Metabolomzusammensetzung im Vergleich zur Kontrolle. Interessanterweise waren die detektierten Veränderungen jedoch für die zwei untersuchten Arten verschieden, d.h. artspezifisch. Die Ergebnisse der Metabolomik könnten daher helfen die unterschiedliche Sensitivität, die auch beim Parameter Reproduktion hervortrat, mit Hilfe der Wirkungen auf der Stoffwechselebene zu erklären. Der große Vorteil der Metabolomik ist dabei, dass eine große Anzahl von Stoffwechselprodukten erfasst werden können, sodass auch bis dahin unbekannte Wirkmechanismen offengelegt werden können.

Passive Sammler

30. PDMS Passivsammler ein ausgezeichnetes Werkzeug zur Identifikation von PCB Punktquellen in Gewässern

Zennegg Markus, Empa

Markus Zennegg (1), Etienne Vermeirssen (2), Nicolas Eichenberger (3), Marin Huser (4), Rico Ryser (5); (1) Eidgenössische Materialprüfungs- und Forschungsanstalt (Empa), Abteilung Advanced Analytical Technologies, 8600 Dübendorf, Schweiz; (2) Schweizerische Zentrum für angewandte Ökotoxikologie, Eawag-EPFL,8600 Dübendorf, Schweiz; (3) Office de l'environnement Canton du Jura, 2882 Saint-Ursanne, Suisse; (4) Amt für Umweltschutz und Energie (AUE), 4410 Liestal, Schweiz; (5) Amt für Wasser und Abfall (AWA), 3014 Bern, Schweiz;

markus.zennegg@empa.ch

Im Jahr 2007 wurden in Fischen aus der Saane im Kanton Freiburg stark erhöhte Gehalte an dioxinähnlichen polychlorierten Biphenylen (dioxin-like PCB, dl-PCB) gemessen. Die Werte übertrafen die Höchstgehalte der Fremd- und Inhaltsstoffverordnung (FIV) für Dioxine und dl-PCB von 8 pg WHO-TEQ/g Frischgewicht (FG) zum Teil um mehr als das Zehnfache. Daneben wurden im Verlauf weiterer Untersuchungen in Fischen aus der Birs im Abschnitt zwischen Choindez (Kt. Jura) und Münchenstein (Kt. Baselland) Konzentrationen bis 60 pg WHO-TEQ/g FG gemessen. Im Gegensatz zur Situation in der Saane, wo die verantwortliche PCB-Kontaminationsquelle rasch gefunden wurde - die ehemalige Deponie "La Pila" - war die PCB Quelle im Bereich der Birs zu diesem Zeitpunkt nicht bekannt. Durch den Einsatz von Passivsammlern aus Silikon (PDMS) an 13 Standorten, verteilt über einen Flussabschnitt von ca. 60 Kilometern, konnte der Haupteintragspfad der PCB in die Birs auf das Gebiet um Courrendlin, flussaufwärts von Delémont eingeschränkt werden. Basierend auf diesen neuen Erkenntnissen wurde ein enges Netz von Passivsammlern im Flussabschnitt oberhalb von Choindez bis unterhalb von Courrendlin gelegt. Die daraus gewonnen Resultate deuteten auf eine PCB-Quelle im Bereich eines Industriestandortes in Choindez hin. Nach weiteren Abklärungen und der Identifikation der PCB-Quelle veranlasste der Kanton Jura den Betreiber des Industriestandortes Massnahmen zu ergreifen, um das unabsichtliche Einleiten von PCB in die Birs zu verhindern und somit die Umwelt zu schützen. Um die getroffenen Massnahmen zu überprüfen wurde während mehreren Jahren die PCB Belastung der Birs, im Bereich um Choindez, durch den Einsatz von PDMS Passivsammlern kontrolliert. Ein deutlicher Rückgang der PCB Belastung konnte in den Jahren 2011 und 2012 anhand dieses Monitorings festgestellt werden. Der sehr positive Trend der PCB Belastung liess sich durch die Untersuchung an Fischen im Jahre 2013 bestätigen. Neueste Daten von Fischanalysen als auch Passivsammlern aus dem Jahre 2014 und 2015 zeigen, dass sich die Situation an der Birs, bezüglich der PCB Belastung, deutlich verbessert hat und die erlaubten Höchstkonzentrationen, von neuerdings 6.5 pg/g FG, problemlos eingehalten werden.

Es ist erstaunlich, dass über zwanzig Jahre nach dem Totalverbot von PCB in der Schweiz vereinzelt immer noch viel zu hohe PCB-Gehalte in Fischen detektiert werden. Diese klare Überschreitung der zulässigen Höchstkonzentration ist nicht auf diffusen Eintrag zurückzu-

führen, sondern deutet auf das Vorhandensein von spezifischen Punktquellen hin, wie die erwähnten Beispiele zeigen. Um den fortlaufenden Eintrag von PCB in die Gewässer zu unterbinden, sind solche Punktquellen rasch zu identifizieren, zu sanieren und zu eliminieren. Um dieses Ziel zu erreichen sind gezielte Abklärungen im Bereich von PCB-Verdachtsfällen wie Altlasten-, Industrie- oder Produktionsstandorten in Gewässernähe nötig. Als ausgezeichnete Methode zur PCB-Quellenidentifikation in Gewässern hat sich der Einsatz von PDMS Passivsammlern erwiesen.

31. Anwendung von Passivsammlern für das Monitoring hydrophober organischer Schadstoffe in Flüssen

Becker Benjamin, Bundesanstalt für Gewässerkunde

Anja Duffek, Peter Lepom, Albrecht Paschke, Christel Möhlenkamp, Evelyn Claus, Sebastian Buchinger, Sabine Schäfer

benjamin.becker@bafg.de

In einem vom Umweltbundesamt geförderten F&E-Projekt (Forschungskennziffer 3713 22 230) wird die Eignung von Passivsammlern für das Monitoring von hydrophoben organischen Chemikalien (HOC) in Flüssen untersucht. Als Sammelphase wurde Silikon ausgewählt, da sich dieses in zahlreichen Studien (u.a. Kraus et al. 2015) für die Untersuchung einer breiten Palette an Zielanalyten bewährt hat. Für das Projekt wurden zunächst Passivsammler an fünf Standorten in den deutschen Abschnitten der Flüsse Saar, Rhein, Elbe, Saale und Donau ausgebracht.

In den drei durchgeführten Kampagnen, im Zeitraum Mai bis Oktober 2014, konnten erfolgreich zeitgemittelte Konzentrationen (CTWA) von Polychlorierten Biphenylen (PCB), Polyzyklischen Aromatischen Kohlenwasserstoffen (PAK) und Dichlordiphenyltrichlorethan (DDT)-Metaboliten an den oben genannten Standorten bestimmt werden. Hierbei wurden signifikante Konzentrationsunterschiede zwischen den jeweiligen Standorten gefunden, die den erwarteten Verhältnissen aufgrund bisheriger konventioneller Monitoringuntersuchungen entsprechen. Zusätzlich zu den standortspezifischen Unterschieden konnten auch unterschiedliche Belastungsmuster der Standorte zwischen und innerhalb von Substanzklassen festgestellt werden. PCB-Kongenere und DDT Metabolite waren in der Elbe am höchsten konzentriert, wohingegen die höchsten Konzentrationen an PAK in der Saar am gemessen wurden. Hierbei waren die Kontaminationsmuster für PAK zwischen der Saar und der Elbe verschieden. Die Donau und der Rhein zeigten durchgehend eine signifikant niedrigere Belastung im Vergleich zu den anderen Flüssen. Insgesamt konnten mit den Passivsammlern Bestimmungsgrenzen bis in den sub-pg/L-Bereich erreicht werden.

In der Kampagne September/Oktober 2014 wurde darüber hinaus die Kopplung der passiven Probenahme mit Biotests für mutagene und endokrine Wirkung (Ames-Test und YES bzw. planarer YES) getestet. Für diesen Zweck wurden die Extrakte der Passivsammler umgelöst und anschließend im Biotest eingesetzt, bzw. wenn möglich der Extrakt direkt eingesetzt. Im Ames-Test mit dem Stamm YG 1041, welcher sensitiv für Nitroarene und aromatische Amine ist, zeigten vor allem Proben der Saar mutagene Wirkungen.

In weiteren Untersuchungen wird die passive Probenahme zur Ermittlung von Kontaminationsschwerpunkten und deren Kopplung mit Biostests eingesetzt.

Referenzen:

Kraus U.R., Theobald N., Gunold R., Paschke A., 2015. Prüfung und Validierung der Einsatzmöglichkeiten neuartiger Passivsammler für die Überwachung prioritärer Schadstoffe unter der WRRL, der MSRL und im Rahmen von HELCOM und OSPAR. Texte 25/2015, Umweltbundesamt (Hrsg.), Dessau-Roßlau, ISSN 1862-4804.

32. Combining passive sampling technologies with toxicity tests to assess effects of micropollutants from wastewater effluents on periphyton

Tlili Ahmed, Eawag

Renata Behra

ahmed.tlili@eawag.ch

Micropollutants (MPs) released into fresh waters via wastewater treatment plants (WWTPs) are recognized as a global problem contributing to the degradation of ecosystem quality. Therefore, the aim of this field study is to causally link real effects resulting from microbial community-level exposure to complex mixture of MPs present in WWTPs effluents. To achieve this goal, we combined time-integrative passive sampling technologies with the assessment of extract toxicity via bioassays; using in-situ sampled periphyton.

Periphyton were sampled up- and downstream of four WWTPs effluents in Switzerland and exposed to various dilutions of extracts from passive samplers that were immersed at the effluent of each site. Tolerance of communities to passive sampler extracts was determined according to the pollution-induced community tolerance (PICT) concept by measuring the inhibition of various functional endpoints. Specific tolerance of the heterotrophic component of periphyton was measured by 14C-leucine incorporation and MicroRespTM method to measure secondary production and heterotrophic respiration, respectively. Specific tolerance of the phototrophic component of periphyton was assessed by 14C-carbonate assimilation and algal fluorescence to measure primary production and photosynthetic yield, respectively. Additionally, we examined shifts in the structure of up- and downstream periphyton by performing a community level physiological profile (CLPP) and a DNA fingerprint analysis.

Results showed that despite low concentrations of MPs detected downstream of the effluents, phototrophic and heterotrophic components of the sampled periphyton displayed increased tolerance towards these MPs. Interestingly, in comparison to heterotrophic functional endpoints, tolerance increase of phototrophic endpoints was variable among sites. These findings might give a first indication on the MPs composition in the effluents (dominance of herbicides or bactericides), which was confirmed by analytical identification of MPs in the passive samplers' extracts. The observed effects were accompanied by changes in the microbial CLPP and in molecular diversity. Overall, our study highlights the sensitivity of the proposed approach to detect effects of low MPs concentrations occurring in the field and to establish a causal link between exposure and the observed ecological effects on freshwater microbial communities.

33. Passive dosing of hydrophobic organic chemicals in toxicity tests with Caenorhabditis elegans

Fischer Fabian 1,2, Institut für Bodenkunde und Bodenerhaltung, IFZ für Umweltsicherung, Justus-Liebig-Universität Gießen

Leonard Böhm1, Christel Möhlenkamp2, Sebastian Höss3, Evelyn Claus2, Rolf-Alexander Düring1, Sabine Schäfer2 1) Institut für Bodenkunde und Bodenerhaltung, IFZ für Umweltsicherung, Justus-Liebig-Universität Gießen 2) Bundesanstalt für Gewässerkunde (BfG), Koblenz 3) Ecossa, Starnberg

fabian.chr.fischer@gmail.com

The chemicals' freely dissolved aqueous concentration (Cfree) is considered to be the bioavailable concentration for diffusive uptake and partitioning. Passive dosing is a new promising technique for controlling and maintaining constant Cfree during toxicity testing since possible analyte losses are compensated by partitioning of chemicals from a saturated reservoir.

This study investigated the applicability of passive dosing of hydrophobic organic chemicals (HOCs) in a chronic toxicity test with Caenorhabditis elegans using silicone O-rings as passive dosing polymer. For this purpose, 10 polycyclic aromatic hydrocarbons (PAHs) that cover a broad range of hydrophobicity were tested as model chemicals. Since Escherichia coli cells are used as food source for C. elegans in the standardized biotest, the effects of E. coli cells on Cfree in the test medium were investigated. During all experiments, Cfree of test chemicals were quantified by in situ solid-phase microextraction (SPME).

Studies on release kinetics of test chemicals revealed that after preincubating the silicone O-ring in the test medium for 24 hours at 60 rounds per minute most PAHs reached equilibrium partitioning. Although HOCs sorb to E. coli cells in C. elegans biotests, Cfree of PAHs were constant at three different E. coli cell densities in test media while applying PAHs by passive dosing. Headspace SPME was used to measure variations in Cfree of four PAHs over time during the entire C. elegans biotests after applying test chemicals either by passive dosing or by active spiking with a co-solvent. After applying PAHs by active spiking, Cfree decreased ≤ 90% until the end of the biotest. In contrast, Cfree remained constant while PAHs were applied by passive dosing.

Passive dosing of single PAHs at their respective maximum water solubility revealed that phenanthrene and pyrene showed with ≤ 100 % very high inhibition on growth and reproduction of C. elegans. Dose-response testing of both PAHs yielded EC50 values that were several orders of magnitude lower compared to values determined in conventional C. elegans tests.

In summary, passive dosing was successfully applied in the chronic toxicity test with C. elegans whereby in situ SPME proved to be suitable for measuring chemicals Cfree.

Umweltchemie

34. Non-extractable residues classification of chemicals in soil

Schäffer Andreas, RWTH Aachen University

Anne Berns, Jens Botterweck, Andrea Breitschwerdt, Philippe Corvini, Rong Ji, Thorsten Junge, Matthias Kästner, Timm Klausmeyer, Anja Miltner, Karolina Nowack, Claudia Possberg, Patrick Riefer, Burkhard Schmidt, Jan Schwarzbauer

andreas.schaeffer@bio5.rwth-aachen.de

Xenobiotics in soil dissipate by transport, binding and degradation processes that depend on the physicochemical properties of the substance and the soil, and on environmental conditions. We will report on the immobilization mechanism of chemicals in soil. If isotope labelled compounds are used to establish a mass balance of their fate in soil, combustion of the thoroughly extracted soil usually reveals a third component beside extractable and volatile incl. mineralized residues, i.e., so called non-extractable residues (NER). NER vary in the amounts that are formed and in the type of binding (covalent vs entrapped) but most often nothing is known on the chemical structures of NER and the potential for long-term release and bioavailability to plants and soil organisms. As examples we will present data on the fate of various chemical compounds.

If bacteria use pollutants as carbon source, they will incorporate the pollutant-derived carbon and other elements into their biomass which later will be incorporated in soil organic matter and thus contributes to NER formation. This has been demonstrated in studies on the biodegradation of several isotope labeled pesticides and pharmaceuticals in soil, where the contribution of microbial biomass residues to NER in soil was quantified. The high amount of label found in biomolecules (fatty acids, amino acids) indicated that large amounts of NER of readily degradable chemicals are of biogenic origin. However, NER of persistent xenobiotics also contain immobilized parent compounds or their primary xenobiotic metabolites. Examples and a scheme for the environmental risk assessment of NER differentiating xenobiotic, either entrapped (type I) or covalently bound (type II), and biogenic residues (type III) will be presented.

Risk assessment of a chemical in soil considers the formation of NER as a process of irreversible binding to soil; such residues are defined as not bioavailable. However, as shown in the examples of the lecture, xenobiotic NER formation may be considered as long-term stabilization of a chemical in soil from which parts may be released. It is reasonable to assume that all chemicals will form NER to various extents leading to complex mixtures of pollutants and their transformation products in soils and sediments. We therefore propose that the environmental risk assessment of chemicals should consider not only dissipation half lives (DT50) due to transport processes, degradation, and formation of NER; rather the potential subsequent release of non-extractable parent substances and xenobiotic metabolites should be taken into account. On the other hand, biogenic NER, formed by readily biodegradable chemicals, are of no environmental concern.

35. Distribution patterns of synthetic pyrethroids in estuarine and delta sediments in Europe

Schinkel Lena, Helmholtz-Zentrum für Umweltforschung
Riccardo Massei, Martin Krauss, Werner Brack

Lena.schinkel@gmx.de

Synthethic pyrethroids (sPYR) are a class of insecticides used in crop protection and in biocidal applications. They are of emerging concern because of their high toxicity towards aquatic organisms. They sorb to soil organic matter and enter water bodies via run-off, where they potentially accumulate in aquatic sediments and can affect benthic organisms. In this study 12 sPYR were chosen for target analysis based on the approval for agricultural and biocidal application within the European Union.

A GC-NCI-MS/MS method has been developed to analyse sPYR in aquatic sediments. A multi-target method was used for sample preparation, including pressurised liquid extraction with subsequent normal phase column chromatography. Massive signal enhancement of sPYR was observed when analysing sediment matrix. Therefore a matrix-matched calibration was performed with non contaminated sediment. Method detection limits ranged between 0.02 and 1.43 ng g 1. Recoveries varied between 62 and 82 %. So far, sediments from different sampling spots at the delta of Danube (3 spots) and Po (4 spots) were analysed for the occurrence of sPYR. Six target compounds were detected in at least one sample (range 0.01 - 3.55 ng g^{-1}). Highest levels were detected for cypermethrin (range 0.71 - 3.55 ng g^{-1}). Toxic units (TU) has been derived based on a previous study about sediment toxicity concerning H. azteca (Amweg et al 2005). TU ranged between 0.01 and 0.18 for individual sPYR. This indicates that the found level theoretically did not cause acute toxicity. However, possible chronic toxicity cannot be excluded.

Sediments of 12 further European river estuaries will be screened for the occurence of sPYR and distribution patterns will be statistically evaluated.

36. Katalytischer Abbau persistenter organischer Schadstoffe – Wettlauf der Analytik mit dem Transformationsgeschehen

Düring Rolf-Alexander, Institut für Bodenkunde und Bodenerhaltung, IFZ für Umweltsicherung, Justus-Liebig-Universität Gießen

Leonard Böhm, Institut für Bodenkunde und Bodenerhaltung, IFZ für Umweltsicherung, Justus-Liebig-Universität Gießen

Rolf-Alexander.Duering@umwelt.uni-giessen.de

Persistente organische Schadstoffe (POPs – persistent organic pollutants), eine der umweltchemisch großen globalen Herausforderungen, sind weltweit ”geächtet”, d. h. deren Herstellung, Inverkehrbringung und Gebrauch sind weitestgehend zu vermeiden. Eine kurzfristige Entlastung von Ökosystemen und der Nahrungskette wird mit diesen Vorgaben jedoch aufgrund der Persistenz dieser Stoffe nicht zu erreichen sein.

Mit dem Einsatz von Palladium-Nanopartikeln (Pd-NP) als effizienten Katalysatoren kann ihr Abbau über reduktive Dehalogenierung mit sehr hohen Umsatzraten erzielt werden. Schrittweise werden dabei die Chloratome durch Wasserstoffatome ersetzt.

Pd wird als Katalysator in der chemischen Industrie benötigt und kann technisch oder mikrobiell recycliert werden.

Im Rahmen des Verbundvorhabens NanoPOP werden an der Justus-Liebig-Universität Gießen Pd-NPs unterschiedlicher Herkunft hinsichtlich ihrer katalytischen Aktivität bewertet.

Erste Ergebnisse zeigten Pd-Nanopartikel als effiziente Katalysatoren zur reduktiven Dechlorierung von Hexachlorbenzol (HCB). Hier wurde der Abbau umweltrelevanter HCB Konzentrationen mit möglichst geringen Mengen an Pd(0)-Nanopartikeln untersucht. Mit einer automatisierten Extraktionsmethode (HS-SPME GC-MS) konnte hierbei der Anteil frei gelösten Schadstoffs in der Wasserphase über die Extraktion aus dem Gasraum und anschließender GC-MS-Analyse bestimmt werden. Je nach Versuchsbedingungen war ein nahezu vollständiger Abbau verschiedener POPs innerhalb weniger Minuten oder Stunden möglich. Langzeitversuche mit zugesetztem Hemmstoff erlaubten die Darstellung des Abbauwegs von HCB über die Bildung der Transformationsprodukte bis zum Benzol. Da die Verfolgung der raschen Dechlorierung mit einer Identifizierung der Transformationsprodukte mit der HS-SPME GC-MS Methode in Echtzeit nicht möglich war, wurde mit der Proton-transfer-reaction time-of-flight mass spectrometry (PTR-ToF-MS) zusätzlich eine on-line Methode zur Aufklärung der Abbauwege verschiedener halogenierter Verbindungen eingesetzt.

37. Hohe PCB Kontamination detektiert in Kalbfleisch aus extensiver Produktion – eine Fallstudie

Zennegg Markus, Empa

Schmid P (1), Kuchen A (2), Beer M (2), Tamborini L (3), Beckmann M (4), Arpagaus S (5), Caduff A (6), and Lanfranchi M (7); (1) Swiss Federal Institute for Materials Science and Technology (Empa), CH-8600 Dübendorf, Switzerland; (2) Federal Food Safety and Veterinary Office FSVO, CH-3003 Berne, Switzerland; (3) Federal Office for Agriculture FOAG, CH-3003 Berne, Switzerland; (4) Cantonal Food Safety and Animal Health Office, CH-7001 Chur, Switzerland; (5) Cantonal Food Safety Authority, Switzerland; (6) Agricultural Education and Extension Center, Plantahof, CH-7302 Landquard, Switzerland; (7) Agency for Nature and Environment of the Canton of Grison, CH-7001 Chur, Switzerland

markus.zennegg@empa.ch

Seit mehr als 40 Jahren sind polychlorierte Biphenyle (PCB) in offenen Anwendungen (z.B. Fugendichtungsmassen, Farbanstrichen, Korrosionsschutzanstrichen usw.) in der Schweiz verboten und seit 1986 gilt ein Totalverbort. Nichts desto trotz sind die zu den persistenten organischen Schadstoffen (POP) gehörenden und unter der Stockholmkonvention geächteten PCB immer noch in einer Vielzahl von technischen Reservoirs, Umweltkompartimenten und Altlasten vorhanden. Aus diesen Reservoirs können PCB freigesetzt werden und via die Umwelt in die Nahrungskette gelangen.

Regelmässig von den Behörden durchgeführte Untersuchungsprogramme im Bereich der Lebens- und Futtermittel zeigen, dass auch heute noch mit Überschreitungen der erlaubten Höchstkonzentrationen (HK) in tierischen Lebensmitteln zu rechnen ist. Drei durchgeführte Monitoring Programme in den Jahren 2003, 2006 und 2008 zeigten, dass Kalbfleisch aus extensiver Produktion gelegentlich die HK von 4 pg WHO-TEQ/g Fett überschreitet. Dies war der Auslöser für eine weitere Untersuchung von Kalbfleisch aus extensiver Produktion im Jahre 2012/2013. 60 Fleischproben von 53 Höfen wurden auf Dioxine (PCDD/F) und dioxinähnliche PCB (dl-PCB) untersucht. Fleisch von drei Höfen überschritt die zulässige HK, dies entspricht immerhin 5% der untersuchten Proben. Während zwei Höfe nur leichte Überschreitungen der HK aufwiesen zeigte ein Hof eine massive Belastung mit PCB. Die im Kalbfleisch dieses Hofes gemessenen Konzentrationen lagen beinahe einen Faktor von fünf oberhalb der HK von 4 pg WHO-TEQ/g Fett. Die zuständigen kantonalen Behörden verhängten ein Verkaufsverbot für jegliches Fleisch von diesem Hof. Erste Analysen der Futtermittel (Silage und Grünfutter) zeigten keine erhöhte PCB Belastung. Abklärende Untersuchungen auf dem Hof zeigten jedoch, dass mehrere Wandfarben im Stallgebäude PCB enthielten und zwar im Konzentrationsbereich von mehreren Prozenten. Diese Farben, welche vor mehr als 40 Jahren aufgetragen wurden, konnten für die Kontamination der Mutterkühe und ihrer Kälber verantwortlich gemacht werden. Das Austragen von ebenfalls PCB belastetem Kuhmist führte zusätzlich zu einem erhöhten PCB Eintrag in die Felder des Bauern. Der Hof musste schlussendlich für rund 250‘000.- CHF saniert werden. Es stellt sich die Frage wie viele Bauernhöfe mit einem ähnlichen Baujahr ebenfalls PCB belastete Farbanstriche verwendeten. In der Schweiz gibt es rund 40‘000 Rinderfarmen. Ist das Problem vielleicht grösser als erwartet? Die Fallstudie zeigt schön, dass auch Jahrzehnte nach dem

Anwendungsverbot von PCB immer noch mit belasteten Gebäuden bzw. Gebäudeteilen zu rechnen ist. Diese wiederum setzen, wie im geschilderten Fall gezeigt, PCB frei und kontaminieren die Umwelt bis hin zu Nahrungsmitteln die schlussendlich auf unserem Teller landen. Die PCB, als klassische Umweltchemikalien, dienen somit als warnendes Beispiel für andere Stoffe die in grösseren Mengen in Bauchemikalien und Bauprodukten eingesetzt werden wie z.B. bromierte Flammschutzmittel, Weichmacher, polychlorierte Paraffine usw. Diese müssen vor ihrem Einsatz auf Unbedenklichkeit und Umweltverträglichkeit geprüft werden und nicht erst danach.

Monitoring von Oberflächengewässern

38. Mikroverunreinigungen in bernischen Gewässern: Belastungssituation und neue ökotoxikologische Beurteilung der Risiken

Ochsenbein Ueli, AWA Amt für Wasser und Abfall des Kantons Bern/Gewässer- und Bodenschutzlabor, Schermenweg 11, 3014 Bern

Jean-Daniel Berset1, Elmar Scheiwiller1, Marion Junghans2 1 AWA Amt für Wasser und Abfall des Kantons Bern/Gewässer- und Bodenschutzlabor, Schermenweg 11, 3014 Bern, Schweiz 2 Schweizerisches Zentrum für angewandte Ökotoxikologie Eawag-EPFL, Überlandstrasse 133, 8600 Dübendorf, Schweiz

ueli.ochsenbein@gmx.ch

In der vorliegenden Studie wurden 5 Fliessgewässer und 13 Seen sowohl in voralpinen als auch in Mittellandeinzugsgebieten des Kantons Bern auf über 80 Mikroverunreinigungen untersucht. Aus den Fliessgewässern wurden Wochensammelproben von Frühjahr bis Herbst entnommen, während in jedem See 4 bis 6 Tiefenprofile untersucht wurden.

Die Gesamtbelastung an Mikroverunreinigungen in Wochensammelproben war in den voralpinen Flüssen tief, in den Mittellandflüssen wurden dagegen deutlich höhere Konzentrationen gemessen (Summe bis ca. 6.5 µg/l). Während der Applikationsperiode wurden zudem deutlich erhöhte Pestizidkonzentrationen ermittelt (Summe bis 4 µg/l). Dies weist darauf hin, dass die Spritztätigkeit in der Landwirtschaft einen direkten Einfluss auf den Pestizidgehalt dieser Gewässer ausübte.

Zur wirkungsbasierten Bewertung der Messergebnisse wurde eine neue Methode angewendet, welche die Abschätzung des Schadstoffrisikos für die Organismengruppen der Algen, der Wirbellosen und der Fische erlaubt. Die Untersuchungsergebnisse zeigten, dass in den Mittellandgewässern insbesondere im Frühjahr ein erhebliches Schadstoffrisiko für Gewässerorganismen vorhanden war. Dieses war zurückzuführen auf die Belastung durch Pestizide. In einem Gewässer, in dem Abwasser nur ungenügend verdünnt wird, bestand wegen der Belastung mit dem Schmerzmittel Diclofenac zusätzlich ein permanentes Risiko für Fische.

Die Messungen zeigten weiter, dass Pestizid-Transformationsprodukte vor allem in den Mittellandgewässern ubiquitär vorhanden waren. In den untersuchten Seen überwog die Summe von 18 gemessenen TP gegenüber den zugehörigen 12 Ausgangswirkstoffen stark. Je nach Mittellandsee bewegte sich der Anteil der TP zwischen ca. 75 und 98 Prozent. Gemäss GSchV sollen in Gewässern keine künstlichen, langlebigen Stoffe vorhanden sein. Die TP fallen in diese Kategorie.

Um die Gewässerorganismen vor schädlichen Einwirkungen zu schützen, sind - vor allem im Mittelland - weitere Anstrengungen zur Reduktion der der Schadstoffbelastungen unbedingt erforderlich.

39. Beurteilungskonzept für Mikroverunreinigungen aus diffusen Einträgen

Wittmer Irene, Umweltchemie, Eawag, Überlandstrasse 133, 8600 Dübendorf, Schweiz

Heinz Singer1, Christian Stamm1, Marion Junghans 2 1 Umweltchemie, Eawag, Überlandstrasse 133, 8600 Dübendorf, Schweiz 2 Schweizerisches Zentrum für angewandte Ökotoxikologie Eawag-EPFL, Überlandstrasse 133, 8600 Dübendorf, Schweiz

Irene.Wittmer@eawag.ch

Diffuse Einträge von Mikroverunreinigungen aus Landwirtschaft und Siedlung führen vor allem in kleinen, aber auch in mittleren und zum Teil sogar in grossen Fliessgewässern zu Spitzenkonzentrationen, die teilweise massiv über den gesetzlichen Anforderungen und auch über ökotoxikologischen Qualitätskriterien liegen. Ziel des vorgestellten Konzeptes ist es, praxistaugliche Empfehlungen für die Beurteilung des Zustandes eines Fliessgewässers in Bezug auf die Belastung durch diffuse Einträge von Mikroverunreinigungen zur Verfügung zu stellen. Eine schweizweite Analyse zeigt, dass Pflanzenschutzmittel und in geringerem Mass Biozide und Schwermetalle die wichtigsten diffus eingetragenen Stoffgruppen sind. Die dominierenden Eintragspfade sind oberflächliche Abschwemmung von behandelten Flächen, Drainagen, Drift, Regenwasserkanäle, Mischwasserüberläufe und die Strassenentwässerungen. Die meisten dieser Einträge erfolgen zeitlich hoch dynamisch, da die Mobilisierung an der Quelle und auch der Transport ins Gewässer mehrheitlich regengetrieben geschehen. Diese Dynamik sowie die Tatsache, dass die Einträge über weite Gewässerstrecken verteilt erfolgen, erschweren eine Erfassung und eine Beurteilung der Belastung. Eine vollständige Erfassung der ökotoxikologischen Effekte auf aquatische Organismen durch diffuse Einträge von Mikroverunreinigungen ist schwierig. Ein pragmatischer Ansatz ist der Vergleich von Messwerten mit ökotoxikologisch basierten Qualitätskriterien. Die Erfassung der akuten Belastung ist aufgrund der hohen Dynamik mit enormem Aufwand verbunden, und wird durch Stichproben nicht repräsentiert. Es wird deshalb empfohlen, zeitproportionale Zweiwochenmischproben zu ziehen und die chronische Belastungssituation zu beurteilen. Zweiwochenmischproben sind in Bezug auf Aufwand und Aussagekraft optimal. Für die Beurteilung wird das Verhältnis der gemessenen Konzentration eines Stoffes in einer zeitproproportionalen Zweiwochenmischprobe zum chronischen Qualitätskriterium bestimmt. Je nach Höhe dieses Verhältnisses, das auch als Risikoquotient (RQ) bezeichnet wird, fällt die Beurteilung zwischen sehr gut (RQ<0.1) bis schlecht (RQ> 10) aus. Das hier vorliegende Konzept schlägt eine Einteilung in die folgenden fünf Zustandsklassen vor: sehr gut – gut – mässig – unbefriedigend – schlecht.

40. EcoImpact – Auswirkungen von Mikroverunreinigungen aus Abwasser auf Fliessgewässerökosysteme: Ökotoxikologische und chemische Untersuchungen an 24 Schweizer Fliessgewässern

Kienle Cornelia, Schweizerisches Zentrum für Angewandte Ökotoxikologie Eawag-EPFL, Dübendorf, Schweiz

Sibylle Birrer, Fabian Deuber, Barbara Ganser, Nadzeya Homazava, Beatrice Läuppi, Janine Mazenauer, Marta Reyes, Sergio Santiago, Andrea Schifferli, Heinz Singer, Barbara Spycher, Etienne Vermeirssen, Christian Stamm, Inge Werner

cornelia.kienle@oekotoxzentrum.ch

Mikroverunreinigungen sind organische und anorganische Stoffe, die in sehr geringen Konzentrationen in Oberflächengewässern vorkommen. Einige von ihnen können bereits bei diesen geringen Konzentrationen Auswirkungen auf aquatische Organismen haben. Ziel des Projektes EcoImpact ist es, die Auswirkungen von Mikroverunreinigungen aus Kläranlagenabläufen auf Fliessgewässerökosysteme zu untersuchen. Hierfür kombiniert das Projekt chemische, mikrobiologische, ökotoxikologische und ökologische Untersuchungen, um die Prozesse in den Flussökosystemen möglichst gut zu verstehen. Dieser Beitrag fokusiert auf einen Teil des Projektes: die ökotoxikologischen und chemischen Untersuchungen.

In den Jahren 2013 und 2014 wurden mehrere Messkampagnen durchgeführt: 2013 erfolgte ein quantitatives Screening auf 400 Mikroverunreinigungen, ebenso wie ökotoxikologische Biotests im Labor und im Feld. An ausgewählten Stellen wurden die Frassaktivität von Amphipoden (Gammarus fossarum) ebenso wie die Fortpflanzung von Wasserflöhen (Ceriodaphnia dubia) untersucht. Desweiteren wurden Auswirkungen auf Photosynthese und Wachstum von einzelligen Grünalgen (Pseudokirchnerielle subcapitata), östogene Aktivität (Yeast Estrogen Screen), und neurotoxische Effekte (Acetylcholinesterase-Hemmtest) in allen Proben untersucht. Im Jahr 2014 wurden 60 Substanzen chemisch-analytisch erfasst und östrogene Aktivität, Algentoxizität und neurotoxische Effekte untersucht.

Die Parameter östrogene Aktivität und Photosynthesehemmung zeigten den Einfluss der Kläranlage gut auf: 17β-Estradiol-Äquivalenz-Konzentrationen (EEQs) waren im Fluss unterhalb der Kläranlage meist höher als oberhalb. Dies traf auch auf Diuron-Äquivalenz-Konzentrationen (DEQ) zu. Die mit chemischer Analytik gemessenen Konzentrationen an Östrogenen unterschätzten die östrogene Aktivität im Biotest teilweise, wohingegen die Konzentrationen an gemessenen Photosystem II-hemmenden Herbiziden gut mit den im Biotest gemessenen DEQ-Werten korrelierten. Eine verringerte Frassaktivität wurde an einer von vier Probenahmestellen gemessen, ebenso war die Fortpflanzung von C. dubia an einer von vier Stellen signifikant verringert.

Die angewendeten Methoden erwiesen sich als gut geeignet für eine chemische und ökotoxikologische Erfassung der Fliessgewässerqualität. Im Allgemeinen konnte die Belastung mit östrogenen Substanzen als relativ gering eingestuft werden: Die EEQs überschritten das effektbasierte Umweltqualitätskriterium (CQK) für 17β-Estradiol (0.4 ng/L) nur an einer Probenahmestelle. Dahingegen überschritten die DEQ-Werte aus dem Algentest in den meisten Fliessgewässern das CQK für Diuron (20 ng/L), teilweise bereits oberhalb der Abwasserrei-

nigungsanlage. Eine weitergehende Datenanalyse wird den Vergleich mit chemischen und ökologischen Daten einbeziehen, um das Gesamtbild zu vervollständigen und um weitere Schlussfolgerungen über die Auswirkungen von Mikroverunreinigungen auf Fliessgewässerökosysteme zu ziehen.

41. Water Quality Screening for Endocrine Disruption and Adaptive Stress Responses by in vitro bioassays in the River Danube in Serbia

König Maria, UFZ Leipzig

Peta Neale2, Muhammad Arslan Kamal Hashmi1, Beate Escher1, Werner Brack1 1 UFZ - Helmholtz Centre for Environmental Research, Leipzig 2 Smart Water Research Centre, Griffith University, Australia

maria.koenig@ufz.de

The river Danube, the second biggest European river, passes ten countries until it is entering the Black Sea. Along its course it runs through large cities like Vienna, Budapest, Novi Sad and Belgrade. This study, part of the project "SOLUTIONS", is dealing with the Danube around Novi Sad in Serbia where wastewater is discharged in the Danube without treatment. The aim is to investigate the toxicological effects of chemicals extracted from water samples upstream and downstream of the biggest wastewater effluent outflow as well as from a reference site. In addition to chemical screening all samples were analyzed with a battery of in vitro cell-based bioassays. The samples were investigated for a wide range of endpoints including receptor-mediated modes of action (ERα - estrogen receptor alpha, AR - androgen receptor, PPARy - peroxisome proliferation receptor gamma, PR - progesterone receptor, GR – glucocorticoid receptor) and adaptive stress responses (ARE – oxidative stress response, NFkB – inflammatory stress response, p53 – genotoxicity). Potential antagonism was also measured for all receptor mediated assays. The results showed a clear toxicological pattern between the different sampling sites. The most responsive endpoints were estrogenicity, anti-androgenicity, peroxisome proliferating responses and inflammation. This study showed the relevance of a well working wastewater treatment before releasing it into the environment as well as the monitoring of water quality.

42. Bestimmung von kritischen chemischen Konzentrationen für benthische Invertebraten an Hand von deutschlandweiten Monitoring Daten

Berger Elisabeth, Senckenberg Gesellschaft für Naturforschung

Dr. Andrea Sundermann Prof. Dr. Peter Haase

elisabeth.berger@senckenberg.de

Flüsse spielen eine besondere ökologisch wie gesellschaftliche Rolle. Um Flussökosysteme vor chemischer Verschmutzung zu schützen, wurden in der EG Wasserrahmenrichtlinie Umweltqualitätsnomen für eine Reihe von Substanzen definiert. Diese Umweltqualitätsnomen basieren auf Laboruntersuchungen mit Einzelsubstanzen und Modellorganismen.

In dieser Studie nutzen wir die im Rahmen der EG Wasserrahmenrichtlinie flächendeckend gesammelten Monitoring-Daten, um mit Hilfe von TITAN (Threshold Indicator Taxa Analysis) die chemischen Konzentrationen zu bestimmen, die in der Umwelt zu einem Rückgang im Vorkommen und der Abundanz von sensiblen Invertebraten führen. Der Fokus liegt auf 25 Substanzen, welche in dem Untersuchungsgebebiet am meisten gemessen und am häufigsten gefunden worden sind. Diese umfassen Pestizide (Isoproturon, Boscalid, Terbutylazin), poly-und monozyklische aromatische Kohlenwasserstoffe (Benzo(a)pyren, Fluoranthen, Anthrazen, Benzol, Toluol) und eine große Gruppe an Abwasser assoziierten Substanzen wie Pharmazeutika, Weichmacher, Flammschutzmittel und Komplexbildnern.

Generell lagen die als kritisch identifizierten chemischen Konzentrationen deutlich unterhalb derer von Umweltqualitätsnormen und anderen PNEC-Werten (predicted no-effect concentration). Nur die sehr strengen Grenzwerte für Benzo(a)pyren und Fluoranthen scheinen die Umwelt ausreichend zu schützen. Als besonders empfindlich konnten verschiedene Taxa aus den Gruppen Plecoptera, Trichoptera, Coleoptera, Diptera and Ephemeroptera identifiziert werden. Diese Ergebnisse werden im Vortag im Kontext von multiplen Stressen diskutiert.

Es wird deutlich, dass die chemische Verschmutzung eine bedeutende Rolle für das Artvorkommen in deutschen Fließgewässern spielt und daher mehr getan werden muss, um den Chemikalieneintrag in Gewässern zu reduzieren.

43. Wie stressorspezifisch sind traitbasierte Indizes?

Kattwinkel Mira, Systemanalyse und Modellierung, Eawag, Schweiz

Christian Stamm, Umweltchemie, Eawag, Schweiz; Nele Schuwirth, Systemanalyse und Modellierung, Eawag, Schweiz

mira.kattwinkel@eawag.ch

Makrozoobenthos-Monitoring erlaubt Rückschlüsse auf die ökologische Qualität von Gewässern. Stressorspezifische Indizes unterstützen die Identifikation der Ursachen für einen schlechten ökologischen Zustand und können damit zur Priorisierung von weiterführenden Untersuchungen und Managementmaßnahmen beitragen. In Felddaten ist jedoch oft eine Korrelation zwischen Indizes für verschiedene Stressoren zu finden. Dies führt zu uneindeutigen Aussagen über die vorherrschenden Stressoren und stellt die Spezifizität der Indizes in Frage. Diese Korrelation kann zwei Ursachen haben: (1) Die Stressoren sind im Feld korreliert; die Korrelation der Indizes gibt also ein richtiges Bild wieder. (2) Die Sensitivitäten der Taxa gegenüber den Stressoren sind korreliert; diese inhärente Korrelation führt zu einer geringen Spezifität der Indizes.

Da Feldmessungen über große Gradienten verschiedener Stressoren häufig fehlen, ist eine Überprüfung der Spezifität auf diesem Wege schwierig. Wir stellen hier ein systematisches Vorgehen vor, um die Ursachen der Korrelation zu unterscheiden und damit ökologische Indizes für das Gewässermonitoring zu verbessern [1]. Am Beispiel des Saprobien-Index, des SPEARpesiticides und anderer Indizes demonstrieren wir die Methode. Zunächst untersuchen wir, ob die Klassifizierung in sensitive und nicht-sensitive Arten gegenüber verschiedenen Stressoren unabhängig verteilt ist. Dann schätzen wir mit Hilfe von Monte Carlo Simulationen zufälliger Gemeinschaften die inhärente Korrelation zwischen verschiedenen Indizes ab. Schließlich testen wir die Nullhypothese, dass die in Felddaten vorliegende Korrelation ausschließlich auf inhärente Korrelation zurückzuführen ist. Um den Einfluss der regionalen Biodiversität zu analysieren, benutzen wir für alle Analysen einerseits einen Taxonpool bestehend aus den Taxa zweier Einzugsgebiete im Schweizer Mittelland und andererseits einen größeren Taxonpool bestehend aus allen in der Schweiz vorkommenden Arten der Europäischen Ökoregion 8.

Im kleineren Taxonpool sind die inhärenten Korrelationen stärker, d.h. Indizes korrelieren ohne Korrelation der Stressoren. Allerdings ist die Korrelation in den Felddaten meist höher als allein auf Grund von inhärenter Korrelation zu erwarten wäre. Ein substantieller Anteil ist also auf korrelierte Umweltbedingungen zurückzuführen. Unsere Ergebnisse unterstreichen den Wert einer Korrelationsanalyse bei der Entwicklung stressorspezifischer, traitbasierter Indizes, insbesondere für den Einsatz in Regionen mit geringer Biodiversität, typisch für Gebiete mit einer langen Geschichte menschlicher Einflüsse.

[1] Schuwirth, Kattwinkel, Stamm 2014. How stressor specific are trait-based ecological indicators for ecosystem management? Sci. Total Environ. 505: 565-571

44. Vergleichende Toxizitätsuntersuchung von Carbamazepin und seiner Hauptmetaboliten gegenüber Chironomus riparius

Heye Katharina, Goethe-Universität Frankfurt am Main

Dennis Becker, Matthias Oetken, Jörg Oehlmann

heye@bio.uni-frankfurt.de

Carbamazepin (CBZ) gehört zu den am häufigsten verwendeten Antiepileptika und wird trotz rückläufiger Verbrauchsmengen in Deutschland regelmäßig in Oberflächengewässern nachgewiesen. Durch die Metabolisierung im Körper entstehen zudem etwa 30 unterschiedliche Metaboliten, die teilweise in höheren Konzentrationen als die Muttersubstanz gemessen werden und ebenfalls zu einer Gefährdung von Sedimentbewohnern beitragen können. Um erstmals Effekte der beiden Hauptmetaboliten, 10,11-Dihydro-10,11-dihydroxy-Carbamazepin (DiOH-CBZ) und Carbamazepin-10,11-epoxid (EP-CBZ) mit den Effekten von CBZ zu vergleichen und ein Risiko für Sedimentbewohner abschätzen zu können, wurden Lebenszyklustests mit der Zuckmücke Chironomus riparius durchgeführt.

Im Verlauf dieser Tests wurden Mortalität, Emergenzzeitpunkt, Geschlechterverhältnis, Gelegegröße und Fertilität untersucht, die als Grundlage für die Berechnung der Populationswachstumsrate dienten.

Die Untersuchung von CBZ und den beiden Hauptmetaboliten ergab mit einer LC50 (40 Tage) von 0,203 mg/kg als zeitlich gewichteter Mittelwert die höchste Toxizität für EP-CBZ, während DiOH-CBZ keine Effekte im untersuchten Konzentrationsbereich (0.171 mg/kg - 1.22 mg/kg) zeigte.

Obwohl CBZ eine niedrigere Toxizität (LC50: 1.11 mg/kg, zeitlich gewichteter Mittelwert) als EP-CBZ aufwies, kommt CBZ in der Umwelt in höheren Konzentrationen vor und birgt ein sehr hohes Risiko für Sedimentbewohner. Trotz der im Vergleich niedrigeren Umweltkonzentrationen von EP-CBZ besteht aufgrund der hohen Toxizität gleichwohl ein Umweltrisiko.

Diese Studie konnte dazu beitragen, die Bedeutung von Metaboliten für die Risikobewertung zu verdeutlichen. Trotz teilweise geringer Konzentrationen können Metaboliten wie auch die Muttersubstanz ein Risiko für die Umwelt bedeuten.

Danksagung – Die Untersuchungen wurden im Rahmen des EU-Projekts ENDETECH durchgeführt, das im 7. Rahmenprogramm für Forschung, technologische Entwicklung und Demonstration unter der Vertragsnummer 282818 gefördert wird.

Risikobewertung Pestizide

45. Vom Monitoring zur Regulierung von Pflanzenschutzmitteln

Knauer Katja, Bundesamt für Landwirtschaft

Annette Aldrich, Agroscope

katja.knauer@blw.admin.ch

Nationale Monitoring Programme zur Überwachung der Umwelt beschreiben den aktuellen Zustand der Umwelt wie z.B. der Oberflächengewässer, des Grundwassers, des Trinkwassers und des Bodens. Diese Programme werden in regelmäßigen Abständen durchgeführt und als Ergebnis steht eine enorme Menge an Messdaten zur Verfügung. Mit dem Monitoring überprüfen die Kantone in der Schweiz, ob die gesetzlichen Anforderungen z.B. an die Wasserqualität der Oberflächengewässer eingehalten werden. Da Pflanzenschutzmittel (PSM) häufig in Schweizer Oberflächengewässer gemessen werden, stellt sich die Frage nach der ökotoxikologischen wie ökologischen Relevanz der gemessenen Konzentrationen und der Bedeutung für die Regulierung von PSM. Erfüllen PSM die Anforderungen an die Gewässerqualität nicht, ist eine Überprüfung der Anwendungsvorschriften im Rahmen der Zulassung möglicherweise notwendig, um den Schutz der Oberflächengewässer vor unannehmbaren Effekten zu garantieren. Welche Kriterien und welche Vorgehensweise sich anbieten, um Monitoring Daten auszuwerten und auf einen möglichen Handlungsbedarf für die Zulassung hin zu analysieren, soll im Rahmen dieses Beitrags präsentiert und diskutiert werden.

46. Vergleichbarkeit modellierter PSM Konzentrationen mit Daten aus dem Schweizer Gewässermonitoring

Fulda Beate, Agroscope

Aldrich Annette, Balmer Marianne, Poiger Thomas

beate.fulda@agroscope.admin.ch

Ein wichtiger Bestandteil der Umweltrisikobeurteilung von Pflanzenschutzmitteln (PSM) ist die Abschätzung des Austrags von PSM von landwirtschaftlichen Flächen in angrenzende Oberflächengewässer. Für die Berechnung des potentiellen PSM Austrags durch Abschwemmung, Drainage und Abdrift, wird in der Schweiz derzeit das aquatische Expositionsmodell EXPOSIT 3.01 (Umweltbundesamt UBA, DE) sowie das Driftkonzept nach Rautmann (2001) verwendet. Diese Modelle sind so konzipiert, dass sie für eine spezifische PSM Anwendung eine worst-case Vorhersage der potentiellen Gewässerkonzentrationen geben. In dieser Studie wurde anhand von Daten aus dem Schweizer Gewässermonitoring von 2005 – 2012 geprüft, ob die Prognosen aus der Risikobeurteilung mit der tatsächlichen Gewässerbelastung übereinstimmen. Basierend auf der jeweiligen in der Schweiz zugelassenen worst-case Anwendungen eines Wirkstoffs, lagen die gemessenen Gewässerkonzentrationen (MEC) in nur wenigen Fällen über den entsprechenden Prognosewerten (PEC). Wenn bei der Wahl des worst-case Szenarios die spezifische Landnutzung im Einzugsgebiet mit berücksichtigt wurde, konnten deutlich mehr Überschreitungen festgestellt werden. Das Vergleichsresultat war somit stark abhängig vom gewählten PEC-Szenario. Hierbei ist zu beachten, dass in der Praxis ein gewisser Anwendungsspielraum für PSM Produkte besteht und daher auch die PEC Werte einzelner Szenarien eine gewisse Variabilität aufweisen können. Zudem müssen Unsicherheiten bei den Messwerten berücksichtigt werden, da viele Standorte beispielsweise nur strichprobenartig oder mit Methoden untersucht wurden, die keine vollständige Erfassung von Peak-Konzentrationen ermöglichen. Für eine detailliertere Evaluation der Expositionsmodelle in Hinblick auf mögliche Rückschlüsse für die Risikobeurteilung sind daher zum einen spezifische Studien in intensiv untersuchten Einzugsgebieten, inklusive detaillierte Angaben zur Landnutzung, sowie eine gute Kenntnis der landwirtschaftlichen Praxis notwendig.

47. Biodiversität und strukturelle Vielfalt in der Agrarlandschaft – eine übergeordnete Perspektive auch für die Pestizidzulassung?

Roß-Nickoll Martina, Institut f. Umweltforschung, RWTH Aachen University

Mark Deubert, Gottfried Lennartz, Richard Ottermanns, Andreas Schäffer, Björn Scholz-Starke, Lucas Streib, Andreas Toschki, Matthias Trapp

ross@bio5.rwth-aachen.de

Biodiversität ist die grundlegende Ressource, die ökosystemare Funktionen und Dienstleistungen garantiert. Da Biodiversität in der Landschaft auf verschiedenen Organisationsebenen realisiert ist, analysieren und klassifizieren wir die Verteilung von verschiedenen Organismengruppen, um die Wechselbeziehung zwischen Diversität und Strukturelementen oder Lebensraumtypen zu verstehen.

Im ersten Teil wird ein Projekt vorgestellt, das in einem deutschlandweiten Gradienten die Lebensgemeinschaften von grasigen Feldrainen klassifiziert und hinsichtlich ihrer Diversität und deren Korrelation zu Standortfaktoren analysiert und diskutiert. Die Untersuchung umfasst 72 Standorte und stellt neben Daten zur Vegetation auch Daten von Tiergruppen wie Carabidae, Araneae, Coccinellidae, Saltatoria, Syrphidae und Hymenoptera, insgesamt über 800 Arten, zur Verfügung.

Es ist allgemein akzeptiert, wenn auch kontrovers diskutiert, dass Artenvielfalt und Habitatqualität sich mit steigender Intensivierung in der Agrarlandschaft dramatisch verschlechtern. Zu diesem Aspekt wird ein zweites Projekt vorgestellt, das die Landschaftsstruktur über die Klassifikation von Landschaftselementen und deren Bedeutung für die Biodiversität erfasst. Ein ökologischer Wert, der die Habitatqualität widerspiegelt, wird genutzt, um die räumliche Verteilung typischer Elemente der Agrarlandschaft am Beispiel von zwei Regionen in Deutschland darzustellen. Es werden 50 Typen ökologisch relevanter Landschaftselemente definiert, die automatisiert aus Fernerkundungs- und Flächennutzungsdaten generiert werden. Die Methodik ist gut etabliert, braucht aber noch die Kalibrierung für weitere Landschaftstypen. Im abschließenden Teil der Präsentation werden verschiedene Szenarien aufgezeigt, die im Rahmen der allgemeinen Agrarpolitik der EU und der Risikominderungsmaßnahmen in der Pflanzenschutzmittelzulassung geeignet wären, den ökologischen Wert der Landschaft zu verbessern. Um das Potenzial einer Landschaft zu adressieren, stellt sich dabei auch die Frage nach einem landschaftstypischen Leitbild für die Agrarlandschaft.

48. Nachhaltigkeitsbewertung von Insektiziden gegen Rapserdfloh

Mathis Marcel, Agroscope Gruppe Ökotoxikologie
S. Breitenmoser, J.-D. Charriere, A. Wunderlich, L. De Baan; O. Daniel

marcel.mathis@agroscope.admin.ch

Seit Herbst 2014 ist europaweit und auch in der Schweiz die Verwendung von Rapssaatgut, welches mit Neonicotinoiden gebeizt wurde, sistiert worden. Das Spritzen mit Pyrethroiden ist die einzige übrig gebliebene Bekämpfungsmassnahme gegen den Schädling Rapserdfloh.

In einer Studie von Agroscope wurden die derzeit gegen den Rapserdfloh erlaubten Bekämpfungsmassnahmen - Spritzen mit Pyrethroiden, mit der nicht mehr zugelassenen Beizung des Saatgutes mit Neonicotinoiden verglichen.

Um aus diesen zwei Möglichkeiten (Spritzung vs. Beizung) die nachhaltigere Strategie zu bestimmen, müssen verschiedene Aspekte der Ökologie und Ökonomie beurteilt und berücksichtig werden. Als Methode wurde eine multikriterielle Entscheidungsanalyse mit repräsentativen und praxisrelevanten Szenarien des Einsatzes von Pflanzenschutzmitteln gegen den Rapserdfloh durchgeführt. Berücksichtigt wurde auch der Einfluss der gesamten Spritzfolge und der örtlichen und zeitlichen Verteilung des Befalls mit Rapserdflöhen.

Für die betriebswirtschaftliche Analyse wurden Volkostenrechnungen für jedes definierte Szenario durchgeführt. Um die Nützlingsschonung einzuschätzen, wurden relevante Nützlinge im Rapsanbau, mittels einer Gefährdungskategorien beurteilt. Das aquatische Risiko wurde für die Organismengruppen Fische, Invertebraten und aquatische Pflanzen mittels der Synops-Methode bewertet. Das Risiko für Bienen wurde anhand von Gefährdungskategorien bewertet. Für die multikriterielle Entscheidungsanalyse wurde ein hierarchischer Bewertungsungsbaum durch Experten festgelegt. Mittels dem Bewertungsbaum und den ökologischen (Aquatik, Bienen, Nützlinge) und ökonomischen Analysen wurde die Nachhaltigkeitsbewertung vorgenommen. Die Methodik und Resultate erlauben eine faktenbasierte Beratung und Unterstützung von Entscheidungsprozessen. Im Vortrag werden mögliche Implikationen eines Verzichtes auf die Beizung mit Neonikotinoiden diskutiert.

49. Klimavariabilität und Insektizid-Anwendung – Indikatoren für kurz- und langfristige Expositions-Vorhersagen

Knillmann Saskia, UFZ- Helmholtzzentrum für Umweltforschung

Polina Orlinskiy, UFZ - Helmholtzzentrum für Umweltforschung Matthias Liess, UFZ - Helmholtzzentrum für Umweltforschung

saskia.knillmann@ufz.de

Der globale Klimawandel hat einen starken Einfluss auf die Landwirtschaft und den damit verbundenen Einsatz von Pestiziden. Besonders für den Insektizideinsatz in den gemäßigten Klimazonen wird eine Zunahme mit steigenden Temperaturen vorhergesagt. Neben diesem Vorhersagen, gibt es jedoch kaum quantitative Studien zur Zunahme der Einsatzraten, der Klimavariabilität und den ökologischen Folgen in aquatischen Ökosystemen.

Wir haben Daten zum Insektizideinsatz in Deutschland und Europa seit 1992 mit verschiedenen Klimadaten verknüpft und Indikatoren für den zukünftigen Einsatz identifiziert. Es wurde beobachtet, dass sich in Deutschland der Beginn der Insektizidanwendung von 1992 bis 2014 um mehr als vier Wochen zu einem früheren Zeitpunkt verschoben hat. Der Einsatzbeginn von Insektiziden korrelierte dabei sehr stark mit den Temperaturen und phänologischen Kenngrößen im jeweiligen Frühjahr ($r > 0.6$). Ähnliche Zusammenhänge mit den thermalen Bedingungen im Frühjahr konnten für den jährlichen Gesamteinsatz von Insektiziden in Deutschland und Europa gezeigt werden, wobei in Deutschland z.B. die Gesamtmenge zwischen 65 und 95 g/ha variierte.

Diese Ergebnisse zum Beginn und Menge des Insektizideinsatzes unter gemäßigten Klimabedingungen präsentieren wichtige Einblicke für das Monitoring von Pestiziden und der möglichen Abschätzung von Pestizideffekten in der Umwelt. Darüber hinaus sind solche Pestizidindikatoren als Basis für die Berechnung von zukünftigen Risikoszenarien notwendig.

50. The ecotoxicological ivory tower, or How ecotoxicology could increase its relevance

Schäfer Ralf B., Institute for Environmental Sciences, University Koblenz-Landau

Leo Posthuma, Centre DMG (Sustainability, Environment and Health), RIVM, Bilthoven, The Netherlands

schaefer-ralf@uni-landau.de

In our presentation, we raise three aspects that limit the relevance of ecotoxicology and risk assessment for real-world problems. First, we show that ecotoxicology has a strong focus on species and individuals, and that ecosystem- and community-based research plays a minor role. That is at variance with the latter often relating to the endpoint of concern. This aspect is mirrored in the attitude of several ecologists towards ecotoxicology. By contrast, ecologists conduct theory-driven research on communities and ecosystems, but rarely handle chemical stressors. Second, simple models are often regarded as a solution to extrapolate from known (single-species test) toxicity data to the ecosystem- and landscape-level. We argue that while such models are generally useful, their predictions need to be falsifiable under real-world conditions and should be tested accordingly with field data. Various studies demonstrate lessons to be learned here. Moreover, we caution against the idea to bridge the scales from biomarkers to ecosystems with a too narrow focus on chemical-oriented approaches as well as ignorance of ecological patterns. Third, we warn against the reification of entities from risk assessment such as the NOEC, HC5 or RAC, which implies handling the latter concepts as facts. Giving examples from medical epidemiology and from previous eco(toxico)logical studies, we argue that scientific hypotheses on the impacts of chemical mixtures in natural systems, for example in ecotoxicological epidemiology, should rely on science-based effect measures rather than be based on regulatory concepts like HC5. Only then will scientific insights provide improved fundaments to risk assessment and management. We end with suggestions how ecotoxicology could increase its relevance for the real world situation.

51. A critical review of prochloraz effects in fish

Perseke Lea, Dr. Knoell Consult GmbH
Lennart Weltje

lea.perseke@web.de

The imidazole fungicide prochloraz is registered in the EU for use on various crops and is one of the most data-rich substances regarding chronic toxicity to fish. The aim of this literature review was to investigate the available literature for consistency in observed effects and mechanisms and for potential differences in sensitivity between fish species and to discuss the relevance of prochloraz exposure for fish populations in the field.

Available literature on the effects of prochloraz on fish was identified through Web of Science, regulatory data and OECD test guideline validation studies. Results from a total of over 50 in vivo experiments are included in this review. Endpoints of interest were apical effects (survival, growth, reproduction, development) as well as biomarkers. The dominant effect of prochloraz in fish seems to be the inhibition of aromatase (CYP19), an enzyme which catalyses the conversion of testosterone to 17β-estradiol. Inhibition of aromatase leads to a lack of 17β-estradiol and a surplus of testosterone, causing various effects in (female) fish, such as reduction of 17β-estradiol concentrations, reduction of vitellogenin or a shift in the sex ratio towards males.

The effects reported in the literature were consistent with the known modes of action and showed that prochloraz may affect the endocrine system of fish and cause effects on growth, with LOECs roughly ranging from 30 to 300 µg/L. Apart from a few exceptions, effects followed monotonic concentration-response curves. The sensitivity of different fish species and endpoints is quite comparable, except that medaka might be more sensitive regarding reproduction. Aquatic invertebrates and algae show a higher sensitivity to short-term exposures than fish.

Measured environmental concentrations of prochloraz in natural surface waters are about 1000 times below the median concentrations causing effects in laboratory tests with fish under continuous exposure. These data suggest that the exposure of prochloraz in the field is too low to cause effects in fish. This notion is supported by the fact that no effects related to prochloraz have been found in natural fish populations.

52. Use of the "Maximum Cumulative Ratio" (MCR) as a tool for prioritization of potential exposures to mixtures of Plant Protection Products: a case study based on surface water monitoring in the USA

Vallotton Nathalie, Dow Europe GmbH, Horgen

Price Paul S. The Dow Chemical Company Toxicology and Environmental Research and Consulting (TERC) 1803 Building Midland MI USA

navallotton@dow.com

Environmental exposure to plant protection products (PPP) can often occur in scenarios wherein multiple PPP are applied or persisting within the same ecosystem. While current regulatory programs evaluate risks on a chemical-by-chemical basis, there is considerable interest in assessing the potential impact of concurrent exposures to multiple PPP. The potential risk associated with PPP concentrations measured in approximately 3000 surface water samples from across the USA (1992 -2001) were evaluated using a tiered approach recommended by the World Health Organization (WHO). The lack of a reported ecotoxicity threshold (i.e., benchmark value) and the occurrence of non-detectable PPP in some monitoring samples were considered with regard to their potential impacts on the risk analysis. In the first-tier assessment, the potential risk from the exposure to each individual PPP was calculated based on its concentration and the lowest aquatic life benchmark (U.S. EPA's Office of Pesticide Program). The potential risk associated with combined exposure of PPP was conservatively derived by assuming a common mode of action and additivity for these effects. The results from the Tier 1 assessed with the MCR approach identified 7.6% of the samples for which a risk alert could not otherwise be identified when evaluating the risk on a chemical-by-chemical basis. In the tier 2 assessment, consideration was given to information on mode of action and defined target species. Separate risk assessments were performed for each ecotoxicity benchmark, which would be considered a Tier 2 analysis in the WHO framework and the Cefic-MIAT decision tree. The findings of Tier 2 were that potential risk from combined exposures was an issue in only 1% of the samples. These concerns were limited to a small subset of PPPs for which only invertebrates and non-vascular plants were identified as being most sensitive. This tiered risk assessment approach combined with the MCR analysis strongly supported the value of categorizing monitoring samples in terms of whether the combined exposures pose little or no risk, where one or more chemicals exceed safe concentrations, and finally to identify exposures where several substances could make a significant contribution to the combined risk to specific non-targets organisms.

Multiple Stressoren

53. Kombinierte Wirkung von Schadstoffen und Umweltstressoren

Liess Matthias, UFZ
matthias.liess@ufz.de

Individuen und Populationen werden im Freiland durch eine Kombination von Schadstoffen und Umweltstressoren belastet. Bisherige Untersuchen deuten darauf hin, dass Umweltstressoren wie Futtermangel, Räuberdruck und Wärmebelastung die Wirkung von Schadstoffen auf Individuen und Populationen verstärken.

Der Vortrag zeigt die Vielfalt möglicher Stressor-Kombinationen und ihrer Kombinationswirkungen auf. Auf der Basis dieser Beobachtungen wird ein universeller Ansatz der Interpretation von kombinierten Wirkungen vorgestellt.

Die Berücksichtigung kombinierter Wirkungen von Schadstoffen und Umweltstressoren ermöglicht eine verbesserte Risikovorhersage. Hierdurch kann das Risiko unvorhergesehener Umweltschäden vermindert werden - und Sicherheitsfaktoren qualifiziert verringert werden.

54. Combined effects of parasites, climate and pollution on brown trout, Salmo trutta, in Swiss rivers

Segner Helmut, Cetre for Fish and Wildlife Health, University of Bern

Thomas Wahli Heike Schmidt-Posthaus Centre for Fish and Wildlife Health, University of Bern
Patricia-Burkhardt-Holm Mensch-Gesellschaft-Umwelt, University of Basel

helmut.segner@vetsuisse.unibe.ch

Stocks of brown trout in Swiss rivers have experienced a drastic decline over the last 20-30 years. This appears to be caused not by a single factor but by the cumulative impact of multiple stressors. One key factor is the infection of brown trout with the myxozoan parasite, Tetracapsuloides bryosalmonae, the etiological agent of proliferative kidney disease (PKD). The disease is widespread among brown trout populations in Switzerland. PKD-induced mortalities depend on water temperature, with negligible mortalities occurring at temperatures up to 12 – 14□C, but strongly increasing mortalities at higher water temperatures. During the last decades, water temperatures of Swiss rivers experienced a climate change-related increase of water temperatures, both of mean values and of summer maxima. The increase was most pronounced in the lowland regions (< 800 m altitude), which is also the region where the highest frequency of PKD infections is observed. The spatial and temporal co-incidence between PKD infection and changes of water suggests a role of this stressor combination in the observed brown trout decline. Brown trout populations in lowland river stretches can be additionally impacted by chemical pollutants due to the strong industrialization and urbanization of the Swiss lowland regions. Chemical exposure could increase susceptibility of trout to the parasite, since many chemicals, particularly compounds binding to estrogen and arylhydrocarbon receptors, have immunosuppressive effects in fish. In conclusion, the brown trout decline in Swiss rivers appears to be mediated through a combination of environmental stressors.

Acknowledgement: This study is financially supported by the EU project MODELKEY and the Swiss National Science Foundation.

55. Cyanobacteria and Daphnia parasite epidemics

P. Spaak[1]
C. Tellenbach[1], N. Tardent[1, 2], B. Keller[3], F. Pomati[1], J. Wolinska[4]
[1]Department of Aquatic Ecology, Eawag, Dübendorf, Switzerland; [2]University Zurich, Zurich, Switzerland; [3]Institute of Systematic Botany, University Zurich, Zurich, Switzerland; [4]Department of Ecosystem Research, Leibniz-Institute of Freshwater Ecology and Inland Fisheries, Berlin 12587, Germany

spaak@eawag.ch

In eutrophic lakes cyanobacterial blooms occur frequently, triggering a cascade of devastating effects for the lake community. Cyanobacteria affect other species in multifarious ways: first, some cyanobacteria taxa are well-known for producing secondary metabolites, which are highly toxic for humans and wildlife. However, the production of cyanotoxins varies within species, with some strains lacking the necessary genes. Second, a high turn-over of blooming cyanobacteria leads to an oxygen shortage in the lake due to decomposition of dead cells at lower depths. Third, the dominance of cyanobacteria in the phytoplankton community will lead to a shift in algal food quality for herbivorous zooplankton, which in turn affects higher levels of the food web. Cyanobacteria lack essential fatty acids, impairing herbivore growth, reproduction, and metabolism. Since Daphnia play a key-role in lake and pond food webs, the direct effect of cyanobacteria has intensely been studied. Whilst the role of cyanobacteria as poor nutrient source for Daphnia is now well-understood, the importance of morphology and toxin production is still controversial. Moreover, cyanobacteria might also have indirect effects on Daphnia in natural systems, by modifying host-parasite interactions. Here, we will show the synchronous occurrence of cyanobacteria and epidemics of the common Daphnia gut parasite Caullerya mesnili in a middle-sized peri-alpine lake over several years. We further show the results of an infection experiment with Microcystis aeruginosa and discuss the involvement of environmental parameters, cyanobacteria in general and microcystins in Caullerya epidemics.
Keywords: Microcystis aeruginosa, Caullerya mesnili, microcystins, species interactions, epidemiology

56. Time-lag effects of combined pesticide and parasite stressors on honey bees

Straub Lars, Institute of Bee Health, University of Bern

Lars Straub1,2, Geoffrey Williams1,2,3, Kitiphong Khongphinitbunjong3, Annette Schneeberger1, Gina Retschnig1, Panuwan Chantawannakul3, Vincent Dietemann2,4, Peter Neumann1,2,4
1.Institute of Bee Health, Vetsuisse Faculty, University of Bern, Bern, Switzerland 2. Agroscope, Swiss Bee Research Centre, Bern, Switzerland 3. Bee Protection Center, Department of Biology, Faculty of Science, Chiang Mai University, Chiang Mai, Thailand. 4. Department of Zoology and Entomology, University of Pretoria, Pretoria, South Africa

lars.straub@vetsuisse.unibe.ch

The ectoparasitic mite Varroa destructor and agricultural chemicals can have drastic effects on honey bee, Apis mellifera, health; however, possible combined effects of pesticides and V. destructor have so far been unexplored. Here, we evaluated possible sub-lethal (teneral body mass) and lethal (longevity) time-lag effects of two neonicotinoids (thiamethoxam and clothinadin) and V. destructor on individual workers alone and in combination. Queenright colonies of similar strength (N=20) were randomly allocated and exposed to one of two treatments (1. Both neonicotinoid pesticides or 2. No neonicotinoid pesticides) for 8 weeks in late Spring 2014 that represented environmentally relevant conditions. One (Summer 2014) and 16 (Fall 2014) weeks post treatment exposure, emerging workers from all colonies were individually screened for mite infestations and transferred to cages (N=3 cages per colony; 10 workers per cage) to examine possible time-lag effects of the pesticides and mite on survival and body weight. In Summer 2014, longevity was reduced by V. destructor, but not by thiamethoxam. Bees from the combined treatment showed mortality rates similar to the V. destructor treatment. However, summer body mass of combined V. destructor and thiamethoxam exposed bees was lower compared to all other treatments. Pesticide treatments alone had no significant influence when compared to the controls; however, V. destructor groups showed reduced body mass measures significantly lower than control and pesticide but not as severe as in the combined treatment. In contrast, Fall 2014 bees exposed to both mites and thiamethoxam showed reduced longevity when compared to all other treatments from summer and fall. Furthermore, body mass from both V. destructor and V. destructor-pesticide treatment were lower compared to the same summer treatments. An identical experimental approach was performed after overwintering of the colonies in Spring 2015 (~37 weeks post pesticide exposure). The spring observations strongly mirrored results observed during Summer 2014. Our data suggest previously overlooked time-lag effects of combined pesticide-mite exposure that could be important to honey bee colony overwinter survival.

Aquatische Ökotoxikologie

57. Mechanistic and systemic understanding of the toxicity of manufactured nanomaterials in Chlamydomonas reinhdardtii

Pillai Smitha, Eawag

Alena Tierbach, Renata Behra, Kristin Schirmer

smitha.pillai@eawag.ch

The rapid development of nanotechnology lead to a rapid proliferation of nanomaterial use in consumer products. They in turn are likely to become a source of different nanoparticles in the environment. Currently, a general lack of information on the ecotoxicology of engineered nanoparticles (NP) prevents an accurate evaluation of their environmental risks. The way forward is to adopt a multidisciplinary approach combining chemical, biological and particle-analysis expertise to estimate the toxicity of any particle specific effects in aquatic organisms. In our study we aim to obtain a mechanistic and systemic understanding of the toxicity of manufactured nanomaterials (MNMs) in the freshwater alga, Chlamydomonas reinhdardtii. For the acute and chronic toxicity analysis several endpoints including growth, oxidative stress and damage, energy and photosynthesis was estimated. We found, not surprisingly, that most of toxicity of silver and zinc nanomaterials is caused due to dissociation of metal ions. The surface modifications due to aging and other subsequent treatment alters the toxicity and bioavailability will also be studied. In addition, we intend to use an integrated systems biology approach, including transcriptomics, proteomics and metabolomics to discover mechanisms of toxicity at the molecular level.

58. Toxicokinetics and internal distribution of cocaine in zebrafish larvae: unexpected accumulation and retention in the eyes

Kirla Krishna Tulasi, 1,2

Ksenia J. Groh2,3, Andrea E. Steuer1, Michael Poetzsch1, Julita Stadnicka2,5, Rik I.L. Eggen2,4, Kristin Schirmer2,4,5, Thomas Kraemer1 1 University of Zurich, Zurich Institute of Forensic Medicine, Department of Forensic Pharmacology and Toxicology, Zurich, 8057, Switzerland 2 Eawag, Swiss Federal Institute of Aquatic Science and Technology, Department of Environmental Toxicology, Dubendorf, 8600, Switzerland 3 ETHZ, Department of Chemistry and Applied Biosciences, Zurich, 8093, Switzerland 4 ETHZ, Institute of Biogeochemistry and Pollutant Dynamics, Zurich, 8092, Switzerland 5 EPFL, School of Architecture, Civil and Environmental Engineering, Lausanne, 1015, Switzerland

krishnatulasi.kirla@eawag.ch

Illicit psychoactive drugs released into aquatic environment as a result of human consumption and incomplete degradation during wastewater treatment, may be potentially harmful to fish due to conservation of major drug targets across vertebrates. However, little is known not only about the effects but also about the internal accumulation and distribution patterns of such compounds following waterborne exposures. Here, we studied the toxicokinetics of cocaine, a widely used illicit drug, frequently detected in surface waters. Temporal profiles of uptake and excretion following waterborne cocaine exposure were characterized in zebrafish at 5-8 days post fertilization (dpf). External and internal concentrations of cocaine and major metabolites were measured by liquid chromatography-tandem mass spectrometry (LC-MS/MS). Internal drug distribution was studied by matrix-assisted laser desorption ionization mass spectrometry imaging (MALDI-MSI), for the first time optimized for use in zebrafish larvae. Our results demonstrated the uptake and biotransformation of cocaine in zebrafish larvae. Elimination occurred after transfer into clean water, however, a plateau was quickly reached, with about one third of initial concentrations still retained after 48 hrs of depuration. MALDI-MSI showed cocaine accumulation in the trunk and in the head region: more precisely, in the brain, but also, surprisingly, in the eyes. In dissected brain, eyes and trunk, cocaine was detected in all the measured tissues, with a highest concentration found in the eyes. After 48 hrs of depuration, most of the remaining cocaine was found in the eyes, while concentrations in the brain and trunk became very low. Our results show that, following waterborne exposure, cocaine is distributed not only to the target organ - brain, but also to the eyes, where it remains for a long time. Perhaps this is one of the reasons that only hypoactive but not hyperactive responses to cocaine have been observed by us and others in behavioural assays with zebrafish larvae. Further studies are needed to evaluate the potential influence of drug accumulation in the eyes on fish vision and behaviour, but also to understand whether the observed distribution patterns are specific to cocaine or could be a more general mechanism relevant for other pharmaceuticals as well.

59. Einfluss unterschiedlicher Futtermengen auf die Reproduktion und den Energiestoffwechsel von Potamopyrgus antipodarum unter chronischer Schadstoffexposition

Brettschneider Denise, Goethe-Universität Frankfurt
Cornelia Geiß; Katharina Ruppert; Jörg Oehlmann;

Brettschneider@bio.uni-frankfurt.de

In den letzten Jahrzehnten hat die Zahl organischer und anorganischer Schadstoffe in der Umwelt stetig zugenommen. Obwohl Mollusken besonders sensitiv auf toxische Substanzen und Endokrine Disruptoren reagieren, sind bisher kaum Standardtests mit Vertretern dieses artenreichen Taxons für die Chemikalienprüfung etabliert. Deshalb wurden vom Umweltbundesamt Dessau zwei Projekte zur Validierung eines Mollusken-Reproduktionstests (ValMol-Repro I und II) mit Potamopyrgus antipodarum gefördert, für die eine standardisierte Prüfrichtlinie entwickelt werden soll. Zur Optimierung des Testdesigns wurde ein möglicher Einfluss verschiedener Futtermengen auf die Reproduktionsleistung der Schnecken unter Substanzexposition untersucht und diejenige Futtermenge identifiziert, bei der reproduktionstoxische und reproduktionsfördernde Effekte abgebildet werden können. Dazu wurden Cadmium als reproduktionstoxische Substanz und Triclocarban als potentiell reproduktionsfördernde Chemikalie in vierwöchigen Reproduktionstests mit P. antipodarum eingesetzt, bei denen ein Viertel der Standarddosis (62,5 µg/Schnecke und Tag), die Standarddosis (250 µg/Schnecke und Tag) oder die doppelte Standarddosis TetraPhyll® (500 µg/Schnecke und Tag) gefüttert wurden. Neben der Mortalität und Reproduktionsleistung wurden die Protein-, Glykogen- und Lipidgehalte der Schnecken als Endpunkte ermittelt, um den Einfluss der Futterdosis und der Schadstoffexposition auf den Energiestoffwechsel zu untersuchen.

Die Reproduktionsleistung der Kontrolltiere nahm mit steigender Futterdosis signifikant zu und führte bei TCC-exponierten Schnecken bei der doppelten Standarddosis TetraPhyll® zu einer Maskierung reproduktionssteigernder Effekte. In den Cd-Versuchen wurde der reproduktionstoxische Effekt nicht durch die Futterdosis beeinflusst, die Effektkonzentrationen lagen bei allen Futtermengen in ähnlichen Konzentrationsbereichen (EC10: 3,35 - 3,49 µg Cd/L, EC50: 8,80 - 12,0 µg Cd/L). Die Energiegehalte der Schnecken nahmen mit steigender Cd-Konzentration und Futterdosis signifikant ab. Mit steigender TCC-Konzentration, aber unabhängig von der Reproduktionsleistung, wurde eine signifikante Zunahme der Lipidgehalte ermittelt (LOEC: <0,04 - 0,05 µg TCC/L). Dies deutet darauf hin, dass TCC als Obesogen wirken und zur Lipidanreicherung führen könnte. Da die Schnecken der Kontrollgruppe, die ein Viertel der Standarddosis TetraPhyll® erhielt, die geringste Reproduktionssteigerung im Vergleich zur Zucht aufwiesen und sowohl reproduktionstoxische als auch reproduktionssteigernde Effekte abbilden konnten, ist diese Futterdosis die geeignetste für den Reproduktionstest mit P. antipodarum.

Danksagung – Die Autoren danken dem UBA für die Finanzierung (FKZ 370861402 und 371165417).

60. In vitro assessment of immunotoxic potentials of chemicals for rainbow trout (Oncorhynchus mykiss)

Rehberger Kristina, Centre for Fish and Wildlife Health, University of Bern

Inge Werner Oekotoxzentrum Eawag-EPFL, Dübendorf and Lausanne Helmut Segner Centre for Fish and Wildlife Health, University of Bern

helmut.segner@vetsuisse.unibe.ch

Numerous legacy and emerging contaminants including persistent organic pollutants and pharmaceuticals are (potentially) immunotoxic to fish. It was shown that exposure of fish to immunotoxic contaminants can adversely affect immunocompetence and pathogen resistance, and thereby may indirectly contribute to the increasing occurrence of pathogen-induced fish mortalities. This situation leads to the question how to screen and assess immunotoxic activities of environmental contaminants. Currently, there exists no established inventory for immunotoxicity testing and assessment in ecotoxicology. The present study aims to examine the suitability of in vitro assays using fish immune cells to screen for potentially immunotoxic chemicals. To this end, primary head kidney leukocytes (resting and stimulated) of rainbow trout (Oncorhynchus mykiss) were exposed to non-cytotoxic concentrations of xenobiotics with different modes of action. The panel of test compounds includes narcotic chemicals, specifically acting non-immunotoxic chemicals, and immunotoxic chemicals which act through different receptor pathways. The selection of the endpoints for detection of an immunomodulatory activity of the test compounds was based on a previously conducted literature review, which identified phagocytic activity, oxidative burst activity, and the expression of cytokines to be most frequently measured and responsive in fish. The results of the present study will clarify whether simple in vitro screening assays are able to capture immunotoxic potentials of xenobiotics irrespective of the specific MoA, and thus may be useful for chemical prioritization in ecotoxicological hazard assessment.

Ackowledgement: This study is financially supported by the EU project SOLUTIONS.

Endokrine Disruption

61. Messung estrogener Aktivität von oxidierten Wässern mit dem planar-YES Bioassay - ein Beitrag zur Methodenentwicklung

Schönborn Andreas, Zürcher Hochschule für angewandte Wissenschaften

Pascal Schmid, Zürcher Hochschule für angewandte Wissenschaften Andreas A. Grimmer, Zürcher Hochschule für angewandte Wissenschaften Oliver Köster, Wasserversorgung Zürich Beat Brüschweiler, Bundesamt für Lebensmittelsicherheit und Veterinärwesen

andreas.schoenborn@zhaw.ch

Der planar-YES ist ein Beispiel für die Kopplung von Dünnschicht-Chromatografie (TLC) mit wirkungsbasierter Analytik (WBA). Ziel dieser Entwicklung ist, das "non-target-screening" von Wasserproben zu vereinfachen und in Zukunft eine direkte Kopplung zur chemischen Analytik zu ermöglichen.

Im OxiScreen Projekt wurden Wasserproben unterschiedlicher Herkunft (Oberflächenwasser, Uferinfiltrat, Evian Wasser als Kontrolle), nativ bzw. aufgestockt mit einem Mix von Mikroverunreinigungen je drei verschiedenen Oxidationsverfahren (Chlorierung, Ozonierung, AOP) unterworfen. Die Proben wurden vor und nach der Behandlung mittels SPE aufkonzentriert und chemisch-analytisch sowie mit dem planar-YES untersucht. Ziel war, neben der Charakterisierung der Wässer eine Aussage über die Auswirkung der Oxidierungsmethode anhand der estrogenen Aktivität zu machen.

Anhand der aufgestockten Proben konnte gezeigt werden, dass die Verfahren AOP und Ozonierung estrogene Wirkung stärker reduzieren als Chlorierung. Diese Wirkung war reproduzierbar und quantifizierbar. In den nativen Oberflächenwasserextrakten wurde estrogene Aktivität < 1 ng/l EEQ gefunden, die durch keine der Oxidierungsmethoden entfernt werden konnte, und die leicht höher als der Geräteblindwert lag.

Die TLC-WBA Kopplung erlaubt, Proben sowohl als Ganzes, als auch nach Auftrennung mit dem planar-YES zu testen. Dadurch lassen sich mehr Aussagen über die Proben treffen, als es mit dem klassischen YES auf Mikrotiterplatten möglich ist. Wir konnten auch zeigen, dass die Verarbeitung der rohen Wasserproben ohne SPE mit diesem Testkonzept in Reichweite liegt.

62. Mixture effects of endocrine active chemicals as determined by human cell based in vitro bioassays

Schlotz Nina, Universitätsklinikum Freiburg
Stephan Flemming, Stefan Günther, Evelyn Lamy

nina.schlotz@uniklinik-freiburg.de

Many of the over 800 substances with suspected endocrine activity have been individually assessed in various bioassays. However, environmental or dietary exposure to single endocrine active chemicals (EAC) is rather the exception; the realistic scenario is a constant challenge by numerous EAC simultaneously. Yet, there is a surprising gap in our knowledge on effects of co-exposure to these substances of concern.

Estrogen receptor (ER) activation as well as induction of proliferation in ER positive cells are assumed to be major hallmarks of estrogenic activity. Measured jointly, both endpoints can provide reasonable estimations regarding the disruptive potential of the respective substances. We investigated mixtures of industrial chemicals, pesticides, as well as phytochemicals. Components of the mixtures were chosen due to their known or suspected estrogenic activity, produced quantity and probability of regular exposure.

Using the ER-CALUX® reporter gene assay and a cell proliferation assay based on the human breast cancer cell line T47D expressing both ER α and β we tested for the potential of EAC mixtures to 1) influence ER-mediated gene expression and 2) induce proliferation in this cell line. We then examined possible interactions between chemicals (additivity, synergy, antagonism) employing a mathematical model based on dose-response data obtained for the single chemicals.

This approach highlights benefits and challenges of in vitro bioassays for assessment of endocrine activity and provides insights into mixture effects of EACs in a human context.

63. Effekte endokriner Disruptoren auf die Reproduktion von Potamopyrgus antipodarum

Geiß Cornelia, Goethe-Universität Frankfurt am Main

Ruppert K, Oehlmann J, Goethe-Universität Frankfurt am Main; Holbech H, Kinnberg, KL, Holbech B, Universität Süddänemark, Odense, Dänemark; Teigeler M, Fraunhofer IME; Askem C, Morris S, CEFAS, Lowestoft, Vereinigtes Königreich; Marin, PS, Miguez, C, Universität Aveiro, Aveiro, Portugal; Beiras, R, Universität Vigo, Vigo, Spanien; Kajankari, P, Penttinen, O-P, Universität Helsinki, Lahti, Finnland; Neiman, M, Universität Iowa, Iowa, USA

geiss@bio.uni-frankfurt.de

In der Risikobewertung von Chemikalien spielten Mollusken bislang eher eine untergeordnete Rolle, da standardisierte und international anerkannte Prüfrichtlinien mit Vertretern dieses artenreichen Taxons fehlten. Dabei zeichnen sich Mollusken besonders durch eine hohe Sensitivität gegenüber endokrin wirksamen Chemikalien (z.B. Organozinnverbindungen, 17α-Ethinylestradiol) und potentiell toxisch wirkenden Substanzen (z.B. Cadmium) aus. Aus diesem Grund wurde vom Umweltbundesamt (UBA) und dem britischen Ministerium für Umwelt, Ernährung und Angelegenheiten des ländlichen Raums (DEFRA) die Erstellung eines Detailed Review Paper (DRP) der OECD [1] initiiert, worin die Notwendigkeit eines standardisierten Molluskentests, mögliche Testdesigns und verschiedene Modellorganismen vorgestellt werden. Zu den vorgeschlagenen Testorganismen gehört u.a. die parthenogenetische Neuseeländische Zwergdeckelschnecke Potamopyrgus antipodarum, mit der eine standardisierte Prüfrichtlinie entwickelt werden soll.

In zwei vom UBA geförderten Projekten („ValMolRepro I und II“) war es das Ziel, den 28-tägigen Partial-Life-Cycle Test mit P. antipodarum als OECD-Richtlinienentwurf auszuarbeiten und entsprechend Ringtests zur Validierung dieses Reproduktionstests zu koordinieren.

Vor diesem Hintergrund wurde u.a. eine internationale Validierungsstudie mit acht Partnern durchgeführt, bei der das Fungizid Prochloraz (PCZ) getestet wurde. Hierbei konnten alle teilnehmenden Labore einen konzentrationsabhängigen signifikanten Rückgang der Reproduktion beobachten. Die Ergebnisse zeigten vergleichbare mittlere (± SD) NOEC- (30,5 ± 8,14 µg/L) und LOEC-Werte (75,0 ± 67,3 µg/L). Die gute Reproduzierbarkeit der Ergebnisse wird auch in den ermittelten EC10-Werten deutlich. Hierbei lag die EC10 der einzelnen Labore zwischen 6,04 µg/L und 45,4 µg/L.

Darüber hinaus wurden an der Goethe-Universität die Effekte der endokrin wirksamen Substanzen Triclocarban (TCC) und Triclosan (TCS) auf die Embryonenzahl von P. antipodarum in einem Reproduktionstest untersucht. Die Ergebnisse für TCC resultierten in einer umgekehrt u-förmigen Konzentrations-Wirkungsbeziehung mit einer Stimulation der Reproduktionsleistung bei niedrigen Konzentrationen, gefolgt von einer Hemmung bei höheren Konzentrationen. Bei Exposition gegenüber TCS konnte eine signifikante Steigerung der Embryonenzahl bis zur höchsten eingesetzten Konzentration (6,26 µg/L) beobachtet werden.

Die durchgeführten Validierungsstudien unterstreichen die Robustheit und die Reproduzierbarkeit des Reproduktionstests mit P. antipodarum. Die ermittelten Effektkonzentrationen für PCZ sind in Übereinstimmung mit bereits publizierten Toxizitätsdaten für andere Invertebra-

ten. Zudem reagiert die Schnecke sensitiv gegenüber endokrin wirksamen Chemikalien, wie TCC und TCS.

[1] OECD (2010): Detailed review paper on molluscs life-cycle toxicity testing. Series on Testing and Assessment, no. 121. ENV/JM/MONO(2010)9, Paris, France.

Danksagung – Die Autoren danken dem Umweltbundesamt für die Finanzierung (FKZ 370861402 und 371165417).

Mikroplastik in der aquatischen Umwelt

64. Mikrokunststoffe in der Umwelt: Ergebnisse einer umfassenden Literaturstudie zu Eintragsquellen, Umweltkonzentrationen, Aufnahme und Effekten in Organismen

Duis Karen, ECT Oekotoxikologie GmbH

Anja Coors, ECT Oekotoxikologie GmbH

k-duis@ect.de

Aufgrund der weitverbreiteten Verwendung und der Stabilität von Kunststoffen überrascht es nicht, dass Makro- und in den letzten Jahren verstärkt auch Mikrokunststoffe (Mikroplastik) weltweit in der Umwelt nachgewiesen wurden. Mikrokunststoffe gelangen aus einer Vielzahl von Eintragsquellen in die Umwelt. Quantitative Aussagen zur Relevanz verschiedener Quellen fehlen bisher weitgehend. Erste Abschätzungen deuten darauf hin, dass in der Umwelt vorliegende Mikrokunststoffpartikel vor allem durch Abrieb und Fragmentierung aus größeren Kunststoffteilen bzw. synthetische Polymere enthaltenden Materialien entstehen. Kunststoffe werden in der Umwelt extrem langsam abgebaut und können über weite Strecken transportiert werden. Das Vorkommen von Mikrokunststoffen wurde v.a. in marinen Lebensräumen untersucht, wo die höchsten Konzentrationen in der Nähe von industriellen und urbanen Zentren und in Konvergenzzonen nachgewiesen wurden. Mikrokunststoffpartikel werden von vielen aquatischen Organismen aufgenommen und meist relativ schnell wieder ausgeschieden. Sie können in der Nahrungskette weitergegeben werden. Bisher gibt es keine Belege für eine Bioakkumulation oder Biomagnifikation. Die Aufnahme von Mikrokunststoffen führt – bei entsprechenden Konzentrationen – v.a. zu einer verringerten Nahrungsaufnahme und infolgedessen zu reduzierten Energiereserven und Auswirkungen auf andere physiologische Funktionen. Basierend auf den ausgewerteten Daten sind die niedrigsten Mikrokunststoffkonzentrationen, die zu einer Beeinträchtigung von über das Wasser exponierten marinen Organismen führten, deutlich höher als im Meerwasser gemessene Konzentrationen. Bei Exposition von Wattwürmern über das Sediment traten Effekte bei einer Konzentration auf, die über den im Meeresboden gefundenen Konzentrationen liegt, aber niedriger ist als die höchsten an der Strandoberfläche nachgewiesenen Konzentrationen. Hydrophobe Schadstoffe reichern sich an Mikrokunststoffpartikeln stark an. Experimentelle Studien und Modellierungen deuten jedoch darauf hin, dass die Relevanz von Mikrokunststoffen als Vektor für den Transport von sorbierten Schadstoffen begrenzt ist. Bevor eine umfassende Bewertung potenzieller durch Mikrokunststoffe verursachter Umweltrisiken durchgeführt werden kann, müssen eine Reihe von Wissenslücken gefüllt werden. So liegen z.B. nur wenige Daten zum Vorkommen von Mikrokunststoffen in limnischen Lebensräumen und zu den Effekten auf Süßwasserorganismen vor. Für terrestrische Lebensräume fehlen diese Daten fast vollständig. Angesichts der Persistenz von Kunststoffen in der Umwelt und der hohen Mikrokunststoffkonzentrationen, die an einigen Standorten gemessen wurden, sollten jedoch schon jetzt auf breiter und globaler Basis Maßnahmen ergriffen werden, um einen weiteren Anstieg der Kunststoffmengen in der Umwelt zu begrenzen.

65. Analytik und Vorkommen von Mikroplastik in Binnengewässern

Storck Florian R., TZW: DVGW-Technologiezentrum Wasser
Heinz-Jürgen Brauch, TZW: DVGW-Technologiezentrum Wasser

florian.storck@tzw.de

Bei Mikroplastik handelt es sich um Fasern oder Partikel aus Kunststoff. Die obere Größengrenze wird meist mit kleiner 5 mm angegeben, eine untere Größe ist bisher nicht definiert. In der Meeresforschung wird Mikroplastik bereits seit einigen Jahren untersucht, in den Binnengewässern steht die Forschung dagegen noch am Anfang. So ist es wenig verwunderlich, dass es bisher weder standardisierte Methoden zur Probenahme, zur Probenaufreinigung und Probenkonzentration noch zum analytischen Nachweis von Mikroplastik gibt. Dieser Beitrag soll den Stand der Technik beleuchten und auf Fallstricke bei Probenahme und Analytik hinweisen.

Für Untersuchungskampagnen werden oft Planktonnetze verwendet, wodurch Partikel kleiner 100 Mikrometer in der Regel nicht erfasst werden können. Filter mit geringerer Maschenweite kommen selten zum Einsatz. Bisher erfolgte meist nur eine oberflächennahe Gewässerbeprobung, was bereits eine systematische Verzerrung bewirken kann. Die Matrix der natürlichen Wasserinhaltsstoffe bereitet schon bei der Probenahme, aber besonders bei der Analytik Probleme, insbesondere, wenn kleine Partikel untersucht werden. Zur Probenaufreinigung werden deshalb Kombinationen verschiedener Wasch- und Aufschlußverfahren angewendet. Der analytische Nachweis und die Identifikation der Polymere kann mit Mikroskopen und gekoppelter Raman- oder Fourier-Transformations-Infrarotspektroskopie erfolgen, wobei man Informationen zu Größenverteilung, Form, Farbe und Material erhält. Eine rein visuelle Einschätzung, ob es sich bei einem Partikel um Mikroplastik handelt, ist wegen der großen Fehleranfälligkeit nicht zu empfehlen, obwohl viele Studien diesen Ansatz wählen.

Mikroplastik hat in Binnengewässern nach gegenwärtigem Wissensstand nur einen geringen Massenanteil (bis 11 mg/Kubikmeter, Partikel 0,5 bis 5 mm, Lechner et al., Environ. Pollut. 2014) verglichen mit natürlichen Wasserinhaltsstoffen (bis 63 mg/L, Rheinüberwachungs-Station Weil am Rhein, Jahresbericht 2013). Nach ersten Einschätzungen werden sich in Gewässern deutlich mehr kleinere als größere Partikel finden. Darauf deuten auch Transportversuche von Plastik in Wasser/Sedimentgemischen hin, die einen Abrieb von bis zu 6 Prozent innerhalb 6 Wochen ergaben. Neben dem Abrieb von Treibgut spielen Einträge aus Kläranlagen für die Gewässerbelastung eine Rolle. So werden vor allem Fasern beim Waschen von Textilien freigesetzt, weniger bedeutsam sind Mikroplastikzusätze zu Kosmetik- und Pflegeprodukten. Weitere Eintragsquellen sind jegliche Plastikmaterialien in Landwirtschaft, Industrie und privaten Haushalten.

Die Autoren danken Dr. Oliver Köster (WVZ), Dr. Oliver Happel, Yanina Müller und Beat Schmutz (TZW).

66. Einfluss der Alterung auf die Freisetzung von Schadstoffen aus Mikroplastik

Bandow Nicole, Bundesanstalt für Materialforschung und Prüfung
Verena Will und Franz-Georg Simon

nicole.bandow@bam.de

Zahlreiche Studien haben gezeigt, dass Mikroplastik in erheblichen Mengen in der Umwelt vorhanden ist und von aquatischen Organismen aufgenommen werden kann. Zur Bestimmung des Risikos, das von diesen Partikel ausgeht, ist es notwendig, das Adsorptions- und Desorptionsverhalten von den im Kunststoff enthaltenen Schadstoffen zu untersuchen.

Ziel dieser Arbeit ist den Einfluss der Alterung von Mikroplastik auf das Freisetzungsverhalten von Schadstoffen zu untersuchen. Dazu wurden drei Recyclinggranulate aus verschiedenen Kunststoffmaterialien ausgewählt: Polyvinylchlorid (PVC), Polyethylen hoher Dichte (HDPE) und Polystyrol (PS). Zur Alterung wurden die Kunstoffe erhöhten Temperaturen (80°C) und UV-Licht bei 80 °C ausgesetzt. Die Hälfte der Proben wurden vor der Alterung mit Ethanol extrahiert, um die Alterung durch die Extraktion von UV-Stabilisatoren und Antioxidantien zu beschleunigen. Nichtgealterte Proben und Proben, die 1000 bzw. 2000 h gealtert worden sind, wurden in Säulenperkolationstests mit destilliertem Wasser eluiert. Eluatproben wurden zu festlegten Wasserfeststoffverhältnissen (W/F) entnommen. In jeder Fraktion wurde der pH-Wert, Leitfähigkeit, Trübung und die Konzentration von gesamten organischen Kohlenstoff (TOC), Anionen ($NO3^-$, Cl^-, F^- und SO_4^{2-}) und Kationen (As, Ba, Ca, Cd, Co, Cr, Cu, Fe, K, Mg, Mn, Mo, Na, Ni, Pb, Si, Sb, Sn, Sr, V, und Zn) bestimmt. Zusätzlich wurde nach Flüssig/Flüssigextraktion ein Screening auf organische Substanzen mit GC-MS durchgeführt. Zur Identifizierung wurden die gemessenen Spektren mit denen aus der NIST-Bibliothek verglichen. Vorläufig identifiziert wurden Substanzen, die in dem Herstellungsprozess verwendet werden oder die als Additive zugegeben werden: z.B. Acetophenon in PS und Diisocyanatomethylbenzol in HDPE. Insgesamt wurden aus den Kunstoffen nur wenige Kationen eluiert: zum einen Alkali und Erdalkalimetalle wie Ca, K, Mg und Na, die häufig als Füllstoffe eingesetzt werden. Die Freisetzung der anderen Kationen ist zum größten Teil sehr gering und liegt oft unter der Nachweisgrenze. Mn, Cu und Co bilden eine Ausnahme: Die freigesetzten Konzentrationen liegen im Bereich von 50 bis 700 µg/L und zeigen einen Trend zu höheren Konzentrationen für das unter UV-Strahlung gealterte Material.

Die Ergebnisse dieser Untersuchung zeigen, dass zumindest für die in dieser Studie ausgewählten Kunststoffe die organischen Substanzen entscheidender sind.

67. Microplastics in Swiss surface waters and going upstream: nature, concentrations, interaction with pollutants

Faure Florian, EPFL

Colin Demars (EPFL GR-CEL) Olivier Wieser (EPFL GR-CEL) Manuel Kunz (BAFU) Luiz Felippe de Alencastro (EPFL GR-CEL)

florian.faure@epfl.ch

Marine microplastic (< 5 mm) water pollution has met growing public and scientific interest in the last years. The situation in freshwater environments remains largely unknown, although they appear to play an important role as part of the origin of marine pollution. Apart from the physical impacts on biota, chemical effects are to be expected as well, especially with smaller particles. This study aimed at assessing the situation for the Swiss Lakes Geneva, Constance, Neuchâtel, Maggiore, Zurich and Brienz, and identifying potential impacts. Lake surface transects, a few rivers as well as urban runoff outlets and WWTPs have been sampled using a floating manta net, and beach sediments have been analysed. Plastics have been sorted in types (fragments, pellets, cosmetic beads, lines, fibres, films, foams) and composition (PP, PE, PS, etc.). Fishes and water birds have been dissected to assess their potential exposure, and analyses of the hydrophobic micropollutants adsorbed to the microplastics as well as some potentially toxic additives they contained have been conducted. It appears that all lakes are affected by this pollution, microplastics of all types and diverse composition having been found in all samples. Birds and fishes are prone to microplastic ingestion, and all the tested chemicals (both adsorbed micropollutants and contained additives) were found above the detection limit, and often the quantification limit. The sources and their respective contribution need to be confirmed and quantified, and the ecotoxicological impacts also need further investigation. Other questions remain open, including the transport and fate of the plastic particles in the environment.

Abwasserbehandlung und -toxizität

68. Testverfahren zur Beurteilung der Behandelbarkeit von Abwasser mit Ozon

Schindler Wildhaber, Y.a,b, Mestankova, H.a, Schärer, M.b, Schirmer, K.a,c,d, Salhi, E.a, Wunderlin, P.e, von Gunten, U.a,c,d

a Eawag - Aquatic research, Überlandstrasse 133, 8600 Dübendorf, Schweiz, b Bundesamt für Umwelt (BAFU), Abteilung Wasser, 3003 Bern, Schweiz, c École polytechnique fédérale de Lausanne (EPFL), 1015 Lausanne, Schweiz, d Eidgenössische Technische Hochschule (ETH) Zürich, 8092 Zürich, Schweiz, e VSA-Plattform „Verfahrenstechnik Mikroverunreinigungen“, Verband Schweiz Abwasser- und Gewässerschutzfachleute (VSA), c/o Eawag, Überlandstrasse 133, 8600 Dübendorf, Schweiz

Stichwörter: Spurenstoffelimination, Ozonung, Testverfahren, Bromat, NDMA, Oxidations- und Reaktionsnebenprodukte

Verschiedene Untersuchungen auf kommunalen Abwasserreinigungsanlagen (ARA) haben gezeigt, dass die Ozonung als zusätzliche Reinigungsstufe zu einer deutlichen Abnahme der im Ablauf der ARA messbaren Mikroverunreinigungen führt. Dadurch lassen sich viele problematische Wirkungen dieser Stoffe im gereinigten Abwasser und in den Gewässern verringern. Bei speziellen Belastungen, wie beispielsweise aufgrund bedeutender Industrie- oder Gewerbeabwassereinleitungen, können durch die Ozonung aber neue Stoffe gebildet werden, die möglicherweise problematisch sind. Daher soll künftig im Einzelfall geprüft werden, ob sich ein bestimmtes Abwassers für eine Behandlung mit Ozon eignet. Dazu wurde ein Testverfahren entwickelt, welches aus fünf Modulen besteht, und in dieser Präsentation vorgestellt wird.

Die ersten vier Module werden im Labor, ein fünftes Modul kann im Pilotmassstab vor Ort durchgeführt werden. Nachfolgend wird ein kurzer Überblick über die einzelnen Module gegeben:

Modul 1: Die Auswirkungen der Abwassermatrix auf die Ozonstabilität werden untersucht, indem die Ozon (O3)– und Hydroxylradikal (•OH)– Expositionen bei einer bestimmten Ozondosis gemessen und mit Referenzabwässern verglichen werden. Dadurch ergeben sich erste Hinweise auf die Behandelbarkeit eines Abwassers mit Ozon, sowie auf unbekannte Inhaltsstoffe, welche allenfalls unerwünschte Transformationsprodukte bilden könnten (siehe Modul 3 und 4).

Modul 2: In diesem Modul werden die Eliminationsraten von ausgewählten Mikroverunreinigungen gemessen, und die ermittelten Raten mit modellierten Werten verglichen.

Modul 3: Mit diesen Untersuchungen wird die Bildung der bekannten unerwünschten Oxidationsnebenprodukte Bromat und NDMA (N-Nitrosodimethylamin) bestimmt.

Modul 4: Mit chemischen Untersuchungen können nicht alle möglichen Transformationsprodukte erfasst werden. Daher wird hier eine Reihe von ausgewählten Biotests durchgeführt, um eine breite Spannweite an möglichen toxischen Wirkungen zu erfassen.

Modul 5: Wenn auf Grund der Resultate aus den Modulen 1 bis 4 keine klare Aussage gemacht werden kann, ob das untersuchte Abwasser für eine Ozonung geeignet ist oder nicht, kann eine Pilotierung vor Ort mit gezielten ökotoxikologischen Begleituntersuchungen weiterführende Informationen liefern.

Die Module 1 bis 4 wurden mit sieben verschiedenen Abwässern an der Eawag getestet. Die Resultate aus diesen Untersuchungen werden in der Präsentation erläutert und diskutiert. Im Rahmen dieser Studie wurde das Modul 5 nicht explizit durchgeführt. Es wird empfohlen, das hier vorgestellte Testverfahren unbedingt vor der Planung und anschliessenden Realisierung einer Ozonung auf ARA durchzuführen.

Quellen

Schindler Wildhaber, Y., Mestankova, H., Schärer, M., Schirmer, K., Salhi, E., von Gunten, U. (2015). Novel test procedure to evaluate the treatability of wastewater with ozone. Water Research 75, 324-335.

Wunderlin, P., Mestankova, H., Salhi, E., Schindler Wildhaber, Y., Schärer, M., Schirmer, K., von Gunten U. (2015). Behandelbarkeit von Abwasser mit Ozon – Testverfahren zur Beurteilung. Aqua & Gas, No.7/8, 28-38.

69. Erste Schweizer Kläranlage mit Ozonung: Verbleib von Spurenstoffen und Entstehung von Transformationsprodukten

Teichler Rebekka, Eawag

Marc Boehler*, Ewa Borowska*, Marc B. Bourgin*, Julian Fleiner*, Juliane Hollender*, Max Schachtler**, Michael Thomann***, Urs von Gunten*, Hansruedi Siegrist*, Christa S. McArdell*; *Eawag, Das Wasserforschungs-Institut des ETH Bereichs, CH-8600 Dübendorf, Schweiz; **ARA Neugut, CH-8600 Dübendorf, Schweiz; ***Holinger AG, CH-4410 Liestal, Schweiz

rebekka.teichler@eawag.ch

Die Abwasserreinigungsanlage (ARA) Neugut ist die erste Schweizer Kläranlage mit erweiterter Behandlung von Abwasser mit Ozon, um auch Mikroverunreinigungen zu entfernen. Die Anlage läuft seit März 2014 störungsfrei. Die ARA hat eine Kapazität von 150’000 Einwohnergleichwerten mit 105‘000 angeschlossenen Einwohnern. Sie reinigt täglich 20’000 – 50’000 m^3 Abwasser mit einer Vorklärung, einer biologischen Behandlung mit Nitrifikation und Denitrifikation, einer biologischen P-Eliminierung und einem Sandfilter, wobei diesem die Ozonung vorgeschaltet wurde. Die Investitionskosten beliefen sich auf 3.3 Mio CHF (3.2 Mio Euro), und mit einem zusätzlichen Energiebedarf von 0.03 kWh/m^3 liegen die Betriebskosten bei 0.023 CHF/m^3. Die Eawag hat die Installation der Ozonung wissenschaftlich begleitet und seit der Inbetriebnahme laufen einige Untersuchungen auf der Anlage. Einerseits wurde die Effektivität der neuen Anlage gemäss den neuen Vorgaben der Gewässerschutzversorgung beurteilt, indem die Elimination der zwölf vorgeschlagenen Leitsubstanzen bei unterschiedlichen Ozondosierungen untersucht wurde. Die 12 Substanzen beinhalten acht, die sehr gut (>80%) mit Ozon oder Aktivkohle eliminiert werden (Amisulprid, Carbamazepin, Citalopram, Clarithromycin, Diclofenac, Hydrochlorothiazid, Metoprolol, Venlafaxin), und vier, die gut (50-80%) eliminiert werden (Benzotriazol, Candesartan, Irbesartan, Mecoprop). Auch die Bildung von Nebenprodukten der Ozonung wie NDMA und Bromat wurde untersucht. In der Präsentation werden Informationen zum neuen Schweizer Gewässerschutzgesetz gegeben und die Resultate der Untersuchungen präsentiert. Es zeigte sich, dass eine Ozondosierung von 2.0 - 3.3 mg/L (0.55 g Ozon /g DOC) für diese Anlage mit relativ tiefem DOC (3.5 - 6 mg/L) ein empfehlenswerter Richtwert ist, um die durchschnittliche Elimination der Leitsubstanzen von >80% gut zu erreichen, auch bei seltenen Spitzenkonzentrationen von Nitrit. Weiter wurden in Laborversuchen ausgewählte Stoffe nach deren Verhalten in der Ozonung untersucht, indem kinetische Studien zu deren Abbau gemacht, und mit hochauflösender LC/MS/MS die entstehenden Transformationsprodukte bestimmt wurden. Es gelang, einige Transformationsprodukte zu identifizieren und quantifizieren. Einige wurden auch in der volltechnischen ARA Neugut gefunden. Im weiteren wurden verschiedene Nachbehandlungen nach der Ozonung (Sandfiltration, Wirbelbett, Festbett, granulierte Aktivkohlefilter) untersucht und miteinander verglichen, um deren Eignung zur weiteren Elimination von Mikroverunreinigungen und der gefundenen Transformationsprodukte zu evaluieren. Die Studie wurde von der EU innerhalb FP7 (DEMEAU project no. 308339), BAFU (UV und ReTREAT Projekte) und SCIEX (Projekt MICROZO no. 12.333) finanziert.

70. Erste Schweizer Kläranlage mit grosstechnischer Ozonung: Ökotoxikologische Untersuchungen zur Beurteilung der Ozonung und verschiedener Nachbehandlungen

Kienle Cornelia, Schweizerisches Zentrum für Angewandte Ökotoxikologie Eawag-EPFL, Dübendorf, Schweiz

Harrie Besselink, Stephan Fischer, Sini Flückiger, Barbara Ganser, Sereina Gut, Sina Hasler, Andrea Schifferli, Sergio Santiago, Kristin Schirmer, Lisa Schlüter-Vorberg, Christina Thiemann, Rita Triebskorn, Bart van der Burg, Etienne Vermeirssen, Mirco Weil, Inge Werner

cornelia.kienle@oekotoxzentrum.ch

Mikroverunreinigungen im Abwasser, beispielsweise aus Rückständen von Medikamenten und Pflegeprodukten, werden durch erweiterte Abwasserbehandlungen reduziert, wodurch der Eintrag dieser Substanzen in Gewässerökosysteme verringert und Trinkwasserressourcen geschützt werden. Frühere Studien haben gezeigt, dass die Ozonung eine vielversprechende Behandlung darstellt, auch wenn reaktive und möglicherweise toxische Umwandlungsprodukte entstehen können. Um Transformationsprodukte, die biologisch abbaubar sind oder an Partikel adsorbieren, aus dem Abwasser zu entfernen, können Nachbehandlungen wie Sandfilter, granulierte Aktivkohlefilter, Wirbel- und Festbett-Behandlungen im Anschluss an die Ozonung eingesetzt werden. Eine der Ozonung nachgeschaltete Stufe mit biologischer Aktivität wird auch vom Schweizerischen Bundesamt für Umwelt (BAFU) empfohlen.

Die Abwasserreinigungsanlage (ARA) Neugut ist die erste Schweizer Kläranlage mit weitergehender Behandlung von Abwasser mit Ozon im großtechnischen Maßstab. In der vorliegenden Studie wurden die Effizienz von Ozonung und verschiedenen Nachbehandlungen zur Reduzierung von ökotoxikologischen Wirkungen im biologisch gereinigten Abwasser untersucht. Ebenso wurde die Effizienz der Nachbehandlungen für die Entfernung von möglichen ökotoxikologischen Auswirkungen von Umwandlungsprodukten aus der Ozonung beurteilt. Folgende Nachbehandlungen wurden bewertet: Sandfilter (großtechnisch), granulierte Aktivkohlefilter sowie Wirbel- und Festbett (jeweils in Pilotanlagen). Ökotoxikologische Effekte wurden mit einer Reihe von Biotests im Labor und auf der Kläranlage beurteilt: Mit in vitro-Testsystemen wurden Gentoxizität, Mutagenität und hormonelle Wirkungen erfasst. Des Weiteren wurden Effekte auf Bakterien, Algen und Wasserflöhe in in vivo-Biotests im Labor untersucht. Auswirkungen auf die Fortpflanzung von Glanzwürmern (Lumbriculus variegatus) und die Entwicklung von frühen Lebensstadien von Regenbogenforellen (Oncorhynchus mykiss) über 28 (Glanzwurm) bzw. 96 Tage (Regenbogenforelle) wurden in Durchflussversuchen auf der ARA evaluiert. Bei den Fischen wurden neben allgemeinen Entwicklungsparametern wie Schlupf, Aufschwimmen und Länge bzw. Gewicht, auch die Veränderungen von Geweben und Organen und die Expression verschiedener schadstoffrelevanter Gene untersucht.

Die Abwasserbehandlung mit Ozon verringerte im biologisch gereinigten Abwasser vorhandene ökotoxikologische Effekte deutlich. Hierzu zählten beispielsweise eine Verbesserung des Wachstum und der Photosynthese von Grünalgen, ebenso wie eine verringerte Toxizität

auf Leuchtbakterien und eine verringerte östrogene Aktivität. Die Nachbehandlungen bewirkten teilweise eine weitere Verringerung der Effekte. Einzig die Fortpflanzung von Wasserflöhen und Glanzwürmern war im Vergleich zu unbelastetem Kontrollwasser in keinem der untersuchten Abwässer beeinträchtigt.

Die Studie wurde vom Bundesamt für Umwelt (ReTREAT Projekt) und von der EU innerhalb FP7 (DEMEAU project no. 308339) finanziert.

71. Advanced wastewater treatment and its contribution to ecosystem health: Long-term and short-term effects of powdered activated carbon filtering in effluent-influenced surface waters

Triebskorn Rita, Universität Tübingen

Rita Triebskorn1, Diana Maier1, Anja Henneberg1, Paul Thellmann1, Katharina Peschke1 and Karl Wurm2 1Animal Physiological Ecology, University of Tübingen, Auf der Morgenstelle 5, 72076 Tübingen, Germany 2 Gewässerökologisches Labor, Tulpenstraße 4, 72181 Starzach, Germany

rita.triebskorn@uni-tuebingen.de

The efficiency of wastewater treatment plant (WWTP) upgrading with powdered activated carbon (PAC) filters for ecosystem health was investigated at three rivers in Southern Germany, the Schussen, the Schmiecha and the Eyach (tributaries of Lake Constance, Danube and Neckar). At the Schussen, the WWTP Langwiese, Ravensburg has been expanded by such a filter in autumn 2013. At the two other rivers, the WWTPs Albstadt-Lautlingen and Albstadt-Ebingen were equipped with PAC-filters more than 20 years ago.

The benefits of the WWTP upgrade for the ecosystem of the Schussen, which have been in the focus of the BMBF-funded project SchussenAktivplus, have recently become obvious: In mean, 80% of micropollutants and related effect potentials and more than three orders of magnitude of (resistant) microbes can be spared to the river system and finally also be kept away from Lake Constance which is an important drinking water reservoir. Although up to now, advantages for the ecosystem of the Schussen could be investigated only for a time span of 1 year, thus only on a short-term run, some positive implications were already visible: Fish health was evidently improved reflected by a better tissue integrity, less genotoxic effects and lower activity of biotransformation enzymes. Also the health status of invertebrates and community indices already showed signs of improvement.

Long-term effects of activated carbon filters were studied at the two other river systems. 20 years ago, before the connected WWTPs were upgraded with PAC filters, these rivers were highly polluted by chemicals from textile industry and rarely any organism could be found there. After the upgrade with PAC, the quality of the receiving water courses has continuously improved. In 2014, we investigated (1) tissue integrity, stress proteins and micronuclei in erythrocytes of resident trout, (2) developmental toxicity, (3) fish population structure, and (4) the integrity of the invertebrate community. The results clearly indicate a healthy status of fish, invertebrates and their communities both upstream and downstream of the two WWTPs, and reflect the high efficiency of the PAC filters for effluent cleaning and, thus, for ecosystem integrity on a long-term run.

72. Efficiency of advanced wastewater treatment technologies – Do we need a further treatment step?

Giebner Sabrina, Goethe-University Frankfurt am Main

Sina Ostermann, Susanne Straskraba, Matthias Oetken, Jörg Oehlmann, Martin Wagner

Giebner@bio.uni-frankfurt.de

Conventional wastewater treatment plants (WWTPs) have a limited capacity to eliminate micropollutants like pesticides, pharmaceuticals and endocrine disrupting chemicals. Hence, wastewater is one of the major sources of micropollutants in aquatic ecosystems. One solution to increase the removal of these substances is the installation of additional treatment steps. Advanced technologies are based on oxidative (e.g. ozonation) or adsorptive treatment (e.g. activated carbon). The WWTP Eriskirch at the river Schussen in Baden-Württemberg is a medium-sized plant that has been upgraded with a combination of ozonation, sand filter and granulated activated carbon filter in a partial flow of the conventionally treated effluent. The efficiency of the applied technologies is assessed by means of chemical analyses and a wide range of ecotoxicological effect studies.

Endocrine effects present in the influent and effluents of WWTP Eriskirch were assessed by in vitro tests which include yeast-based reporter gene assays (estrogenicity, anti-estrogenicity, androgenicity, anti-androgenicity), but also by in vivo studies, such as the reproduction test with the mudsnail Potamopyrgus antipodarum. Genotoxic effects in the samples were investigated with the Ames fluctuation test using the bacterium Salmonella thyphimurium.

The results indicate that the influent and effluents of the WWTP exhibit endocrine and toxic activities in vitro and in vivo. Estrogenic and anti-androgenic activities were relatively high in the influent and were significantly reduced after the conventional treatment. The advanced technologies further decreased the estrogenic activity by 45-80%, but remaining concentrations of estrogens still exceed environmental quality standards and hence might be environmentally relevant. Furthermore, we observed persistent anti-estrogenic activity in the conventional treatment that was not removed by the advanced technologies. In the reproduction test with P. antipodarum the number of embryos was significantly decreased in all effluents compared to the negative control. This might be due to reproductive toxicants which were not eliminated by the advanced technologies. The Ames test on mutagenicity revealed an increase of mutagenicity after ozonation compared to the conventional treatment. Sand filtration as post treatment step reduced this ozone induced mutagenicity. These results highlight the importance of a filter system as post treatment to an ozonation step to minimize toxic effects from potentially oxidation byproducts.

The investigations are part of the project SchussenAktivplus, funded by the Federal Ministry for Education and Research (BMBF) under project code 02WRS1281J.

73. Process performance of a PAC-UF system for the removal of micropollutants from a biologically treated municipal wastewater

Svojitka Jan , Fachhochschule Nordwestschweiz

Jonas Löwenberg, Dominique Ritty, Armin Zenker, Harrie Besselink, Bart van der Burg, Andrea Schifferli, Cornelia Kienle, Thomas Wintgens

jan.svojitka@fhnw.ch

Adsorption on powdered activated carbon (PAC) is an efficient and flexible method for the re-moval of micropollutants from wastewaters. In the present study, tertiary treatment of effluent from a municipal wastewater treatment plant (WWTP) by PAC sorption was tested in combina-tion with an ultrafiltration membrane, to separate PAC from the water (PAC-UF).

A pilot system consisting of two identical membrane filtration units (each had a tank volume of 150 L) was used, so that the results from the two units could be compared. The pilot sys-tem was supplied with wastewater from a full-scale WWTP with continuous dosing of PAC and was operated for a period of approx. 14 months. The subsequent ultrafiltration step consisted of flat-sheet submerged ceramic membrane modules (0.8 m^2 each unit).

During different phases of operation, several operation parameters were modified and their influence on the performance of the filtration unit was evaluated. Modifications of the opera-tion parameters comprised: aeration intensity for the sparging of the membrane surface, filtration flux, frequency of chemically enhanced backwash (CEB), the CEB protocol or the addition of $FeCl_3$ as a coagulant. While the aeration and filtration flux had a strong influence on the fouling of the membranes, the addition of $FeCl_3$ did not considerably improve the filtration performance.

In several measurement campaigns the samples of influent and effluents from both filtration units were analyzed for selected micropollutants (benzotriazole, sulfamethoxazole, car-bamaze-pine, mecoprop, diclofenac) in order to assess their removal in the PAC-UF system under a given set of operational parameters. The highest removal efficiencies were observed for ben-zotriazole and carbamazepine, the lowest ones for sulfamethoxazole.

One measurement campaign was focused on the assessment of ecotoxicological effects of the effluents from the PAC-UF units. Effects on the luminescence of bacteria (Aliivibrio fisch-eri), on the photosynthesis and growth of single-celled green algae (Pseudokirchnerielle subcapitata) were assessed as well as hormonal effects in receptor activation assays (Yeast Estrogen Screen and CALUX® assays) and neurotoxic effects in an acetylcholine esterase inhibition assay.

The study was carried out in the European FP7 project Demeau (http://demeau-fp7.eu/).

This research has received funding from the European Union’s Seventh Framework Pro-gramme under the grant agreement no. 308339.

74. Enzymatic degradation of antibiotics in wastewater – A comparison between chemical analysis and ecotoxicological evaluation

Becker Dennis, Department Aquatic Ecotoxicology, Goethe University Frankfurt, Germany

Rob Schoevaart, Chiralvision BV, Leiden, Netherlands; Jean De Gunzburg, Olivier Couillerot, DaVolterra, Paris, France; Sara Rodríguez-Mozaz, Marta Llorca, Saulo Varela, Catalan Institute for Water Research (ICRA), Girona, Spain; Damià Barceló, Water and Soil Quality Research Group, Barcelona, Spain; Matthias de Cazes, José Sanchez-Marcano, Marie-Pierre Belleville, Institut Européen des Membranes (IEM), Montpellier, France; Jörg Oehlmann, Martin Wagner, Department Aquatic Ecotoxicology, Goethe University Frankfurt, Germany

dennis.becker@bio.uni-frankfurt.de

Micropollutants are continuously released into the environment in low concentrations. Concerns have been raised regarding their toxicity and their limited degradation during conventional wastewater treatment.

Among these pollutants, antibiotics are frequently present in treated wastewaters and may pose an important public health issue in the near future, especially regarding the distribution of resistant bacteria. The treatment with an enzymatic approach could be a promising alternative or addition to existing technologies by using specifically designed enzymes degrading antibiotics and other micropollutants. So far, most studies evaluated their removal by chemical analysis only, but especially regarding transformation products (TP) an ecotoxicological assessment is equally important.

In this study osmosed water samples spiked with antibiotics and real wastewater samples were treated with a fungal laccase and analysed by chemical analysis and ecotoxicological assessment. The latter was based on a growth inhibition test with Bacillus subtilis for the detection of antibiotic activity.

We investigated the enzymatic degradation of a single antibiotic, tetracycline, with special interest in the formation of generated TPs. In addition, we also investigated the degradation of a mixture of 38 antibiotics comprising seven antibiotic families. These experiments were conducted in osmosed water. In addition, hospital wastewater samples were enzymatically treated, where apart from spiked tetracycline also 11 other antibiotics were detected.

Chemical analysis revealed that tetracycline and also the antibiotics in the mixture were successfully removed with an effectiveness of 70-100%. Moreover, the identified TPs of tetracycline did not induce a response in the ecotoxicological assays. However, the removal rates for the antibiotic mix detected by chemical analysis could not be explained by the reduction of the corresponding antibiotic activity by ecotoxicological evaluation, this was maybe due to generated TPs. Regarding the elimination of antibiotics in the wastewater samples, the removal was not as effective as observed in osmosed water. The antibiotic activity and toxicity even increased after enzymatic treatment.

These findings demonstrate the significance to combine chemical analysis with ecotoxicological evaluation. A removal of a compound does not necessarily translate into the reduction of

toxicity. This is especially important when evaluating a novel treatment technology like this enzymatic approach.

Acknowledgement – “This project has received funding from the European Union’s Seventh Framework Programme for research, technological development and demonstration under grant agreement No 282818”

Poster

Ökotoxikologie von Bauchemikalien

P1. Beschreibung der Auswaschverläufe von Bioziden aus bewitterten Materialien anhand eines Regressionsmodells

Schoknecht Ute, Bundesanstalt für Materialforschung und -prüfung (BAM), Berlin

H. Mathies (BAM); R. Wegner, MPA Eberswalde, Materialprüfanstalt Brandenburg GmbH, Eberswalde; S. Uhlig, H. Baldauf, QuoData GmbH, Dresden

ute.schoknecht@bam.de

Die Anwendung mathematischer Verfahren zur Interpretation von Daten aus Leachingstudien war Bestandteil eines vom Umweltbundesamt in Dessau geförderten Forschungsvorhabens.

In Freilandversuchen mit einer biozidhaltigen Farbe (Deckanstrich, Produkttyp 7 nach EU Biozidverordnung) und einem imprägnierten Textil wurde die Auswaschung von bioziden Wirkstoffen an bis zu drei verschiedenen Standorten zu verschiedenen Zeiten beobachtet. Dazu wurden Wirkstoffgehalte im ablaufenden Regenwasser bestimmt. Ein Vergleich kumulativer Emissionen mit Bezug auf das von den Testflächen ablaufende Wasservolumen ergab Kurvenverläufe, die für die verschiedenen Wirkstoffe in der untersuchten Farbe bzw. dem Textil unterschiedlich gut übereinstimmen. Verglichen wurden auch die Wirkstoffmengen, die in den ersten 10 l/m² Ablaufwasser gefunden wurden. Dieses Volumen repräsentiert etwa 300 l/m² Regen. Für Versuche an den unterschiedlichen Standorten lagen die Standardabweichungen der freigesetzten Wirkstoffmengen für die untersuchten Wirkstoffe zwischen 4 und 33 %.

Offensichtlich verursachen unter anderem die tatsächlich gegebenen Witterungsbedingungen die beobachteten Unterschiede. Ein Regressionsmodell, das neben Versuchsparametern auch meteorologische Daten für die Versuchszeiträume an den jeweiligen Standorten berücksichtigt, erlaubt eine gute Beschreibung der Emissionsverläufe. Dabei ist das Ablaufwasservolumen der dominierende Faktor. Weitere wichtige Faktoren sind die Menge des Schlagregens, Globalstrahlung, Temperatur und relative Luftfeuchte.

P2. Modellierung der Auswaschung und Umweltexposition von Bauinhaltsstoffen

Hochstrasser Florian, HSR Hochschule für Technik, Rapperswil, Schweiz

Dirk Engelke, Olaf Tietje, Michael Burkhardt

florian.hochstrasser@hsr.ch

Aus Bauprodukten, die den Umwelteinflüssen ausgesetzt sind, werden potenziell schädliche organische und anorganische Stoffe (z. B. Biozide, Metalle, PAK, Weichmacher) ausgewaschen und in die Umwelt emittiert. Für die Zulassung von Bauprodukten, die Risikoabschätzung von problematischen Stoffen wie auch eine vorausschauende Bewirtschaftung von Niederschlagswasser in bebauten Gebieten wird die Bedeutung von modellgestützten Simulationen in Europa im Rahmen von CPR, BPR oder REACH in den kommenden Jahren stark zunehmen.

Das Modell COMLEAM (Construction Materials Leaching Model) ist in der Lage, aus Eingabedaten zu Wetter, Geometrie (z. B. Gebäudehöhe, Fläche, Exposition) und Auswaschungsdaten, die in Feld- und Laborversuchen ermittelt wurden, die Emission aus beregneten vertikalen und horizontalen Bauteilen zu berechnen. Als treibende Kraft für die Auswaschung an vertikalen Bauteilen, z. B. Fassaden, dient der Schlagregen. Der resultierende Abfluss wird pro Bauteil (z. B. Dach, Fassade) in einem Schnittstellenkompartiment bilanziert und den Umweltkompartimenten Boden, Grundwasser und Oberflächengewässer zugeführt. An festgelegten Orten der Beurteilung (point of compliance) wird das Risiko für Boden und Gewässer beurteilt. Gegenwärtig wird das Modell in Java realisiert und soll zukünftig über eine Benutzerschnittstelle im Browser als Webapplikation bereitstehen. Der Rechenkern und die Datenbank liegen auf einem zentralen Server.

Unter Berücksichtigung von Baumaterialien, Flächenanteilen pro Expositionsrichtung, Auswaschfunktionen, realen Siedlungsstrukturen und Gebäudetypen mit Hilfe von GIS-Systemen werden unterschiedliche Szenarien evaluiert und getestet. So wird es möglich sein, typische Siedlungsszenarien zu modellieren und eine mögliche Gewässerbelastung sowie Risiken abzuschätzen.

Das Modell COMLEAM soll darüber hinaus in einem Modellkonzept integriert werden, welches mit der Gruppe ESTIMATE, in der Partner aus Deutschland und den Niederlanden mitwirken (Fraunhofer IME Schmallenberg, BAM Berlin, RWTH Aachen, ECN Petten, RMI Ober-Ramstadt), erarbeitet wird. Dadurch sollen Schnittstellen zwischen Modellen zur Auswaschung und zur Umweltrisikobeurteilung besser genutzt werden.

Erste Resultate von COMLEAM werden an der SETAC-GLB vorgestellt.

P3. Aquatic exposures to chemical mixtures: an urban typology Fate and Effects of Chemicals from Diffuse Sources and Stormwater

Galay Burgos Malyka, ECETOC

Derek Muir (Environment Canada), Thomas Parkerton (Exxonmobil), William Adams (Rio Tinto), Karel De Schamphelaere (Ghent University), Juliane Hollender (Eawag), Marion Junghans (Schweizerisches Zentrum für angewandte Ökotoxikologie Eawag-EPFL), Graham Merrington (WCA Environment), Noriyuki Suzuki (Japanese National Institute for Environmental Studies), Richard Williams (UK Centre for Ecology & Hydrology), Graham Whale (Shell Health Services), Dick de Zwart (RIVM)

malyka.galay-burgos@ecetoc.org

Urban regions are the fastest growing areas globally. By 2030 it is estimated that nearly 60% of the world population will be in urban areas (United Nations 2014). Urban areas have historically been located along water bodies that support economic development through transportation of goods via shipping. Infrastructure for collection and treatment of industrial and domestic wastewater is expanding but generally is outstripped by urban growth (Corcoran et al 2010). The growing area of impervious surfaces of the urban environment can represent a direct source of contaminants to surface waters during storm events unless storm-water management infrastructure such as catch basins and retention ponds is present (Marsalek et al 2007).

Urban storm-water runoff has been described as presenting one of the greatest challenges to ensuring clean water for human and ecological health (National Research Council 2008). Storm water overflows from combined sewer system, particularly in older cities and incomplete sewerage infrastructure in urban areas provide additional sources. The urban scenario also has to consider non treated or partially treated point sources from industrial origin, substances used for material protection (e.g. biocides in facade paint), external use by households (e.g. domestic pesticides, fertilizers) as well as diffuse inputs from dry and wet atmospheric deposition. A preliminary assessment of contaminants reported in urban storm waters and combined overflows shows that metals (Cu, Zn, Al, Fe, V, Pb), phosphorus, insecticides, and PAHs are chemicals of concern. As a consequence of these various sources, biota present in water catchments subject to increasing urbanization will be exposed to a diffuse mixture of chemical stressors.

This poster, which is an output of the SETAC Pellston workshop on “Simplifying environmental mixtures: An aquatic exposure-based approach via exposure scenarios” focuses on the urban discharge scenario that includes runoff from impervious surfaces, sewer overflows and industrial inputs.

Our aim is to develop an urban typology or typologies that can be used to both help understand the ecological response of urban rivers and simplify the risk assessment process for evaluating chemicals comprising this mixture. This requires detailed information on the chemicals of concern (concentrations, typical emission rates from various surfaces etc.) as well as on urban hydrology.

P4. Ecotoxicological assessment of immersion samples from façade render

Vermeirssen Etienne, Schweizerisches Zentrum für Angewandte Ökotoxikologie Eawag-EPFL, Dübendorf, Schweiz

Conrad Dietschweiler; Sophie Campiche; Marion Junghans; Andrea Schifferli; Cornelia Kienle; Inge Werner; Michael Burkhardt

etienne.vermeirssen@oekotoxzentrum.ch

To protect façade renders from growth of bacteria, fungi and algae, biocides can be added to a render before it is applied onto a façade. These biocides will gradually leach out and thus have the potential to affect soil or aquatic ecosystems. In this study the leaching behaviour of biocides from three render formulations was evaluated: one render containing free, another render containing encapsulated biocides (Terbutryn, OIT, DCOIT) and a control render without biocides. The renders were applied onto extruded polystyrene panels and water samples were generated over nine immersion cycles of the panels. Concentrations of the biocides were measured and the toxicity of the first and ninth immersion samples was determined using bioassays. Toxicity to aquatic organisms was evaluated by assessing inhibition of photosynthesis and algal growth rate, inhibition of bacterial luminescence and inhibition of daphnid population growth. Toxicity to soil organism was assessed by determining avoidance behaviour of worms and reproductive output in springtails. Encapsulation reduced the leaching of Terbutryn, OIT, and DCOIT 4-, 17-, and 25-fold compared to free biocides. Generally, the toxicity of water from render containing encapsulated biocides was always lower than that of render with free biocides and toxicity was 4- to 5-fold lower for the ninth immersion day compared to the first immersion day sample for both free and encapsulated samples. For the aquatic organisms, inhibition of photosynthesis was the most sensitive endpoint, followed by algal growth rate, bacterial bioluminescence and daphnid reproduction. At all tested sample concentrations, none of the samples with biocides caused effects on soil organisms. Results from bioassays matched quite well with expected bioassay responses based on chemical analysis and the toxicity of the individual biocides. It could be concluded, that the toxicity of given concentrations on algae is explained by Terbutryn whereas the toxicity on bacteria and daphnids is caused by DCOIT and OIT. The results thus indicated that other components in the render did not add to the toxicity of the individual biocides. Overall, the approach combining a standard leaching test with standard bioassays is very promising to evaluate the ecotoxicity of biocides leached out from façade renders.

Biotest-Normungsverfahren

P5. Effect-based tools for monitoring (xeno)estrogens in surface waters: Variability and reproducibility of sample preparation and 5 different in vitro assays

Kunz Petra Y., Swiss Centre for Applied Ecotoxicology Eawag-EPFL, Dübendorf, Switzerland (current address: Federal Office for the Environment FOEN, Air Pollution Control and Chemicals Division, Biocides and Plant Protection Products Section, Berne, Switzerland)

Selim Aït-Aïssa, Nicolas Creusot, Nadzeya Homazava, B. Sumith Jayasinghe, Cornelia Kienle, Sibylle Maletz, Andrea Schifferli, Christine Schönlau, Nancy D. Denslow, Henner Hollert and Inge Werner

petra.kunz@bafu.admin.ch

In vitro bioassays are increasingly used to assess estrogenic activity of environmental water samples, and have been suggested as suitable tools for monitoring estrogenic contamination of surface waters. Such assays are of particular use as they measure the overall estrogenic activity of a sample, including the potent steroids 17β-estradiol (E2) and 17α-ethinyl estradiol (EE2). Monitoring of E2 and EE2 under the WFD watch-list mechanism is currently an analytical challenge as proposed annual average Environmental Quality Standards (AA-EQS) of 400 and 35 pg/L, respectively are below limits of quantification for most routine analytical methods. Sensitive in vitro assays could circumvent current detection problems by measuring the overall estrogenic activity of all (xeno)estrogens present in an environmental sample.

It has been shown, however, that different in vitro assays lead to different E2-equivalent (EEQ) concentration for the same sample. Reasons for these differences are known, but it remains unclear how to use and interpret bioassay results. The aims of this study were (1) to compare the variability and reproducibility of EEQs measured in five in vitro assays (YES, ER-Calux, MELN, T47D-KBluc and GeneBlazER), including sample preparation (SPE) and, to (2) evaluate their applicability as effect-based tools for environmental monitoring.

Our data shows that ERTAs are comparable to chemical measurements regarding variability and repeatability of the derived EEQ concentrations. In the different ER-TAs overall variability ranged between 6 and 17 % for highly polluted samples and, 7 to 27 % for low polluted samples spiked with a concentration of (xeno-)estrogens below the AA-EQS for E2 of 0.04 ng/L. In all cases, recoveries were between 72 and 125 %.

ERTAs are promising tools to screen for estrogenic activity in environmental water samples. Within the watch list mechanism of the WFD such a screening step, preceding the analytical monitoring will reduce monitoring costs and provide valuable information on water quality.

P6. Comparison of data analysis strategies to derive 17β-estradiol equivalent concentrations in in vitro bioassays

Simon Eszter, Schweizerisches Zentrum für Angewandte Oekotoxicologie EA-WAG-EPFL, Dübendorf, Schweiz

Petra Kunz, Cornelia Kienle, Inge Werner, Etienne Vermeirssen

eszter.simon@oekotoxzentrum.ch

In vitro assays used in ecotoxicology typically generate sigmoidal dose-response curves when a reference compound is applied in increasing concentration. These dose-response curves are used to derive equivalent concentrations for environmental samples. However, curves generated for environmental samples are often not parallel to that of the reference, or do not achieve minima or maxima values that are similar to the reference. This may be due to low expected activity of the sample, sample solubility problems or cytotoxicity. Low activity may be expected for surface and/or drinking waters. When this occurs, the type of data analysis conducted will influence the equivalent concentrations derived. This makes the comparison of studies conducted in different laboratories challenging. Furthermore, there is a need for harmonized data analysis to translate bioassay data from science to regulation.

In this study, data of a previous study were re-analyzed. The original data, which compared 17β-estradiol equivalents (EEQs) of reconstituted water samples and extracts assessed by five commonly used in vitro bioassays, were calculated by interpolating the response of the sample into the dose-response curve of the reference compound (17β-estradiol). Data points from the lower linear region of the sample dose-response curve, but still above the assay limit of detection (LOD), were considered for EEQ calculations.

We now used the raw data and determined EEQs using the “PC10 approach”. First, all data were normalized, where 0% refers to the response in the solvent control and 100% is the maximum response fitted for the reference dose-response curve. Second, the 10% effect level of the sample was interpolated from the reference dose-response curve.

Results did not differ much between the two data analysis strategies as could be expected – in both cases data points from the lower part of the sample dose-response curves were evaluated. One additional influencing factor on the EEQ concentration derivation might be the fact that data were generated from mixtures of standard compounds and not from more complex environmental samples. However, it still has to be evaluated if this played a role in the outcome. To assess this further, additional calculations and graphical examples with real-life water samples will be shown. The “PC10 strategy” appears to circumvent the problems described above regarding the derivation of equivalent concentrations, and to be a more robust and objective method for in vitro test data analysis.

P7. Standardised ecotoxicological tests for the soil compartment: applications and future developments

Campiche Sophie, Swiss Centre for Applied Ecotoxicology Eawag/EPFL

Etienne Vermeirssen, Inge Werner, Benoit J.D. Ferrari Swiss Centre for Applied Ecotoxicology Eawag/EPFL, 8600 Dübendorf, Switzerland E-mail contact: sophie.campiche@centreecotox.ch

sophie.campiche@centreecotox.ch

Currently, more than 40 internationally agreed (i.e. ISO or OECD) test methods are available for assessing soil quality or effects of chemicals on the soil fauna. Among the invertebrates used to identify potentially toxic chemicals in soil, the compost worm Eisenia fetida is certainly the oldest representative, as described in the OECD guideline 222 on earthworm acute toxicity from 1984. Since then, the number of standardised methods using soil organisms from different trophic levels or evaluating diverse endpoints have increased steadily. Within the aim of developing an integrated approach for the evaluation of Swiss soil quality, the Ecotox Centre is building a tool box based on a battery of practice-oriented toxicity tests. We will present suitable and relevant ISO or OECD ecotoxicological methods that have already been considered and applied in various projects. We will also present promising tools that are under development or that are considered for standardisation.

Sedimentökotoxikologie

P8. Ein neues Testsystem für Gewässersedimente

Brinke Alexandra, Bundesanstalt für Gewässerkunde
Georg Reifferscheid, Roland Klein, Sebastian Buchinger, Ute Feiler

buchinger@bafg.de

Als funktionelle und strukturelle Elemente erfüllen Pflanzen eine Schlüsselrolle in aquatischen Systemen. Trotzdem gibt es nur wenige ökotoxikologische Testsysteme - im Speziellen Sedimentkontakttest - mit Sedimentkontakttest. Aufgrund großer Unterschiede bezüglich der Aufnahmepfade und Sensitivitäten zwischen den einzelnen Taxa, ist die Entwicklung neuer Testsysteme mit Makrophyten für eine holistische Bewertung des Gewässer- und Sedimentzustands jedoch wünschenswert. In dieser Studie präsentieren wir einen neuen Ansatz zur Bewertung der Sedimentqualität limnischer Gewässer mit dem Testorganismus Oryza sativa.

Reispflanzen wurde auf Arsen, Cadmium, Chrom und Nickel dotierten artifiziellen, sowie auf kontaminierten natürlichen Sedimenten exponiert. Wurzel- und Sprosswachstum konnten als sensitivste Endpunkte. Auf artifiziellen Sedimenten zeigte sich des Wurzelwachstum als sensitiver gegenüber dem Sprosswachstum, während sich das Sprosswachstum als der sensitivere Endpunkt auf natürlichen Sedimenten erwies.

Der Test orientiert sich bezüglich seiner Testparameter am Sedimentkontakttest mit Myriophyllum aquaticum nach ISO 16191 und kann daher ohne großen Mehraufwand als Ergänzung des etablierten Testsystems eingesetzt werden. Ein informativer Mehrwert ergibt sich durch die taxonomischen Unterschiede zwischen beiden Pflanzen. So handelt es sich bei der Reispflanze um eine monokotyle Pflanze, weshalb physiologische Unterschiede und damit Unterschiede in der Sensitivität zu kontaminierten Sedimenten gegenüber dem dikotylen Makrophyten M. aquaticum zu erwarten sind. Um den Zugewinn an Informationen bezüglich Sedimenttoxizität zu validieren, wurden EC50-Werte aus Expositionen auf artifiziellen Sedimenten verglichen, sowie vergleichende Expositionen auf natürlichen Sedimenten der beiden genannten Testorganismen durchgeführt.

P9. A new module for the sediment compartment within the Modular Stepwise Procedure (MSK) for the assessment of rivers and streams in Switzerland: the ModSed project

Casado-Martinez Carmen, Oekotoxzentrum Eawag-EPFL
B.J.D. Ferrari, I. Werner

carmen.casado@centreecotox.ch

Sediments are an ecologically important compartment of surface waters. According to the Swiss Water Protection Ordinance [1], sediments must not accumulate persistent pollutants to ensure the protection of aquatic life. Although a national program for the monitoring of sediment quality such as the one implemented for soils (NABO) is absent, existing data illustrate that sediment contamination is present in Switzerland and there are cases where it is of concern for the health of aquatic ecosystems. Despite this, to date no harmonised guidelines/methods or quality standards are available for sediment-quality monitoring.

According to a questionnaire sent to cantonal agencies, the availability of adequate sediment quality guidelines for selected chemicals to classify in situ sediments according to their ecotoxicological risk is a top need [2]. Of second priority is the harmonisation of sampling strategies, including the preparation of samples for further analyses.

In response to the needs of cantons and other stakeholders, the Ecotox Centre together with Eawag and Bafu, started working on a new module “sediment” within the framework of the Modular Stepwise Procedure (“MSK”) used in Switzerland for surface water quality monitoring. The module is conceived to aid cantonal and private agencies in conducting sediment quality assessments in a harmonised regulatory framework and support the cantonal water protection institutions to fulfil their obligations regarding Swiss water protection laws. The module “sediment” is planned to complement existing MSK modules and thereby to achieve a more holistic assessment of Swiss surface waters by incorporating the sediment compartment.

The first phase of the project deals with the development of a sediment assessment system based on the chemical status and will address the two top priorities for cantons: 1) a harmonised protocol for sediment sampling and pre-treatment and 2) numerical sediment quality guidelines for selected contaminants and a related quality classification system for selected compounds. The output should be a technical guidance document with a validated and calibrated methodology ready for use by cantonal and private labs.

References

[1] Office Fédéral de la Protection de l’Environnement, 1998. Ordonnance du 28 octobre 1998 sur la protection des eaux (OEaux). No. RS 814.201. Berne, Suisse.

[2] Flück R, Chèvre N, Campiche S. 2011. Surveillance de la qualité des sédiments en Suisse : Synthèse d’un questionnaire. Septembre 2011, Centre Ecotox, Lausanne, Suisse.

P10. Bioavailability and toxicity of sediment-bound contaminants (PCBs and metals) using an integrative approach: case study of the Venoge river (Switzerland)

Molano-Leno Lidia, Oekotoxzentrum Eawag-EPFL

M.C. Casado-Martinez(1), N. Estoppey(2), L.F. De Allencasto(2), I. Werner(1), B.J.D. Ferrari(1)

(1) Oekotoxzentrum Eawag-EPFL, Switzerland (2) Central Environmental Laboratory, Ecole Polytechnique Fédérale de Lausanne (EPFL), Switzerland

carmen.casado@centreecotox.ch

The Venoge is a small river flowing into the Lake Geneva, the largest water body of Switzerland. Previous monitoring campaigns have shown increased concentrations of PCBs in fishes, which were reported to exceed maximal levels permitted by the European Union for human consumption. The quality of the surface waters of the river are well characterized, both in terms of chemical contamination and benthic community structure. However, no information of sediment quality was available so far. We have used an integrated methodology that includes sediment physico-chemical measurements, the ecotoxicological effects of collected sediments using a battery of sediment toxicity tests, and the bioaccumulation of contaminants to benthic invertebrates to assess the contribution of sediments to the overall quality of the water body.

Sediments were collected at several sampling sites along the Venoge and they were analyzed for PCBs and metals concentrations as well as other ancillary parameters important in determining contaminant bioavailability. The battery of tests used for the characterization of the ecotoxicological properties of sediments included several standard sediment-contact tests using different species and endpoints: the growth inhibition test using the aquatic macrophyte Myriophyllum aquaticum (ISO 16191); the growth and mortality test using the ostracod Heterocypris incongruens (ISO 14371); the emergence test using midge larvae of the species Chironomus riparius (AFNOR 90-339) and the survival and growth test using the amphipod Hyalella azteca (ISO 16303). Bioaccumulation was also assessed in midge larvae under laboratory and field conditions.

The chemical characterization classified the sediments as having intermediate levels of contamination, with concentrations increasing at downstream sampling sites. The toxicity tests showed variable toxicity depending on the test organism and the test endpoint, although the highest toxicity levels were also observed at the site closer to the river mouth. Bioaccumulated concentrations in midge larvae showed direct uptake of PCBs from sediments to this benthic invertebrate. The results obtained in this case study are integrated to assess whether sediments are contributing to the impairment of the beneficial uses of this aquatic ecosystem and identify which tools from those included within the proposed approach provide useful information within a decision-making framework.

P11. The influence of multiwalled carbon nanotubes on the toxicity and bioaccumulation of triclocarban in Lumbriculus variegatus

Bick Vanessa, RWTH

Hennig, Michael Patrick Schäffer, Andreas Maes, Hanna Maja

vanessa_jung@arcor.de

The rising amount of plastic waste being released to the aquatic environment is of great concern due to their diverse effects on living organisms. Especially multiwalled carbon nanotubes (MWCNT), which are contained in several plastic products and can be released to the aquatic environment in the process of weathering, may offer sorption sites for highly sorptive substances like triclocarban (TCC). As a consequence of sedimentation this TCC-MWCNT-sorbate might be available to sediment dwelling organisms and have influence on the toxicity and bioaccumulation. As TCC is exclusively employed as a bacteriostat in bar soaps, the major route for TCC disposal is through domestic sewage and it has already been detected in streams and municipal biosolids as well as in sediments.

This study focuses on the acute toxicity of TCC in the presence of MWCNT on Lumbriculus variegatus. The toxicity tests (14 days) follow the OECD 225 and take five concentration (0.5 mg/kg dw – 8 mg/kg dw and 0.4 mg/kg dw – 2 mg/kg dw) of TCC into account while the concentration of MWCNT is fixed (1 mg/l). The toxicity of TCC itself is tested as a reference to illustrate the effect of MWCNT on the worms. First results show a NOEC lower than 0,5 mg/kg dw. Subsequently, a bioaccumulation study shows the ingestion and metabolism of 14C-TCC in the presence of MWCNT in Lumbriculus variegatus by analysing the radioactivity in the worm tissue at different time points during a 14-day exposition. These studies are accompanied by sorption studies according to the OECD 106 to determine the distribution coefficient (Kd) and sorption isotherm as means to explain the effect of MWCNT as a sorption site for TCC in the sediment water system. For all studies quartz sand is used as sediment to illustrate the effect of MWCT as the main sorbent.

It is to be expected that TCC adsorbs stronger to MWCNT containing sediment. Hence, the distribution coefficient is higher compared to a scenario without MWCNT. As a consequence TCC might be more bioavailable for Lumbriculus variegatus and finally leads to a higher bioaccumulation and possibly a higher toxicity.

P12. Zeit- und Konzentrationsabhängigkeit von Verfügbarkeit und Effekten dioxinähnlicher Schadstoffe in Regenbogenforellen (Oncorhynchus mykiss) während der Resuspension von Sedimenten

Brinkmann Markus, RWTH Aachen University

Kathrin Eichbaum, Mathias Reininghaus, Sven Koglin, Ulrike Kammann, Lisa Baumann, Helmut Segner, Markus Zennegg, Sebastian Buchinger, Georg Reifferscheid, Henner Hollert

markus.brinkmann@bio5.rwth-aachen.de

In den vergangenen Jahrzehnten haben dioxinähnliche Verbindungen (DLCs) wie polychlorierte Dibenzo-p-Dioxine und Dibenzofurane (PCDD/F), sowie einige polychlorierte Biphenyle (PCB) und polyaromatische Kohlenwasserstoffe (PAK), großes Interesse in der wissenschaftlichen Gemeinschaft erfahren. DLCs können sich aufgrund ihrer physikalisch-chemischen Eigenschaften in Sedimenten, aber auch in Wasserorganismen (z.B. in Fischen) und entlang der Nahrungskette anreichern. Sie verursachen eine Reihe akuter und chronischer Schadeffekte, die größtenteils durch ihre Bindung an den Arylhydrocarbonrezeptor vermittelt werden. DLCs stellen somit ein beträchtliches Risiko für die Tierwelt, aber auch für die menschliche Gesundheit dar. Bis heute ist jedoch nicht vollständig geklärt, ob und in welchem Umfang sedimentgebundene DLCs, z.B. bei Hochwasserereignissen, direkt für Fische bioverfügbar sind.

Um die zeit- und konzentrationsabhängige Aufnahme und damit verbundene Effekte von DLCs zu untersuchen, wurden Expositionsexperimente mit Suspensionen drei unterschiedlich stark belasteter nativer Sedimente aus Rhein und Elbe durchgeführt. Von den beiden kontaminierten Sedimenten aus Prossen und der Zollelbe in Magdeburg (beide Elbe, Deutschland) wurden jeweils fünf Verdünnungen mit einem mäßig kontaminierten Sediment aus Ehrenbreitstein (Rhein, Koblenz, Deutschland) hergestellt und in Suspension gebracht. Juvenile Regenbogenforellen (Oncorhynchus mykiss) wurden mit diesen Verdünnungen, sowie den unverdünnten Sedimenten, unter semistatischen Bedingungen für 90 Tage exponiert.

Die Bioverfügbarkeit partikelgebundener PCDD/F, PCB und PAK während der Resuspension der Sedimente konnte anhand instrumenteller chemischer Analysen von Muskelgewebe und Gallenflüssigkeit der exponierten Tiere nachgewiesen werden. Die Aufnahme der Substanzen und die damit verbundenen Effekte in den Forellen waren meist proportional zu ihrer Konzentration in den Sedimentverdünnungen. Die Reaktionen der untersuchten Biomarker reflektierten dabei den unterschiedlichen Kontaminationsgrad der Sedimente: die cyp1a (Cytochrom P450 1A) mRNA-Expression und die Aktivität der 7-Ethoxyresorufin-O-Deethylase (EROD) in der Leber der Fische reagierte rasch und mit hoher Empfindlichkeit, während eine erhöhte Anzahl von Mikrokernen und anderen Kernaberrationen, sowie histopathologischen Läsionen zu einem späteren Zeitpunkt auftraten und eine starke Indikation für potenzielle Langzeitwirkungen darstellen.

Die Ergebnisse zeigen deutlich, dass PCDD/F, PCB und PAK während der Resuspension von Sedimenten eine erhöhte Bioverfügbarkeit zeigen können, was darüber hinaus zu potenziell nachteiligen toxikologischen Wirkungen führen kann. Für eine solide Risikobewertung im Kontext der Europäischen Wasserrahmenrichtlinie und der damit verbundenen Rechtsvorschriften und Bewirtschaftungspläne ist ein starker Fokus auf sedimentgebundene Schadstoffe daher unerlässlich.

Aquatische Ökotoxikologie

P13. Auswirkungen von Metamitron auf das Wachstum der Wasserlinse Lemna gibba und die Grünalge Pseudokirchneriella subcapitata

Janz Patricia, Goethe-Universität Frankfurt am Main
Katharina Heye, Matthias Oetken, Jörg Oehlmann

p_janz@stud.uni-frankfurt.de

Pflanzenschutzmittel werden weltweit angewendet und können durch unbeabsichtigten Eintrag in Gewässer zu einer Belastung von aquatischen Ökosystemen führen. Ein intensiv verwendetes Herbizid im Rübenanbau ist Metamitron. Es gehört zur Gruppe der Triazinone und hemmt die Photosyntheseleistung von Pflanzen über die Blockierung des Photosystems II. Somit stellt es eine potentielle Gefährdung für Primärproduzenten dar. Eine solche Beeinflussung der Primärproduzenten kann sich kaskadenartig auf höhere trophische Stufen der Nahrungskette auswirken. Entsprechend spielen photoautotrophe Organismen bei der Testung von Umweltchemikalien eine wichtige Rolle.

Um den Einfluss von Metamitron auf aquatische Primärproduzenten zu untersuchen, wird ein Wachstumshemmtest mit der einzelligen Grünalge Pseudokirchneriella subcapitata durchgeführt. Das Wachstum wird nach 72 Stunden über die Veränderung der optischen Dichte gemessen, worüber die Wachstumsrate und die Effektkonzentrationen ermittelt werden. Das Testprinzip entspricht der OECD-Richtlinie 201.

Neben Algen sind höhere Wasserpflanzen wichtige Primärproduzenten in aquatischen Systemen. Die Toxizität von Metamitron für diese Gruppe wird durch die Stellvertreterart Lemna gibba untersucht. Gemäß der OECD-Richtlinie 221 (Stand 2006) wird der Einfluss auf das Wachstum über die Endpunkte Trockengewicht und Anzahl der Fronds über einen Zeitraum von 7 Tagen ermittelt. Diese Parameter werden verwendet, um die Wachstumsrate von L. gibba zu bestimmen und Effektkonzentrationen zu berechnen.

Die Ergebnisse der laufenden Experimente werden während der Tagung auf einem Poster präsentiert.

Danksagung – Die Untersuchungen werden durch das Bundesministerium für Bildung und Forschung (BMBF) im Rahmen des Projektes „NiddaMan“ unter dem Förderkennzeichen 02WRM1367A gefördert.

P14. Effects of methylmercury on aquatic primary producers: a transcriptomic approach

Beauvais-Flueck Rebecca, University of Geneva
V. I. Slaveykova, C. Cosio

rebecca.flueck@unige.ch

Mercury (Hg) is amongst the most toxic metals with no known biological function. Its properties: volatile, long-range transportable and biomagnified in the aquatic trophic chain have led Hg to become a global environmental issue, irrespective of the presence of local emission sources. Aquatic primary producers, such as phytoplankton and macrophytes, are key players of the biogeochemical cycle of mercury (Hg) in the aquatic environment. Indeed, the Hg bioaccumulation step from water to primary producers has been shown to be the largest increase of Hg concentrations in aquatic ecosystems reaching 104-fold and greater. It is therefore of the upmost importance to understand uptake and effects of Hg in primary producers. The effects of Hg on aquatic primary producers were only sparsely investigated until now. While it has been shown that Hg affects photosynthesis and the activity of oxidative stress enzymes in microalgae, its effects were less studied in macrophytes, especially concerning MeHg. Moreover most studies were conducted at high concentrations only and were thus not representative of environmental conditions. Using a transcriptomic approach (RNA-Seq), we therefore studied the effects at the molecular level of MeHg (5 $x10^{-11}$, 5 $x10^{-10}$, 5 $x10^{-9}$ and 5 $x10^{-8}$ M), in the microalga Chlamydomonas reinhardtii and the macrophyte Elodea nuttallii, adapting bioinformatics analyses to the available genomic information for the studied species. Strong response of the transcripts involved in photosynthesis was observed at all MeHg concentrations in the alga, but only at the two highest concentrations in the macrophyte. Concomitantly, MeHg affected genes involved in carbohydrates, amino acids and lipids metabolisms, as well as the expression of many transcripts coding for transporters. In fact, while ABC and calcium transporters were significantly affected by MeHg in C. reinhardtii, ATPases and ammonium transport were disregulated in E. nuttallii. Finally, metal transporters, e.g. zinc transporters, were significantly affected in both species. The transcriptome response to MeHg in the two representative aquatic primary producers was congruent with their morphological, physiological and sensitivity differences.

P15. Lemna sp. and Daphnia magna in pulsed dose test designs relating to the EFSA guidance document on tiered risk assessment

Eser Sabine, Eurofins Agroscience Services EcoChem GmbH
Claudia Zawadsky, Silke Falk, Guido Gonsior, Nathalie Schwalbach

SabineEser@eurofins.com

In the latest guidance document of the European Food Safety Authority (EFSA Journal 2013;11(7):3290) tiered risk assessment with aquatic organisms for plant protection products is outlined. In edge-of field surface waters time-variable exposure towards plant protection products is common. Therefore it is stated that for risk assessment the predicted exposure regimes may be simulated by pulsed dose tests instead of performing constant exposure as in standardised lower tier tests. To reflect the actual exposure scenario the number and spacing of pulses (toxicological (in)dependence of repeated pulse) is of high importance for an appropriate risk assessment.

Here pulsed dose tests employing macrophytes (OECD 221: Lemna sp. Growth Inhibition Test and OECD 239: Water-sediment Myriophyllum spicatum Toxicity Test) and invertebrates (OECD 211: Daphnia magna Reproduction Test) are presented with the focus on test design and performance.

P16. Algal flow-through test system for assessment of time-variable exposure scenarios of pesticides

Obert-Rauser Patrick, Eurofins Agroscience Services EcoChem GmbH
Ulrich Memmert, Guido Gonsior, Christoph Hafner, Denis Weber

PatrickObert-Rauser@eurofins.com

The algal growth inhibition test according to OECD 201 is routinely used to assess the toxicity of substances as pesticides, biocides etc. on algae. This standard test design with a static exposure period of three days simulates a simple scenario assuming a constant exposure in a static water body. The exposure of organisms towards agrochemicals in the aquatic environment on the other hand usually follows more complex patterns. Generally, agrochemicals are applied seasonally and the entry of them into surface waters via spray drift, run off or drainage therefore is typically time-variable. According to the EFSA “Guidance on tiered risk assessment for plant protection products for aquatic organisms in edge-of-field surface waters” (2013) a more realistic fate can be taken into account during a higher-tier risk assessment of plant protection products. Here we present a single species algal flow-through system which offers the possibility to test the effects of time-variable, e.g. pulse-dosed, concentrations to algal populations including the potential to prove population recovery during a test period of several weeks to months. Thus, the algal flow-through system is a helpful tool to improve the environmental risk assessment of substances like herbicides towards algae by testing more realistic time-variable concentrations.

P17. Lead cromate in aquatic compartments - Acute ecotoxic effects on Algae and Daphnia and Derivation of Predicted No Effect Concentrations (PNEC) in the Context of REACH

Könnecker Gustav, Fraunhofer ITEM

Oliver Licht, Stefan Hahn, Andrea Wenzel, Markus Simon, Thorsten Klawonn, Jürgen Koppenhöfer

gustav.koennecker@item.fraunhofer.de

Enhancement of naturally occurring background concentrations of metals and metal compounds in the environment from anthropogenic sources may lead to raising risks for potentially exposed organisms. This concern is addressed by the European REACH regulation in the methodology described by ECHA in a specific technical guidance document for the chemical safety assessment for metals and metal compounds. A considerable number of metals, among them various lead as well as chromium compounds, have already been evaluated under European regulatory context. Therefore, EU Risk Assessment Reports and REACH dossiers are available, which served as a basis for the derivation of Predicted No Effect Concentrations (PNEC) for relevant environmental compartments. However, as no experimental effect data for lead chromate itself has been published so far, short-term ecotoxicity studies on the freshwater algae Pseudokirchneriella subcapitata and invertebrate Daphnia magna were conducted. Results from these studies were used to assess whether read-across concepts as seen in many REACH dossiers on different chemical compounds, containing the same metal, can be regarded as appropriate.

Due to its very low water solubility, maximum soluble test concentrations of lead chromate had to be prepared according to the principal of “Water Accommodated Fraction” (according to OECD technical guidance) and diluted to final test concentrations.

Lead chromate caused a concentration-dependent inhibition of algal growth with a 72 h ErC50 (specific growth rate) and EyC50 (yield) of 3012 µg and 1291 µg $PbCrO_4$/L, respectively, with NOECs of 528 µg $PbCrO_4$/L for both parameters. The maximum achievable $PbCrO_4$ concentration in the daphnia test (median 167 µg/L) had no effect on immobilisation.

Experimental results correlate very well with other available effect data on the same trophic levels and confirm the assumption, that the ecotoxicity of the tested metal compounds is predominantly caused by the cation. Based on the available effect data, included in risk assessment dossiers prepared under various regulatory context, PNEC values for lead chromate were derived according to the procedure laid down in the ECHA technical guidance documents.

P18. Genotoxicity biotesting strategy integrating cell-based and zebrafish embryo bioassays

Di Paolo Carolina, RWTH Aachen University

Lilli Senn 1, Yvonne Mueller 1, Beat Talmann 1, Sebastian Heger, Henner Hollert 1, Thomas-Benjamin Seiler 1 1 Institute for Environmental Research (Biology V), RWTH Aachen University, Aachen, Germany

carolina.dipaolo@bio5.rwth-aachen.de

The occurrence of genotoxic compounds in aquatic systems can present a serious threat for humans and aquatic organisms. Therefore unexplained genotoxic effects caused by environmental samples present a challenge for Effect Directed Analysis (EDA). As a result, biotesting strategies for genotoxicity assessment can highly benefit future EDA studies. Currently, different bioassays are available to measure and assess genotoxicity. Among eukaryotic systems, the micronucleus test (MN) is a robust method previously applied to different in vitro and in vivo experimental models. Unfortunately the MN is a work-intensive and time-consuming method, characteristics which reduce its applicability in EDA. At the same time, the p53-CALUX assay is a recently developed reporter gene cell-based bioassay that presents great potential for high-throughput screening. Even if previous studies have demonstrated a correlation between the induction of p53 and micronucleus formation, the p53-CALUX has been only limited applied so far.

This study aimed to advance small-scale genotoxicity bioassays and develop a biotesting strategy for genotoxicity assessment in EDA. The micronucleus test was established using two new experimental models: the ZFL zebrafish liver cell line and zebrafish early larvae. Also, the p53-CALUX assay was established in our laboratory. The bioassays were applied to investigate the toxicity of selected chemicals, including the environmental contaminants 3-nitrobenzanthrone, 1-nitropyrene and benzo[a]pyrene; and the positive control chemicals 4-nitroquinoline 1-oxide, cyclophosphamide and actinomycin D. There was also investigation of non-specific toxicity, through the MTT assay for the cell-based systems, and by the assessment of lethal and sublethal morphological endpoints in the zebrafish.

Results from the cell-based bioassays demonstrate the importance of determining the levels of non-specific toxicity that can occur simultaneously to the genotoxicity investigation. For instance, the peaks of p53 induction were observed shortly before or concurrently with reduced cell-viability effects. At the same time, high cytotoxicity levels should be avoided, as those can cause general cell stress that result in p53 induction or even micronucleus formation. Similar approach was followed to investigate MN on the organism level. The levels of non-specific toxicity were considered during the selection of exposure concentrations and also for the interpretation of results, in order to minimize the occurrence of false positive or false negative results. The newly established MN tests were successfully applied to evaluate model compounds; as a result, one in vitro (ZFL) and one organism-level (embryos) zebrafish bioassays are available for future genotoxicity investigations.

P19. Normalentwicklung des Kopfskeletts im Embryo der Dickkopfelritze (Pimephales promelas) und dessen strukturelle Veränderungen nach Belastung mit Cumarin und Warfarin

Mieck Susanna, Universität Heidelberg

Svenja Böhler, Thomas Braunbeck

mieck@stud.uni-heidelberg.de

Die Dickkopfelritze (Pimephales promelas) ist zusammen mit dem Zebrabärbling (Danio rerio) und dem Japanischen Medaka (Oryzias latipes) eine der drei am häufigsten genutzten Test-fischarten der OECD. Trotz der intensiven Nutzung in Toxizitätstests ist jedoch verhältnismä-ßig wenig über die Entwicklung der Dickkopfelritze bekannt.

In dieser Studie wurde die Normalentwicklung der Knorpel- und Knochenstrukturen im Schädel der Dickkopfelritze vom ersten Auftreten bis 144 h dokumentiert. Zur Erkennung der Strukturen im Kopfbereich des heranwachsenden Embryos wurde ein neues Färbeprotokoll basierend auf den Methoden von Inohaya (Medaka Book, Chapter 4.4.1, 2007) und Walker & Kimmel (Biotechnic & Histochemisty 2007, 82(1): 23-28) entwickelt. Bei der Auswertung wurde das erste Auftreten der einzelnen Strukturen mit der Notochordlänge ins Verhältnis gesetzt.

Zur Validierung der neuen Färbemethode wurden Fischembryotests mit Cumarin und dem Cumarin-Derivat Warfarin nach der OECD Test-Guideline 236 durchgeführt, wobei neben Daten zur akuten Toxizität auch teratogene Endpunkte erfasst wurden. Das knorpelige und knöcherne Schädelskelett des Embryos wurde nach der Belastung auf Veränderungen hin untersucht. Auffälligkeiten wurden dokumentiert und nach ihrer Ausprägung und Schwere semiquantitativ ausgewertet.

P20. Effects of pharmaceuticals in aquatic organisms and their relevance for ecosystems – a literature review

Schwarz Simon, Universität Tübingen

Kristin Berg, Ina Ebert, Manfred Frey, Dirk Jungmann, Jörg Oehlmann, Matthias Oetken, Rita Triebskorn

si.schwarz@uni-tuebingen.de

A vast diversity of human and veterinary pharmaceuticals is frequently detected in aquatic ecosys-tems. However, effects of these substances in biota are far from being understood. Since the principle of pharmacovigilance demands the examination of environmental effects, it is crucial to develop new, effect-based strategies for the monitoring of pharmaceuticals.

Within the framework of the project 'EffPharm', we conducted a survey of published literature (2011 to 2013) to gain an overview which pharmaceutical groups appear to be of highest relevance for biota, and which organism groups and effect endpoints react most sensitively to pharmaceuticals. A data-base was created and the studies were evaluated with respect to the reported lowest observed effect concentrations (LOECs), which were subsequently compared to measured environmental concentra-tions (MECs) in surface waters. Additionally, we investigated the reliability of studies reporting LOECs in close range to MECs.

Overall, 452 studies were examined, of which 232 directly investigated effects of pharmaceuticals. The most frequently studied organism group was fish, with Danio rerio being the most commonly used species, followed by molluscs. It was shown that studies on effects of pharmaceuticals are conducted with a huge variety of species. However, only few of them are investigated in more than one study and studies rarely apply native species of high ecological relevance in Central Europe. Considering relevant endpoints, behaviour, vitellogenin synthesis, growth, reproduction and histopathological changes were among the most sensitive responses. Based on the information extracted from these studies, the analgesic diclofenac, the β-blocker propranolol, the lipid regulator gemfibrozil and the antibiotics erythromycin and sulfamethoxazole were identified as environmentally most relevant pharmaceuticals. Unfortunately, the evaluation also showed that only a minor part of the investigated publications can be considered as fully reliable; most studies included at least some minor shortcomings, e.g. the lack of chemical analyses of exposure concentrations. Furthermore, there still appears to be a huge knowledge gap considering mixture and sediment toxicity. The population relevance of many observed effects remains unclear.

For further monitoring strategies we propose a mode of action-driven approach specific for substance classes rather than tests on prioritized substances.

P21. Effects of the beta-blocker metoprolol on the embryonic development and health of brown trout Salmo trutta f. fario

Schmieg Hannah, Universität Tübingen

Simon Schwarz, Marco Scheurer, Heinz R. Köhler, Rita Triebskorn

hannah.schmieg@student.uni-tuebingen.de

The discharge of human and veterinary pharmaceuticals into surface waters via wastewater treat-ment plants is a growing problem in Central European countries. Considering demographic changes, the consumption and environmental concentration of pharmaceuticals used for treating age-related diseases are known to increase. However, despite the frequent detection of various substances in aquatic ecosystems, knowledge on their effects in biota is still scarce. Available data are based on standard biotests with model test organisms which often lack ecological relevance. One of the most frequently used pharmaceuticals and, hence, also frequently detected in surface waters, is the beta-blocker metoprolol. However, only very few effect studies are available. Therefore, further information on the effects of metoprolol on aquatic ecosystems is of particular interest and importance.

Our study investigated the effects of metoprolol on the health status and on the embryonic devel-opment of brown trout, Salmo trutta f. fario, a species of high ecological relevance for Central Eu-rope. The applied concentrations were 0.1, 1, 10, 100, and 1000 µg/L metoprolol, thus including sev-eral concentrations in ranges reported for German surface waters.

Juvenile fish (six month post hatch) were exposed for a time period of 28 days. Besides biometric measurements and mortality, the level of the stress protein Hsp70 was measured as a general marker of proteotoxicity. Furthermore, histology of liver, kidney, gill, and heart was assessed reflecting the overall health condition of the trout. The exposure of trout eggs/embryos was based on OECD 212. Recorded endpoints in these tests included mortality, time until eyes were developed, time to hatch, number of hatched larvae and number of healthy larvae at the end of the test. In addition, the degree of lipid peroxidation in trout hatchlings was determined to assess oxidative stress.

The analysis showed no significant proteotoxic, embryotoxic or hepatotoxic effects. Further histological and biochemical analyses will complement the study.

Overall, our study aims at providing further information on the effects of environmentally relevant concentrations of a frequently used pharmaceutical in a species of high ecological relevance in Ger-many and adjacent countries.

P22. Effect-directed Analysis of a water extract from River Danube near Novi Sad (Serbia)

Seidensticker Sven, Helmholtz Centre for Environmental Research UFZ, Leipzig, Germany; RWTH Aachen University, Institute for Environmental Research (Bio V), Aachen, Germany

Eberhard Küster, Martin Krauss, Tobias Schulze, Muhammed Arslan Kamal Hashmi, Thomas-Benjamin Seiler, Wibke Busch, Werner Brack, Henner Hollert

sven.seidensticker@ufz.de

The protection of freshwater ecosystems is one of today's greatest environmental challenges and therefore, their pollution is an issue of emerging concern. One part of the EU-funded SOLUTIONS project is a case study of River Danube in Novi Sad, Serbia, aiming to investigate how the discharge of untreated wastewater in local water bodies affects the river ecosystem. For adequate water quality assessment and prioritization of pollutants it is important to know which substances are present and which should be particularly monitored. In applying an effect-directed analysis (EDA) the intention is to analyze both the effects occurring in exposed fish embryos (as an alternative to animal tests with fish) and the substances which are responsible for the observed effects. Three on-site large volume solid phase extraction samples were taken from River Danube, near Novi Sad in Serbia (approx. 2 km upstream of a wastewater discharge point, directly behind the discharge point (approx. 200 m), and about 7 km further downstream). To investigate the toxicity of the different samples the 96 h-fish embryo acute toxicity test (OECD 236 FET-test) with Danio rerio embryos was performed with decreasing extract concentrations defined as relative enrichment factors (REF). The first FET-test results showed a significant difference between the sampling sites. The highest LC50 and thus the lowest toxicity were detected at upstream site (REF of 54); the highest toxicity was observed directly behind the discharge point (REF of 4.4). The LC50 of the downstream site was in between the first two (at REF of 30). Sublethal effects such as a strong malformation of the tail and the head of embryos as well as a modified chorda structure were observed especially in lower concentrated samples from the site immediately downstream of the discharge site. Data about fractionation results after stepwise reducing the complexity of samples will be shown. Toxic and teratogenic effects of the fractions identified by the FET-test and contaminants presumably responsible for these effects will be presented.

P23. Physiologisch basierte toxikokinetische Modelle zur In vitro-in vivo-Extrapolation Rezeptor-vermittelter Effekte in Fischen

Brinkmann Markus, RWTH Aachen University

Kathrin Eichbaum, Sebastian Buchinger, Georg Reifferscheid, Thuy Bui, Andreas Schäffer, Henner Hollert, Thomas G. Preuss

markus.brinkmann@bio5.rwth-aachen.de

Die Europäische REACH-Verordnung wurde vom Europäischen Parlament und dem Rat eingerichtet, um negative Auswirkungen von Industriechemikalien auf Mensch und Umwelt prospektiv zu bewerten bzw. zu vermeiden. Um dieses Ziel zu erreichen sind Hersteller und Importeure von Chemikalien verpflichtet, toxikologische und ökotoxikologische Daten bereitzustellen, weshalb prinzipiell eine große Zahl neuer Tierversuche erforderlich ist. Die Europäische Chemikalienagentur (ECHA) ist dabei stets um die Minimierung unnötiger Tierversuche mit Wirbeltieren bemüht – wenn möglich sollen Tierversuche durch experimentelle (z.B. In vitro-Tests) oder nicht-experimentelle (z.B. Grouping- oder Weight-of-Evidence-Ansätze) Alternativmethoden ersetzt werden. Die Daten in vielen der bis 2011 bei der ECHA eingegangenen Dossiers waren jedoch lückenhaft und die Begründungen für die Verwendung von Alternativmethoden oft unzureichend. In vitro-Tests kamen vergleichsweise weniger häufig zum Einsatz. Insbesondere die Aussagekraft von In vitro-Befunden für die Auswirkungen einer Substanz in vivo wird häufig infrage gestellt. Um diese Lücke zu schließen wurde ein physiologisch-basiertes toxikokinetisches (PBTK) Modell verwendet, um wässrige Expositionskonzentrationen in In vivo-Experimenten mit Regenbogenforellen (Oncorhynchus mykiss) in interne Konzentrationen, z.B. in der Leber, umzurechnen. Zu diesem Zweck wurde eine umfassende Datenbank experimenteller In vitro- und In vivo-Daten zur Induktion von EROD (Ethoxyresorufin-O-deethylase) und Vitellogenin (VTG) durch verschiedenen Monosubstanzen zusammengetragen. Es konnte gezeigt werden, dass die mittlere Effektkonzentrationen (EC50-Werte) basierend auf der internen Konzentration in der Leber wesentlich besser mit EC50-Werten aus In vitro-Tests korrelierten als EC50-Werte auf Basis von wässrigen Expositionskonzentrationen. Die vorliegende Studie hat gezeigt, dass PBTK-Modelle zur In vitro-In vivo-Extrapolation wichtiger Biomarker für Mechanismus-spezifische Effekte verwendet werden können. Die Kombination von In vitro-Tests und In silico-Methoden hat daher das Potenzial, eine Gesamtreduktion der für toxikologische und ökotoxikologische Experimente benötigten Wirbeltiere zu erreichen.

P24. Expression of Phase II Biotransformation Enzymes at Early Life Stages of Zebrafish (Danio rerio)

Tierbach Alena, Eawag

Ksenia J Groh (1,2) Kristin Schirmer (1,3,4) Marc J-F Suter (1,3) (1) Eawag, Swiss Federal Institute of Aquatic Science and Technology, 8600 Dübendorf, Switzerland (2) ETH Zürich, Swiss Federal Institute of Technology, Department of Chemistry and Applied Biosciences, 8093 Zürich, Switzerland (3) ETH Zürich, Swiss Federal Institute of Technology, Institute of Biogeochemistry and Pollutant Dynamics, 8092 Zürich, Switzerland (4) EPF Lausanne, School of Architecture, Civil and Environmental Engineering, 1015 Lausanne, Switzerland

alena.tierbach@eawag.ch

Early developmental stages of zebrafish (Danio rerio) are increasingly employed as alternative to the conventional acute fish toxicity test. One important requirement for the establishment of an alternative test model is that the toxicological outcomes can be directly extrapolated to the test system it aims to replace. This implies that both models should be comparable in terms of uptake and biotransformation of xenobiotics. A limited expression of biotransformation enzymes at early developmental stages can lead to an under- or overestimation of the toxicity of compounds which are targets of phase I and II metabolism. However, little is known about the time resolved expression of xenobiotic metabolizing enzymes in zebrafish and thus about the chemical groups which might display different patterns of toxicity within different life stages.

In this study, we have developed a targeted proteomic approach to characterize the expression of glutathione-S-transferases, a group of phase II enzymes, during the zebrafish development. Selected members of the family are monitored in zebrafish embryos by mass spectrometry on the basis of proteotypic peptides and peptides characteristic for the enzyme groups of interest. The results collected in this study will help to establish methods for the detection of further enzymes involved in the phase I and phase II metabolism and fill some of the existing knowledge gaps regarding the comparability of metabolic capacity of fish at different life stages.

P25. Cell –type specific arylhydrocarbon (AhR) receptor signaling in liver and immune cells of rainbow trout.

Möller Anja Maria, Centre for Fish and Wildlife Health, University of Bern

Markus Spiliotis Institute of Parasitology, University of Bern Helmut Segner Centre for Fish and Wildlife Health, University of Bern

helmut.segner@vetsuisse.unibe.ch

The cytosolic transcription factor aryl hydrocarbon receptor (AhR) senses small chemical molecules including important environmental contaminants, such as dioxins, polychlorinated biphenyls and polyaromatic hydrocarbons. Upon activation, the AhR induces via the dioxin-response element to gene transcriptional changes, which are cell-specific and associate with cell-specific toxicity. Hallmarks of AhR-mediated toxicity in vertebrates are hepatotoxicity and immunosuppression. Here, we used primary cultures of rainbow trout immune cells and liver cells in combination with transcriptomics to examine cell-specific differences in the response to toxicant-induced AhR activation. Immune cells were isolated from head kidney and spleen by mechanical disaggregation, and hepatocytes were isolated from the liver by collagenase perfusion The cells were exposed to equipotent concentrations of the AhR ligand, benzo(a)pyrene (BaP). Global gene expression analysis showed that in total, 672 genes were regulated in liver cells, 134 in head kidney cells and 408 in spleen cells. Among the genes that were commonly upregulated in liver, head kidney and spleen, were CYP1A1, CYP1A3 and sulfotransferase. GO-term enrichment analysis of the regulated genes revealed an cell-specific response, with liver cells showing alterations particularly in pathways related to biosynthesis metabolism whereas immune- and homeostasis-related pathways were most affected in head kidney and spleen cells. In conclusion, the findings point to pronounced cell type-specific effects of AhR activation and provide a basis to assess organ-specific toxicities of environmental contaminants.

Ackowledgement: This study is financially supported by the EU project KEYBIOEFFECTS

P26. Thyroid disruption in developing zebrafish (Danio rerio) embryos – effects on eye development and function

Baumann Lisa, Centre for Fish and Wildlife Health, University of Bern

Albert Ros Kristina Rehberger Centre for Fish and Wildlife Health, University of Bern Stephan Neuhauss Insitute for Molecular Life Sciences, University of Zürich, Helmut Segner Centre for Fish and Wildlife Health, University of Bern

helmut.segner@vetsuisse.unibe.ch

Environmental contaminants with endocrine disrupting properties are not only able to disrupt the reproductive system of fish, but also their thyroid system. Comparably little is known about the developmental, physiological and behavioral consequences of thyroid disruption in fish. As the thyroid system plays an important role in the development of craniofacial structures and the eyes, its environmentally induced disruption can impact important fitness-related parameters such as metamorphosis, behavior or visual capacities of the larvae. The present study aims to investigate the relation between initiating molecular events of thyroid disruption and morphological and physiological changes of the embryonic development of zebrafish (Danio rerio) with focus on the eye development and function. Two modes of action were studied: interference with thyroid hormone synthesis with the pharmaceutical Propylthiouracil (PTU), and binding to thyroid hormone receptors with the flame retardant Tetrabromobisphenol A (TBBPA). Changes in gene-expression were analyzed with qRT-PCR and visualized with whole mount in situ hybridization. The influence on visual capacities of the embryos was investigated by measurement of the optokinetic response (OKR). The results provide initial insight if and how chemical interactions with molecular events in the thyroid axis of zebrafish translate into morphological and physiological alterations of the developing embryos.

Acknowledgement: This study is financially supported by German Research Foundation

P27. Xenobiotic defense system in early life stages of rainbow trout (Oncorhynchus mykiss)

Kropf Christian , Centre for Fish and Wildlife Helath, University of Bern; University of Applied Sciences and Arts Northwestern, Muttenz

Helmut Segner Centre for Fish and Wildlife Health, University of Bern Karl Fent University of Applied Sciences and Arts Northwestern, Muttenz Swiss Federal Institute of Technology, ETH Zürich

helmut.segner@vetsuisse.unibe.ch

At present, little is known about the presence, expression and function of biological defense systems in early development stages of teleost fish. Here we analyzed the expression of uptake transporters, phase I and phase II enzymes and ABC transporters during development of eleu-thero-embryos from 1 to 20 days post hatching (dph). The aim was to obtain an overview on the mRNA profiles in different body parts of eleuthero-embryos and to analyze the function of ABC-transporters. The transcript profiles of eight ABC transporter genes (abcb1a, abcb1b, abcc1, abcc2, abcc3, abcc4, abcc5, abccg2), two phase I (cyp1a, cyp3a), three phase II (gstp, putative ugt1, putative ugt2), two uptake transporters (oatp1d, putative oatp2) and an SLC47 efflux transporter (mate7) are described. Transcripts of most phase I and II enzymes and ABC trans-porters showed a variable abundance in different body parts during development. The function of ABC-transporters was determined by use of inhibitor assays. MK571 inhibited the uptake of rhodamine B by the eleuthero-embryos at 1 and 10 dph, while cyclosporin A and reversin205 had little effects. Clotrimazole led to inhibition of rhodamine B uptake at all development stages and in all body parts, whereas clofibric acid showed no effect. In addition, clotrimazole led to changes in some transcript levels. The expression of uptake and efflux transporters, xenobiotic metabolizing enzymes and the functionality of ABC transporters suggest the presence of a defense system in eleuthero-embryos of rainbow trout.

Acknowledgement: This study is financially supported by the Swiss National Science Foundation

P28. Comparative assessment of xenobiotic clearance in primary hepatocytes from cold- and warmwater fish species, rainbow trout and common carp

Bischof Ina, Fraunhofer IME, Schmallenberg

Ralf Böhm MVZ Interdisziplinäres Kinderwunschzentrum, Düsseldorf Monika Hüben Horst Jürling Christian Schlechtriem Frainhofer IME, Schmallenberg Helmut Segner Centre for Fish and Wildlife Health, University of Bern

helmut.segner@vetsuisse.unibe.ch

The standard test for regulatory purposes to determine bioaccumulation of xenobiotics in fish is the OECD TG 305, an in-vivo test which uses a high number of animals (> 100). Recently, in-vitro metabolism assays using isolated hepatocytes from fish have been introduced as possible alternative to the in-vivo bioaccumulation test. Currently, work is under way to demonstrate the reproducibility of the method supporting a formal validation. A drawback, however, is that the available in-vitro protocols have been developed exclusively for rainbow trout (Oncorhynchus mykiss), but not for the other fish species commonly used under TG 305 such as common carp (Cyprinus carpio). In order to overcome this limitation and to foster the acceptance of the in-vitro method, it must be demonstrated that the protocol is not restricted to one specific fish species. The aim of this study was to evaluate if the existing in-vitro methodology for determining xenobiotic clearance in isolated hepatocytes of rainbow trout can be adapted to carp hepatocytes. In a first step, a protocol for isolating carp hepatocytes based on a two-step collagenase-perfusion technique was developed, leading to high cell yield and good cell quality. In a next step, existing protocols for measuring xenobiotic clearance were applied to freshly isolated hepatocytes from trout and carp. As model substances, benzo[a]pyrene (BaP) and methoxychlor (MCX) were used. Incubation temperature for carp hepatocytes was 20°C, while it was 15°C for trout hepatocytes. Irrespective of the higher incubation temperature in the carp assay, xenobiotic clearance rates tended to be lower with carp hepatocytes than with trout hepatocytes. The results from this study demonstrate that the in-vitro metabolism assay protocol developed for the cold water species rainbow trout in principal is transferable to other species. What remains to be evaluated is whether the observed differences of the in vitro metabolic rates between carp and trout hepatocytes are able to predict species differences of in vivo xenobiotic metabolism.

Acknowledgement: This study is financially supported by “Stiftung Forschung 3R”

P29. RTgill-W1 cell line assay for predicting fish acute toxicity: evaluation of a round-robin test

Knöbel Melanie, Eawag

S.E. Belanger 2, P. Berckmans 3, M.J. Bernhard 2, L. Bláha 4, S.D. Dyer 2, T. Haupt 5, J.L.M. Hermens 6, M.T. Hultman 7, H. Laue 5, A. Lillicrap 7, M. Mlnaříková 4, A. Natsch 5, J. Novák 4, T.L. Sinnige 6, K.E. Tollefsen 7, V. von Niederhäusern 5, H. Witters 3, K. Schirmer 1 1 Eawag, Swiss Federal Institute of Aquatic Science and Technology, Dübendorf, Switzerland 2 The Procter & Gamble Company, Environmental Stewardship Organization, Cincinnati, Ohio, USA 3 VITO-ABS team, Flemish Institute for Technological Research, Mol, Belgium 4 RECETOX, Masaryk University, Brno, Czech Republic 5 Givaudan Schweiz AG, Dübendorf, Switzerland 6 IRAS-Tox, Utrecht University, Utrecht, The Netherlands 7 Norwegian Institute for Water Research (NIVA), Oslo, Norway

melanie.knoebel@eawag.ch

The rainbow trout (Oncorhynchus mykiss) gill cell line, RTgill-W1, has been tested to predict fish (in vivo) acute toxicity, hence, an animal alternative to tests used for chemical risk assessment. In a previous study, 34 organic industrial chemicals (selected using pre-defined criteria), were tested and compared to the acute toxicity reported in the US EPA fathead minnow data base. The majority of data was within a 10-fold range versus acute fish lethality when taking into account bioavailable exposure concentrations. Moreover, the outcome of the cell line assay compared very well to results obtained in the zebrafish embryo toxicity test (FET), which has meanwhile been accepted as the OECD test guideline 236. These findings provided the impetus to initiate the RTgill-W1 cell line assay as a potential animal replacement method to the same level of international acceptance. Through support of CEFIC-LRI and UK NC3Rs, an Eawag-led round-robin test, involving six industrial and academic research laboratories from Europe and the USA was conducted. The overall goal was to test the robustness of the established methodology. In particular, the transferability of the RTgill-W1 cell line assay and the intra- and inter-laboratory reproducibility was evaluated.

Seven laboratories provided data for the round-robin testing, either for the cell line assays and/or analyses of chemical concentrations. Chemical selection and quantitative analysis as well as conductance of the cell line assay followed criteria and protocols used in the initial study. Experiments with the cell line assay include a minimum of three biological replicates (cells from different passages) with three technical replicates in each test run. Results of cell viability measurements are based on verified exposure concentrations. Indeed, the RTgill-W1 cell line assay procedure was transferred successfully to each of the participating laboratories. Improvements to the original standard protocols were implemented based on the suggestions provided by the participants – leading to further simplification.

Data was analysed using diverse statistical methods with a specific focus on inter- and intra-laboratory variability, providing a solid basis that will likely establish this method as an animal-free alternative to the fish acute toxicity test.

P30. Untersuchung des Wirkmechanismus von TBT in Potamopyrgus antipodarum - Rangefindertest mit RXR-Agonisten und einem RXR-Antagonisten

Bruhn Torben, Goethe-Universität Frankfurt am Main
Cornelia Geiß, Katharina Ruppert, Jörg Oehlmann

torben.bruhn@stud.uni-frankfurt.de

Das als Antifouling-Biozid verwendete Tributylzinn (TBT) ist einer der giftigsten Substanzen für Wasserorganismen. TBT gehört zu den Endokrinen Disruptoren (ED) und wirkt bereits in sehr geringen Konzentrationen. Es führt bei mehr als 260 Arten der Weibchen getrenntgeschlechtlicher Caenogastropoden zur Ausbildung von männlichen Geschlechtsorganen, dem sogenannten Imposex. Ein vorgeschlagener Modellorganismus für die Untersuchung endokrin wirksamer Substanzen ist die Neuseeländische Zwergdeckelschnecke Potamopyrgus antipodarum, mit der bereits zahlreiche Untersuchungen mit diesem androgenen ED durchgeführt worden sind. Der genaue Wirkmechanismus von TBT ist bisher jedoch noch nicht vollständig aufgeklärt. Neben der Hypothese einer Hemmung der Aromatase wird in der Literatur eine Beteiligung des Retinoid X Rezeptors (RXR) diskutiert, an dem TBT agonistisch wirkt.

Vor diesem Hintergrund wurden drei Substanzen, die in Versuchen mit anderen Gastropoden als Imposex auslösende Substanzen in Verbindung mit dem RXR identifiziert wurden, untersucht. In einem 28-tägigen Rangefindertest mit P. antipodarum wurden die Effekte von 9-cis-Retinsäure als natürlichem Liganden des RXR, des synthetischen Agonisten HX630 und des synthetischen Antagonisten HX531 in Kombination mit einer festen TBT-Konzentration von 160 ng-Sn/L in fünf verschiedenen Konzentrationen von 0,01 bis 100 µg/L auf die Reproduktion von P. antipodarum getestet. Als Positivkontrolle diente die feste TBT-Konzentration.

Nach vier Wochen wurden die Mortalität und die Embryonenzahlen der Zwergdeckelschnecken ausgewertet. Die Ergebnisse ergaben für 9-cis-Retinsäure einen tendenziellen und bei HX630 sowie bei HX531 in Kombination mit TBT einen signifikanten Rückgang der Embryonenzahlen. Im Beitrag werden diese Ergebnisse des Rangfindertests vorgestellt und diskutiert.

P31. Influence of Multiwalled Carbon Nanotubes on the Toxicity of Triclocarban and its Bioaccumulation in Daphnia magna and Artemia salina

Treidy Stephan, RWTH Aachen

Hennig Michael Patrick Schäffer Andreas Maes Hanna Maja

stephantreidy@yahoo.de

Disposal and degradation of multiwalled carbon nanotubes (MWCNTs)-containing plastic products may lead to a release of MWCNT in different environmental media. Due to the ability of MWCNT to sorb various, mostly lipophilic compounds like triclocarban (TCC), it is necessary to investigate sorption, mixture toxicity and bioaccumulation in water invertebrates after exposure.

This study was set up to examine the sedimentation of 14C-MWCNT (1 mg/L) in artificial fresh- (Elendt medium, M4) and sea water (35 g/L sea salt, pH 8.1, SW) within 8 days. In both media, the sorption behavior of 14C-TCC on MWCNT was evaluated. Afterwards, acute mixture toxicity of MWCNT and TCC to Daphnia magna (OECD 202) and Artemia salina (adapted to OECD 202) were investigated. Furthermore, uptake and elimination of 14C-TCC and 14C-MWCNT were quantified in order to derive the bioaccumulation potential of the biocide and the nanomaterial alone and in combination.

14C-MWCNT deposited faster in SW than in M4 (18% ± 1,7% in M4, 1% ± 0,18% in SW after 192 h). Higher bioaccumulation of 14C-MWCNT after 48 h was shown in daphnids (5.97 ± 0.99 µg CNT/ mg dw; n=4) compared to in brine shrimps (0.45 ± 0.04 µg CNT/ mg dw; n=4). Furthermore, toxicity tests illustrated a toxic effect of TCC towards both organisms. The lowest observed effect concentration (LOEC) amounted to be 40 µg/L for daphnids ≤ 2,5 µg/L for brine shrimps. Median effect concentrations (EC50) of 22 µg/L (daphnids) and 9 µg/L (brine shrimp) could be calculated.

The presence of MWCNTs reduced the toxicity of TCC only for Daphnia magna. For Artemia salina the acute toxicity is even enhanced. These results show that MWCNTs lower the bioavailability of TCC to saltwater shrimps although this nanomaterial is relatively largely taken up. Hence, it seems that TCC bound to MWCNTs is hindered from passing the gut epithelium of Daphnia Magna. This is important with regard to possible food chain transport of MWCNT-bound TCC.

P32. Verhaltensversuche mit Zebrafisch-Larven zur Grenzwertbestimmung von drei Modellsubstanzen

Hartmann Sarah, RWTH Aachen- Institut für Umweltforschung

Leonie Nüsser, Thomas-Benjamin Seiler, Henner Hollert

sarah.hartmann@rwth-aachen.de

Im Rahmen des Kooperationsprojekt W3-Hydro soll ein Computersystem entwickelt werden, dass dazu dient, Verunreinigungen im Wasserkörper einer Trinkwasseranlage schnellst möglichst anzuzeigen, um weder menschliches noch tierisches Wohl zu gefährden. Dafür wird in diesem Projekt ein Biosensor entwickelt, der auf Verhaltensveränderungen von Zebrabärbling-Larven reagiert, welche durch eine Kontamination ausgelöst wurden. Verhaltensversuche bekommen auf dem Gebiet der Ökotoxikologie eine immer wichtigere Bedeutung. Die Larven des Zebrabärblings (Danio rerio) reagieren sehr sensitiv auf toxische Substanzen und sind daher ein wichtiger und etablierter Modellorganismus in der Ökotoxikologie geworden. Experimente mit Danio rerio gelten bis zu einer Zeit von 120 hpf (Stunden nach Befruchtung) als tierversuchsfrei und stellen somit eine gute Alternative zu echten Tierversuchen dar. Veränderungen im Schwimmverhalten und der Aktivität der geschlüpften Larven sind sensible Endpunkte und erlauben somit eine schnelle Detektion von toxischen Substanzen. Darüber hinaus erlauben Ergebnisse aus kontrollierten Verhaltensversuchen im Labor, Rückschlüsse auf mögliche Veränderungen der Verhaltensweise von Fischen, die in kontaminiertem Gewässer leben. In dieser Arbeit wird experimentell mithilfe eines Durchfluss-Well-Plattensystems die Aktivität der belasteten Larven gemessen und mit einer Negativkontrolle verglichen. Durchgeführt werden die Experimente mit einem Trackinggerät (DanioVision, Noldus, Wageningen, Niederlande). Vor Versuchsbeginn werden die Larven randomisiert auf eine herkömmlichen 96-Well Platte aufgetragen, um bei der Auswertung das Verhalten jedes Individuums einzeln auszuwerten und Platteneffekte auszuschließen. Das System wird mit einem erklärten Hell-Dunkel-Rhythmus und über einen bestimmten Zeitraum programmiert, um in zeitlich genau definierten Abständen eine Reaktion der Larven zu induzieren, die über die normale Grundaktivität hinausgeht. Eine Veränderung im Verhalten soll so schnell wie möglich detektiert werden. Daher beträgt der Expositionszeitraum für die Larven lediglich 90 Minuten. Im Forschungsprojekt werden die Substanzen Permethrin, Cadmium und Chinolin untersucht. Aufgrund der gut untersuchten „modes of action“, wurden diese Substanzen als Modellvertreter ihrer Stoffklasse ausgewählt. Des Weiteren sollen molekulare Untersuchungen Aufschluss darüber geben, ob diese Substanzen zu einer veränderten Genexpression führen. Die Gene cyp1a, cyp1b1 und cyp1c1, ahRr2, abcc5, abcc2 und tuba1 dienen für eine der drei Stoffklassen als spezifische Marker. Ziel der Arbeit ist es zu determinieren wie sensitiv das Verhalten von Zebrabärbling-Larven als ökotoxikologischer Endpunkt reagiert und ob ein solcher Versuchsansatz genutzt werden kann, um einen on-line Biosensor zu entwickeln.

P33. In vitro assessment of immunotoxic potentials of chemicals for rainbow trout (Oncorhynchus mykiss)

Rehberger Kristina, Centre for Fish and Wildlife Health, University of Bern

Inge Werner Oekotoxzentrum Eawag-EPFL, Dübendorf and Lausanne Helmut Segner Centre for Fish and Wildlife Health, University of Bern

helmut.segner@vetsuisse.unibe.ch

Numerous legacy and emerging contaminants including persistent organic pollutants and pharmaceuticals are (potentially) immunotoxic to fish. It was shown that exposure of fish to immunotoxic contaminants can adversely affect immunocompetence and pathogen resistance, and thereby may indirectly contribute to the increasing occurrence of pathogen-induced fish mortalities. This situation leads to the question how to screen and assess immunotoxic activities of environmental contaminants. Currently, there exists no established inventory for immunotoxicity testing and assessment in ecotoxicology. The present study aims to examine the suitability of in vitro assays using fish immune cells to screen for potentially immunotoxic chemicals. To this end, primary head kidney leukocytes (resting and stimulated) of rainbow trout (Oncorhynchus mykiss) were exposed to non-cytotoxic concentrations of xenobiotics with different modes of action. The panel of test compounds includes narcotic chemicals, specifically acting non-immunotoxic chemicals, and immunotoxic chemicals which act through different receptor pathways. The selection of the endpoints for detection of an immunomodulatory activity of the test compounds was based on a previously conducted literature review, which identified phagocytic activity, oxidative burst activity, and the expression of cytokines to be most frequently measured and responsive in fish. The results of the present study will clarify whether simple in vitro screening assays are able to capture immunotoxic potentials of xenobiotics irrespective of the specific MoA, and thus may be useful for chemical prioritization in ecotoxicological hazard assessment.

Ackowledgement: This study is financially supported by the EU project SOLUTIONS

P34. The immune system of fish as a target of estrogen-active environmental compounds

von Siebenthal Elena Wernicke, Centre for Fish and Wildlife Health, University of Bern

Richard Burki Michael Wenger Helmut Segner Ayako Nakayama Centre for Fish and Wildlife Health, University of Bern

helmut.segner@vetsuisse.unibe.ch

Endocrine disruption, in particular disruption by estrogen-active compounds, has been identified as an important ecotoxicological hazard in the aquatic environment. Research on the impact of endocrine disrupting compounds (EDCs) on wildlife has focused on disturbances of the reproductive system. However, there is increasing evidence that EDCs affect a variety of physiological systems other than the reproductive system. Here, we provide evidence for the immune system of fish being a target of estrogenic substances of fish by showing that (i) piscine immune cells express estrogen receptors, (ii) exposure of fish to estrogens modulates immune gene expression, (iii) exposure of fish to estrogens impairs the resistance towards infectious pathogens thereby leading to increased mortalities. The immunomodulating effects of estrogens occur at the same concentration range which is able to impact gonad differentiation and reproduction. These findings suggest that estrogenic compounds can impact the ecological fitness of fish not only through impairment of the reproductive system, but possibly also via a modulation of the immune defense system.

Acknowledgement: This study is financially supported the Swiss National Science Foundation

P35. NanoCompetence in der Gesellschaft: Forschung – Vermittlung -- Gestaltung

Gräf Tonya, Universität Bremen, UFT, Allgemeine und theoretische Ökologie
Jürgen Warrelmann, Juliane Filser

grf@uni-bremen.de

Der fortwährend steigende Gebrauch von Nanomaterialien im alltäglichen Leben führte im letzten Jahrzehnt zu einem wachsenden Umwelteintrag der Materialien aus anthropogenen Quellen über diverse Eintragspfade. Gleichzeitig befindet sich die Nanotechnologie auch weiterhin in einem Entwicklungsprozess. Es besteht daher die dringende Notwendigkeit, aufgrund der nicht zu unterschätzenden positiven Einsatzmöglichkeiten der Nanotechnologie, die Rahmenbedingungen für einen zukünftig sicheren Umgang den neuen Gegebenheiten anzupassen. Um zu den positiven und negativen Aspekten der neuen Technologie einen gesamtgesellschaftlich kritischen Diskurs ins Leben zu rufen bzw. am Leben zu halten, ist es notwendig, die in wissenschaftlichen Studien gewonnenen Daten unter verschiedensten Aspekten aufzuarbeiten und einer Mehrheit der Bevölkerung zugänglich zu machen, um damit das Urteilsvermögen der VerbraucherInnen auf eine solide Grundlage zu stellen.

Der hierfür gewählte Forschungsansatz des Promotionskollegs NanoCompetence (Homepage unter www.nano.uni-bremen.de) basiert auf der Idee, Forschungsgruppen aus dem gesellschafts- wie auch naturwissenschaftlichen Bereich paritätisch zusammenzuführen. Durch die Bildung von Tandems, jeweils aus naturwissenschaftlichem und gesellschaftswissenschaftlichem Bereich, ist eine interdisziplinäre Bearbeitung der Thematik gewährleistet.

Auf Basis der derzeitigen Datenlage für Kupferoxid- und Ceroxid-Nanopartikel ist eine umfassende Gefährdungsabschätzung für diese Nanopartikel nicht möglich. Die im Rahmen des Promotionskolleg NanoCompetence durchzuführenden Untersuchungen für diese beiden gewählten Fallbeispiele leisten einen wichtigen Beitrag Datendefizite auszugleichen und in einen gesellschaftlichen Kontext zu stellen. Erste laufende Partikelstudien sollen ergänzende Einblicke in die teilweise unzureichende Datenlage bezüglich ökotoxikologischer Auswirkungen in aquatischen und terrestrischen Systemen geben. Fragestellungen nach dem Einfluss der Expositionsdauer auf die Testsysteme und mögliche Sensitivitätsunterschiede der beiden Systeme, sollen untersucht und bewertet werden. Die Ergebnisse der Vorstudien mit Daphnien und Enchytraeiden als Modellorganismen für aquatische bzw. terrestrische Testsysteme fließen in den Beitrag ein.

Terrestrische Ökotoxikologie

P36. Bestimmung der Bestäubungseffizienz von mit Imidacloprid behandelten Hummeln (Bombus terrestris) anhand der Effekte auf Qualität und Quantität der Ernte von Ackerbohnen (Vicia faba)

Eschenbach Eva, Institut für Umweltwissenschaften, Universität Koblenz-Landau
Claudia Wollmann, Philipp Uhl, Peter Stahlschmidt, Carsten A. Brühl

eva.eschenbach@gmx.de

Bestäubung ist eine wichtige Ökosystem-Dienstleistung welche in den letzten Jahren immer mehr abnimmt. Eine Hauptgruppe unter den Bestäubern sind Insekten und genauer, Bienen. Dazu gehört nicht nur die Honigbiene, sonder ebenso Solitärbienen und Hummeln. Vor allem der Beitrag der Hummel zur Bestäubung wird oft unterschätzt, dabei ist kommerzielle Bestäubung für einige Kulturpflanzen ohne Hummeln heutzutage kaum noch vorstellbar (beispielsweise in Gewächshäusern und Obstplantagen). Zwei wichtige Gründe für den hohen Rückgang von Hummelarten und Völkern in den letzten Jahren sind steigende Pestizid-Applikationen und, durch Intensivierung der Landwirtschaft entstehende, Habitatfragmentierung/-zerstörung, wovon auch wichtige Lebensräume von Hummeln wie beispielsweise Feldsäume betroffen sind. Neben der Mortalität spielen auch subletale Effekte eine große ökonomische und ökologische Rolle bei der Bestäubung. Bisher haben sich viele Studien hauptsächlich mit subletalen Effekten (z.B. reduzierter Sammelflug) auf die Hummel beschäftigt.

Die vorliegende Studie soll nun mögliche Effekte von Pestiziden auf die Bestäubungseffizienz von Hummeln untersuchen und damit den Einfluss auf die Qualität und Quantität der Ernte. In einem Halbfreiland-Versuch werden Ackerbohnen (Vicia faba) mit dem Insektizid Imidacloprid aus der Gruppe der Neonicotinoide behandelt und anschließend 30 Tage von Bombus terrestris Mikro-Kolonien bestäubt. Die Bohnenschoten werden nach der Reife geerntet und auf Qualität (Proteingehalt, Krankheitsbefall) und Quantität (Menge, Gewicht) untersucht. Es werden zwei Imidacloprid Konzentrationen untersucht, 100 % für V. faba im Feldfrucht-Szenario und 30 % für V. faba im Feldsaum-Szenario. Wir erwarten, dass die Qualität und Quantität der Schoten bzw. Samen durch eine verringerte Bestäubungseffizienz der Hummeln beeinflusst wird. Vor allem der Proteingehalt der Samen könnte reduziert werden, was ökonomische Konsequenzen nach sich ziehen würde, da die Ackerbohne gerade wegen ihres hohen Proteingehalt (=Energiegehalt) angebaut wird. Die Studie wird von Juli bis Mitte August durchgeführt, Ergebnissen werden spätestens Ende August erwartet.

P37. OECD Guideline 227, Vegetative Vigour Test – Nehmen Substratvolumen und -oberfläche sowie die Art der Biomassebestimmung Einfluss auf die Effekte bei einer Pflanzenart?

Siemoneit-Gast Sandra, Forschung

Inga Braje, Roland Kubiak

sandra.siemoneit@agroscience.rlp.de

Die oben genannte Richtlinie wurde entwickelt, um ein Testdesign zur Hand zu haben, mit dem die Effekte auf das vegetative Wachstum von terrestrischen Pflanzen nach Pflanzenschutzmittel (PSM)- Einwirkung erforscht werden kann. Im 2-4 Blattstadium der im Topf vorgezogenen Pflanzen wird das zu testende Pflanzenschutzmittel auf die Blattoberfläche appliziert. Nach der Applikation werden die behandelten Pflanzen gegen die unbehandelte Kontrolle verglichen, um Rückschlüsse auf Vitalität und Wachstum ziehen zu können. Hierbei kann die Laufzeit der Studie bis 28 Tage betragen, jedoch wird eine Laufzeit von 21 Tagen für eine Anzahl von Pflanzenarten als ausreichend erachtet.

In der Richtlinie wird unter dem Punkt "Test Design" hervorgehoben, dass die Pflanzenanzahl pro Topf der Pflanzenspezies, der gewählten Topfgröße sowie der Testlaufzeit angemessen zu wählen ist. Hintergrund dieser Empfehlung ist es, den Pflanzen über Substratvolumen und -oberfläche angemessene und einheitliche Wachstumsbedingungen zu bieten, sowie Konkurrenz um Platz und Licht während der experimentellen Phase auszuschließen. Effekte auf Vitalität und Wachstum sollen einzig und allein auf die Einwirkungen des PSM zurückzuführen sein.

Unter dem Punkt "Observations" wird in der Richtlinie darauf hingewiesen, dass bei der abschließenden Biomasse-Bestimmung wahlweise sowohl die oberirdische Frisch- als auch die Trockenmasse bestimmt werden kann. Es wird angenommen, dass dies keinen Einfluss auf das Ergebnis nimmt.

Am Institut für Agrarökologie der RLP AgroScience wurden an drei ausgewählten Pflanzenarten (Zea mays, Lactuca sativa und Brassica napus) die beiden oben genannten Punkte der Richtlinie bezüglich ihres Einflusses auf die erfassten Effekte hin GLP-konform untersucht. Hierfür wurde entsprechend den Vorgaben aus der Richtlinie ein Rate-Response Test durchgeführt. Als Testsubstanz wurde ein handelsübliches, als ALS-Hemmer wirkendes Herbizid gewählt. Die Kultur der oben genannten Pflanzenarten fand in zwei Topfgrößen und in unterschiedlicher Pflanzendichte statt. Die einzelnen Testvarianten selbst standen – nach Pflanzenart geordnet – zeitgleich im Gewächshaus. Am Ende der experimentellen Phase wurden sowohl das Frisch- als auch das Trockengewicht je Pflanzenart erfasst.

Der Einfluss von Substratvolumen und -oberfläche sowie der Art der Biomassebestimmung werden dargestellt und diskutiert.

P38. Wing contact as a new exposure scenario in acute toxicity testing with the solitary bee Osmia bicornis

Wollmann Claudia, Universität Landau
Eva Eschenbach, Philipp Uhl, Peter Stahlschmidt, Carsten Brühl

woll7400@uni-landau.de

In the Tier I approach, the risk of pesticides to bees is usually assessed through oral and contact exposure. With application of the substance on the thorax representing all possible contact exposure scenarios, routes of entry other than the thorax are not considered in risk assessment. To present, only one study has dealt with the uptake of insecticides through the wings in the honey bee Apis mellifera. In our study we aim to investigate this new route of exposure via wing contact with the solitary bee Osmia bicornis. An LD50 value of the active substance dimethoate will be established for Osmia bicornis males by applying the substance on the rear part of the wings that is not in direct contact with the abdomen. In addition, a standard thorax contact test will be set up. A comparison of acute thorax and wing contact LD50 values will determine if contact exposure through the wings is a relevant exposure scenario that should be considered in pollinator risk assessment.

P39. Nesting success of Osmia bicornis females in landscapes with varying levels of food abundance

Wollmann Claudia, Universität Landau
Eva Eschenbach, Philipp Uhl, Peter Stahlschmidt, Carsten Brühl

woll7400@uni-landau.de

Pollination is a key ecosystem service in decline due to a number of factors of which habitat decline and fragmentation pose one of the major problems. Wild pollinators like the red mason bee Osmia bicornis rely on suitable habitats as nesting and foraging sites. Existing data on the foraging range of Osmia bicornis females is not conclusive and it is unclear if the females are able to explore alternative food sources when they are scarce around the habitat site. In this study we aim to investigate how food availability influences the attractiveness of nesting aids to Osmia bicornis and the subsequent brood development. In a field trial, nesting aids containing female and male cocoons are placed in three different types of landscapes that provide varying levels of food abundance. Pollen samples from the nesting aids used for the provision of the larvae are analysed for their origin. The pollen is inspected to determine if low food availability in the field provided as habitat results in an adaptation of their foraging behaviour and in an increased flight range. First inspections of the collected pollen indicate that Osmia bicornis females are indeed able to adapt their foraging behaviour in landscapes with scarce food resources and that abundance of Osmia bicornis is not influenced by the degree of habitat isolation.

Bodenökotoxikologie

P40. Comparison of soil invertebrate feeding activity and copper concentration in two vineyards with different cultivation ages

Campiche Sophie, Schweizerisches Zentrum für angewandte Oekotoxikologie Eawag-EPFL

Eva Kohlschmid(2), Emilie Grand(1),Peter Kauf(3) Otto Daniel(2), Inge Werner(1), Benoît J.- D. Ferrari(1) (1) Swiss Centre for Applied Ecotoxicology Eawag/EPFL, 8600 Dübendorf, Switzerland (2) Agroscope, Institut für Pflanzenbauwissenschaften IPB, Forschungsgruppe Ökotoxikologie (3) Zürcher Hochschule für Angewandte Wissenschaften (ZHAW)

sophie.campiche@centreecotox.ch

Viticulture still relies on copper based fungicides applied against Downy Mildew. Copper accumulates in soil if used continuously. In this study we investigated two different vineyards, the first one was 100 years under cultivation and the second one was 15 years under cultivation, in terms of metal concentration and soil functional activity. Soil samples from the two sites were taken in two seasons (2011 and 2014) for soil moisture content and copper, lead as well as zinc content analysis. Additionally the soil functional activity was assessed by using the bait-lamina method, measuring soil invertebrate feeding activity.

The total copper content of the two sites differed by a factor of 8, due to high copper contents for the site which was 100 years under cultivation. The feeding activity at this site was lower in both seasons compared to the site which was 15 years under cultivation. The role of copper compared to environmental and management related factors influencing the feeding activity of soil organisms is discussed.

P41. Using the combined Bait Lamina method in the laboratory for evaluating the effects of substances on the feeding activity of the worm E. Andrei

Campiche Sophie, Swiss Centre for Applied Ecotoxicology Eawag/EPFL

M. Visse, I. Werner, B.J.D. Ferrari Centre Ecotox Eawag/EPFL, EPFL-ENAC-IIE-GE station 2, 1015 Lausanne, Suisse

sophie.campiche@centreecotox.ch

Among the tools used to assess soil quality, the bait lamina test is a simple, fast and inexpensive method to measure the functional aspect of the soil ecosystem. The feeding activity (biological activity) of soil invertebrates is assessed in situ at the community level by measuring the disappearance of a series of organic bait ("bait") embedded in small PVC sticks inserted vertically into the soil. The method allows studying the pollution gradients on the field, but also to assess the impact of pesticide applications on the biological activity of the soil. It can also be used as a screening method for a first characterization of the sites and their biomonitoring. A draft of an international standard (ISO / DIS 18311) is available since 2014.

Given the ecological relevance of the biological response assessed in situ by this method, we investigated the usefullness of this approach in a priori risk assessment of substances in the laboratory. The objective was thus to adapt the bait lamina method for measuring the feeding activity of the species Eisenia andrei in a simple test under laboratory condition. Briefly, 5 bait-lamina sticks are planted in each 500-mL replicate containing the soil contaminated to the desired concentration of test substance and 5 E. andrei. After 48-h of exposure, the percentage of bait cosumed by earthworms is calculated for each of the tested concentrations and the EC50 is determined. Our results showed a clear concentration-response relationship. Moreover, in order to give an insight of the sensitivity, this method was compared in parallel to the standardized avoidance test (ISO 11268-2) and the reproduction test (ISO 17512-1). Preliminary results show similar sensitivity of the Bait Lamina feeding test and the avoidance test. Concerning the reproduction test, it appears less sensitive.

The use of such a relatively simple laboratory method to assess feeding activity of earthworms will allow the rapid screening of soil samples for sublethal effects due to the impact of chemicals, and an assessment of the functional aspect of soils under standardized conditions. This approach shows promise for the a priori ecological risk assessment of chemicals.

Passive Sammler

P42. Suitability of Chemcatcher® passive samplers for the comprehensive screening of micropollutants in rivers

Moschet Christoph, Eawag, Swiss Federal Institute of Aquatic Science and Technology

Etiënne Vermeirssen, Heinz Singer, Christian Stamm, Juliane Hollender

cmoschet@ucdavis.edu

The goal of our study was to determine the suitability of passive sampling for a comprehensive screening of a very large number of polar to semi-polar micropollutants that are occurring in streams and rivers. A field study was carried out where 45 Chemcatchers® were deployed, for two week periods, in five agriculturally and urban influenced Swiss rivers from March to July 2012. During the same time periods, two- week time-proportional composite water samples were taken for comparison. The investigated 322 substances – pesticides, pharmaceuticals, industrial chemicals and their transformation products with divers physico-chemical properties (logKow -3 to 5, neutral, anionic, cationic, and zwitterionic species) – were analysed by liquid- chromatography high-resolution tandem mass spectrometry. Results showed that Chemcatchers® are perfectly suited for the qualitative screening of polar micropollutants. The number of detected substances was similar for both sampling methods (204 for passive samples vs. 207 for composite water samples), limits of quantification were comparable (median: 1.3 ng/L vs. 1.6 ng/L), and the handling of Chemcatchers® in the field and laboratory is fast and easy. The fact that so many different substances accumulate on the sampler also opens the door for a comprehensive screening for suspects or unknowns. In-situ calibrated sampling rates (field Rs) could be determined for 88 compounds, i.e. where a good regression was obtained between water concentration vs. sampled mass on passive sampler disk. It could be clearly shown that substances with moderately fluctuating concentrations such as pharmaceuticals showed better correlations than substances with highly fluctuating concentrations such as pesticides ($R2 > 0.75$ for 93% and 60% of the investigated substances, respectively). It was observed that ionic species had significantly lower field Rs than neutral species. However, a correlation between determined field Rs and hydrophobicity (logDow) could only predict Rs with large uncertainties. We conclude that substances with relatively constant concentrations can be quantified accurately in the field by passive sampling. To do so, substance-specific Rs for these compounds need to be determined. For that purpose, in-situ calibration is a very robust method and the determined Rs can be used in future monitoring studies in rivers with similar environmental conditions.

Umweltchemie

P43. Abbauwege und Kinetik der nanoPd(0) katalysierten Dehalogenierung von Polychlorierten Biphenylen

Axt Florian, Institut für Bodenkunde und Bodenerhaltung, IFZ für Umweltsicherung, Justus-Liebig-Universität Gießen

Leonard Böhm, Manuel Wohde, Rolf-Alexander Düring, Institut für Bodenkunde und Bodenerhaltung, IFZ für Umweltsicherung, Justus-Liebig-Universität Gießen

Florian.M.Axt@umwelt.uni-giessen.de

Bei Polychlorierten Biphenylen (PCB) handelt es sich um persistente organische Schadstoffe (POPs), die u.a. als Weichmacher und Hydraulikflüssigkeiten verwendet wurden. Die industrielle Herstellung und Nutzung von PCB wurde im Rahmen der Stockholmer Konvention 2001, zusammen mit weiteren POPs („Dirty Dozen“), weltweit untersagt. Dennoch sind PCB ubiquitär verbreitet. Aufgrund ihrer hohen toxikologischen Relevanz als Kanzerogene, Teratogene und endokrine Disruptoren, sowie den nur sehr langsam ablaufenden natürlichen Degradationsmechanismen, ist es sinnvoll, potentielle Remediationsverfahren zu untersuchen.

Metall-Nanopartikel haben das Potential, die vollständige reduktive Dehalogenierung von POPs wie Chlorbenzole, Dioxine und PCB innerhalb kürzester Zeitspannen zu katalysieren. Dazu können unterschiedliche Arten von Metall-Nanopartikeln verwendet werden. Bevorzugt werden aufgrund von möglichen Wiederaufbereitungsverfahren, sowie aus Kostengründen, vor allem bimetallische Partikel (bspw. Pd-Fe, Ni-Fe, Cu-Fe, Ag-Fe). Versuche mit Hexachlorbenzol haben gezeigt, dass der Abbau vor allem durch den induktiven Effekt als wichtigsten ladungsverändernden Effekt beeinflusst wird. Bei PCB ist zudem eine sterische Hinderung, bedingt durch die Doppelringstruktur, zu erwarten. Die hohe Reaktionsgeschwindigkeit der katalysierten Dehalogenierungsprozesse erschwert dabei die Analytik erheblich.

In dieser Studie wurde die Dechlorierung von PCB mithilfe von chemisch synthetisierten Pd(0)-Nanopartikeln und ausgewählten PCB-Kongeneren als Edukt untersucht. Die Versuche wurden unter anoxischen Bedingungen in Mikroreaktionssystemen (10mL) durchgeführt, H2 diente dabei als Elektronendonator. Die Analyten wurden mittels SPME-GC/MS detektiert. Das Dehalogenierungspotential der Pd(0)-Nanopartikel wurde in Anwesenheit verschiedener Pd(0)-Konzentrationen (0,25 – 10 mg L^{-1}) quantifziert.

Eine vollständige Dechlorierung bis hin zu Biphenyl konnte bei allen untersuchten Kongeneren nachgewiesen werden. Die Pd(0)-Nanopartikel wiesen dabei eine katalytische Aktivität von bis zu 378 L g^{-1} min $^{-1}$ bei einer Konzentration von 3 µg L^{-1} PCB auf. Experimentelle Ansätze mit Additiven, um die Reaktionsgeschwindigkeit zu verlangsamen, waren nicht erfolgreich. Dennoch konnten unter Verwendung niedriger Pd(0)-Konzentrationen zahlreiche Intermediate nachgewiesen werden.

Die hier verwendete Methode eignet sich gut, um die nanoPd(0) katalysierte Dehalogenierung von PCB näher zu untersuchen. Abweichende Dehalogenierungsgeschwindigkeiten ausgewählter PCB-Isomere lassen auf den je nach Chlorierungsposition abweichenden

Einfluss von induktiven und mesomeren Effekten sowie sterischer Hinderung schließen. Um das Verständnis der einflussnehmenden Effekte noch umfassender aufklären zu können, sind weitere Versuche mit ausgewählten Kongeneren notwendig.

P44. Method development for mercury speciation in biota

Fettig Ina, Bundesanstalt für materialforschung und -prüfung
Christian Piechotta Enrica Alasonati Claudia Swart Jan Koschorrek Paola Fisicaro

ina.fettig@bam.de

In 2000 the European Waterframework Directive (WFD 2000/06/EC) was remitted to standardize the monitoring of aquatic ecosystems and ground waters within the EU. Furthermore, the improvement of water quality and the sustainable usage of water are main objectives of the WFD. Within the scope of the project “Traceability for mercury measurements” of the European Metrology Research Programme (EMRP) quantitative analytical methods for the priority substance mercury will be developed.

Inorganic Mercury and its methylated chemical forms are highly toxic to human, animal and environmental health. Its high affinity to accumulate in terrestrial and aquatic biosystems makes it a particularly insidious threat to environmental sustainability. Due their long lifetimes and the ability to be transported in air over long distances lead to an ubiquitous occurrence in all environmental compartments and make them to pollutants of global concern. The assessment of the ongoing effect of mercury on humans and the environment is critically dependent on reliable accurate measurements to assess concentrations and trends. This is challenging because of the various chemical forms of mercury and its presence in a number of different matrices.

One of the main aims of the project is:

• To develop primary measurement procedures for mercury speciation in water and biota in order to improve mercury monitoring through the aquatic ecosystems and to support European legislation. This will include the evaluation of transformation artefacts associated with sample collection and preparation, in order to minimise species conversion post-sampling.

The project scope will be presented. First results of mercury measurements in biota will be demonstrated. Therefore samples of the Environmental Specimen Bank from UBA are extracted and analyzed with GC-ICPMS. Quantification is carried out with isotope dilution.

P45. Einfluss der Zwischenschicht-Kationen in Tonmineralen auf das Sorptionsverhalten von Hexachlorbenzol

Heyde Benjamin, Institut für Bodenkunde und Bodenerhaltung, IFZ für Umweltsicherung, Justus-Liebig-Universität Gießen

Leonard Böhm, Rolf-Alexander Düring, Institut für Bodenkunde und Bodenerhaltung, IFZ für Umweltsicherung, Justus-Liebig-Universität Gießen

Benjamin.Heyde@umwelt.uni-giessen.de

Hexachlorbenzol (HCB) ist als persistenter und hydrophober organischer Schadstoff (HOC) gemäß Stockholm-Konvention weltweit geächtet. Nachwievor ist es in zahlreichen Umweltkompartimenten nachweisbar, in sehr hohen Konzentrationen u.a. in Sedimenten des Rheins. Die organische Substanz (OS) in Böden und Sedimenten gilt als Hauptsorbent für die Sorption von HOCs. Allerdings finden sich in der Literatur auch Hinweise auf die Adsorption von HOCs an mineralische Bestandteile. Hierbei gibt es Hinweise auf eine Abhängigkeit der Sorption von den mineralischen Zwischenkationen. Dies wird unter anderem auf die Größe der Hydrathüllen und deren Effekt auf die n-π Elektronen-Donator-Akzeptor Interaktion (n-π EDA) zurückgeführt.

In der folgenden Arbeit wurde der Einfluss von Zwischenschichtkationen in Tonmineralen auf die Sorption von HCB anhand von Alkali- und Erdalkalimetallen am Beispiel von Montmorillonit als Referenzmineral und Rheinsediment untersucht. Hierzu wurden Tonminerale aus mit HCB kontaminierten Sedimenten isoliert und sonstige Matrixbestandteile eliminiert. Die Sorptionsstudien mit HCB wurden angelehnt an OECD TG 106 durchgeführt; Sorptionsisotherme und Verteilungskoeffizienten (Kd) wurden ermittelt. Aufgrund der HCB-Kontamination der Sedimentproben wurden die Studien mit 13C-HCB durchgeführt. Entsprechend Voruntersuchungen zur Adsorptionskinetik wurden die freigelösten Moleküle 24 Stunden nach der Dotierung mittels headspace solid-phase microextraction (HS-SPME) und GC/MS analysiert. Das Ausmaß der Kationenumbelegung in den Tonmineralzwischenschichten wurde mit inductively coupled plasma optical emission spectrometry (ICP-OES) überprüft. Texturpräparate der umbelegten Tonminerale wurden mit Röntgendiffraktometrie (XRD) untersucht um den Effekt der Kationen auf die Basisabstände zu ermitteln. In den Tonmineralproben wurde die verbleibende OS bestimmt.

Für Montmorillonit ergaben sich log Kd-Werte zwischen 1,20 und 3,91. In den Sedimentproben betrug der log Kd zwischen 3,4 und 3,7. Bezüglich des Referenztons zeigte sich eine ausgeprägte Korrelation mit dem Ausmaß der Hydrathülle der untersuchten Kationen. In den Sedimentproben hingegen ergab sich keine eindeutige Korrelation. Dies kann in der Menge und Zusammensetzung der verbleibenden OS (< 0,4 %) nach der Vorbehandlung begründet sein. Insbesondere die Rolle von Black Carbon (BC) bedarf weiterer Untersuchungen.

Die Ergebnisse tragen dazu bei, die Bedeutung von Tonmineralen in Bezug auf das Sorptionsverhalten von HCB zu beurteilen und die Modellierung des Umweltverhaltens dieses Schadstoffes in Sedimenten und Böden zu verbessern.

P46. Analysis and occurrence of a broad range of surfactants in a wastewater and agriculturally impacted river

Krauskopf Lisa-Marie, UFZ Leipzig

H. Byers, W. Brack, M. Krauss, J. Oehlmann

lisa-marie.krauskopf@ufz.de

Surfactants are used in a wide range of products like household and industrial cleaning agents, personal care products, and pesticide formulations The extent of surfactant production and use is not without risk for the aquatic environment, previous work have shown they might be potential harmful to aquatic ecosystems. The objective of our study was to assess the occurrence of a wide range of surfactants in the river Holtemme in Saxony-Anhalt (Germany), which is affected by multiple stressors as a result of wastewater discharge and agricultural land use. In order to obtain a comprehensive surfactant distribution profile of selected river stretches, samples were taken from sediment, Cladophora algae and river water.

Extraction methods were individually selected and developed with regard to respective sample source. Water samples were taken in 20 mL test tubes and extracted using a QuEChERS approach consisting of a salt-assisted liquid-liquid extraction into acetonitrile, an evaporation of the acetonitrile phase and reconstitution in methanol. This novel method aimed at reducing compound losses due to surface adsorption. Sediment and Cladophora samples were lyophilized and a sequential extraction using pressuring liquid extraction combined with further clean-up steps (normal phase and liquid-liquid extraction) was developed. The cationic, nonionic, anionic and zwitterionic surfactants in all extracts are currently analyzed by liquid-chromatography coupled to high resolution mass spectrometry.

P47. Robustheitsstudie zu optimalen Extraktionsbedingungen für OCP und PCB aus Boden mit unterschiedlichem Corg-Gehalt

Lehnik-Habrink Petra, Bundesanstalt für Materialforschung und -prüfung

Ulrike Mülow Stephanie Näther Wolfram Bremser Christian Piechotta

petra.lehnik-habrink@bam.de

Organochlorpestizide und PCB sind trotz Einsatzverbots aufgrund ihrer Persistenz nach wie vor in der Umwelt, insbesondere im Boden, nachweisbar. Entsprechend sind in der deutschen Bodenschutzverordnung (BBodSchV) nutzungsabhängige Prüf- und Maßnahmewerte für den Eintragspfad Boden-Mensch, Boden-Pflanze und Boden-Grundwasser festgelegt, bei deren Überschreitung eine Bodensanierung bzw. –austausch zu erfolgen hat. Die Extrahierbarkeit der organischen Schadstoffe kann durch den Gehalt an Huminstoffen in der Bodenmatrix zu nicht- bzw. schwer extrahierbaren Rückständen führen. Basis für eine zuverlässige Risikoeinschätzung insbesondere für den direkten Wirkungspfad Boden-Mensch ist die vollständige Extraktion der Schadstoffe aus dem Boden. Gültige DIN-, EN- und ISO-Normen zur Bestimmung von OCP und PCB unterscheiden sich teilweise drastisch bezüglich der Extraktionsmethode und Extraktionszeit.

Im Rahmen der Robustheitsstudie wurden vier Böden mit unterschiedlichem Gesamtkohlenstoff-Gehalt (TOC) und gleichem OCP/PCB-Gehalt durch Verschneiden von einem unkontaminierten Referenzboden mit dotiertem Kompost generiert. Die dotierten Analytgehalte orientierten sich an den Werten der BBodSchV. Diese vier Materialien wurden mittels Accelerated Solvent Extraction (ASE), Soxhlet Extraktion und Flüssig-flüssig-Extraktion aufbereitet. Die Dauer der Flüssig-flüssig-Extraktion variierte zwischen 0.5, 1-, 3-, 6- und 12 Stunden. Die weiteren Analysenparameter wie Extraktionsmittel, Clean-up und Quantifizierung mittels Gaschromatographie-Massenspektrometrie (GC-MS) wurden konstant gehalten.

Bei der statistischen Auswertung der Ergebnisse zeigte sich keine Abhängigkeit der Extraktionsausbeute vom TOC-Gehalt. Längere Extraktionszeiten bei der Flüssig-flüssig-Extraktion zeigten etwas höhere Extraktionsausbeuten, die mit den Ergebnissen der ASE- und Soxhlet-Extraktion korrelieren. Allerdings waren die Standardabweichungen der beiden letztgenannten Methoden deutlich höher, insbesondere bei thermolabilen OCP.

P48. Biokonzentration und Abbau von organischen Spurenstoffen in Phytoplanktonspezies

Stravs Michael, Eawag

Francesco Pomati, Eawag; Juliane Hollender, Eawag, ETH

stravsmi@eawag.ch

Mithilfe Flüssigchromatographie - hochauflösender Massenspektrometrie wurden Biokonzentration und Abbau von organischen Spurenstoffen in zwei Cyanobakterien-Spezies (Microcystis aeruginosa, Synechococcus sp.) sowie in einer Grünalgen-Spezies (Chlamydomonas reinhardtii) untersucht. Es wurde eine Gruppe von Strobilurin-Fungizden und eine Gruppe von Conazol-Fungiziden untersucht, sowie zusätzlich einige häufig in Gewässern vorkommende Pharmazeutika.

Es zeigte sich eine lineare Abhängigkeit des log BCF-Werts von der Hydrophobizität. Hierbei war die Korrelation über alle untersuchten Stoffe schwach (R^2 < 0.2), hingegen innerhalb der Stoffklassen der Strobilurin- und Conazol-Fungizide eindeutig stärker (Strobilurine: R^2 = 0.75, Conazole: R^2 = 0.65). Zusätzlich wurde beobachtet, dass Strobilurine (Trifloxystrobin, Kresoxim-Methyl) in Gegenwart der untersuchten Cyanobakterien-Spezies eine stark beschleunigte Hydrolyse zeigen. Die Ergebnisse weisen auf eine mögliche Rolle von Phytoplankton im Verbleib und Abbau von Mikroverunreinigungen hin.

P49. Methodenentwicklung zur Dünnfilmmikroextraktion von iodierten Röntgenkontrastmitteln am Beispiel von Iohexol und möglicher Dehalogenierungsprodukte

Keil Benjamin M., Institut für Bodenkunde und Bodenerhaltung, IFZ für Umweltsicherung, Justus-Liebig-Universität Gießen

Leonard Böhm, Rolf-Alexander Düring, Institut für Bodenkunde und Bodenerhaltung, IFZ für Umweltsicherung, Justus-Liebig-Universität Gießen

Benjamin.M.Keil@umwelt.uni-giessen.de

Iodierte Röntgenkontrastmittel wie Iohexol sind in Abwässern, v.a. von Krankenhäusern, in signifikanten Konzentrationen nachweisbar und besitzen eine hohe Umweltrelevanz. Aufgrund ihrer hohen Polarität (log KOW 1,37 bis -3,05) und Persistenz passieren sie Kläranlagen nahezu ungehindert und sind daher ubiquitär in Fließgewässern und sogar Grundwasserleitern nachweisbar.

Im Hinblick auf eine mögliche Dehalogenierung der iodierten Röntgenkontrastmittel mit Pd(0)-Nanopartikeln war das Ziel dieser Studie die Entwicklung einer Dünnfilmmikroextraktionsmethode (Thinfilm microextraction TFME) zur Extraktion von iodierten Röntgenkontrastmitteln und ihrer möglichen Dehalogenierungsprodukte. Eine besondere Herausforderung stellte dabei die hohe Polarität von Iohexol (log KOW = -3,05) dar. Zum Einsatz kam daher eine TFME-Beschichtung aus Polystyrol-Divinylbenzol (PS-DVB) als möglichem Sorbens für polare Analyten.

Untersucht wurden der Einfluss von Sorptions- und Desorptionsdauer mit unterschiedlichen Analytkonzentrationen und unterschiedlichen Agitationsintensitäten während Sorption und Desorption, sowie verschiedene Elutionsmittel bei der Desorption. Auch wurden die Stabilität der PS-DVB-Beschichtung und die Wiederholbarkeit der Ergebnisse über die Versuche ausgewertet. Weiter wurde ein automatisiertes Verfahren für unterschiedliche Sorptions- und Desorptionszeitspannen der einzelnen Proben innerhalb eines Versuchs untersucht. Der Nachweis erfolgte mittels HPLC-MS.

Die Ergebnisse belegen die gute Eignung von PS-DVB für die Extraktion von Iohexol. Nach 120 min Adsorption und 60 min Desorption in Methanol konnten Wiederfindungsraten von über 80 % erreicht werden. Diese reduzierten sich jedoch durch Alterung des Sorbens und stabilisierten sich bei ca. 25 %. Durch eine geplante Veränderung der Beschichtung soll die Wiederfindungsrate konstant hoch gehalten werden.

Die Validierung der TFME-Methode erfolgte mittels solid-phase extraction (SPE) mit Isolute ENV+-Kartuschen. Weitere Versuche zur Überprüfung des Einflusses der Temperatur auf die Sorptions- und Desorptionskinetik sind geplant um den Extraktionsprozess weiter zu beschleunigen.

Erste Versuche zur Dehalogenierung von Iohexol durch Palladium-Nanopartikel führten zu einer signifikanten Reduktion von Iohexol, die erwarteten deiodierten Produkte konnten jedoch nicht nachgewiesen werden. Weitere Versuche sollen den Eliminierungsprozess klären und mögliche Abbauprodukte identifizieren.

Monitoring von Oberflächengewässern

P50. Determination of tributyltin in surface water systems

Richter Janine, Bundesanstalt für Materialforschung und -prüfung

Ina Fettig Rosemarie Philipp Christian Piechotta Norbert Jakubowski Ulrich Panne

Janine.richter@bam.de

The widespread of organotin compounds (OTC), used for example as pesticides, antifouling coatings and PVC stabilizers, results in an extensive input into the environment. OTC show toxic effects already at trace level. The public focus lies on the toxic and estrogenic effective tributyltin (TBT) and its metabolites. In 2000 the European water framework directive (WFD 2000/06/EC) was remitted to standardize the monitoring of aquatic ecosystems and ground water within the EU. The WFD aims to improve the quality of environmental waters and their sustainable usage. The claimed limit of quantification for TBT is 0.06 ng L-1 for the whole water body. A sensitive analytical method is required to achieve this demand.

The development of traceable measurement methods for monitoring TBT under the European water framework directive is presented. Therefore different extraction methods like liquid liquid extraction (LLE), solid phase extraction (SPE) and solid phase microextraction (SPME) were evaluated at ultra trace level. The extracts were analyzed using GC-ICP-MS. The quantification was realized by isotope dilution mass spectrometry (IDMS). The feasibility for detecting TBT in real water samples at the WFD concentration level will be demonstrated. Further results on stability and possible transformation processes of TBT and the influence of environmental factors like radiation, humic substances (HS) and suspended particulate matter (SPM) on the recovery will be presented.

P51. Investigations on biodiversity and ecosystem health of the river Nidda within the framework of the project 'NiddaMan'

Dieterich Andreas, Universität Tübingen

Mona Schweizer, Rita Triebskorn, Denise Brettschneider, Katharina Heye, Jörg Oehlmann, Matthias Oetken, Ulrike Schulte-Oehlmann, Uwe Kunkel, Arne Wick, Thomas Ternes, Heinz-Rüdiger Köhler

A-Dieterich@gmx.de

With almost 100 km length and a catchment of 2000 km², the river Nidda represents a great number of European streams. Being in its natural state near the spring in the Vogelsberg Mountains in Upper Hesse (Germany), the river Nidda rapidly changes its chemical and biological water status due to anthropogenic influences along its course. Discharges of local wastewater treatment plants, diffuse emissions of agricultural substances and substance inputs from tributaries as well as structural alterations are affecting the river on its way downstream until entering the river Main near Frankfurt (Germany).

The 'biodiversity and ecosystem health part' of the BMBF-project 'NiddaMan' investigates the ecological importance of diverse stress factors on the fish and invertebrate biocoenoses inhabiting the river Nidda catchment area. Laboratory and field studies will help to identify new biological parameters allowing for causal monitoring of deficient water conditions and the quantitative description of important organism-mediated ecosystem functions. Furthermore, efficiency and effects of water management measures will be examined. Samples from representative sites will be analysed chemically as well as biologically, considering e.g. genotoxic, cytotoxic, embryotoxic, estrogenic and anti-estrogenic effect potentials. Additionally active and passive monitoring approaches will complement our study. The overall aims of this project part are the development of strategies to counteract water quality impairment induced by environmental stressors and to support river ecosystem recovery.

Acknowledgement:

The project 'NiddaMan' is funded by the Federal Ministry for Education and Research (BMBF) within the ReWaM initiative under project code 02WRM1367.

P52. Vergleich neuartiger Geräte zur Schwebstoffgewinnung für das chemische Gewässermonitoring - SchwebSam

Wilcsek Kristiana, Universität Trier, Abteilung Hydrologie

Sabine Keßler (1), Thorsten Pohlert (2), Reinhard Bierl (1), Vera Breitung (2) (1) Universität Trier, Abteilung Hydrologie (2) Bundesanstalt für Gewässerkunde (BfG), Am Mainzer Tor 1, D-56068 Koblenz, Germany

wilcsek@uni-trier.de

In diesem Projekt wird die Vergleichbarkeit vier verschiedener Verfahren zur Entnahme von Schwebstoff für die Rückstandsanalytik untersucht. Dazu wurden zwei schwimmende Integralsammler, der BfG-Binnensammler und ein neuartiger Rohrsammler (modifiziert nach Phillips et al. (2000)) sowie zwei Geräte zur Gewinnung von Stichproben, die Durchflusszentrifuge und der Hydrozyklon, verwendet. An den Entnahmestellen Iffezheim/Rhein und Koblenz/Mosel wurden von August 2013 bis August 2014 im vier Wochen Abstand Schwebstoffproben gewonnen.

Die Gehalte der Schwebstoffe an organischem Kohlenstoff (TOC), Gesamtphosphor (GP), Blei (Pb), Zink (Zn), Kupfer (Cu), Nickel (Ni), Chrom (Cr) und Cadmium (Cd), des Korngrößenanteils < 63 µm sowie der Gehalte an polyzyklischen aromatischen Kohlenwasserstoffen (PAK), polychlorierten Biphenylen (PCB) und Hexachlorbenzol (HCB) wurden hinsichtlich des Einflusses der Entnahmetechnik (Sammelprobe, Stichprobe), des Entnahmegeräts (Binnensammler, Rohrsammler, Durchflusszentrifuge, Hydrozyklon) und der Messstelle (Iffezheim/Rhein, Koblenz/Mosel) mittels dreifaktorieller Varianzanalyse ausgewertet und diskutiert.

Bei den Konzentrationen von Pb, Cr, Cd und HCB wurde weder ein signifikanter Einfluss der Entnahmetechnik, noch des Entnahmegerätes festgestellt. Hinsichtlich GP, TOC und Cu zeigt sich die Entnahmetechnik als signifikante Einflussgröße. In den Stichproben werden höhere TOC-Gehalte beobachtet als in den Sammelproben, ein Effekt, der ggf. auf Abbauvorgänge während der Expositionsdauer zurückzuführen ist. Für Ni wurden signifikante Unterschiede bezüglich der Entnahmegeräte festgestellt. Die höchsten Gehalte sind wahrscheinlich durch Metallabrieb im Fuß- bzw. Kopflager der verwendeten Durchflusszentrifuge verursacht. Für die Kenngröße Zn hat das Entnahmegerät offensichtlich entscheidenden Einfluss auf das Ergebnis. Der Hydrozyklon liefert um etwa 40 mg/kg höhere Zn-Gehalte. Der Anteil der Korngrößenfraktion < 63 µm ist in der Durchflusszentrifuge am höchsten. Die PAK- und PCB-Konzentrationen waren in den Stichproben signifikant höher als in den Integralproben. Während die Integralsammler untereinander homogene Ergebnisse lieferten, zeigt der Hydrozyklon systematisch höhere Gehalte an PAK.

Die physikalischen Kenngrößen, Elementgehaltmessungen und Gehalte organischer Schadstoffe der integrativen Sammler (BfG-Binnensammler und Rohrsammler) sind stets vergleichbar. Verglichen mit der „Referenzmethode“ Durchflusszentrifuge ergeben sich signifikante Abweichungen lediglich hinsichtlich des Gehalts an TOC, GP und des Korngrößenanteils <63 µm. Sieht man von den Ergebnissen für die Korngrößenanteile < 63 µm und PAK ab, sind Hydrozyklon und Durchflusszentrifuge als gleichwertige Schwebstoffentnahmegeräte einzustufen.

P53. Biomonitoring mit Gammarus pulex im landwirtschaftlich intensiv belasteten Eschelisbach, Thurgau

Langer Miriam, Oekotoxzentrum EAWAG

Barbara Ganser Margie Koster Cornelia Kienle Etienne Vermeirssen Inge Werner

Miriam.langer@oekotoxzentrum.ch

In einer landwirtschaftlich intensiv genutzten Region des Bodenseeraums wird ein kleines Fliessgewässer, der Eschelisbach, im Rahmen des Schweizer NAWA Spez Projektes (Nationale Beobachtung Oberflächenwasserqualität - Spezialuntersuchungen) im Jahr 2015 chemisch engmaschig untersucht. Das Ziel ist es während des Frühlings und Sommers, in den Monaten mit den höchsten Pestizideinträgen, die Peak Konzentrationen von 300 ausgewählten Pestiziden in einem kleinem Fliessgewässer, dem Eschelisbach, zu evaluieren.

Um zusätzlich ökotoxikologische Effektdaten zu erhalten, wird im 2-wöchigen Takt die Mortalität und Frass Aktivität von Gammarus pulex in einem aktiven Biomonitoring untersucht. Zusätzlich sollen neben Genexpressions-Analysen auch die Hemmung der Acetylcholinesterase in den Gammariden gemessen werden.

In Wasser Mischproben werden ausserdem zur Detektion der östrogenen Aktivität ein YES Tests (Hefezell Östrogentest) und zur Detektion von Photosynthese-Hemmern ein kombinierter Algentests durchgeführt. Als Referenzstelle fungiert ein unbelasteter Bach ähnlicher Grössenordnung in der Nähe des Eschelisbaches.

P54. Aktives Monitoring zur Fischgesundheit in Fließgewässern am Beispiel der Steinlach

Jacob Stefanie, Universität Tübingen

Sabrina Wilhelm Rita Triebskorn

stefanie.jacob@student.uni-tuebingen.de

Es gibt eine Vielzahl an Faktoren, die sich auf die Gesundheit von Fischen in einem Fluss auswirken können. Kläranlagenabflüsse können Spurenstoffen enthalten, die während der Reinigung nicht entfernt werden können. Außerdem können Regenüberlaufbecken, Sickerwässer aus Mülldeponien und Pestizideinträge von Feldern mögliche Schadstoffquellen darstellen. Chemikalien mit toxischen Wirkungen können unter anderem Schäden in Geweben, im Erbgut oder im Nervensystem hervorrufen. Stoffe mit hormoneller Wirkung können zur Verweiblichung von männlichen oder zur Vermännlichung von weiblichen Fischen führen und so die Reproduktionsfähigkeit einschränken (Jobling et al., 1998).

In der aktuellen Studie wurde an der Steinlach bei Tübingen, einem Zufluss des Neckars, ein aktives Monitoring mit Regenbogenforellen (Oncorhynchus mykiss) durchgeführt, um den Einfluss einer Kläranlage (KA Dußlingen) auf die Fischgesundheit nachzuweisen. Hormonelle und toxische Einflüsse auf die Fische wurden mittels einer Biomarkerbatterie (Histopathologie von Lebern und Gonaden, Mikrokerntest, Vitellogenin-Induktion) untersucht. Jeweils 20 Fische wurden für circa sieben Wochen in zwei Käfigen oberhalb und unterhalb des Abflusses der Kläranlage gehalten. Als Kontrollen dienten Fische aus einer Forellenzucht.

Bei der histopathologischen Betrachtung von Lebern und Gonaden konnte kein signifikanter Unterschied zwischen den Forellen, die in den Käfigen oberhalb und unterhalb der Kläranlage gehalten wurden, und der Kontrolle festgestellt werden. Somit ist eine gewebstoxische Wirkung in der Steinlach nicht nachweisbar. Die Anzahl der gefundenen Mikrokerne lag sowohl bei den Kontrollfischen als auch bei den Fischen aus den Käfigen innerhalb des Bereiches der natürlichen Variabilität. Es kann davon ausgegangen werden, dass keine genotoxischen Stoffe in der Steinlach vorkommen, die zu einer erhöhten Anzahl an Mikrokernen führen könnten. Auch bei der Untersuchung der Vitellogenin-Induktion gab es keinen signifikanten Unterschied zwischen den Fischen, die in den Käfigen oberhalb und unterhalb der Kläranlage gehalten wurden, und den Kontrollfischen. Das lässt darauf schließen, dass es keine östrogene Wirkung in der Steinlach gibt.

Abschließend ist zu sagen, dass die Steinlach ein relativ unbelastetes Gewässer ist und die Fischgesundheit nicht signifikant beeinträchtigt wird.

Toxizität von Chemikalienmischungen

P55. Risk of herbicide mixtures as a key parameter to explain phytoplankton fluctuation in a great lake: the case of Lake Geneva, Switzerland

Chèvre Nathalie, Université de Lausanne

Lucie Büchi Orlane Anneville Frédéric Rimet Agnès Bouchez Vincent Gregorio

nathalie.chevre@unil.ch

It is now recognised that chemicals detected in the environment may act together and that mixture effects should be taken into account in ecological risk assessment. However, to date, very few approach were proposed to reach this goal. In ecotoxicology, mixture effects were largely influenced by toxicological theory. Basically, two models are used to assess the mixture effects: the concentration addition and the independent action model. Synergisms and antagonisms effects are generally neglected, as they are hard to predict. For ecological risk assessment, these two models can be combined either with the classical safety factor method or with the species sensitivity distribution curves. Such an approach is however poorly applied until now, mainly for two reasons: the lack of data needed (ecotoxicological data, mode of action of the chemicals), and the lack of validation of the approach.Indeed, mixture risk assessment predictions have rarely been confronted with biological changes observed in the environment. In this study, long-term monitoring of a European great lake, Lake Geneva, provides the opportunity to assess to what extent the predicted toxicity of herbicide mixtures explains the changes in the composition of the phytoplankton community next to other classical limnology parameters such as nutrients. To reach this goal, the gradient of the mixture toxicity of 14 herbicides regularly detected in the lake was calculated using concentration addition and response addition models. A temporal gradient of toxicity was observed which decreased from 2004 to 2009. Redundancy analysis and partial redundancy analysis showed that this gradient explains a significant portion of the variation in phytoplankton community composition with and without having removed the effect of all other covariables. Moreover, species that are significantly influenced, positively or negatively, by the decrease of toxicity in the lake over time are highlighted. It can be concluded that the herbicide mixture toxicity is one of the key parameters to explain phytoplankton changes in Lake Geneva.

P56. On the appropriateness of using the common mixture toxicity models CA and IA on species sensitivity distributions: a theoretical approach

Chèvre Nathalie, Université de Lausanne

Vincent Gregorio Marion Junghans

nathalie.chevre@unil.ch

Environmental species are not only exposed to single substances but typically to mixtures. Therefore it is of great importance to assess the ecological risk not only substance by substance but also for mixtures of compounds. Two models are generally used to predict mixture toxicity: the concentration addition (CA) (or dose addition) and the response addition (RA) (or independent action) models. Their adequacy to predict mixture toxicity of similarly acting compounds (CA) and dissimilarly acting compounds (RA) for single species was already shown during the last decades. To assess the risk of chemical mixtures to ecosystems, these two models have also been proposed to be applied directly on species sensitivity distribution (SSD) curves to derive a multi substances potentially affected fraction of species (msPAF). Usually, this is done in two steps: (i) the CA model is applied on individual SSDs of similarly acting compounds and then (ii) the RA model is applied on the predicted SSDs by CA between groups of similarly acting compound. However, as mentioned above the validity of mixture models were shown only for single species when applied on single dose-response curves and not on multiple species SSDs. For a validation of this approach, one should therefore first apply the mixture models for each species, then construct the mixture SSD to calculate the msPAF and compare it with a msPAF derived by applying the mixture models directly on the SSDs. This approach is however rarely done because the information on the whole dose-response curves of each single species for all substances is needed and this information is poorly available. In this study, we highlighted the difference between the two methodologies (SSD combination with CA/RA models or single species mixture assessment followed by SSD construction) to derive a msPAF. To compare the two methods we calculate msPAF with different cases, varying the EC50s and the slopes of the individual dose-response curves. The results show that the predictions with CA are quite similar between the two methods and small differences can be explained by the goodness of fit of SSDs and the species tolerance correlation among substances. However, risk underestimation are observed with large number of substances or with SSD built based on substances with flat dose-response curves. The application of RA may result in a differing msPAF between the two methods also when the individual dose-response curves are flat. Finally, we define cases in which the two methodologies give similar results and for which the use of CA and RA directly on SSDs to predict the risk of mixture compounds is appropriate.

P57. Umweltrisikobewertung von Chemikalienmischungen in verschiedenen regulatorischen Zusammenhängen

Coors Anja, ECT Oekotoxikologie
a.coors@ect.de

Das Thema “Mischungstoxizität” wird seit vielen Jahren intensiv in der Ökotoxikologie bearbeitet. Inzwischen ist die Berücksichtigung von Mischungstoxizität in einigen regulatorischen Zusammenhängen (u.a. der europäischen Verordnung zur Zulassung von Biozidprodukten und den Leifäden zur Umweltrisikobewertung von Pflanzenschutzmitteln) gefordert. In anderen Bereichen (zB. der Umweltrisikobewertung von Arzneimitteln) wird die Mischungstoxizität in der wissenschaftlichen Literatur thematisiert, ist aber noch nicht regulatorisch verankert worden. Ein weiteres Beispiel sind Abläufe von Kläranlagen, über die Substanzen als Mischung in die Umwelt entlassen werden, die einzeln in z.T. verschiedenen Gesetzen und nach unterschiedlichen Anforderungen reguliert sind. Das Poster wird einen Überblick geben zur Umweltrisikobewertung von Mischungen in verschiedenen regulatorischen Zusammenhängen. Wesentliche Fragen bei der Berücksichtigung der Mischungstoxizität in der Umweltrisikobewertung werden exemplarisch erläutert am Beispiel von Forschungsprojekten der letzten Jahre im Bereich Pflanzenschutzmittel, Biozidprodukte und Kläranlagenabläufe. Zu diesen Fragen zählt u.a. die Definition der zu bewertenden Mischung, die Abgrenzung von Synergismus und Antagonismus sowie die Folgen der Mischungsberücksichtigung in der Umweltrisikobewertung.

P58. Effect of Al2O3 nanoparticles on the toxicity of the neonicotinoid insecticide thiacloprid

Lorenz Carla S., Universität Tübingen

Leonie Jäger, Leyla Guluzada, Leilei Luo, Yucang Liang, Reiner Anwander, Stefan B. Haderlein and Heinz-R. Köhler

lorenz.carla@web.de

Artificial particles in the nanoscale become more and more a topic in ecotoxicology, since their usage has increased during the last years tremendously. However, the knowledge about the fate and interactions of nanoparticles in the environment is still scarce. As a contribution to fill this gap of knowledge, the objective of this study was to investigate how the toxicity of the neonicotinoid insecticide thiacloprid is altered by Al_2O_3 nanoparticles. Effects were examined in the non-biting midge Chironomus riparius, a well-established study organism in ecotoxicology. Tests with thiacloprid and nanoparticles, as single substances, were conducted, followed by experiments with mixtures of them. For the mixture experiments one may hypothesize that nanoparticles can cause, for example, mechanical stress, or they may increase the uptake of thiacloprid, resulting in (more-than-) additive toxicity of nanoparticles and thiacloprid. Another possible mixture effect could be that the nanoparticles influence the uptake of thiacloprid negatively, resulting in weaker toxicity of the insecticide. Interestingly, our results deriving from an acute toxicity test clearly showed the mortality of fourth instar larvae of C. riparius to be significantly reduced at given concentrations of thiacloprid, if Al_2O_3 nanoparticles were present. However, mechanistical explanations for this decreased mortality remain unclear. Until now, sorption of thiacloprid to the Al_2O_3 nanoparticles can be excluded. Consequently, further research is needed to answer this question. Moreover, the induction of the stress protein hsp70 will be measured, and a sediment-water chironomid toxicity test using spiked sediment (OECD 218) will be conducted to determine how the presence of Al_2O_3 nanoparticles affects other endpoints in the life cycle of C. riparius like, e.g., the emergence rate or weight gain.

P59. Biotransformation ausgewählter Azolfungizide im Flohkrebs Gammarus pulex und deren Wirkung in Fungizidmischungen

Rösch Andrea, Eawag/ETH Zürich

Sabine Anliker, Juliane Hollender

andrea.roesch@eawag.ch

Schadstoffe, wie zum Beispiel Pestizide, sind allgegenwärtig in aquatischen Ökosystemen und ein gemeinsames Auftreten verschiedener Pestizide wird häufig beobachtet.

Eine Verbindungsklasse von Fungiziden sind Azole. Dazu gehören unter anderem Triazole und Imidazole. Ihr Wirkungsmechanismus in Pilzen beruht auf der Hemmung der Ergosterol-Biosynthese. In Nicht-Zielorganismen können Azole die Wirkung anderer Pestizide erhöhen, da sie die Cytochrom-P450-Monooxygenasen hemmen, die eine entscheidende Rolle bei der Metabolisierung und somit Entgiftung von Schadstoffen spielen. Die Bindung zwischen Azol und Enzym erfolgt über den nukleophilen Stickstoff im Azolring und das Eisen im Enzym. Folglich können Biotransformationsreaktionen, die am Azolring stattfinden, einen wesentlichen Einfluss auf die Enzymhemmung haben.

In der vorliegenden Arbeit wurden verschiedene Triazol- (Cyproconazol, Epoxiconazol, Fluconazol, Propiconazol, Tebuconazol) und Imidazolfungizide (Ketoconazol, Prochloraz) ausgewählt, um deren Biotransformation in Gammarus pulex zu untersuchen.

Dazu wurden G. pulex für 24 Stunden Fungizidkonzentrationen von 200 µg L^{-1} ausgesetzt. Zur Bestimmung von Aufnahme-, Biotransformations- und Eliminationsraten wurde ein toxikokinetisches Experiment durchgeführt. Um die Enzymhemmung zu beurteilen, wurden G. pulex Fungizidmischungen ausgesetzt, die aus Azoxystrobin und jeweils einem der ausgewählten Azole zusammengesetzt waren. Die Proben wurden mittels Online-Festphasenextraktion gekoppelt mit Flüssigchromatographie und hochauflösender Tandem-Massenspektrometrie gemessen. Die Identifizierung der Biotransformationsprodukte (BTP) erfolgte über Isotopenmuster und über die Interpretation von MS/MS-Spektren.

Verschiedene Biotransformationsreaktionen traten auf, überwiegend Oxidations- und Konjugationsreaktionen. Die häufigste Reaktion war eine Hydroxylierung am Azolring oder am aliphatischen Teil des Moleküls. Ringspaltungen und Ringverluste wurden nur für Imidazole beobachtet. Generell fanden Biotransformationsreaktionen selten am Azolring statt, sodass das aktive Zentrum der Fungizide erhalten blieb. Jedoch waren die gemessenen internen BTP-Konzentrationen gering, was sich auch in den kleinen Biotransformationsraten wider spiegelte. Dies weist darauf hin, dass die gebildeten BTP bezüglich der Reduzierung der Bioakkumulation sowie bezüglich der Enzymhemmung von untergeordneter Bedeutung sind.

Eine starke hemmende Wirkung wurde für die Fungizidmischung bestehend aus Azoxystrobin und Prochloraz beobachtet, ausgedrückt in geringeren BTP-Konzentrationen und erhöhter Mortalität im Vergleich zur alleinigen Exposition zu Azoxystrobin.

Obwohl die in dieser Studie eingesetzten Konzentrationen im Vergleich zu Umwelt-konzentrationen relativ hoch waren, ist es wahrscheinlich, dass ein gemeinsames Auftreten von Azolen mit anderen Schadstoffen zu einer erhöhten Toxizität von bereits stark toxischen Verbindungen führen kann.

Risikobewertung Pestizide

P60. Exposure profiles in aquatic mesocosm risk assessment

Ernst Johannes, ecotox consult , Hochschule RheinMain

Jan Philip Nickel (ecotox consult) Lara Petschick (ecotox consult) Renja Bereswill (ecotox consult) Michael Meller (ecotox consult) Reinhard Debus (Hochschule RheinMain)

j-ernst@ecotox-consult.de

The use of FOCUSsw-modeled exposure profiles in mesocosm-based aquatic risk assessment - triggered by the new EU guidance on tiered risk assessment for plant protection products for aquatic organisms in edge-of-field surface waters (EFSA Journal 2013;11(7):3290) - is investigated. This project aims to develop applicable methods for the implementation of new regulatory requirements regarding the linking of mesocosm effect studies with exposure profiles.

The work relies on an extensive database of mesocosm studies covering substances with diverse properties to ensure the relevance for the regulatory practice. For each substance, results from mesocosm studies with respect to effect and fate are compared with the respective FOCUSsw-modeled exposure data.

The arising questions can be split in two main issues:

(1) For the derivation of a Regulatory Acceptable Concentration (RAC), the EU guidance requires to verify whether the rate of dissipation of the active substance in the test system is realistic to worst case compared to that predicted for the field. The selection of adequate dissipation data from a mesocosm study or the assessment of multiple peaks in an exposure profile are discussed. Possibilities to characterize and compare measured mesocosm exposure and the predicted exposure profiles are investigated, e.g. using adequate parameters such as area under the curve or peak size and width.

(2) For using the Ecological Recovery Option (ERO) to derive a RAC, the recovery of the ecosystem has to be verified in order to upgrade a provisional ERO-RAC to an official ERO-RAC. Therefore the EU guidance proposes to evaluate the exposure profile focusing on peaks exceeding the Ecological Threshold Option (ETO)-RAC but not the ERO-RAC. The independence of peaks and the adequate recovery time span are discussed as well as further questions, e.g. the assessment of successive exposure peaks not addressed in the exposure regime of the mesocosm design.

The different approaches are evaluated to develop a scientific sound strategy for mesocosm-based higher-tier risk assessments to meet the regulatory requirements of the new EU guidance.

P61. Aquatische Risikobeurteilung von PSM – Der agronomische Unsicherheitsfaktor

Fulda Beate, Agroscope

Aldrich Annette, Balmer Marianne, Poiger Thomas

beate.fulda@agroscope.admin.ch

Im Rahmen der Umweltrisikobeurteilung von Pflanzenschutzmitteln (PSM) wird der Austrag von PSM von landwirtschaftlichen Flächen in angrenzende Oberflächengewässer abgeschätzt. In der Schweiz wird für die Berechnung des potentiellen PSM Austrags durch Abschwemmung und Drainage derzeit das aquatische Expositionsmodell EXPOSIT 3.01 (Umweltbundesamt UBA, DE) verwendet. Bei diesem Modell ist die geschätzte Konzentration eines PSM Wirkstoffes im Gewässer (predicted environmental concentration, PEC) abhängig von dessen Wirkstoffeigenschaften (DT50 Boden, Koc) sowie der landwirtschaftlichen Praxis, d.h. Zeitpunkt und Häufigkeit der Applikation, Zeitintervall zwischen den Applikationen sowie Kulturstadium/Bodendeckung zum Zeitpunkt der Applikation. In vielen älteren Bewilligungen wurden diese agronomischen Parameter oftmals nicht klar festgelegt, sodass in der Praxis ein gewisser Anwendungsspielraum besteht. Die für einen Wirkstoff berechneten PEC Werte können daher je nach angenommenen Anwendungsszenario teilweise stark variieren. In dieser Studie wird beispielhaft für verschiedene Wirkstofftypen gezeigt, wie sich Unsicherheiten in den agronomischen Inputparametern auf die berechneten PEC Wert auswirken können. Diese Unsicherheiten sind insbesondere relevant, wenn das Expositionsmodell mittels Daten aus dem Gewässermonitoring evaluiert werden soll. Damit aus dem Vergleich von gemessenen und berechneten Gewässerkonzentrationen Rückschlüsse auf die Risikobeurteilung möglich sind, ist eine gute Kenntnis der landwirtschaftlichen Praxis notwendig.

P62. Pflanzenschutzmittel: Mögliche Risikominderungsmassnahmen für Vögel

Gauch Roger, Agroscope

Daniel, Otto

roger.gauch@agroscope.admin.ch

Um ein akzeptables Risikopotential für Lebewesen ausserhalb der Pflanzenkultur zu erreichen, muss bei der Anwendung von Pflanzenschutzmittel (PSM) je nach Risiko ein Abstand zu Gewässern und Biotopen eingehalten werden. Diese Risikominderungsmassnahme (RMM) hat den Vorteil, dass sie auf die meisten PSM und Kulturen anwendbar ist und die Risikominderung quantifiziert werden kann.

Für mobile Lebewesen wie Vögel, für die auch ein potentielles Risiko durch eine PSM-Exposition innerhalb der Kultur bestehen kann, ist es schwieriger, geeignete Risikominderungsmassnahmen zu finden, die sowohl quantifizierbar als auch in einer grossen Bandbreite an PSM und Kulturen anwendbar wären. Die meisten derzeit in den nationalen Zulassungen von EU-Staaten verwendeten RMM beschränken sich auf Saatbeizmittel, Granulate, Pellets, Schneckenkörner und Rodentizide.

Mögliche RMM für Vögel wurden durch Literatursichtung und Expertenbefragung gesucht und kategorisiert. Die RMM wurden anschliessend bewertet bezüglich ihrer Umsetzbarkeit und ihres Wirkungspotenzials. Die vielversprechendsten Massnahmen werden präsentiert.

P63. Ausgewählte zeitliche Abfolgen von Pflanzenschutzmittel-Anwendungen: Vergleich mit Zulassungsparametern

Kohler Esther, Agroscope, Institut für Pflanzenbauwissenschaften IPB, 8820 Wädenswil, Schweiz

Laura de Baan, Annette Aldrich, Otto Daniel

esther.kohler@agroscope.admin.ch

Um das ökotoxikologische Risiko von Pflanzenschutzmitteln (PSM) beurteilen zu können, sind Informationen zur Anwendungspraxis nötig. Bei der Definition der guten landwirtschaftlichen Praxis werden Faktoren wie die Variabilität der Standorte, angebaute Sorten, Schädlingsdruck etc. berücksichtigt. In der Zulassung werden dann Anwendungsparameter und Auflagen festgelegt, welche eine landwirtschaftliche Produktion ermöglichen und gleichzeitig die Risiken von Mensch und Umwelt im akzeptablen Bereich halten sollen.

Die Anzahl wiederholter Applikationen des gleichen PSM-Produktes, Dauer der Spritzintervalle, Behandlungszeitpunkte und Aufwandmengen haben einen grossen Einfluss auf die berechneten Wirkstoffkonzentrationen in der Umwelt und somit auf das Ergebnis der Risikobeurteilung.

Im Rahmen des Agrarumweltmonitorings werden in der Schweiz seit 2009 jährlich Daten zum PSM-Einsatz von rund 300 Betrieben erfasst. Die Feldkalendereinträge liefern parzellenspezifische Informationen zu den verwendeten PSM-Produkten, Aufwandmengen, behandelten Kulturen und Anwendungszeitpunkten. Basierend auf dieser Datengrundlage wurden wiederholt in einer Kultur gespritzte PSM analysiert und je Wirkstoff die Aufwandmengen, zeitlichen Abstände und Anzahl der verschiedenen PSM Anwendungen bestimmt. Diese Daten aus dem Agrarumweltmonitoring werden mit den Angaben in der Zulassung verglichen.

Der Vergleich erlaubt eine Darstellung der zeitlichen Variabilität der Anwendungsparameter und eine Abschätzung inwiefern die in der Zulassung festgelegten Parameter den in der Praxis üblichen Parametern entsprechen.

P64. Risikobewertung für den Einsatz von Pflanzenschutzmitteln im Forst

Lautenschlager Karin, Agroscope Wädenswil
Laura de Baan, Annette Aldrich, Otto Daniel

Karin.Lautenschlager@agroscope.admin.ch

In der Forstwirtschaft werden Pflanzenschutzmittel (PSM) hauptsächlich angewandt, um Holzpolter vor holzschädigenden Insekten (beispielsweise Nutzholzborkenkäfern) zu schützen. Hierfür müssen in der Schweiz die Forstämter eine Ausnahmebewilligung bei den zuständigen Kantonen beantragen. Für den Gebrauch sind mehrere Produkte mit den Wirkstoffen Cypermethrin, Alpha-Cypermethrin, Zeta-Cypermethrin, Deltamethrin, Chlorpyrifos und Lambda-Cyhalothrin zugelassen. Um Oberflächengewässer vor dem Eintrag der Produkte durch Drift und Abschwemmung zu schützen, müssen Abstände von 20 - 50 m zu Oberflächengewässern eingehalten werden.

Für die ökotoxikologische Risikobewertung für die Zulassung von PSM ist das Wissen über die Anwendung zentral. In dieser Studie wurden die tatsächlich angewendeten Mengen an PSM im Forst eines Schweizer Kantons evaluiert. Hierzu wurden von über 250 PSM-Behandlungen im Jahre 2014 Informationen zur PSM Menge, behandelter Holzmenge und Koordinaten der Holzpolter ausgewertet. Anhand der Daten wurde eine ökotoxikologische Risikobewertung für aquatische Organismen durchgeführt und diskutiert. Mittels GIS Analysen wurden zu dem die Abstände von den mit PSM behandelten Holzpoltern zu den nächstgelegenen Oberflächengewässern ermittelt.

Aus der Studie können wertvolle Rückschlüsse zur Weiterentwicklung von Risikominderungsmassnahmen für den Forstbereich gezogen werden.

Multiple Stressoren

P65. Can highly stenotherm Antarctic fish cope with anthropogenic pollutants in a warming Southern Ocean?

Strobel Anneli, Universität Basel, MGU

Patricia Holm, Helmut Segner

anneli.strobel@unibas.ch

Climate change and anthropogenic pollution are of increasing concern also in remote areas such as Antarctica. Importantly, future climate warming is expected to increase the levels of contaminants in the Southern Ocean. The extreme stenothermy of Antarctic notothenioid fish led to a limited physiological capacity to deal with altered environmental conditions, such as increasing seawater temperature or anthropogenic pollution.

To determine the potential toxicity of organic pollutants for Antarctic fish, we analyzed species differing in trophic position and physiology (red-blooded: Notothenia rossii, Gobionotothen gibberifrons, white-blooded: Chaenocephalus aceratus & Champsocephalus gunnari), and compared them to rainbow trout (Oncorhynchus mykiss). We assessed hepatic biotransformation capabilities by measuring actual rates of xenobiotic metabolism in vitro in liver S9 fractions. In addition, we measured the thermal sensitivity of enzymes involved in xenobiotic metabolism, namely, ethoxyresorufin-O-deethylase and glutathione-S-transferase, at rising assay temperatures.

Our results revealed similar metabolic enzyme activities and metabolic clearance rates between red- and white-blooded Antarctic fish, but significantly lower rates in comparison to rainbow trout. Furthermore, our data showed no capacity of Antarctic fish to increase their biotransformation enzyme activities with warmer temperatures. Such low detoxification enzyme activities and the limited thermal plasticity of those enzymes will consequently result in a relatively higher bioaccumulation of xenobiotics in the tissues of Antarctic fish compared to temperate species.

As a consequence, bioaccumulation factors for metabolizable lipophilic contaminants may be higher in Antarctic than in temperate fish. Furthermore, increasing water temperatures in the Southern Ocean will additionally compromise the low detoxification capacities of Antarctic fish.

P66. Acclimation of Chlamydomonas reinhardtii to ultraviolet radiation and its impact on chemical toxicity

Korkaric Muris, Eawag

Mao Xiao, Renata Behra, Rik I.L. Eggen

Korkaric@googlemail.com

Aquatic organisms are frequently exposed to various chemical and environmental stressors, such as ultraviolet radiation (UVR), potentially causing so-called multiple stressor effects. The temporal dynamics of multiple stressor exposure in the aquatic environment have rarely been addressed, and interactions have predominantly been studied in simultaneous stressor exposure experiments. However, in the environment, organisms can acclimate to stressful environmental conditions, which might decrease or increase their tolerance (co-tolerance) towards chemical pollutants. It has been hypothesised that co-tolerance might be expected for similar acting stressors due to the action of common defense mechanisms. To test this, the green alga Chlamydomonas reinhardtii was used to assess whether pre-acclimation to UVR exposure had an influence on its tolerance towards chemical stressors of photosynthesis, namely rose bengal, paraquat and diuron. Results showed that during prolonged UVR exposure, growth and photosynthesis of C. reinhardtii were partially inhibited, and that algae quickly increased tolerance to more intense UVR exposure. The observed UVR acclimation might in part be explained by an increase in the cellular content of lutein, a photoprotective xanthophyll, as shown by HPLC analysis of algal pigments. UVR acclimated algae were found to be more tolerant to rose bengal, whereas paraquat and diuron toxicity were unaffected by previous UVR exposure. These results suggest a common involvement of singlet oxygen in the effects of both UVR and rose bengal. Moreover, UVR acclimation appears to rely, in part, on the action of fast and reversible mechanisms related to single oxygen defense. Overall, this study highlights that in order to predict the impact of environmental and chemical stressors on aquatic organisms, temporal dynamics of multiple stressors have to be considered and knowledge of the molecular toxicity mechanisms of stressors is needed.

Endokrine Disruption

P67. Estrogens from Non-Point Sources in Swiss Surface Water

Gattiker Etienne, Swiss Federal Institute of Technology Zurich (ETHZ)

Dr. Robert Kase, Swiss Centre of Applied Ecotoxicology Dr. Michael Sander, Institute of Biogeochemistry and Pollutant Dynamics ETHZ

e.gattiker@gmx.ch

The aim of this study was to review information on estrogenic compounds in Swiss surface waters originating from non-point sources and to evaluate if their input can be estimated and a risk assessment is currently feasible.

In vitro and in vivo potencies of steroidal estrogens, phyto-mycoestrogens and pesticides were derived by the ratio of EC50 and LOEC values, respectively. The natural estrogen load, expressed as 17β-estradiol-equivalents (EEQ), was calculated for human and livestock using estrogen excretion rates, relative in vivo potencies and population data (52.5-18.4 and 821.6-1273.5 kgEEQ/a). The estrogen input of wildlife could not assessed due to insufficient population data or estrogen excretion rates. The estrogenic load originating from assessed pesticides was calculated based on sale volumes and relative potencies (7.1 kgEEQ/a).

The EEQ's originating from phyto- and mycoestrogens could not derived but were mainly estimated to 1644.5.-16719.0 and 555.6-1740.6 kgEEQ/a respectively. Sorption in soil and sediment was assessed by Koc values. Mineralisation half-live values (T½) are calculated from reviewed data assuming a first-order kinetic. Estrogens are fast degraded (<2d) but complete mineralisation is slow (T½, E2: 30-551 d, T½, E1: 35-636 d).

The fate of estrogens in soil is complex and sorption behaviour or degradation pathways are not fully elucidated yet. The whole fraction of (xeno)estrogen lost from soil to surface water was estimated and following the input in Swiss surface water was evaluated for point and non-point sources (5.5-18.3 and 4.6-9.5 kgEEQ/a). Biodegradation in water (T½, E2: 0.1-23.4 d, T½, E1: 0.08-3.47 d) is faster compared to sediment under aerobe (T½, E2: 0.74-7.1 d, T½, E1: 15.2-40.1 d) or anaerobe conditions (T½, E2: 0.37-45.5 d, T½, E1: 34.1-80.9 d).

Sediment may act as a long-term reservoir whereas sediment-borne exposure and effects must be further investigated. The high spatial and temporal variations of input and dilution impede a risk assessment. Midsize rivers in intense agricultural areas with mainly rain-fed run-off are probably affected by non-point sources.

To detect risk from estrogenic sources integrative monitoring tools like receptor activating bioassays are valuable to screen sediment and surfaces water. If this option would be applied more systematically important info is provided if more need for action is necessary to reduce estrogenic input from non-point sources.

P68. Bisphenol A-freie Babyflaschen: Eine Quelle Endokriner Disruptoren?

Jeckel Nina, Goethe-Universität Frankfurt am Main

Martin Wagner, Aquatische Ökotoxikologie, Goethe-Universität Frankfurt am Main, wagner@bio.uni-frankfurt.de

nina.jeckel@gmx.de

Hintergrund: Im Jahr 2011 wurde die Herstellung und Einfuhr Bisphenol A-haltiger Babyflaschen in den Mitgliedsstaaten der Europäischen Union untersagt (Richtlinie 2011/8/EU). Seitdem sind zahlreiche Ersatzprodukte verfügbar, die kein Bisphenol A (BPA) enthalten. Dennoch kann nicht ausgeschlossen werden, dass BPA-freie Ersatzpolymere andere, bisher nicht untersuchte Endokrine Disruptoren enthalten.

Zielsetzung: Das Ziel dieser Studie ist es deshalb, das Auslaugen Endokriner Disruptoren aus BPA-freien Babyflaschen zu untersuchen. Um unbekannte endokrin-wirksame Chemikalien sowie deren Cocktaileffekte detektieren zu können, wurde ein Bioassay-basierter Ansatz angewendet, bei dem die endokrine Aktivität charakterisiert wird, die aus Babyflaschen freigesetzt wird.

Methoden: Untersucht wurden Flaschen aus Polypropylen, Polyethersulfon, Polyamid und Copolyester (TritanTM). Die Produkte wurden einem Migrationsversuch mit Wasser unterzogen. Zudem wurden sie Stressfaktoren wie Hitze oder UV-Licht ausgesetzt und anschließend mit unterschiedlichen Lösemitteln extrahiert. Die aus den Versuchen gewonnenen Proben wurden mit Hilfe von Hefe-basierten Reportergen-Assays auf östrogene, anti-östrogene und anti-androgene Aktivität untersucht.

Ergebnisse und Diskussion: Erste Ergebnisse dieser Studie weisen darauf hin, dass BPA-freie Babyflaschen endokrin-wirksame Chemikalien enthalten können. Im zehntägigen Migrationsversuch wurden verschiedene endokrine Aktivitäten nachgewiesen: Wasserproben aus Polyethersulfon- und Polyamidflaschen wiesen östrogene Aktivität auf. Im Fall einer Polypropylenflasche konnte das Auslaugen von Chemikalien mit anti-androgener Aktivität detektiert werden.

Weiterhin wurden Flaschen mit Methanol befüllt und in einem Ultraschallbad behandelt. Bei diesen Proben konnte in Hefe-basierten Reportergen-Assays kein Auslaugen endokrin-wirksamer Chemikalien nachgewiesen werden. Dies deutet darauf hin, dass die im Migrationsversuch detektierten Substanzen sehr hydrophil sind und sich nicht mit mittelpolaren Lösemitteln extrahieren lassen. Weitere Migrations- und Stressversuche werden zurzeit durchgeführt, um den Einfluss von Versuchsdauer, Intensität des zugefügten Stresses und Art des Lösemittels auf das Auslaugen Endokriner Disruptoren aus den untersuchten Babyflaschen genauer bestimmen zu können.

Schlussfolgerung: Diese ersten Erkenntnisse belegen, dass BPA-freie Babyflaschen keineswegs frei von Endokrinen Disruptoren sind. Diese laugen unter realistischen Bedingungen aus und sollten in zukünftigen Studien chemisch und toxikologisch charakterisiert werden.

P69. Miniaturization of a reporter gene cell-based anti-androgenicity assay for effect-directed analysis of surface water sample

Kaufmann Kristina, RWTH-Aachen Institute for Environmental Research

Carolina Di Paolo, Matthias Muschket, Jochen Kuckelkorn, Henner Hollert, Thomas-Benjamin Seiler

kristina.kaufmann@rwth-aachen.de

In times of steadily rising pollution of aquatic ecosystem particular attention has to be paid to endocrine disruptors that can influence whole endocrine processes like organism development or reproduction processes. So far, studies have focused mostly on compounds that have estrogenic activity. However, the necessity and interest to investigate compounds that affect the androgenic hormone system are increasing. The investigation of antiandrogenic compounds in surface waters is important to protect aquatic organisms and humans against negative effects on their endocrine system, fertility or offspring. Antiandrogenicity in environmental samples is often caused by unknown or non-target compounds. In this way, there is need to improve the investigation of antiandrogenicity in effect-directed analysis (EDA) in order to identify substances that are not routinely monitored by chemical analysis methods or even unknown bioactive compounds. In a previous investigation, antiandrogenicity was identified in a surface water sample of the Saale/Mulde chatchment. The present study, implemented within the EDA-EMERGE project, aimed to develop and apply a miniaturized biotesting strategy for the EDA of this antiandrogenic sample. The MTT and the antiAR Calux® (BDS) assays were miniaturized in order to reduce the volume of sample and fractions required for biotesting and to achieve a higher throughput of the test system. The miniaturization was implemented by reducing the medium volume during the exposure step and by adapting the dosing method. Comparing to the standard bioassays version, the miniaturized antiAR assay provided highly comparable results for the analysis of known antiandrogenic compounds (flutamide, 1-hydroxypyrene, bisphenol A) and the bioactive surface water sample. Currently, the miniaturized bioassay is being applied to evaluate the fractions of the surface water sample. In future studies, the miniaturized bioassay can be implemented in effect directed analysis and biotesting of environmental samples or compounds.

P70. Validierung und Anwendung eines zellkultur-basierten Assays für Screening von endokrin wirksamen Stoffen in Abwasser

Kroller Theophil, Institut Biochemical Engineering, Fachhochschule Technikum Wien

Norbert Kreuzinger, Institut für Wassergüte, Ressourcenmanagement und Abfallwirtschaft, Technische Universität Wien; Dominik Rünzler, Institut Biochemical Engineering, Fachhochschule Technikum Wien

kroller@technikum-wien.at

In den letzten Jahrzehnten gab es bedeutende Fortschritte in der Beseitigung von Verunreinigungen im Abwasser durch Kläranlagen. Ein im Bereich Kläranlagen immer wichtiger gewordenes Thema stellen sogenannte Mikroverunreinigungen dar, die ihren Eintrag unter anderem in Form von Medikamenten, Pflanzenschutzmitteln oder Industriechemikalien finden. Diese Arbeit beschäftigt sich mit einer Gruppe dieser Mikroverunreinigungen, den hormonell aktiven Mikroverunreinigungen, im Besonderen mit der Detektion von Östrogenen. Östrogene stellen eine besondere Belastung für aquatische Ökosysteme dar, so kann es unter anderem zur Verweiblichung von Fischpopulationen kommen. Das Institut für Wassergüte der Technischen Universität Wien betreibt derzeit eine Versuchskläranlage mit dem Zweck verschiedene technische Maßnahmen zum Abbau hormonell aktiven Mikroverunreinigungen zu vergleichen. Durch chemische Analytik können zwar bekannte Stoffe einzeln im Abwasser quantifiziert werden; biologische Assays ermöglichen jedoch die Messung eines Gesamteffekts zum Beispiel aus Östrogen und Östrogen-ähnlichen Stoffen ohne Anreicherungs- und Trennungsschritte. Beim hier eingesetzten in vitro Assay wurde die 2004 von V. Wilson stabil mit einem Luciferase Reportersystem für Östrogen transfektierte humane Brustkrebs Zelllinie T47D eingesetzt. Um zytotoxische Wirkungen der Abwasserproben auf die Reporter-Zellen auszuschließen, wurden alle Proben zunächst steril filtriert und wo notwendig entsprechend verdünnt. Mittels Neutral-Rot-Aufnahme Assay konnten mögliche in vitro Zytotoxizitäten ausgeschlossen werden, um damit im nachfolgenden Östrogen-Assay tatsächlich nur hormonelle Wirkungen der Abwasserproben zu bestimmen. Die Reproduzierbarkeit und Sensitivität des T47D-Kbluc-Assays wurde an Abwasserproben optimiert, bevor in einer ersten Untersuchungsreihe von 7 Kläranlagen in Österreich die Mengen an Substanzen mit Östrogen-ähnlicher Wirkung nach der Abwasserreinigung bestimmt wurden. In der zweiten Untersuchungsreihe wurde die Effizienz des Abbaus von Östrogen-ähnlichen Stoffe der Versuchskläranlage untersucht. Damit ist der Assay als Screening Methode für Abwasserproben einsetzbar.

P71. Beeinflussen unterschiedliche Lagerungsbedingungen die endokrine Aktivität von Abwasserproben?

Ostermann Sina, Goethe-Universität Frankfurt am Main

Sabrina Giebner und Jörg Oehlmann

sina.ostermann@stud.uni-frankfurt.de

In konventionellen Kläranlagen werden Endokrine Disruptoren oft nicht oder nur unzureichend abgebaut und gelangen daher mit dem gereinigten Abwasser in die Gewässer. Obwohl negative Wirkungen von hormonaktiven Substanzen auf Organismen im Labor in zahlreichen Publikationen bereits beschrieben wurden, sind die ökologischen Folgen für aquatische Ökosysteme bisher noch unzureichend untersucht. Aus diesem Grund widmen sich zahlreiche Projekte der Untersuchung endokriner Effekte im Abwasser und Oberflächenwasser mit Hilfe von In-vivo-Tests und In-vitro-Assays. Bei der Analyse entsprechender Proben ist eine Lagerung nach der Probenahme bis zur nachfolgenden Untersuchung oft unvermeidlich. Da nicht davon ausgegangen werden kann, dass alle hormonaktiven Substanzen während der Probenlagerung stabil bleiben, sind die Fragen der optimalen Probenaufbereitung für die nachfolgende Lagerung, der geeignetsten Lagerungsmethode und des maximal möglichen Lagerungszeitraums von entscheidender Bedeutung, um endokrine Aktivitäten in den Proben verlässlich erfassen zu können.

Vor diesem Hintergrund wurden Aliquots mehrerer Originalproben nach Lagerung unter verschiedenen Bedingungen über einen definierten Zeitraum in Hefereportergen-Assays getestet, um eine Aussage über die Probenstabilität treffen zu können. Dafür wurden native Zulaufproben verschiedener Kläranlagen bei 4°C und –20°C über mehrere Wochen gelagert und in wöchentlichen Abständen auf ihre (anti-)östrogene und (anti-)androgene Aktivität getestet. Um verschiedene Probenaufbereitungsmethoden miteinander vergleichen zu können, wurden die Proben weiterhin direkt nach der Probenahme angesäuert, filtriert und ebenfalls in wöchentlichen Abständen getestet.

Erste Ergebnisse der unterschiedlichen Lagerungs- und Aufbereitungsarten werden im Beitrag vorgestellt und diskutiert.

Mikroplastik in der aquatischen Umwelt

P72. Bestimmung der Mikroplastikfracht in unterschiedlichen Reinigungsstufen der Kläranlage Frankfurt Niederrad

Hohoff Marie-Kristin, Goethe-Universität Frankfurt am Main

Christian Scherer(1), Nicole Brennholt(2), Georg Reifferscheid(2) und Martin Wagner(1) (1)Goethe Universität Frankfurt am Main, Aquatische Ökotoxikologie, Max-von-Laue-Straße 13, 60323 Frankfurt am Main (2)Bundesanstalt für Gewässerkunde, Referat Biochemie Ökotoxikologie, Am Mainzer Tor 1, 56068 Koblenz

marie.hohoff@freenet.de, c.scherer@bio.uni-frankfurt.de

Durch ihre hohe Beständigkeit und vielseitigen Einsatzmöglichkeiten werden Kunststoffe weltweit in immer größeren Mengen produziert. Mehrere Studien belegen das Vorkommen von Mikroplastik im Sediment und im Freiwasser diverser Meere und Küstenabschnitte [1].

Flüsse, die Mikroplastik aus dem Inland transportieren gelten als Haupteintragspfad von Mikroplastik in die Meere. Es ist anzunehmen, dass hierbei nicht nur unsachgemäße Müllentsorgung und Fischerei eine Rolle spielen, sondern auch die unzureichende Reinigung von häuslichen und industriellen Abwässern [2].

Um die Effektivität der Abwasserreinigung und im Speziellen die Effektivität einzelner Reinigungsstufen im Hinblick auf die Entfernung von Mikroplastik zu untersuchen, werden Wasserproben der Kläranlage Frankfurt Niederrad (1,3 Mio. Einwohnerwerte) analysiert. Mit Hilfe einer Pumpe werden nach fünf unterschiedlichen Reinigungsstufen (Sandfang, Vorklärung, Zwischenklärung, Nachklärung, Festbettdenitrifikation) bis zu 1000 L Abwasser über einen Kerzenfilter (Maschenweite von 80 µm) aufkonzentriert. Nach einem enzymatischen Verdau des Filterrückstands werden die potentiellen Plastikpartikel quantifiziert und spektroskopisch identifiziert.

Anhand der generierten Daten zur Mikroplastikfracht können die Reinigungsleistung der einzelnen Klärstufen analysiert und etwaige Methoden zur Verbesserungen der Klärleistung diskutiert werden.

Literatur:

1. Barnes D, Galgani F, Thompson RC, Barlaz M (2009) Accumulation and fragmentation of plastic debris in global environments. Philosophical transactions of the Royal Society of London. Series B, Biological sciences 364 (1526): 1985–1998.

2. Wagner M, Scherer C, Alvarez-Muñoz D, Brennholt N, Bourrain X et al. (2014) Microplastics in freshwater ecosystems: what we know and what we need to know. Environ Sci Eur 26 (1): 12.

P73. Sind Kläranlagen relevante Eintragspfade von Mikroplastik in aquatische Ökosysteme?

Parsch Christoph, Johann Wolfgang Goethe Universität Frankfurt am Main

Christian Scherer[1], Nicole Brennholt[2], Georg Reifferscheid[2] und Martin Wagner[1] [1] Goethe Universität, Aquatische Ökotoxikologie, Max-von-Laue-Strasse 13, 60323 Frankfurt am Main [2] Bundesanstalt für Gewässerkunde, Referat Biochemie Ökotoxikologie, Am Mainzer Tor 1, 56068 Koblenz

christophparsch2000@yahoo.de

Die Belastung der Umwelt durch Mikroplastik rückt mehr und mehr in den Fokus wissenschaftlicher Forschung. Auf der Suche nach den Quellen für Mikroplastik in marinen Ökosystemen wurden in früheren Studien Kläranlagen als Eintragspfade herausgestellt, die damit auch zu einer direkten Belastung limnischer Ökosysteme beitragen können.

Um die Rolle von Kläranlagen als Eintragspfade für Mikroplastik in die Umwelt zu charakterisieren, wurden in der vorliegenden Studie fünf Kläranlagen verschiedener Größenklassen mit Kapazitäten von 250 bis 1,35 Millionen Einwohnerwerten untersucht. Es wurden jeweils 540 bis 1000 L Wasser aus dem Ablauf der Kläranlagen entnommen und unter Einsatz einer Pumpe über einen Kerzenfilter mit einer Porengröße von 80 µm geleitet. Die aus organischem und anorganischem Material bestehenden Filterrückstände wurden enzymatisch verdaut, um die organischen Bestandteile zu reduzieren und die Identifizierung potentieller Mikroplastikpartikel zu erleichtern. Mit Hilfe spektroskopischer Methoden werden derzeit die Mikroplastikpartikel in den entsprechend behandelten Proben identifiziert und quantifiziert. Die Bestimmung der Anzahl an Plastikpartikeln und die qualitative Erfassung der Polymertypen in den Abläufen der Kläranlagen erlauben eine differenzierte Abschätzung über das Ausmaß des Eintrags in Abhängigkeit von der Größe der untersuchten Kläranlagen. Somit könnten die Daten einen wichtigen Beitrag zur Ermittlung von Eintragspfaden und relevanten Quellen von Mikroplastikpartikeln in aquatischen Ökosystemen leisten.

P74. Mikroplastik in den Ozeanen – Was bislang über Vorkommen und biologische Konsequenzen bekannt ist

Frey Sylvia, OceanCare

sfrey@oceancare.org

Plastikmüll gelangt zum grössten Teil vom Land über Flüsse, Abwasser, Wind, Überschwemmungen und direkt vom Ufer her in die Ozeane. Ein geringerer Teil des Plastikmülls im Meer stammt von Schiffen. Gemäss neusten Schätzungen werden durch 192 Küstenländer 275 Millionen Tonnen an Plastikabfall generiert, wovon im Durchschnitt 8,8 Millionen Tonnen in die Ozeane gelangten. Ohne Gegenmassnahmen wird sich die Menge des in die Ozeane eingespülten Plastiks innert 10 Jahren verzehnfachen.

Genau wie Makroplastik findet sich Mikroplastik auch an weit entlegenen Orten, fernab jeglicher Zivilisation in allen Ozeanen von der Antarktis bis in die Arktis, an Stränden, an der Wasseroberfläche, in der Wassersäule und am Meeresgrund. Aktuellen Schätzungen zufolge befinden sich weltweit rund 5,25 Billionen Mikroplastikpartikel in oberflächennahen Wasserschichten in den Ozeanen. Die zugrunde liegenden Modellrechnungen zeigten jedoch auch, dass es wesentliche Mikroplastik-Senken in den Ozeanen geben muss wie zum Beispiel Ablagerung in Sedimenten und Aufnahme durch Organismen.

Mittlerweile gibt es zahlreiche Publikationen, die zeigen, dass Mikroplastikpartikel von Vögeln, Meeressäugern, Meeresschildkröten, Fischen und diversen Invertebraten (Wirbellosen) wie Muscheln, Krebstieren, Würmern und filtrierenden Planktonorganismen aufgenommen werden. Zudem wurde nachgewiesen, dass sich auf Mikroplastikpartikeln im Wasser gelöste Schadstoffe wie Pestizide

und polychlorierte Biphenyle (PCB) in hoher Konzentration anreichern können.

Nichtregierungs-Organisationen (NGOs) spielen nebst universitären Instituten eine wichtige Rolle bei der Erforschung der Abundanz, Zusammensetzung und Effekte des Mikroplastiks in den Ozeanen. Obwohl bereits wichtige Forschungsresultate vorliegen, gibt es noch viele offene Fragen, insbesondere zu den Folgen von Mikroplastik und den im und am Plastik vorhandenen Schadstoffen für die verschiedenen marinen Organismen und der Verbreitung des Mikroplastiks und der Schadstoffe entlang der Nahrungskette.

Abwasserbehandlung und –toxizität

P75. Environmental and economic profiles of powdered activated carbon adsorption with ultrafiltration by polymeric and ceramic membranes to remove micropollutants from pre-treated wastewater

Oberschelp Christopher, University of Applied Sciences and Arts Northwestern Switzerland (FHNW), School of Life Sciences, Institute for Ecopreneurship and RWTH Aachen University, School of Business and Economics

Thomas Gross, University of Applied Sciences and Arts Northwestern Switzerland (FHNW), School of Life Sciences, Institute for Ecopreneurship, Gründenstrasse 40, 4132 Muttenz, Switzerland Anna Kounina, Quantis, Parc Scientifique EPFL, Bâtiment D, 1015 Lausanne, Switzerland Arnaud Dauriat, Quantis, Parc Scientifique EPFL, Bâtiment D, 1015 Lausanne, Switzerland Christian Remy, Berlin Centre of Competence in Water (KWB), Cicerostrasse 24, Berlin 10709, Germany Kristina Wencki, IWW Water Center, Moritzstrasse 26, 45476 Mülheim an der Ruhr, Germany Andreas Hein, IWW Water Center, Moritzstrasse 26, 45476 Mülheim an der Ruhr, Germany Aurélie Gallice, Veolia, Environmental and Socio-Economics Group, Research Center of Maisons-Laffitte, Chemin de la digue, 78603 Maisons-Laffitte, France Thomas Wintgens, University of Applied Sciences and Arts Northwestern Switzerland (FHNW), School of Life Sciences, Institute for Ecopreneurship, Gründenstrasse 40, 4132 Muttenz, Switzerland Christoph Hugi, University of Applied Sciences and Arts Northwestern Switzerland (FHNW), School of Life Sciences, Institute for Ecopreneurship, Gründenstrasse 40, 4132 Muttenz, Switzerland

christopher.oberschelp@rwth-aachen.de

Several technologies to degrade and/or remove micropollutants (MPs) found in wastewater treatment plant (WWTP) effluents exist. These include oxidative treatment such as ozonation and adsorption using activated carbon. Such additional treatment steps reduce local MP discharge and hence eco- and human toxicological hazards. However, additional resource requirements including costs are considerable. To compare the benefits and costs of these technologies for decision support a combination of an environmental Life Cycle Analysis (LCA) and an economic Life Cycle Costing (LCC) of a pilot-scale powdered activated carbon adsorption with subsequent ultrafiltration (PAC/UF) by polymeric or ceramic membranes treating secondary effluent at a municipal WWTP in Switzerland are presented. Ceramic membranes have gained attention due to their mechanical strength, higher permeability and longer lifetime compared to polymeric membranes. Their application has however been limited due to relatively high investment costs. We estimated environmental benefits from the removal of five MPs measured at the WWTP, i.e. sulfamethoxazole, carbamazepine, mecoprop, diclofenac and benzotriazole, and in addition from MPs commonly detected in Swiss wastewater. Recent studies at the same WWTP show that PAC/UF at PAC dosages of ca. 20 mg·L-1 remove considerable fractions of MP concentrations typically found in secondary effluent. Our LCA indicates that aquatic ecotoxicity and human non-cancer toxicity of the effluent could be reduced by about 75% with full-scale PAC/UF post treatment. These substantial benefits are however accompanied by off-site CO2 emissions considerably increasing global warming potential (GWP) of the current WWTP. This is mainly due to PAC production and membrane aeration applied in membrane operation, causing 68% and 17%

of the GWP increase, respectively. Due to longer lifetime of ceramic membranes, specific treatment costs do not significantly differ between polymeric and ceramic membranes with ca. 0.30 CHF·m-3 wastewater. In conclusion, a PAC/UF system reduces eco- and human toxicity of effluents with environmental and financial trade-offs. Further research should aim to minimize PAC dosing and consider alternative adsorbents, renewable PAC raw materials as well as membrane operation modes without aeration. An urban water cycle perspective in LCA/LCC can help taking into account the reduction of MPs at source to minimize end-of-pipe treatment requirements.

P76. Online Biomonitoring at WWTPs: emission vs. immission approach

Gerhardt Almut, LimCo International GmbH
Rastetter N., Hofer M., Kuch B., Bühler C. und Di Lorenzo T.

almutg@web.de

Urban waste water treatment plants face the challenge to provide an optimal purification process within a few hours, independent of varying concentrations and composition of the chemical substance cocktail in the incoming water. Environmental legislation in Europe and Switzerland require the reduction of emissions of selected priority substances and their toxic potential in order to protect the aquatic biota in the receiving stream. Actually there is a broad discussion to establish advanced purification steps in WWTPs as measure to reduce toxicity.

In this context the WWTP Limmattal is pioneer in establishing a real-time based online biomonitoring system to monitor the biological responses of ecologically relevant aquatic organisms, Gammarus fossarum, (saproby water class II indicator) in both the effluent (emission control) and in the Limmat stream (immission control). The system has been operated successfully for about 2 1/2 yrs.

In this poster we present the results of the comparison of the toxic potential in the effluent versus the Limmat receiving stream regarding survival and behavior of Gammarus fossarum based on longterm monitoring data.

P77. Online Biomonitoring at WWTPs: before and after advanced PAK-step

Gerhardt Almut, LimCo International GmbH
Rastetter N., Metzger S., Schönung J., Schymiczek K., Hein A.

almutg@web.de

Urban waste water treatment plants face the challenge to provide an optimal purification process within a few hours, independent of varying concentrations and composition of the chemical substance cocktail in the incoming water. Environmental legislation in Europe requires the reduction of emissions of selected priority substances and their toxic potential in order to protect the aquatic biota in the receiving stream and achieve good water quality status according to WFD.Several WWTPs test advanced purification technologies.

For the first time ever, we tested a real-time based online biomonitoring system to monitor the biological responses of ecologically relevant aquatic organisms, Gammarus fossarum, (saproby water class II indicator) before and after a PAK purification step in the WWTP Mannheim Sandhofen.

During our short-term pilot run of the Multispecies Freshwater Biomonitor (MFB) we found Gammarus fossarum to be positively affected by the PAK purification in terms of better survival, higher locomotory activity and less stress ventilation behavior. Further studies to back up these preliminary results are planned.

P78. Recycling of phosphorus from sewage sludge in agriculture: Eco-toxicological assessment with aquatic and soil indicator species

Rastetter Nadja, LimCo International GmbH
Gerhardt A.

n_rastetter@web.de

Sewage sludges contain substantial concentrations of phosphorus, which represents an essential but limited resource for plant growth. But even heavy metals and organic pollutants like pharmaceuticals, pesticides etc. are components of the so-called biosolids, therefore new technologies for recycling of phosphorus from sewage sludge are necessary. For recycling of phosphorus as a fertiliser (crystalised or thermally treated P-recyclates) in agriculture, the toxic potential on target species of affected environmental compartments (soil, water, sediment) has to be tested. The direct acute toxic effects of the P-recyclates and a reference fertiliser Triple-Superphosphate TSP were assessed with 1) the duckweed Lemna minor by growth inhibition, decrease of biomass and growth length 2) the freshwater shrimp Gammarus fossarum by mortality and behaviour (movement activity and feeding behaviour) 3) the tiger worm (Eisenia fetida) by its avoidance behaviour. Additionally, the eluates of the two most effective products in the direct assessment of ecotoxicity were tested by G. fossarum, the most sensitive species in this study.

Low and relevant concentrations of TSP could have a negative effect on G. fossarum (0.6 g DM kg^{-1}). Applicable concentrations of the P-recyclates had no negative effect on the tested target organisms. Only high concentrations (≥ 5 g DM kg^{-1}; ≥ 5 g DM l^{-1}) of the crystalised P-recyclates resulted in increased mortality and/or behavioural changes and growth inhibition. Recyclates from thermally treated sludge or ash had even low toxic effects at high concentration levels. Their eluates had a lower or equal effect on the gammarids.

Applicable concentrations of the tested products in agriculture, calculated by the P2O5-content, had no negative effect on the test species under laboratory conditions. Re-using the thermally treated materials should be uncritical. For excluding negative effects in the field testing of environmental samples are suggested. The research project P-REX is supported by the European Commission under the Seventh Framework Programme (Priority: “From Prototype to Market”/ Contract No. 308645).

P79. Ökotoxikologische Untersuchungen des Einflusses einer Abwasserozonung auf den Gewässerzustand der Wurm im Rahmen des Projektes DemO3AC

Schiwy Sabrina, Institut für Umweltforschung- RWTH-Aachen

Sibylle Maletz; Yvonne Müller; Sarah Könemann; Ira Brückner; Hermann Stepkes; Monika Hammers-Wirtz; Regina Dolny; Kassandra Klaer; Wilhelm Gebhardt; Volker Linnemann; Johannes Pinnekamp und Henner Hollert

s.schiwy@bio5.rwth-aachen.de

Die Europäische Wasserrahmenrichtlinie schreibt als zu erreichendes Qualitätsziel den guten ökologischen Zustand von Gewässern bis zum Jahr 2015 vor. Nur 20 % der deutschen Oberflächengewässer konnten das Ziel bisher erreichen. Eine mögliche Ursache könnte sein, dass eine Vielzahl von anthropogenen Spurenstoffen in Kläranlagen nicht vollständig eliminiert wird und so in die aquatische Umwelt gelangt. Aus diesem Grund soll im Rahmen des Projekts „Demonstrationsvorhaben Ozonung des Abwassers auf der Kläranlage Aachen-Soers“ (DemO3AC) eine großtechnische Abwasserozonung als weitere Reinigungsstufe in den Klärprozess integriert werden. Gefördert wird das Projekt durch das Ministerium für Klimaschutz, Umwelt, Landwirtschaft, Natur- und Verbraucherschutz des Landes Nordrhein-Westfalen.

Neben der grundsätzlichen Eignungsprüfung und der Evaluierung von Optimierungsmöglichkeiten liegt der Fokus der begleitenden Untersuchungen vor allem auf dem Einfluss der Abwasserozonung auf die Wurm, die der Kläranlage als Vorfluter dient. Die Wurm führt bei Mittel- und Niedrigwasserabfluss ca. 70 % behandeltes Abwasser. Zunächst wird der Status-quo des Gewässers anhand verschiedener Analysen erhoben und anschließend mit den Daten nach Inbetriebnahme der großtechnischen Ozonung verglichen. Eine umfassende chemische Analyse der Spurenstoffe wird durch das Institut für Siedlungswasserwirtschaft der RWTH Aachen sowohl vor als auch nach Inbetriebnahme der Ozonungsanlage durchgeführt. Zudem sollen Untersuchungen zur mikrobiologischen Zusammensetzung und zur Antibiotikaresistenz durch das Institut für Angewandte Mikrobiologie der RWTH durchgeführt werden.

Ein wichtiger Teilaspekt des Projekts besteht in der ökotoxikologischen Bewertung der Wurm, das in dem Posterbeitrag konzeptionell vorgestellt werden soll. Mit den standardisierten Testverfahren für die Abwasserbewertung nach DIN und ISO (Bakterien-, Algen-, Daphnien- und Fischembryotoxizität) können akute und chronische Wirkungen anthropogener Spurenstoffe auf verschiedenen trophischen Ebenen untersucht werden. Zudem sollen zelltoxikologische Testsysteme eingesetzt werden, um die estrogene Aktivität, Effekte auf die Steroidgenese und die mutagene Wirkung des Abwassers zu untersuchen. Auf Ebene der Organismen sind Untersuchungen in vivo und in situ mit der Regenbogenforelle (Oncorhynchus mykiss), der Zwergdeckelschnecke (Potamopyrgus antipodarum) und dem Bachflohkrebs (Gammarus spec.) geplant. Alle genannten ökotoxikologischen Testverfahren werden vor Inbetriebnahme der Ozonungsanlage durchgeführt. Anschließend soll evaluiert werden, welche der Biotests sich generell für die Testung der Abwasserspurenstoffe eignen. Diese werden dann nach Inbetriebnahme der Ozonungsanlage angewandt und mit den vorhandenen Daten vergleichen. Erste Ergebnisse werden im Herbst 2015 erwartet.

P80. Effects of ozonation with different post-treatments on the early development of rainbow trout in a Swiss wastewater treatment plant

Thiemann Christina, University of Tübingen and Ecotox Centre Eawag-EPFL

Kienle Cornelia, Ganser Barbara, Schlüter-Vorberg Lisa, Vermeirssen Etienne, Weil Mirco, Werner Inge and Triebskorn Rita

mail@christina-thiemann.net

The presence of micropollutants in wastewater related to human lifestyles, as e.g. pharmaceuticals and personal care products, requires advanced wastewater treatment techniques to prevent environmental pollution. Previous studies have shown ozonation to be a promising technique to eliminate micropollutants, although reactive and potentially toxic transformation products can occur. Post-treatments like sand filtration, granulated activated carbon, fixed and turbulent fluidized beds are thus necessary and recommended e.g. by the Swiss Federal Office for the Environment (FOEN).

In the present study, effects of a full-scale ozonation with subsequent post-treatments on the early development of juvenile trout were assessed in a flow-through system. As post-treatments, a full-scale sand filtration and pilot-scale granulated activated carbon, fixed and turbulent fluidized bed were compared and assessed for their ability to remove biologically degradable transformation products from ozonation.

The standard test organism Oncorhynchus mykiss, an easy to rear, pollutant-sensitive organism, was used for experiments at the wastewater treatment plant Neugut in Dübendorf, the first in Switzerland to remove micropollutants using this combination of techniques. A 96-day Fish Early Life Stage Toxicity test was run according to OECD Guideline No. 210 with subsequent biomarker analyses including histopathological alterations in liver and gill as well as oxidative stress in liver and filet samples using the ferrous oxidation-xylenol orange assay.

There were no significant differences between treatments regarding pre-hatch survival, length and weight of individual fish. However, the endpoints hatch, swim-up and post-hatch survival showed significant differences between treatments, indicating a better suitability for some of the tested techniques. Whether a differentiation between the different wastewater treatment technologies is possible by biomarker analyses will be discussed.

This project received funding from the FOEN (project ReTREAT).

P81. Does wastewater treatment plant upgrading with activated charcoal filtering reduce endocrine and embryotoxic effects in fish?

Wilhelm Sabrina, Animal Physiological Ecology, University of Tuebingen
Henneberg Anja, Triebskorn Rita

sabrina_wilhelm@live.de

Aquatic organisms have to cope with a variety of anthropogenic substances, as e.g. pharmaceuticals or human care products, which are continuously released into the aquatic environment. In order to reduce the general surface water pollution, new wastewater treatment plant technologies have been developed during the last years. However, little is known about their efficiency with respect to ecosystem health.

The current work is integrated into the BMBF project “SchussenAktivplus", which examined the efficiency of different wastewater treatment plant technologies to reduce micropollutants and potentially pathogenic bacteria from surface waters and the resulting advantages for the ecosystem. For this purpose, chemical and microbiological analyses combined with biological effect studies were conducted prior and after the application of new technologies to several wastewater treatment plants and storm water overflow basins connected to the Schussen River, a major tributary of Lake Constance.

The present study focuses on endocrine and embryotoxic effects in trout two years after the upgrade of the wastewater treatment plant Langwiese (Ravensburg, Germany) with an additional activated charcoal filter. In order to assess these effects, vitellogenin levels in adult and juvenile fish exposed to surface water of the Schussen and the Argen River (as a reference site) were analyzed. Normally, the egg yolk precursor protein vitellogenin is only produced by mature female fish. However, vitellogenin content in male and juvenile trout can serve as a biomarker of exposure to estrogen-like substances in the environment. In order to examine embryotoxic effects, fertilized brown trout (Salmo trutta f. fario) and rainbow trout (Oncorhynchus mykiss) eggs were actively exposed to surface water of the two rivers, and mortality and hatching success were recorded. Overall, the results obtained indicate an impact of the additional activated charcoal filter stage on endocrine and embryotoxic effects in fish.

P82. Transkriptomanalyse des toxikogenomischen Potenzials einer komplexen Abwasserprobe sowie der Einfluss auf das lokomotorische Verhalten von Zebrabärbling-Embryonen

Quati Gina, RWTH-Aachen, Institut für Umweltforschung/ Helmholtz Zentrum für Umweltforschung, Leipzig

Leonie Nüßer1, Eberhard Küster2, Martin Krauss2 Thomas-Benjamin Seiler1 Werner Brack1,2 & Henner Hollert1 1 Institut für Umweltforschung, Rheinisch-Westfälisch-Technische-Hochschule Aachen 2 Department für bioanalytische Ökotoxikologie & wirkungsorientierte Analytik, Helmholtz Zentrum für Umweltforschung-UFZ, Leipzig

gina.quati@rwth-aachen.de

Durch Kläranlagenabläufe werden Xenobiotika in Oberflächengewässer eingetragen. Diese können einerseits deren Qualität beeinträchtigen, das Funktionieren des Ökosystems stören und andererseits über das Trinkwasser für den Menschen verfügbar werden. In dem vorliegenden Projekt wurde das toxikogenomische Potenzial eines Extrakts einer Abwasserprobe des industriellen Kläranlagenauslaufes der Gemeinschaftskläranlage in Bitterfeld-Wolfen untersucht. In einer chemischen Analyse wurden in dem Extrakt u.a. zwei Barbiturate (Pento- und Secobarbital) in Konzentrationen bis zu 215 µg/L detektiert. Im Fischembryotoxizitätstest (FET) wurden sodann Embryonen des Zebrabärblings (Danio rerio) für 120 h mit dem Abwasserextrakt exponiert. Eine Transkriptomanalyse zeigte dass mehr als 10% der Transkripte, im Vergleich zur Negativkontrolle, um ein Vielfaches hoch oder runter reguliert waren. Unter anderem waren Gene reguliert, die der „crystallin protein family“ zuzuordnen sind. Diese sind u.a. am Aufbau der Linse im Fischauge beteiligt. Ziel der Arbeit ist es nunmehr, die Substanzen der komplexen Abwasserprobe zu identifizieren, die für die Effekte auf das Transkriptom der Embryonen des Zebrabärblings verantwortlich sind. Dazu werden zunächst die Ergebnisse der Transkriptomanalyse mittels quantitativer real-time PCR (qPCR) verifiziert. Zusätzlich wurden Effekte auf das Bewegungsmuster gezeigt, die mittels photo-motor-response (PMR) Video-tracking beobachtet wurden. Erste Ergebnisse des Video-tracking zeigten, dass die mit dem Extrakt belasteten Zebrabärbling-Embryonen eine verminderte lokomotorische Aktivität besitzen. Die Verifizierung mittels qPCR soll anhand verschiedener Expositionsszenarien erfolgen. Die Auswertung der Transkriptomanalyse wies darauf hin, dass ein Großteil der Effekte auf dioxinähnlich wirkende Substanzen in der Abwasserprobe zurückzuführen sind. Die Wirkung dioxin-ähnlicher Substanzen ist AhR-vermittelt. Deswegen wurde eine Co-Exposition mit ß Naphtoflavone (ßNF), stellvertretend für Ahr-vermittelte Substanzen, durchgeführt. Sowohl der Abwasserextrakt wie auch ßNF wurden in verschiedenen Konzentrationen sowie in Kombination mit dem AhR Antagonisten α Naphtoflavone (ANF) getestet, um zu untersuchen, ob ein Teil der Effekte bei Blockierung des Ah-Rezeptors, ausbleibt. Um den Einfluss der Barbiturate auf die Veränderung im Transkriptom zu untersuchen, wird derzeit eine weitere Co-Exposition mit diesen durchgeführt.

Erstautoren/Presenter-Verzeichnis

Name	Vorname	Präsentation No.
**A**		
Aldrich	Annette	24
Axt	Florian	P43
**B**		
Bandow	Nicole	66
Baumann	Lisa	P26
Beauvais-Flueck	Rebecca	18, P14
Becker	Dennis	74
Becker	Benjamin	31
Beggel	Sebastian	21
Berger	Elisabeth	42
Bick	Vanessa	P11
Bischof	Ina	P28
Böhler	Svenja	12
Brettschneider	Denise	59
Brinke	Marvin	10
Brinke	Alexandra	P8
Brinkmann	Markus	9, P12, P23
Bruhn	Torben	P30
Buchinger	Sebastian	7
Burkhardt	Michael	2
**C**		
Campiche	Sophie	28, P7, P40, P41
Casado-Martinez	Carmen	P9
Caspers	Norbert	5
Chèvre	Nathalie	P55, P56
Chiaia-Hernandez	Aurea	27
Coors	Anja	P57

D

Di Paolo	Carolina	13, 20, P18
Dieterich	Andreas	P51
Duis	Karen	64
Dünne	Matthias	15
Düring	Rolf-Alexander	36

E

Ernst	Johannes	P60
Eschenbach	Eva	23, P36
Eser	Sabine	P15

F

Faure	Florian	67
Feiner	Mona	21
Fettig	Ina	P44
Fischer	Stephan	16
Fischer	Fabian	33
Frey	Sylvia	P74
Fulda	Beate	46, P61

G

Galay Burgos	Malyka	P3
Gattiker	Etienne	P67
Gauch	Roger	P62
Geiss	Cornelia	63
Gerhardt	Almut	P76, P77
Giebner	Sabrina	72
Gräf	Tonya	P35

H

Hartmann	Jason	14
Hartmann	Sarah	P32
Heyde	Benjamin	P45
Heye	Katharina	44
Hochstrasser	Florian	P2

Hohoff	Marie-Kristin	P72
**J**		
Jacob	Stefanie	P54
Jagodzinski	Lucas S.	22
Janz	Patricia	P13
Jeckel	Nina	P68
Jundhans	Marion	39
**K**		
Kattwinkel	Mira	43
Kaufmann	Katharina	25
Kaufmann	Kristina	P69
Keil	Benjamin M.	P49
Kienle	Cornelia	40, 70
Kirla	Krishna Tulasi	58
Knauer	Katja	45
Knillmann	Saskia	49
Knöbel	Melanie	P29
Koglin	Sven	19
Kohler	Esther	P63
König	Maria	41
Könnecker	Gustav	P17
Korkaric	Muris	P66
Krauskopf	Lisa-Marie	P46
Kroller	Theophil	P70
Kropf	Christian	P27
Kunz	Petra Y.	P5
**L**		
Langer	Miriam	P53
Lautenschlager	Karin	P64
Lehnik-Habrink	Petra	P47
Liess	Matthias	53
Lorenz	Carla S.	P58

M

Märkl	Veronika	4
Mathis	Marcel	48
Mieck	Susanna	P19
Molano-Leno	Lidia	P10
Möller	Anja Maria	P25
Moschet	Christoph	P42

O

Oberschelp	Christopher	P75
Obert-Rauser	Patrick	P16
Ochsenbein	Ueli	38
Ostermann	Sina	P71

P

Parsch	Christoph	P73
Perseke	Lea	51
Pillai	Smitha	57

Q

Quati	Gina	P82
Quesada-Garcia	Alba	17

R

Rastetter	Nadja	P78
Rehberger	Kristina	60, P33
Richter	Janine	P50
Rösch	Andrea	P59
Ross-Nickoll	Martina	47

Sch

Schäfer	Ralf B.	50
Schäffer	Andreas	34
Schindler	Yael	6
Schinkel	Lena	35
Schiwy	Sabrina	11, P79
Schlotz	Nina	62

Schmieg	Hannah	P21
Schoknecht	Ute	1, P1
Schönborn	Andreas	61
Schwarz	Simon	P20
**S**		
Segner	Helmut	54
Seidensticker	Sven	P22
Siemoneit-Gast	Sandra	P37
Simon	Eszter	P6
Svojitka	Jan	73
**Sp**		
Spaak	Piet	55
Spann	Nicole	29
St		
Storck	Florian R.	65
Straub	Lars	56
Stravs	Michael	P48
Strobel	Anneli	P65
**T**		
Teichler	Rebekka	69
Tellenbach	Christoph	55
Thiemann	Christina	P80
Tierbach	Alena	P24
Tlili	Ahmed	32
Treidy	Stephan	P31
Triebskorn	Rita	71
**V**		
Vallotton	Nathalie	52
Vermeirssen	Etienne	3, 8, P4
von Siebenthal	Elena Wernicke	P34

W

Wächter	Daniel	26
Wilcsek	Kristiana	P52
Wilhelm	Sabrina	P81
Wittmer	Irene	39
Wollmann	Claudia	P38, P39
Wunderlin	Pascal	68

Z

Zennegg	Markus	30, 37
